# Nutrient Inputs to the Laurentian Great Lakes by Source and Watershed Estimated Using SPARROW Watershed Models^1^

**DOI:** 10.1111/j.1752-1688.2011.00574.x

**Published:** 2011-10

**Authors:** Dale M Robertson, David A Saad

**Keywords:** watershed modeling, Great Lakes, nutrients, streams, nonpoint-source pollution, point-source pollution

## Abstract

**Abstract:**

Nutrient input to the Laurentian Great Lakes continues to cause problems with eutrophication. To reduce the extent and severity of these problems, target nutrient loads were established and Total Maximum Daily Loads are being developed for many tributaries. Without detailed loading information it is difficult to determine if the targets are being met and how to prioritize rehabilitation efforts. To help address these issues, SPAtially Referenced Regressions On Watershed attributes (SPARROW) models were developed for estimating loads and sources of phosphorus (P) and nitrogen (N) from the United States (U.S.) portion of the Great Lakes, Upper Mississippi, Ohio, and Red River Basins. Results indicated that recent U.S. loadings to Lakes Michigan and Ontario are similar to those in the 1980s, whereas loadings to Lakes Superior, Huron, and Erie decreased. Highest loads were from tributaries with the largest watersheds, whereas highest yields were from areas with intense agriculture and large point sources of nutrients. Tributaries were ranked based on their relative loads and yields to each lake. Input from agricultural areas was a significant source of nutrients, contributing ∼33-44% of the P and ∼33-58% of the N, except for areas around Superior with little agriculture. Point sources were also significant, contributing ∼14-44% of the P and 13-34% of the N. Watersheds around Lake Erie contributed nutrients at the highest rate (similar to intensively farmed areas in the Midwest) because they have the largest nutrient inputs and highest delivery ratio.

## Introduction

The Laurentian Great Lakes constitute the largest freshwater system in the world, and have nearly 25% of the United States (U.S.) and Canadian populations in their watersheds. The Great Lakes receive water and accompanying nutrients from many tributaries draining areas ranging from pristine forests, to intensively farmed areas, to large urban centers, and nutrient input from these tributaries is extremely variable (Sonzogni *et al.*, 1978; Rathke and McRae, 1989; Robertson, 1997). This nutrient loading (input or mass over a specified period of time) has caused eutrophication to various degrees and scales, from small and large bays around the Great Lakes (e.g., Green Bay in Lake Michigan) to wide-scale eutrophication in some of the Great Lakes themselves (e.g., Lake Erie) (Sonzogni *et al.*, 1979). Several researchers have reported on the effects of eutrophication in the Great Lakes, including excessive growth of planktonic and attached algae (cladophera) (Schultze, 2005), turbidity, changes in biotic composition, undesirable taste and odor, and promotion of anoxic conditions to various degrees (IJC, 1970; Burns and Ross, 1972; Nurnberg, 1995).

As a result of degradation in the water quality in the Great Lakes in the 1960s, Canada and the U.S. signed the Great Lakes Water Quality Agreement (GLWQA) in 1972. The overall goal of the Agreement was to restore and maintain the physical, chemical, and biological integrity of the Great Lakes. In addition to identifying lake-wide problems, an assessment by the International Joint Commission (IJC) Water Quality Board in 1973 listed 42 Areas of Concern (AOC) and identified the beneficial use impairments, including such issues as fish advisories, toxic contamination, degraded aquatic communities, and eutrophication (Freedman and Monson, 1989). Because of the eutrophication issues, the 1972 GLWQA agreement was renewed in 1978, and identified phosphorus (P) as the nutrient of primary concern in the Great Lakes, and defined target P loads for each lake; a P-load reduction supplement was later signed in 1983. Although P has been identified as the nutrient of primary concern for most areas of the Great Lakes, nitrogen (N) has been identified as a factor correlated with the biological integrity of Midwest streams (Wang *et al.*, 2007). Under recommendations of the Clean Water Action Plan of 1998, the U.S. Environmental Protection Agency (USEPA) developed a national strategy to reduce concentrations of P and N and improve the beneficial ecological uses of surface waters of the U.S. by establishing waterbody-specific nutrient criteria for rivers and streams, wetlands, lakes (including the Great Lakes) and reservoirs, and estuaries (USEPA, 1998). Although reductions in loading have reduced most open-lake eutrophication problems, except for Lake Erie (R. Kreis, USEPA, 2004, written communication), eutrophication problems are still common in many nearshore areas (cladophera) (Schultze, 2005) and embayments (DePinto *et al.*, 2006). In 2004, the Great Lakes Interagency Task Force was established to develop a restoration plan for the Great Lakes and coordinate restoration efforts, including efforts to reduce nutrient pollution. In 2010, the Great Lakes Restoration Initiative was implemented to target the most significant problems, including nonpoint-source pollution, and track progress in addressing them. Therefore, to achieve the in-lake nutrient standards under consideration for the Great Lakes and its embayments, nutrient loads must be reduced.

Detailed water-quality and streamflow data are required to determine the status of nutrient loading to each Great Lake, where and from what sources those loads originate, and where and what kinds of actions would be most beneficial to reduce nutrient input to the Great Lakes. Nutrient loadings to the entire Great Lakes system were initially estimated as part of the IJC efforts, in which the Pollution from Land Use Activities Reference Group (PLUARG) estimated nutrient and suspended solids loadings for 1975 and 1976 (Sonzogni *et al.*, 1978). Those loadings, which were estimated for 43 monitored tributaries during 1975 and 110 monitored tributaries during 1976, were then used to compute yields (mass per unit area per time) for each monitored tributary. The unit-area yields were then applied to nearby unmonitored tributaries to estimate their contributions and the total loads of nutrients to each Great Lake from the U.S. and Canadian areas and to each lake as a whole. The IJC used the PLUARG approach to continue making annual regionalized estimates of P loading to each Great Lake on the basis of data collected from key tributaries until 1991 (Rathke and McRae, 1989; Lesht *et al.*, 1991; D. Dolan, IJC, Great Lakes Regional Office, Windsor, Ontario, 1995, oral communication). These studies found that management actions in the watershed did reduce the loading to all of the Great Lakes, with the possible exception of Lake Ontario (R. Kreis, USEPA, 2004, written communication). Beginning in the early 1990s, the number of key tributaries around the Great Lakes being monitored by the U.S. Geological Survey's (USGSs) National Stream Quality Accounting Network began to decrease (Alexander *et al.*, 1998). As a consequence, total loading to each lake had to be estimated based on data from the fewer remaining monitored sites, each of which now represented much larger parts of the watersheds. Because of the extensive extrapolations needed from the few remaining sites, estimations of annual P loading were continued only for Lake Erie (D. Dolan, University of Wisconsin-Green Bay, 2005, written communication). Limited loading estimates have been made as part of short-term, large-scale loading studies, such as the Lake Michigan Mass Balance Study (Rossmann, 2006), but are available for only a few lakes. Reduction in monitoring in key tributaries not only makes it difficult to evaluate nutrient loading conditions for these tributaries, it also makes it difficult to determine whether or not the target loads are still being met.

Approaches have been developed for ranking basins and targeting management as part of Total Maximum Daily Load (TMDL) activities (e.g., Fox River Basin TMDL) (WDNR, 2009), TMDL-like activities (e.g., Saginaw Bay Phosphorus Reduction Strategy) (MDNR, 1991), and studies to implement specific practices at an individual state scale (Tomer *et al.*, 2003), but few studies have targeted specific watersheds at broad geographic scales, such as the entire Great Lakes Basin. Richards (1989) described a few approaches to estimate loading from unmonitored or partially monitored areas needed for complete basinwide estimates and basin ranking. A few large-scale approaches/models developed for these purposes near the Great Lakes include: (1) unit-area-yield methods for estimating loads from unmonitored rivers from specific monitored rivers, which were applied to Great Lakes from late 1970s to 1990s (PLUARG approach) (Sonzogni *et al.*, 1978; Rathke and McRae, 1989; Lesht *et al.*, 1991; Robertson, 1997), (2) a GIS-based erosion model for the Great Lakes tributaries developed to assess and compare their relative loadings of sediments, status of conservation practices, and their potential for further reductions in sediment and contaminant loadings (Ouyang *et al.*, 2005), and (3) a predictive GIS-based model developed by Diebel *et al.* (2009) to estimate sediment and P load reduction that should be achievable following installation of riparian buffers in basins throughout Wisconsin. Results of this latter model were used to rank all of the basins in Wisconsin based on their potential load reductions.

Reduction in monitoring of the key tributaries not only makes it difficult to evaluate nutrient conditions in these tributaries, it also reduces the certainty in whether total target loads are still being met (basinwide load estimation) and in the origins of the nutrients. Previous modeling approaches used in the Great Lakes region also do not enable the evaluation and comparison of all of the basins (basin ranking/targeting), nor do they enable the main sources of nutrients to be identified (source apportionment). A principal weakness in the previous modeling approaches is that they do not explicitly describe the locations in watersheds where long-term storage or permanent removal (e.g., through denitrification) of nutrients occur during transport, which complicates attempts to identify the upstream sources that contribute to the total load in downstream receiving waters.

Recent research has led to the development of a spatially explicit model (SPAtially Referenced Regressions On Watershed attributes, or SPARROW) for describing the sources, transport, and fate of nutrients in watersheds (Smith *et al.*, 1997). This modeling approach addresses many of the limitations of the modeling techniques that had been previously used to estimate tributary loads to the Great Lakes. SPARROW uses a process-based mass-balance approach with a spatially detailed digital network of streams and reservoirs to track the attenuation of nutrients during their downstream transport from each source, and is calibrated using statistical techniques. SPARROW assessments of nutrient sources, transport, and delivery to coastal waters have been completed for the entire U.S. (Smith *et al.*, 1997; Alexander *et al.*, 2008), and the highest nutrient yields were found to be from watersheds in the Midwest (Iowa, Illinois, Indiana, and western Ohio), parts of which drain to the Great Lakes. SPARROW models also have been developed for specific regions of the U.S. including the Chesapeake Bay watershed (Preston and Brakebill, 1999), and the southeastern part of the U.S. (Hoos and McMahon, 2009). Results from SPARROW models also have been used to rank watersheds in the Mississippi River Basin based on delivery of nutrients to the Gulf of Mexico (Robertson *et al.*, 2009a). This approach accounted for uncertainties in the model predictions and enabled water-resource managers to assess the probability that a watershed belongs to a group of watersheds with the highest yields. The results from the ranking process have been used recently by the U.S. Department of Agriculture (USDA), along with other information, to target 41 large watersheds in the Mississippi River Basin where financial assistance will be provided for implementing various conservation practices (USDA, 2010).

**FIGURE 1:**
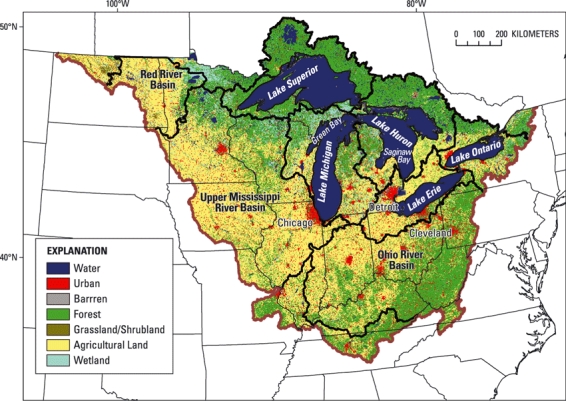
Land Use and Land Cover Across the Great Lakes Basin and Upper Midwest With Selected Metropolitan Centers Identified (U.S. drainage, USGS, 2000; Canadian Drainage, Geobase, 2009). Major River Basin #3 (MRB3) represents the U.S. portion of this area. All major basins are delineated.

In this paper, we describe P and N SPARROW models developed for the Upper Midwest (Great Lakes and Upper Mississippi, Ohio, and Red River Basins). This area is one of the eight large geographical regions across the U.S. (referred to as major river basins, or MRBs) identified by the National Water-Quality Assessment (NAWQA) Program of the USGS as the basis for assessments of status and trends. The NAWQA Program has integrated the SPARROW modeling approach in the interpretation of nutrient transport in six of these MRBs (Preston *et al.*, 2009). The Upper Midwest is referred to as MRB3 (Figure 1). Previous national SPARROW models applied to the Midwest were calibrated on the basis of data from 425 sites on relatively large rivers throughout the U.S. (median drainage-basin size of 10,500 km^2^); therefore, it was difficult to evaluate the ability of these models to estimate loads in small Midwestern streams. Models developed in the study described here are based on loading information from more than 700 sites estimated from water-quality data assembled from the major sampling agencies throughout MRB3. The use of information from watersheds throughout MRB3, which share land-use and climate characteristics similar to those in the Great Lakes Basin, increased the number of monitoring sites available for estimating nutrient sources and processes in the model (i.e., model calibration), thereby providing a more accurate model representation of the full range of conditions and water-quality processes within the Great Lakes Basin. Many of these sites are on much smaller streams than those included as input to previous national SPARROW models, thereby potentially enabling the model to better represent headwater areas within MRB3. The spatial variability in stream nutrient loading is accounted for by using watershed information (sources of nutrients and environmental characteristics) assembled for the MRB3 region, including variables unavailable for previous models, such as point-source contributions of nutrients. Results from the calibrated SPARROW models are used to: (1) determine P and N loads to each Great Lake (from the U.S. part of their basins), (2) determine the total P (TP) and total N (TN) load from each tributary draining more than 150 km^2^ to each Great Lake, (3) rank the individual tributaries to each lake based on their relative loading and yields, (4) determine the relative importance of each P and N source, and (5) determine which environmental factors significantly affect the delivery of P and N from the land to the streams in the Upper Midwest.

## Methods

### Study Area

SPARROW attributes models were developed for the U.S. part of the Upper Midwest (Great Lakes, Upper Mississippi River, Ohio River, and Red River Basins, referred to as MRB3 as shown in Figure 1). Land use and land cover in the region consists primarily of forested areas in the northern and southeastern parts and agricultural areas in the western and central parts. Several major metropolitan areas, including Chicago, Illinois, Detroit, Michigan, and Cleveland, Ohio, lie within the region. The Laurentian Great Lakes consist of five lakes linked by relatively short connecting channels. SPARROW models were only developed for the U.S. drainage areas because comparable water-quality, stream-network, and environmental-setting data were not readily available to develop SPARROW models for the Canadian part of the basin. The morphometric characteristics of each lake are given in Table 1. All of the Great Lakes have relatively small drainage area-to-surface area ratios, ranging from 1.5 for Lake Superior to 3.4 for Lake Ontario, and have mean depths ranging from 19 m (Lake Erie) to 147 m (Lake Superior).

**TABLE 1:** Morphometric Characteristics, Drainage-Basin Size, and Total Target Annual Phosphorus Load for Each Great Lake

Great Lake	Lake Area (km^2^)^1^	Lake Volume (km^3^)^1^	Mean Depth (m)	Drainage Area (km^2^)^1^	U.S. Drainage Area (km^2^)	Drainage Area-to-Surface Area Ratio	Target Annual Phosphorus Load^2^ (tonnes – to the entire lake)
Superior	82,100	12,100	147	124,115	43,594	1.5	3,400
Michigan	57,800	4,920	85	116,396	116,395	2.0	5,600
Huron	59,600	3,540	59	131,614	41,369	2.2	2,800
Erie	25,700	484	19	77,519	55,488	3.0	11,000
Ontario	18,960	1,640	86	63,750	35,661	3.4	7,000

1 Great Lakes Information Network (2009).

2 Target loads specified in Annex 2 of the Great Lakes Water Quality Agreement of 1972.

### SPARROW Model

SPARROW attributes is a GIS-based watershed model that uses a mass-balance approach to estimate nutrient sources, transport, and transformation (i.e., losses) in terrestrial and aquatic ecosystems of watersheds under long-term steady-state conditions (Smith *et al.*, 1997; Alexander *et al.*, 2008). A brief overview of the SPARROW model is given here and included in the Supporting Information, and a detailed description is given in Schwarz *et al.* (2006). The application of SPARROW here includes nonconservative transport and mass-balance constraints. Given a specification of nutrient sources, the model is used to estimate nutrient delivery to streams (“land-to-water” delivery) in relation to statistically significant landscape properties such as climate, soils, topography, drainage density, and artificial drainage variables. Coefficients estimated for each nutrient source represent the amount of P or N delivered to streams, and are expressed as fractions for mass variables (e.g., farm fertilizer input) or absolute quantities (kg/km^2^/year) for land-use variables (e.g., input from urban and open areas). Spatially variable land-to-water delivery factors such as climate and soil characteristics may account for differences in the rates of delivery of nutrient mass to streams. The values of the source coefficients provide an estimate of land-to-water delivery under the assumption that all the spatially variable delivery factors are uniformly distributed at average conditions throughout the area being considered. In this way, the coefficients provide a general indication of the amount of nutrient mass delivered to streams. Part of the nutrient flux is attenuated or decayed, via loss in the stream or in a reservoir or lake, as it travels down the stream network (instream/reservoir loss). This loss has been estimated in SPARROW models as a function of the average velocity in streams and areal hydraulic load in reservoirs (Alexander *et al.*, 2008).

A variety of model specifications were evaluated to determine which sources of nutrients and landscape characteristics, among those that can be reasonably represented for the entire study area and described within the construct of SPARROW, are important in controlling TP and TN transport. Therefore, some factors affecting transport may not be able to be included in the models. In some cases, SPARROW source variables serve as surrogates for other nutrient sources that are spatially correlated with the variables specified in the model. For example, developed land area (or impervious land cover) may serve as a surrogate measure of various diffuse urban sources in the model, which may include nutrient runoff from impervious surfaces, inflows from groundwater in urbanized catchments related to fertilizers and septic systems, and nitrogen deposition associated with vehicle emissions of nitrous oxides. Variables identified as statistically significant in explaining the distribution in TP and TN loads (using *p* < 0.05 as the test for significance of individual coefficients) were retained or, if sources were statistically insignificant, they were combined with other sources in a series of model runs until an acceptable specification, in terms of model fit [overall standard root mean square error (RMSE), *R*^2^ values, model-estimated coefficients, variance inflation factors, and residual plots] was obtained. Parameter coefficients associated with the sources, land-to-water delivery factors, and instream-loss and reservoir-loss terms were statistically estimated using weighted nonlinear least squares regression (NLLSR) (Schwarz *et al.*, 2006), based on calibrations with long-term mean annual loads *normalized* (described below) to 2002 [the steady-state response variables in the model at 810 (for TP) and 708 (for TN) monitoring stations throughout MRB3]. In previous studies (Qian *et al.*, 2005), Bayesian nonlinear regression has also been used to calibrate SPARROW models in an attempt to better incorporate data and model uncertainties. The monitoring sites, which are distributed throughout MRB3, represent the range in environmental characteristics of the watersheds of tributaries throughout the Great Lakes Basin. There is no reason to believe that watersheds of the Great Lakes tributaries have any unique characteristics that are not found in basins across MRB3. Using sites from throughout MRB3, rather than just those in the Great Lakes Basin, increased the number of dependent sites that could be used in the model calibration process and resulted in a better representation of the full range of conditions in the basin.

To demonstrate the robustness of the coefficients in the calibrated models, the 90% confidence interval for each coefficient was determined, assuming each NLLSR coefficient corresponds to a *t*-distribution with (*N*− *k*) degrees of freedom, where *N* is the number of monitoring sites and *k* is the number of coefficients estimated in the model. In addition, a nonparametric bootstrap procedure was used to estimate mean coefficient values from 200 individual model calibrations. In each repetition, different random weights, drawn from a uniform [0,1] distribution, were applied to each model observation (load estimates). In this procedure, the distributions of parameter estimators are inferred by assessing their empirical distributions implied by all available sample data, as opposed to the population of all possible data or subsets of the available data. Therefore, the model skill is based entirely on the model calibrations, and not evaluated based on independent data not used in the calibration. See Schwarz *et al.* (2006) for a more complete description of these procedures.

SPARROW attributes mean annual predictions of nutrient mass for stream reaches include the load, yield, volumetrically weighted concentration, and source-share contributions (percentage of the load for each source). Stream loads and yields are estimated for two spatial domains: incremental reach drainage areas and total drainage area upstream from any location. The *incremental load/yield* reflects the quantity of nutrients transported from the incremental land area to an individual reach outlet after accounting for the effects of instream attention processes (e.g., long-term storage and denitrification) associated with one-half the reach time of travel and any reservoirs in that particular reach. The *total load/yield* represents the accumulated load from all upstream areas after accounting for the effects of all instream and reservoir attenuation processes upstream of the reach outlet. In addition, the incremental and total loads or yields from any location can be described as that part of the load/yield that is ultimately transported downstream to a specific location (“delivered”), possibly a receiving water body, after accounting for the downstream removal/attenuation in streams and reservoirs. The delivered increment load/yield from a reach is calculated by multiplying the incremental loads/yields by the SPARROW estimate of the “delivery fraction” for that reach. The total delivered load at the specified location is then computed as the sum of the delivered incremental loads. Each of these metrics provides management-relevant information about the sources and fate of nutrients from local to regional spatial scales.

To reduce the effects of potential biases in the models when estimating the total loading to each Great Lake and when ranking each of their tributaries on the basis of their delivered yields, SPARROW results were used only to predict the loads from unmonitored reaches in each basin. Marginal areas are defined as areas along the Great Lakes shoreline where tributaries either do not exist or are too small to be defined on the stream coverage network and the last (downstream) reach on larger tributaries. Nonpoint-source contributions of nutrients in the marginal areas were estimated from the SPARROW simulations, but point-source contributions in those areas, most of which were the largest point sources in the study area and occurred downstream of the sites used in the calibration, were treated differently from those higher in the watershed. In the marginal areas, point-source loads derived from the USEPAs Permit Compliance System (PCS) database were directly added to model results in lieu of model predictions of instream loads from point sources in these areas. This approach can be used in the marginal areas because little if any attenuation occurs in these reaches and because there are no modeled reaches downstream that would be affected by incorporating point sources in this manner. All loading from the marginal areas is referred to as “direct” loading, and loading from areas upstream of these areas is referred to as “tributary” loading. For comparison with previous studies that did not include point sources in the marginal areas to the lakes, the contributions from tributary loading (including upstream point sources) and contributions from all nonpoint-source inputs from the marginal areas were combined and referred to as “watershed” loading; therefore, the point sources in the marginal areas and those discharging directly into the Great Lakes were not included in the “watershed” loading.

Only tributaries with drainage areas >150 km^2^ were included in the ranking process. This scale is generally consistent with the lowest resolution environmental information and the drainage-basin size of the monitoring stations used to estimate the model parameters; ∼5% of the monitored sites had drainage areas of <150 km^2^.

### Data Used to Calibrate the SPARROW Models

Four types of data are used to “build” SPARROW models: stream network information to define stream reaches and catchments; loading information for many sites within the model boundaries (dependent variables); information describing all of the sources of the nutrient or other constituent being modeled (independent variables); and information describing the environmental setting of the area being modeled that causes statistically significant variability in the land-to-water delivery of nutrients (independent variables). Each type of data is described below.

#### Stream Network Information

Water flow paths were defined by streams and reservoirs included in the enhanced stream-reach file 1 (RF1; 1:500,000 scale) with incremental reach catchments delineated with 100-m digital elevation models (Brakebill *et al.*, this issue). Within RF1, MRB3 has ∼12,000 reaches (reach catchment sizes: 5th percentile, ∼45 km^2^; 95th percentile, ∼43,600 km^2^; median, ∼480 km^2^).

#### Stream-Load Information

Two types of data are needed to estimate loads at each monitored site: daily flow data and instream water-quality concentration data. Daily flow data for each site for the period 1971-2006 were retrieved from the USGS National Water Information System (NWIS) database. Water-quality data for the period 1970-2007 were retrieved primarily from two databases: the USEPA's STOrage and RETrieval (STORET) database and USGS's NWIS database. These data were augmented with additional data from the Wisconsin Department of Natural Resources, Indiana Department of Environmental Management, Ohio River Valley Water Sanitation Commission (ORSANCO, 2009), and Heidelberg College (P. Richards, Heidelberg College, 2009, unpublished data). Only those data from agencies that collected water-quality information on a regular basis and used standardized protocols for sample collection and laboratory analysis were used in these analyses. Additional steps were taken to screen out erroneous data in the datasets: very high or very low concentrations were investigated and “fixed” when possible (such as unit errors and recording errors) or removed if warranted. See Saad *et al.* (this issue) for more information on this procedure.

The long-term mean annual nutrient loads for each monitored site were computed with the rating curve/regression procedure in the Fluxmaster computer program (Schwarz *et al.*, 2006). This procedure combines water-quality data at a monitoring station with daily flow values to provide more accurate load estimates than can be obtained by using individual water-quality measurements alone. TP and TN loads were determined with log-linear water-quality regression models that related the logarithm of constituent concentration to the logarithm of daily flow, decimal time (to compensate for trends), and season of the year (expressed using trigonometric functions of the fraction of the year). Regression models were fit to data from each potential load site (sites with >25 samples and corresponding long-term flow data, described in the Supporting Information and in Saad *et al.*, this issue). Each model was then used to estimate a long-term mean annual load *normalized* to the 2002 base year (Preston *et al.*, 2009) by first estimating daily loads for 1971-2006 detrended to 2002. Detrended daily loads to 2002 were estimated by removing the linear trend in the concentration-discharge relation by replacing the time value to 2002.5 and using detrended daily flows. The flow model used in the detrending process included seasonal terms and accounts for auto-regressive behavior in daily flows. All loads were also adjusted for log re-transformation biases. Detrended annual loads were then computed by aggregating the daily detrended loads for all years in which a complete record of daily flow was available, and averaged over all such years in the 1971-2006 period to obtain a mean annual detrended load for 2002. The 2002 base year was selected to coincide with the most recently available explanatory geospatial data. See Schwarz *et al.* (2006) for a more detailed description of the process of computing long-term mean annual normalized loads. The long-term mean annual TP and TN loads for each monitored site are included in the Supporting Information.

Normalizing mean annual nutrient loads to a base year adjusts for differences in station record lengths and sample sizes, and adjusts for temporal variability related to long-term linear trends, and interannual changes in flow. The use of normalized loads in the SPARROW models provides more robust mass-balance estimates of nutrient sources and transport processes than would an approach based solely on hydrologic records for any single year or for short multiyear periods with potentially unusual weather conditions (Schwarz *et al.*, 2006).

A wide range in watershed sizes of the sites with load information was used in model calibration: 5th percentile ∼156 km^2^ and 95th percentile ∼36,500 km^2^. The median size of the catchments for the sites used in calibrating the MRB3 models (1,950 km^2^) is much closer to the median catchment size of the model (480 km^2^) than the sites used in the previous national SPARROW models (10,500 km^2^). This should enable the MRB3 models to better represent headwater areas than the national models. The calibration sites used in the MRB3 models, however, still underrepresented the smallest catchments in the model.

#### Nutrient-Input Information

Input to SPARROW models includes data that attempt to describe or quantify all of the major sources of TP and TN. These data have been refined since earlier SPARROW models were developed (Smith *et al.*, 1997; Alexander *et al.*, 2008). The combination of more calibration sites and more accurate nutrient source data, such as those used in this study, should enable improved identification of regional nutrient sources. Because of the model's mass-balance structure, if major sources are not included in the model then the mass from these nonincluded sources (which are reflected in the measured stream loads) will either be represented by other sources that are spatially correlated with the missing sources or reflected in the model error. After evaluating a variety of model specifications, the final SPARROW model for TP was calibrated as a function of six P sources: point sources, confined manure, unconfined manure, farm fertilizers, urban and developed open lands (collectively referred to as urban areas), and a combination of forest and wetland (collectively referred to as forested areas). The final TN model was calibrated as a function of five N sources: point sources, atmospheric deposition, confined manure, farm fertilizers, and additional agricultural inputs from cultivated lands (described in more detail below). P or N inputs (as point sources, land applied, or as related to land-use characteristics) for each catchment in the stream-reach network were estimated for the 2002 base year, or as close to the base year as possible. All sources and specified land-use characteristics are described briefly in this section, but in detail in the Supporting Information. Inputs from point sources (including sewage treatment, commercial, and industrial effluent) were estimated by Maupin and Ivahnenko (this issue) from data in the USEPAs PCS database supplemented with data obtained directly from the states of Wisconsin and Minnesota (J. Schmidt, Wisconsin Department of Natural Resources, and S. Weiss, Minnesota Pollution Control Agency, 2007, written communication). TP and TN effluent loads were computed for each point-source location using methods and procedures described by McMahon *et al.* (2007) and Hoos *et al.* (2008).

The catchments were used to allocate spatial data on nutrient sources and landscape and aquatic characteristics to each reach (Wieczorek and Lamotte, 2011; unless otherwise noted all spatial data in this paper are from this source). National Atmospheric Deposition Program (NADP) wet deposition of total inorganic nitrogen estimates were used as a proxy for the *regional* contributions of total (wet plus dry) nitrogen deposition, given that NADP estimates primarily reflect regional emission patterns from stationary sources (Elliott *et al.*, 2007) and that the regional patterns of wet and dry deposition are generally correlated over large areas of the U.S. (Baumgardner *et al.*, 2002; Holland *et al.*, 2005). These data were compiled from a 1-km grid constructed from the original data using GIS processing techniques and detrended to 2002. Fertilizer and manure inputs of P and N are based on county-level estimates of fertilizer sales and animal wastes made by Ruddy *et al.* (2006). Nutrients associated with livestock wastes reflect contributions from the excreted wastes of *unconfined* animals on farms, pastures, and rangelands and from the excreted wastes of *confined* animals, including those in concentrated animal feeding operations. Confined animal wastes include recoverable manure that may be applied to nearby farmlands as well as unrecoverable manure that is lost during the collection, storage, and treatment of the waste. General land-use related inputs were based on the respective amount of area in each of the land types represented in the 2001 National Land Cover Data (NLCD) (Figure 1) (USGS, 2000). Additional agricultural nutrient inputs from cultivation are modeled as a function of the total cropland area. Because of the calibration techniques used in SPARROW, the additional estimated agricultural inputs from cultivated lands represent inputs that are spatially uncorrelated with the nutrient inputs associated with the fertilizer sales and manure data, and may reflect the effects of such processes as N fixation by legumes (e.g., soybeans and alfalfa), mineralization of soil organic matter and plant residue, nutrients previously added to the soil from other sources (e.g., manure), excreted wastes of unconfined animals, or fertilizer applications and management practices that are not accounted for by the fertilizer sales data. Nutrient inputs from urban areas were based on the combined areas for low-density, medium-density, and high-density urban and open areas. Urban areas serve as a surrogate measure of various diffuse urban sources in the model. These sources may include nutrient runoff from impervious surfaces and inflows from groundwater in urbanized catchments related to such sources as fertilizers, septic systems, and atmospheric deposition from vehicle emissions. Inputs from forested areas were based on the combined area of all forested and wetland areas.

#### Environmental-Setting Information

Following an approach similar to the one used for determining the importance of the various constituent sources, statistical methods were used to identify specific characteristics important in explaining variability in nutrient delivery to streams and losses in streams and reservoirs. Many environmental characteristics thought to be important in nutrient delivery were examined to determine statistically significant land-to-water delivery factors and instream-loss and reservoir-loss factors in the SPARROW models. Environmental-setting information, such as soil permeability, was compiled for each catchment similar to how nutrient inputs were estimated. Instream-loss and reservoir-loss factors were estimated for each stream and reservoir reach using data and methods summarized below and described in detail by Brakebill *et al.* (this issue).

Environmental-setting variables determined to significantly influence land-to-water delivery of TP include soil permeability and fraction of the stream catchment with tile drains. For the TN model, significant variables include stream drainage density, precipitation, air temperature, fraction of stream catchment underlain by tile drains, and clay content of the soil. Soil characteristics (permeability and clay content) were compiled from the USDA STATSGO database using methods described by Wolock (1997). Tile drain information was compiled from the 1997 National Resource Inventory dataset compiled by the Natural Resource Conservation Service. Mean air temperature and precipitation, representing the 30-year (1971-2000) average, were obtained from the PRISM database (PRISM Climate Group, 2009). Drainage density was calculated as total stream reach length divided by catchment area. All of the environmental-setting information used in the models is described in more detail in the Supporting Information.

Time of travel, based on stream velocity, was the factor used to describe nutrient removal (loss) in streams (categorized as small, medium, and large streams) and the inverse of the hydraulic loading was used to describe nutrient removal in reservoirs. Time of travel for each reach was estimated from average annual flow during 1975-2007 based on data from streamgaging stations throughout the U.S. (D. Wolock, USGS, 2009, written communication). Hydraulic loading for each reservoir was calculated as average flow divided by reservoir surface area based on information from the National Inventory of Dams (USACE, 2007).

## Results

### Calibration of SPARROW Models

The SPARROW TP model for MRB3 was calibrated as a function of six P sources (point sources, confined manure, unconfined manure, farm fertilizers, urban areas, and forested areas); two land-to-water delivery factors (soil permeability and fraction of the stream catchment with tile drains); nutrient removal in streams that is a function of the time of travel, which is based on stream velocity; and one factor describing removal/deposition in reservoirs (Table 2). The coefficients for all of these sources and factors were highly significant (*p* < 0.01), indicating that each of the sources, land-to-water factors, and instream/reservoir factors are important in describing the distribution in measured loads. The coefficients were also robust (see the relatively small standard errors compared to magnitude of the coefficient, 90% confidence intervals, and the mean bootstrap estimates that were within 10% of the NLLSR estimates as shown in Table 2). This model explained ∼93% of the variance in the 810 monitored loads and ∼73% of the variance in the monitored yields, and it had an overall RMSE of 0.493 (all of these statistics are based on comparisons of loads and yields in natural log units).

**TABLE 2:** Summary of SPARROW Models and Calibration Results for TP and TN

				90% Confidence Interval for the Model Coefficient				
							
Parameter	Parameter Units	Coefficient Units	Model Coefficient Value	Low	High	Standard Error of the Model Coefficient	Probability Level (*p* value)	Bootstrap Estimate of Coefficient (mean)
**Total phosphorus model**								
Sources								
Point sources (total)	kg	Fraction, dimensionless	1.068	0.835	1.302	0.142	0.0000	1.083
Manure (confined)	kg	Fraction, dimensionless	0.086	0.068	0.104	0.011	0.0000	0.085
Manure (unconfined)	kg	Fraction, dimensionless	0.032	0.015	0.049	0.010	0.0009	0.033
Fertilizers (farm)	kg	Fraction, dimensionless	0.029	0.023	0.036	0.004	0.0000	0.029
Forest, wetland, and scrubland	km^2^	kg/km^2^/year	14.7	11.8	17.5	1.723	0.0000	14.6
Urban and open areas	km^2^	kg/km^2^/year	52.3	28.6	76.0	14.4	0.0001	48.9
Land-to-water delivery								
Soil permeability (log)	cm/hr	Dimensionless	−0.652	−0.757	−0.546	0.064	0.0000	−0.636
Tiles (fraction of catchment with tiles)	Fraction	Dimensionless	−1.164	−1.477	−0.852	0.190	0.0000	−1.138
Aquatic loss								
Stream loss (m^3^/s < 1.416)	m^3^/s	m/year	0.198	0.079	0.317	0.072	0.0064	0.191
Stream loss (1.417 < m^3^/s < 2.265)	m^3^/s	m/year	0.298	0.134	0.462	0.100	0.0029	0.288
Reservoir loss	year/m	m/year	4.837	2.995	6.678	1.118	0.0000	4.371
Summary statistics								
RMSE		0.493						
Adjusted *R*^2^		0.927						
Yield *R*^2^		0.729						
Number of sites		810						
**Total nitrogen model**								
Sources								
Atmosphere (total)	kg	Fraction, dimensionless	0.513	0.447	0.579	0.040	0.0000	0.526
Point sources (total)	kg	Fraction, dimensionless	0.789	0.604	0.975	0.113	0.0000	0.792
Manure (confined)	kg	Fraction, dimensionless	0.291	0.200	0.382	0.055	0.0000	0.301
Fertilizers (farm)	kg	Fraction, dimensionless	0.131	0.068	0.194	0.038	0.0003	0.130
Additional agricultural sources	km^2^	kg/km^2^/year	625.1	136.3	1,113.8	296.7	0.0178	629.9
Land-to-water delivery								
Drainage density (log)	km/km^2^	Dimensionless	0.134	0.041	0.228	0.057	0.0184	0.146
Precipitation	mm/year	mm/year	0.002	0.001	0.002	0.000	0.0000	0.002
Air temperature	°C	°C	−0.041	−0.074	−0.009	0.020	0.0355	−0.039
Tiles (fraction of catchment with tiles)	Fraction	Dimensionless	1.133	0.922	1.343	0.127	0.0000	1.148
Clay (average areal clay content as fraction)	Fraction	Dimensionless	0.014	0.007	0.021	0.004	0.0006	0.013
Aquatic loss								
Stream loss (m^3^/s < 1.133)	m^3^/s	m/year	0.424	0.259	0.590	0.100	0.0000	0.467
Stream loss (1.134 < m^3^/s < 1.982)	m^3^/s	m/year	0.233	0.074	0.392	0.096	0.0158	0.293
Reservoir loss	year/m	m/year	6.710	4.317	9.103	1.453	0.0000	6.992
Summary statistics								
RMSE		0.408						
Adjusted *R*^2^		0.953						
Yield *R*^2^		0.849						
Number of sites		708						

Notes: SPARROW, SPAtially Referenced Regressions On Watershed attributes; TP, total P; TN, total N; RMSE, root mean square error.

In SPARROW models, the inputs from each source, except point sources, are modified by land-to-water delivery factors. The values of the coefficients associated with each source incorporate the variability resulting from these land-to-water delivery factors. Therefore, the TP model indicates that ∼100% of the point sources (coefficient = 1.068) reach the stream compared to only ∼3-9% for farm fertilizers and confined manure (coefficients = 0.029 and 0.086, respectively). A higher percentage of TP from manure from confined animals (∼9%) reaches the streams than from unconfined animals (∼3%). TP from confined animals reflects contributions from both the runoff from feedlots and the nutrients in manure that is recovered from confined operations and applied on nearby fields. On average, forested areas contribute ∼15 kg/km^2^/year (Table 2) compared to ∼52 kg/ km^2^/year from urban areas (not including input from point sources). Reckhow *et al.* (1980) conducted a literature search of nutrient export rates for selected land uses. Annual P export from forested areas ranged between 2 and 83 kg/km^2^ compared to 19 to 623 kg/km^2^ from urban areas. Therefore, the estimated yields from this study for forested and urban areas are in the range of those published in other studies.

Based on the sign of the land-to-water delivery coefficients (positive values reflect enhanced delivery with increasing values of that factor), TP yields are generally higher in areas with lower soil permeability and a lower fraction of tile drains (negative signs for these coefficients indicate inverse relations). Streams were subdivided into three sizes based on their average mean annual velocities. Instream loss is significant in small (mean annual flow less than ∼1.4 m^3^/s; <50 ft^3^/s) and medium (mean annual flow ∼1.4-2.3 m^3^/s; 50-80 ft^3^/s) streams, but not significant in large streams (mean annual flow greater than ∼2.3 m^3^/s; 80 ft^3^/s). TP removal/deposition in reservoirs is also significant.

**FIGURE 2:**
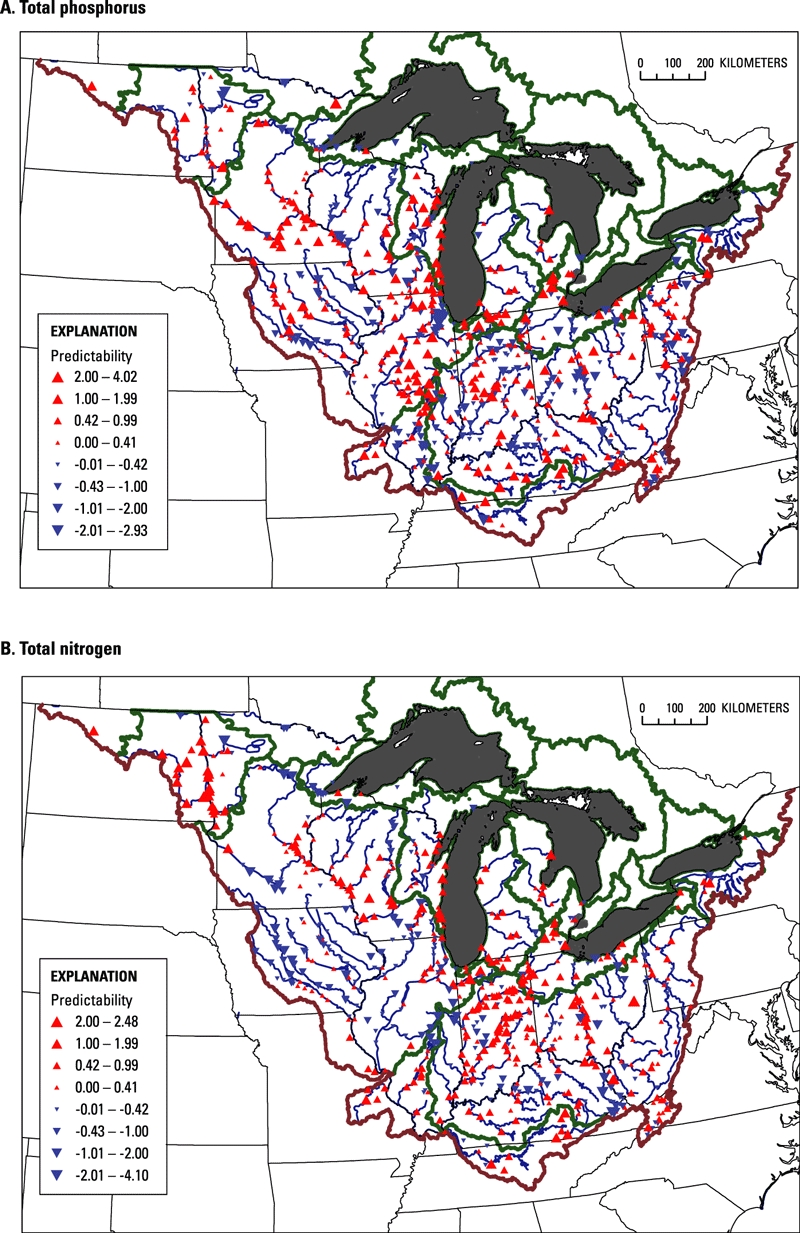
Predictability of the MRB3 SPAtially Referenced Regressions On Watershed Attributes (SPARROW) Models for (A) Total P (TP) and (B) Total N (TN). The predictability is expressed as the number of doublings (overprediction – positive values or upward pointing triangles) or foldings (underprediction – negative values or downward pointing triangles) of the measured loads at each site computed with Equation 1. All major basins are delineated.

The ability of the SPARROW TP model to simulate the observed loads is demonstrated in Figure 2A. Because positive errors can become very large but negative errors are constrained by an estimated load of zero, the prediction errors are reported on a log base 2 scale (Equation 1), such that 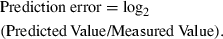
(1)

Accordingly, positive errors indicate overpredictions by the model (cases where the ratio of predicted to measured is >1), whereas negative errors indicate model underpredictions (ratio of predicted to measured is <1). For example, overpredictions of +1 and +2 indicate that the predicted value is 2× and 4× the measured value, respectively. In contrast, underpredictions of −1 and −2 indicate that the predicted value is 0.5× and 0.25× of the measured values, respectively. Most predictions were within ±1 log base 2 units of the measured values, indicating good model predictability, which corresponds to predicted loads that are within 0.5-2.0× of the measured loads. The only consistent regional biases in model predictions are in southern Illinois and southwestern Iowa, where the model underpredicted TP loads, and around the western side of Lake Michigan, central Illinois, and southern Minnesota where the model slightly overpredicted TP loads. Highest individual positive errors (overpredictions) were typically associated with small reservoirs not included in the model, such as in southeastern Michigan.

The SPARROW TN model was calibrated as a function of five N sources (atmospheric deposition, point sources, confined manure, farm fertilizers, and additional agricultural sources in cultivated areas); five land-to-water delivery factors (stream drainage density, precipitation, air temperature, fraction of stream catchment with tile drains, and the fraction of clay soils in the stream catchment); N removal in streams that is a function of the time of travel, which is based on stream velocity; and one factor describing N removal in reservoirs (Table 2). Coefficients for all sources and factors were highly significant (*p* < 0.05) indicating that each of the sources, land-to-water factors, and instream/reservoir factors are important in describing the distribution in the measured loads. The coefficients were also robust (see the relatively small standard errors compared to magnitude of the coefficient, 90% confidence intervals, and the mean bootstrap estimates that were within 10% of the calibrated NLLSR estimates except that for instream loss in moderate-sized streams that was within 26% as shown in Table 2). This model explained ∼95% of the variance in the 708 monitored loads, ∼85% of the variance in the monitored yields, and the model had an overall RMSE of 0.408 (all of these statistics are based on comparisons of loads and yields in natural log units).

The source coefficients indicate that ∼50% of the N from inorganic atmospheric deposition (discussed in more detail below), ∼80% of the point sources, and ∼10-30% of the farm fertilizers and confined manure, respectively, reach the stream. Additional agricultural inputs from cultivated agriculture were estimated to contribute ∼625 kg/km^2^/year (Table 2), which may reflect contributions from N fixation, mineralization of soil organic matter and plant residue, excreted wastes of unconfined animals, or fertilizer applications and management practices that are not reflected by the fertilizer sales data. This value only represents part of the N exported from the watershed in streams in agricultural areas and does not include export from associated fertilizers, manure from confined animals, and atmospheric inputs; therefore, the annual export rate was expected to be and was toward the low end of that found by Reckhow *et al.* (1980) for most agricultural areas: 97-7,960 kg/km^2^. N from manure from unconfined animals and urban areas were tested but found to be insignificant at *p* < 0.05. On the basis of the signs of the land-to-water delivery coefficients, TN yields are higher in areas with more tile drains, more precipitation, higher clay content soils, cooler air temperatures, and higher density of streams. Instream loss was significant in small (mean annual flow less than ∼1.1 m^3^/s; 40 ft^3^/s) and medium (mean annual flow ∼1.1-2.0 m^3^/s; 40-70 ft^3^/s) streams, but similar to that found for TP, insignificant in large streams (mean annual flow greater than ∼2.0 m^3^/s; 70 ft^3^/s). Removal/deposition in reservoirs was also significant.

The ability of the SPARROW TN model to simulate the observed loads is demonstrated in Figure 2B. Most predictions were generally within ±1 unit of the measured values, indicating good model predictability: that is, predicted loads within 0.5-2.0× of the measured loads. The only consistent regional biases in the TN model predictions were in central Iowa where the model underpredicted loads, and around Lake Michigan, northern Indiana, and along the Red River where the model overpredicted loads.

### Incremental Nutrient Yields

**FIGURE 3:**
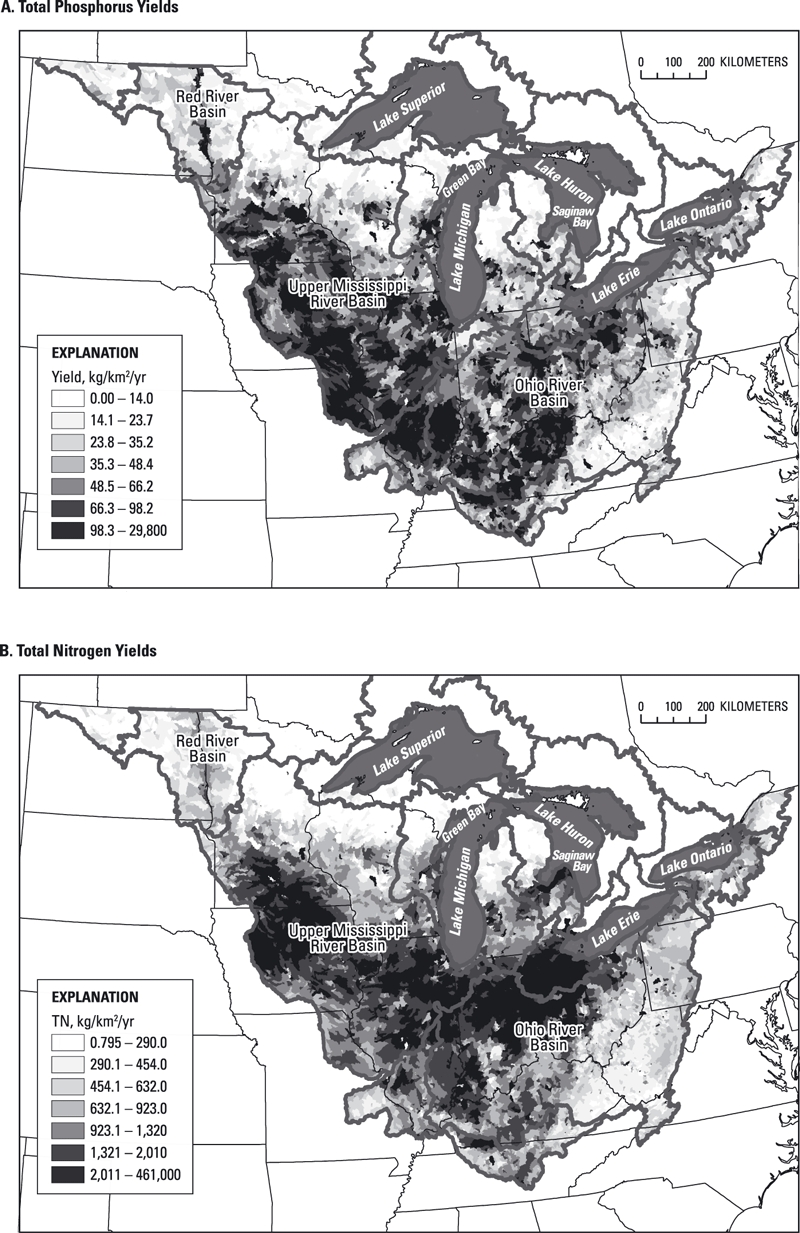
Distribution of Incremental Annual Yields of (A) Total P (TP) and (B) Total N (TN) for the SPAtially Referenced Regressions On Watershed Attributes (SPARROW) Catchments Within MRB3. All major basins are delineated.

Distributions in incremental TP and TN yields from each SPARROW model catchment (incremental load generated within a specific reach divided by its incremental upstream area, and only adjusted for attenuation associated with one-half the reach time of travel and any reservoirs in that particular reach) are shown in Figure 3. Incremental yields are mediated by the amount and type of nutrients supplied to the catchment and by environmental factors affecting their transport to streams (land-to-water delivery). The mean incremental annual TP yield was 79 kg/km^2^, whereas the mean incremental annual TN yield was 1,310 kg/km^2^ (Table 3). The very high standard deviations in the yields demonstrate that the spatial distribution of yields is highly skewed. Data in Table 3 also demonstrate the range in the importance of specific nutrient sources. For example, some catchments are dominated by one source (such as point sources or atmospheric deposition); however, in other catchments these sources may be insignificant. Highest annual yields (>1,000 kg TP/km^2^ and >25,000 kg TN/km^2^) were from relatively small catchments dominated by point sources, such as Detroit and Chicago; however, lower but still relatively high annual TP yields (110-1,000 kg/km^2^) and TN yields (2,300-25,000 kg/km^2^) were primarily from catchments in the intense agricultural areas of the Central Mississippi and Ohio River Basins. The main differences in geographic patterns in TP and TN yields can be explained primarily by differences in the distribution in the types of agricultural sources that contribute nutrients. TP yields were highest in areas with animal agriculture and TN yields were highest in areas with crop-oriented agriculture; highest TP yields were generally a little north (southwestern Wisconsin) and south (southern Illinois) of the areas with the highest TN yields. Within the Great Lakes watershed, relatively high nutrient yields were found primarily in eastern Wisconsin, central Michigan, and northern Ohio. The sum of incremental TP and TN loads and yields, with confidence intervals, from all of the 8-digit Hydrologic Unit Code (HUC8) (Seaber *et al.*, 1987) watersheds throughout MRB3 are summarized in the Supporting Information (note: these incremental loads/yields are not adjusted for prediction biases).

**TABLE 3:** Summary Statistics of Estimated Annual Yields and Source Shares From Incremental Catchments in MRB3

	Total Phosphorus							Total Nitrogen						
		
			Percentiles							Percentiles				
						
Variable	Mean	SD	10th	25th	Med	75th	90th	Mean	SD	10th	25th	Med	75th	90th
Yield (kg/km^2^)														
Incremental yield^1^	79.0	502.0	11.9	21.2	41.9	72.8	114.0	1,310	6,100	244	424	768	1,440	2,360
Delivered yield^2^	52.2	83.0	10.3	18.7	39.5	72.3	103.0	1,080	1,430	176	378	735	1,420	2,370
Source shares (%)^3^														
Atmospheric deposition	-	-	-	-	-	-	-	50.6	30.8	17.0	22.2	42.0	81.2	97.9
Point sources	7.7	19.2	0.0	0.0	0.0	3.2	26.1	5.0	15.7	0.0	0.0	0.0	0.9	11.2
Manure (confined)	18.1	18.5	0.0	2.2	12.3	29.0	47.6	10.7	10.9	0.1	1.8	7.4	16.2	27.4
Manure (unconfined)	9.0	9.9	0.5	2.2	5.7	11.8	23.5	-	-	-	-	-	-	-
Farm fertilizer	22.9	21.8	0.2	3.5	17.2	36.2	55.9	21.6	16.6	0.4	4.9	20.6	37.0	44.2
Additional agricultural sources	-	-	-	-	-	-	-	12.2	10.6	0.0	1.6	10.4	21.5	26.2
Forested land	27.7	28.8	1.0	3.8	14.9	47.4	76.3	-	-	-	-	-	-	-
Urban and open areas	14.5	17.3	2.7	4.6	8.7	16.9	31.5	-	-	-	-	-	-	-

Notes: Med, median (50th percentile); TP, total P; TN, total N; -, not in the model.

1 The amount of TN or TP generated within a given incremental catchment that makes it to the catchment outlet, and incorporates the effects of instream attenuation processes associated with one-half the reach time of travel and any reservoirs in that particular reach.

2 The amount of TN or TP generated within a given incremental catchment that is ultimately delivered to the end of a basin or Great Lake.

3 The amount (share) of TN or TP, in percent, generated within a given incremental catchment that can be attributed to the sources in the model.

### Nutrient Delivery to the Great Lakes

**FIGURE 4:**
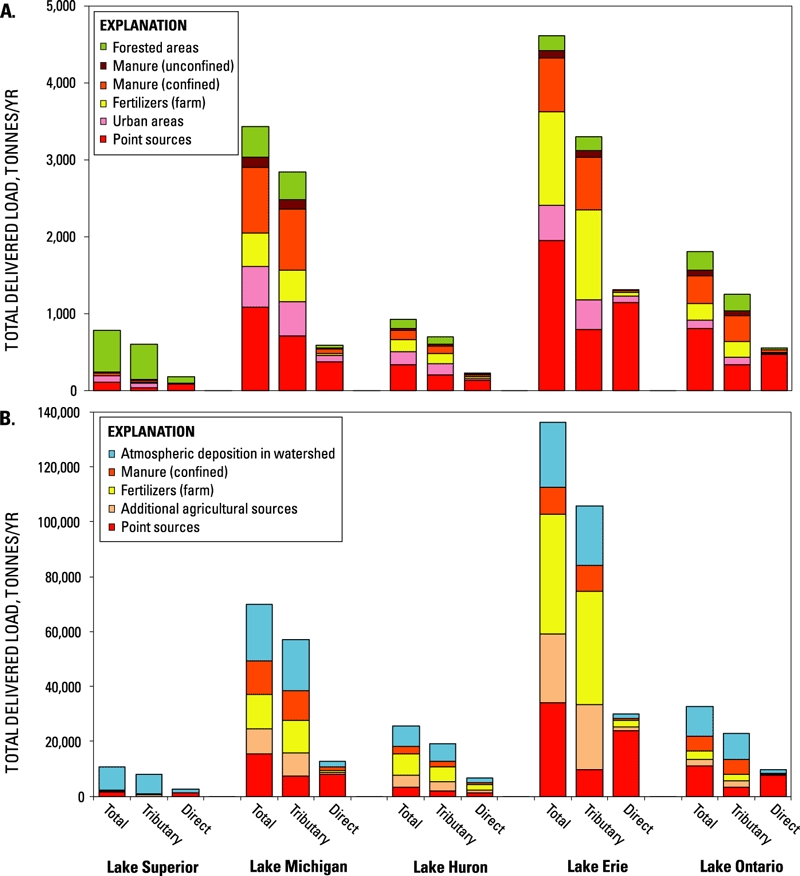
Total Annual Delivered Load (subdivided into tributary loading and direct loading from marginal areas around the lake) to Each Great Lake for (A) Total P (TP) and (B) Total N (TN). Loads are subdivided by source. (Note: input from direct atmospheric deposition is not included; all percentages by individual source are given in the Supporting Information.)

To reduce the effects of biases in the predictions from the SPARROW models for individual streams with measured loads (Figure 2) when estimating the nutrient load delivered to each Great Lake, model results were used only to predict the loads from unmonitored reaches in each of the basins and were not used to estimate point-source contributions in the marginal areas around the lakes where there would be little if any attenuation. Annual TP loading (U.S. contributions) ranged from 782 MT (metric tonnes) into Lake Superior to 4,610 MT into Lake Erie (Table 4; Figure 4A). Total annual loading to Lake Michigan, the only lake whose entire watershed is within the U.S., was 3,430 MT. Direct loading from the marginal areas around each lake ranged from ∼17% (Lake Michigan) to ∼30% (Lake Ontario) of this total. Annual TN loading (U.S. contributions) ranged from 10,900 MT into Lake Superior to 136,000 MT into Lake Erie (Table 4 and Figure 4B). The TN loading to Lake Michigan was 70,000 MT. Direct loading from the marginal areas around each lake represented ∼18-30% of this total. These loads do not incorporate atmospheric deposition directly on the surface of the lakes.

**TABLE 4:** Estimated Annual Loading and Yields of TP and TN Into Each Great Lake, Normalized to 2002

		Total Phosphorus						Total Nitrogen		
			
Great Lake	U.S. Drainage Area (km^2^)	2002 Total U.S. Load (tonnes)^1^	2002 Total U.S. Yield (kg/km^2^)^1^	Delivery Ratio^2^	2002 Total U.S. Load From Direct Point Sources (tonnes)	2002 U.S. “Watershed” Loading (tonnes)	1983-1985 U.S. “Watershed” Loading (tonnes)^3^	2002 Total U.S. Load (tonnes)^1^	2002 Total U.S. Yield (kg/km^2^)^1^	Delivered Load to Total Nondecayed Load Ratio
Superior	43,594	782	17.9	0.92	75	707	1,500	10,900	250	0.91
Michigan	116,395	3,430	29.5	0.86	374	3,060	3,230	70,000	601	0.84
Huron	41,369	927	22.4	0.91	126	801	1,550	25,900	625	0.89
Erie	55,488	4,610	83.1	0.96	1,150	3,470	5,670	136,000	2,450	0.96
Ontario	35,661	1,800	50.6	0.89	464	1,340	1,270	32,800	919	0.86

Notes: TP, total P; TN, total N.

1 Loads and yields from the U.S. part of each lake's watershed, and do not include direct atmospheric deposition.

2 The delivery ratio is computed as the total delivered load divided by the total nondecayed load.

3 Loads from Rathke and McRae (1989).

Nutrient yields from the watersheds of each lake were computed to adjust for differences in basin size by dividing the total loading (including all direct loading) by the drainage area contributing the load (Table 4). Annual TP yields ranged from 17.9 kg/km^2^ into Lake Superior to 83.1 kg/km^2^ into Lake Erie. Annual TN yields ranged from 250 kg/km^2^ into Lake Superior to 2,450 kg/km^2^ into Lake Erie. For both TP and TN, yields from the Lake Erie Basin were much higher than from the other basins. The Erie Basin has the largest percentage of agricultural areas (Figure 1) and the largest contribution from point sources (Figure 4). Yields were second highest from the Ontario Basin, followed by those from Michigan/Huron, and Superior Basins. The Superior Basin had the smallest amount of agriculture and the smallest contribution from point sources.

The total delivered load/yield to each Great Lake is mediated by the amount and type of nutrients supplied to all of the upstream catchments, the environmental factors affecting their transport to streams, and the losses that occur in the transport downstream. Therefore, the spatial arrangement of the nutrient sources (i.e., distance from lake and presence of intervening reservoirs) can affect the final amount delivered to a waterbody. The ratio of the total delivered load to the total nondecayed load (referred to as delivery ratio) was computed for each Great Lake (all of the attenuation occurring in streams and reservoirs were removed from the nondecayed loads in Table 4). The delivery ratios vary from 0.96 for Lake Erie to ∼0.85 for Lake Michigan, indicating between 4 and 15% of the nutrients entering the streams are lost in transport. This demonstrates that more of the nutrients entering Lake Michigan streams are lost in transport than in Lake Erie streams. The higher losses in Lake Michigan streams may be caused by many of its tributaries passing through reservoirs, such as the Fox River, Wisconsin that flows through Lake Winnebago prior to reaching Green Bay, and many of its point sources located more upstream in the watershed compared to Lake Erie with fewer reservoirs and most point sources located near the lake. Losses in streams entering the other lakes are between these extremes.

### Sources of Nutrients

Sources of P to the Great Lakes include inputs from point sources (sewage, commercial, and industrial sources), confined manure, unconfined manure, farm fertilizers, undefined inputs from urban and developed open lands (urban areas), and undefined inputs from a combination of forest and wetland (forested areas). Each of these sources was highly significant in the SPARROW model. The total inputs from these sources are modified by land-to-water delivery factors, and instream and reservoir losses. The percentage of the P input to each Great Lake from each of these sources is shown in Figure 4A (actual values are in the Supporting Information). This model did not specifically identify atmospheric contributions or additional agricultural sources of P; therefore, if such sources exist, they would be incorrectly attributed to the other defined sources.

Inputs from urban and agricultural areas, on a percentage basis, were relatively similar for each Great Lake, except Lake Superior. P from point sources represented ∼30-44% of the total input to each lake, except for Lake Superior for which it represented only 14% of the total. The largest source to Lake Superior was from forested areas. Most of the point sources were either input directly to the lakes or near their shorelines (direct loading): for Lake Superior 71% of the total input from point sources occurred in the marginal areas, compared to ∼58% for Lakes Erie and Ontario, and 35-38% for Lakes Michigan and Huron (Figure 4). For Lake Erie, >40% of all of its point sources was directly input into the Detroit River. Point sources in combination with urban areas contributed ∼47-54% of the input to each lake except Lake Superior (24%). Inputs from agricultural sources represented ∼33-44% of the total input into each lake except into Lake Superior (7%). The breakdown between inputs from manure and farm fertilizers was quite variable. Inputs from farm fertilizers were more important for Lake Erie (26% of the total input), whereas inputs from manure (mostly from confined animals) were more important for Lakes Michigan and Ontario (∼24-29% of the total input). Inputs from unconfined animals represented only 2-4% of the TP input to each lake.

Sources of N to the Great Lakes include inputs from point sources, confined manure, farm fertilizers, additional agricultural sources, and atmospheric deposition (Figure 4B; and in the Supporting Information). This model did not specifically identify contributions from unconfined animal operations, or from forested or urban areas; therefore, if such sources exist, they would be incorrectly attributed to the other defined sources. In general, agricultural inputs were the largest source of N to each lake, except for Lake Superior where atmospheric deposition was the major source. Inputs from agricultural sources (manure, fertilizer, and additional agricultural inputs from such sources as fixation, and mineralization of organic matter and plant residue) represented ∼58% of the input for Lakes Huron and Erie, 48% for Lake Michigan, ∼33% for Lake Ontario, and ∼7% for Lake Superior. The breakdown between inputs from manure, farm fertilizers, and additional agricultural sources was quite variable. Inputs from fertilizers were generally the largest agricultural source, representing ∼30% of the TN input to Lakes Erie and Huron, but only representing 18% of the input to Lake Michigan, 9% into Lake Ontario, and 1% into Lake Superior. Manure and the additional agricultural sources each contributed ∼7-18% of the total inputs, except for Lake Superior where it represented only 1-2%. Inputs from manure were more important than the additional agricultural sources in Lakes Michigan and Ontario, whereas inputs from additional agricultural sources were more important than manure in Lakes Huron and Erie. Point sources represented ∼13-34% of the total input to each Great Lake. Much of the input from point sources was either input directly to a lake or very near their shorelines (52-77% of the total point sources for all lakes except for Lake Huron where only 37% of the total point sources were input near the shorelines).

### Relative Nutrient Input by Tributary

The tributaries to each lake with drainage areas >150 km^2^ were ranked based on their respective total delivered loads and yields. All loads (and confidence intervals) and yields, and their respective rankings based on TP and TN loads and yields, for all tributaries to each Great Lake are listed in the Supporting Information. Because of the approach used to estimate total loading using the measured loads where available, confidence intervals on the rankings of tributaries, such as shown in Robertson *et al.* (2009a), could not be determined.

For all lakes, the highest ranked tributaries, based on TP and TN loadings, were directly related to watershed size. For Lake Superior, the largest contributors were the St. Louis and Ontonagon Rivers. For Lake Michigan, the largest contributors were the Fox, Grand, and St. Joseph Rivers (Fox River had highest TP loading and the Grand River had highest TN loading). For Lake Huron, the largest contributor was the Saginaw River. For Lake Erie, the largest contributor was the Maumee River. For Ontario, the largest contributors were the Oswego and Genesee Rivers.

**FIGURE 5:**
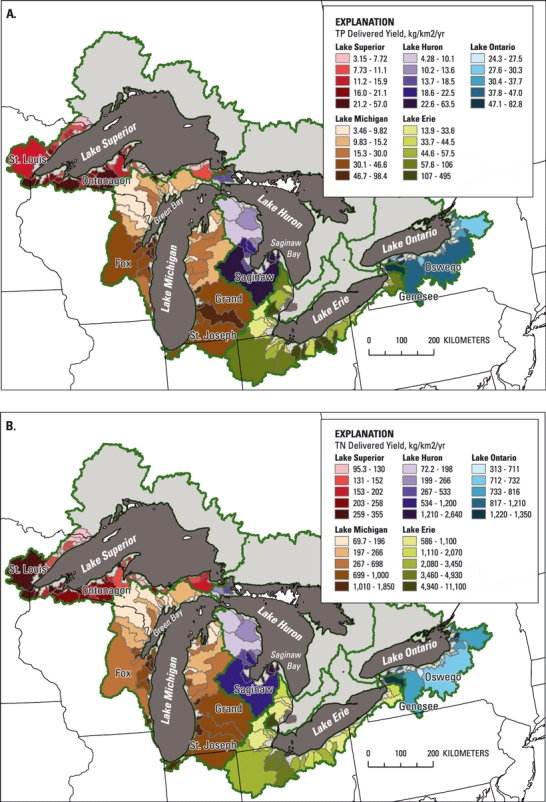
Distributions of Total Annual Delivered Yield From Each Tributary >150 km^2^ to Each Great Lake for (A) Total P (TP) and (B) Total N (TN). Yields from tributaries to each Great Lake are scaled independently. The entire drainage of each lake is delineated; however, only the tributaries in the U.S. are included in the analyses.

After adjusting for the watershed size of each tributary, it is possible to determine where in each basin the largest relative amounts of nutrients originate and where targeted actions may have the largest impact (Robertson *et al.*, 2009a). Annual TP and TN yields ranged from 3 kg TP/km^2^ and 95 kg TN/km^2^, respectively, in the pristine areas northwest of Lake Superior to 495 kg TP/km^2^ and 11,100 kg TN/km^2^, respectively, near Detroit (Figure 5). For Lake Superior, highest TP yields are along the western side of the south shore of the lake and highest TN yields are along the southwest tip of the lake. For Lake Michigan, highest TP and TN yields are mostly near the central part of the lake; however, high TN yields are also from the south shore. For Lake Huron, highest TP and TN yields are from the south shore of Saginaw Bay. For Lake Erie, in addition to very high yields near Detroit, high TP and TN yields are from the southwest shore of the lake; however, highest TN yields are from areas slightly west of the highest TP yields. For Lake Ontario, highest TP yields are from the southwest side of the lake. In contrast, highest TN yields occur along the southeast and southwest sides of the lake. Only in a few cases are the highest loads and yields to a lake the same, for example the Saginaw River flowing into Lake Huron for TP and the St. Louis flowing into Lake Superior for TN.

## Discussion

### Comparisons to Nutrient Loading and Ranking in Previous Studies

The entire landscape-derived nutrient loading could be estimated only for Lake Michigan because the other lakes have large areas draining from Canada, and comparable water-quality, stream-network, and environmental-setting data were not readily available to develop SPARROW models for these areas. Annual P loadings to Lake Michigan were estimated from 1974 to 1991 by the IJC and then from 1994 and 1995 as part of the Lake Michigan Mass Balance Study (Madenjian *et al.*, 2002; Rossmann, 2006). Total annual loadings to Lake Michigan were estimated to be ∼6,000 MT from 1974 to 1979 and then declined to ∼3,000-5,000 MT from 1980 to 1991. In 1994 and 1995, the total annual load was estimated to be ∼3,330 and 2,940 MT, respectively. For environmental conditions similar to 2002, the total annual P load to Lake Michigan is estimated to be ∼3,690 MT [3,430 MT found in this study plus 260 MT estimated by Rossmann (2006) for atmospheric deposition on the surface of the lake]. Therefore, the TP loading based on the results from this study for circa 2002 is similar to that estimated as part of the Lake Michigan Mass Balance Study and suggests that long-term average loading has remained relatively unchanged since ∼1980. Annual loading to the lake remains well below the GLWQA target P load of 5,600 MT (Table 1).

Annual P loadings were estimated from 1983 to 1985 for each Great Lake as a whole and from the Canadian and U.S. areas independently by Rathke and McRae (1989). Their loading estimates from the U.S. area did not include point-source input from the marginal areas around the lakes; therefore, “watershed” loadings (total load minus point sources in the marginal areas) from the present study are compared with those from 1983 to 1985 (Table 4). Watershed loadings to Lakes Michigan and Ontario are similar in both studies, suggesting that the total loads to these lakes have not changed much since the 1980s except possibly from direct point sources in marginal areas around the lakes. Watershed P loadings to Lakes Superior, Huron, and Erie estimated in this study, however, are lower than those estimated in 1983-1985, suggesting that the loadings may have decreased. In estimating the loads from each monitored river with Fluxmaster, trends in constituent concentrations and loads were also computed. In the tributaries that contribute the largest loads to each of these three lakes, there was a decreasing trend in TP loads from 1970 to 2007 (including the Saginaw River, which contributes ∼60% of the total load to Lake Huron; the Maumee River, which contributes ∼50% of the total load to Lake Erie; and St. Louis and Ontonagon Rivers, which contribute ∼30% of the total load to Lake Superior) (Figure 5). All of these trends were statistically significant at *p* < 0.05 except that in the St. Louis River (*p* = 0.17). No consistent trends were found in the major contributors to Lakes Michigan and Ontario.

Only a few studies have attempted to rank the tributaries to each Great Lake based on their individual loads (Sonzogni *et al.*, 1978; Rathke and McRae, 1989; Robertson, 1997). The findings from all of these studies agree with those of this study for TP (Supporting Information Table S6): for Lake Superior the highest loads come from the St. Louis and Ontonagon Rivers (in decreasing order); for Lake Michigan the highest loads come from the Fox, Grand, and St. Joseph Rivers; for Lake Huron the highest load comes from the Saginaw River; for Lake Erie the highest load comes from the Maumee River; and for Lake Ontario the highest loads come from the Oswego and Genesee Rivers. The earlier studies were not able to rank all of the tributaries based on their yields because yields computed for the monitored tributaries were used for the unmonitored tributaries, which demonstrates a benefit of using a model such as SPARROW, which can estimate the loadings and variable yields from all large and small tributaries over a large area rather than just those that were monitored.

In addition to ranking tributaries to each Great Lake, SPARROW results can be used to rank subbasins within the larger tributary watersheds and describe relative differences in the importance of nutrient sources among subbasins. This information could be useful in prioritizing management actions in TMDL or TMDL-like efforts, such as in the Fox River Basin draining into Lake Michigan (WDNR, 2009) or the Saginaw River Basin draining into Lake Huron (MDNR, 1991), and useful in guiding the types of management actions needed to reduce the loads from specific sources.

### Factors Affecting the Sources and Delivery of Nutrients

The statistical methods and digital network of stream and reservoir reaches used in SPARROW enable results of the model simulations to be used to: (1) establish links between water quality and constituent sources, (2) track the transport of constituents to streams and downstream receiving waters, and (3) assess the natural processes that attenuate constituents as they are transported from land and downstream (Preston *et al.*, 2009). The coefficients associated with the various nutrient sources describe the fraction or amount (kg/km^2^) of each source reaching the stream (Table 2). However, individual sources identified in SPARROW (as in any model) may act as surrogates for various other sources that are spatially correlated with the loads. As a result of these interactions, some ambiguity can be expected in the source-share descriptions that may make the interpretation of the coefficients associated with a few of the source terms difficult. An example of this difficulty can be seen with point sources. The coefficient associated with point sources is expected to be near 1.0 because nutrients are usually directly input into a stream. There are a few reasons, however, why this coefficient may differ from 1.0. The estimation of the inputs from some facilities may be biased low because some inputs may be unaccounted for, such as those from combined sewer overflow (thus leading to a coefficient value >1.0). In this study, care was taken to identify point sources but some may still be unidentified. Point-source loading of P has been well measured in the Midwest, which is probably the reason why in the SPARROW TP model, the coefficient for point sources was very close to 1.0. It appears that the N inputs from point sources may have been overestimated in this study, which may have resulted in the coefficient being <1.0. This is not surprising given that N inputs from many point sources are not measured and were estimated based on the theoretical concentrations for specific facility types (Maupin and Ivahnenko, this issue).

For each of the sources, the best available data were used when the actual input data were not available. For example, for atmospheric N deposition, TN deposition rates were not available and were therefore estimated from wet deposition (ammonia plus nitrate) estimates that are spatially correlated with total deposition, which resulted in total inputs from atmospheric deposition being underestimated. Therefore, although the coefficient (0.513) suggests that ∼50% of the input from atmospheric deposition reaches the stream, the actual percentage of N delivered from atmospheric deposition is likely to be less.

To try to minimize specific sources from being mistakenly apportioned in SPARROW results, special efforts were made to include all sources of P and N in the models. However, in the TP model, we did not include atmospheric inputs, or application of sludge or biosolids from wastewater treatment plants, and we were unable to identify additional inputs from agricultural sources; therefore, their contributions would be attributed to other sources in the model. For atmospheric P input, the input was probably attributed to forest and urban area sources and in the other source terms in agricultural areas. Atmospheric P input has been measured in only a few local studies. Robertson *et al.* (2009b) estimated annual atmospheric deposition of P to be ∼14 kg/km^2^, which is small compared to the inputs of fertilizers and manure, but it is similar to that estimated to be exported from forested areas (Table 2). Therefore, atmospheric P input to forested areas may be in equilibrium with what is leaving these areas, which suggests the main long-term source to forested areas is atmospheric deposition. Part of the urban source term (∼25%) may be from atmospheric P deposition. Inputs of P from sludge or biosolids from wastewater treatment plants are expected to be included in the agricultural and urban sources because that is where these by-products are deposited.

In the TN model, we were unable to identify inputs from unconfined animal manure, inputs from forested and urban areas, and inputs from sludge or biosolids from wastewater treatment plants; therefore, their contributions would be attributed to other sources in the model. N in unconfined animal manure was found to be insignificant. This may actually occur because most of the N in manure deposited by unconfined animals may be volatilized prior to runoff from the fields (Meisinger and Jokela, 2000; Diebel and Vander Zanden, 2009) and may be redeposited elsewhere as part of atmospheric deposition.

Within SPARROW models, the sources of nutrients are modified by the land-to-water delivery factors (Table 2), and collectively they account for the spatial variability in the amount of nutrients that reach streams. For example, the coefficients associated with the fraction of the catchment containing tile drains (−1.164 for TP and 1.133 for TN) indicate that tile drains increase the delivery of N to streams but decrease the delivery of P. Tile drainage has been shown to increase the delivery of soluble nutrients (such as nitrate) to surface waters because of the rapid conveyance of drainage water that has leached nutrients from the upper soil profile (David *et al.*, 1997). Tile drainage, however, tends to reduce surface runoff from an area and thus decreases delivery of sediment and sediment-bound nutrients such as P to surface-water bodies (Zucker and Brown, 1998).

Natural processes also attenuate the flux of nutrients being transported downstream. Results from this study indicate that there is a reduction in P and N loads in small-to-medium sized streams (mean annual flow less than ∼2 m^3^/s); however, instream loss in large streams (mean annual flow greater than ∼2 m^3^/s; ∼70 ft^3^/s) was found to be insignificant. Alexander *et al.* (2009) also found much more attenuation of nutrients in small streams than in larger rivers, especially for N. In addition to instream loss, nutrients may be deposited in lakes or reservoirs. Many of the tributaries to the Great Lakes pass through lakes or through reservoirs prior to entering a Great Lake, such as the Fox River, which flows through Lake Winnebago prior to reaching Green Bay on Lake Michigan. Results from the SPARROW models indicate significant reduction in P and N loads as the tributaries pass through impoundments. Collectively the losses of nutrients in streams and reservoirs are reflected in the delivery ratio (ratio of total delivered load to total nondecayed load as shown in Table 4).

## Summary and Conclusions

As a result of nutrient loading, eutrophication problems have developed in many areas of the Great Lakes. In an attempt to find the sources of these problems and prioritize efforts for remediation, detailed load estimates are needed. In recent years, however, the number of monitored tributaries has shrunk. In an attempt to address these issues in the absence of more complete monitoring data, SPARROW models were developed for P and N for a 2002 base year for the Upper Midwest region of the U.S. Results from the SPARROW models were used to accomplish four objectives:

Lakewide TP and TN loadings were determined. Total watershed loadings to Lakes Michigan and Ontario were found to be similar to those estimated in the 1980s, whereas loadings to Lakes Superior, Huron, and Erie were lower than those estimated in the 1980s, suggesting that the nutrient loadings to these lakes may have decreased from the mid-1980s to 2002. Highest loading rates (yields) were into Lake Erie and lowest rates were into Lake Superior. The spatial arrangement of the nutrient sources (i.e., distance from lake and presence of reservoirs) also affected the amount delivered to each Great Lake. The ratio of the total delivered load to the total nondecayed load varied from 0.96 for Lake Erie (with fewer intervening reservoirs and point sources nearer the lake) to ∼0.85 for Lake Michigan (with more reservoirs and point sources further from the lake).

Total P and TN loadings were determined from each tributary and the tributaries to each lake were ranked based on their relative loadings and yields. Highest loads were from rivers with the largest drainage basins; however, the highest yields were found in areas having intense agriculture and large point sources of nutrients. This study evaluated all tributaries to the lakes that drained areas >150 km^2^, whereas previous modeling approaches for the Great Lakes did not enable all of the tributaries to be evaluated and compared because they relied only on yields from monitored tributaries. Results from the SPARROW models can also be used to rank subbasins in large tributaries.

The relative importance of different P and N sources was determined. Input from agricultural operations was a significant source of nutrients, contributing ∼33-44% of the P and ∼33-58% of the N, except from areas around Lake Superior with small areas of agricultural land. Point sources of nutrients were still significant to total loads, contributing ∼14-44% of the P and 13-34% of the N.

The environmental factors that significantly affect the delivery of P and N from the land to the streams in the Upper Midwest were determined. Tile drainage increased the delivery of N but decreased the delivery of P. Instream loss was important for P and N in small-to-medium sized streams, but insignificant in larger rivers. In addition to instream loss, P and N deposition in lakes and reservoirs on tributaries to the Great Lakes was important in reducing nutrient delivery to the lakes.

## SUPPORTING INFORMATION

**Material S1.** A description of the SPAtially Referenced Regression On Watershed attributes (SPARROW) model.

**Material S2.** Data Requirements. Specifications used in the Major River Basin number 3 (MRB3) SPARROW models (Table S1).

**Material S3.** Summary of measured (with Fluxmaster) and estimated (with SPARROW) loads for each monitoring site used in the calibration of the SPARROW models (Table S2).

**Material S4.** Description of the SPARROW input data.

**Material S5.** Figures demonstrating SPARROW model calibration for total phosphorus (TP) and total nitrogen (TN).

**Material S6.** Output from the MRB3 TP and TN SPARROW models for:

- Loads and yields for each Great Lake and nearby large river basin (Tables S3 and S4);
- Loads and yields for each HUC8 (Tables S5_TP and S5_TN);
- Loads and yields for each tributary > 150 km^2^ to each Great Lake (Tables S6_TP and S6_TN). Tributaries to each Great Lake are ranked by relative loads and yields.

**Material S7.** Comparison of the yields and relative sources of P and N among Great Lakes watersheds with those from the Upper Mississippi River, Ohio River, and Red River watersheds.

**Material S8.** References for Supplemental Material.

Please note: Neither AWRA nor Wiley-Blackwell is responsible for the content or functionality of any supporting materials supplied by the authors. Any queries (other than missing material) should be directed to the corresponding author for the article.
