# Supplementary material for: Nutrient Inputs to the Laurentian Great Lakes by Source and Watershed Estimated Using SPARROW Watershed Models
Source: J Am Water Resour Assoc. 2011 Oct;47(5):1011–33. doi: 10.1111/j.1752-1688.2011.00574.x (PMC3307632; doi:10.1111/j.1752-1688.2011.00574.x)
Supplement: Supplementary file 1 [file jawr0047-1011-SD1.pdf]

Nutrient Inputs to the Laurentian Great Lakes
by Source and Watershed Estimated Using SPARROW Watershed Models

By
Dale M. Robertson and David A. Saad

Supporting Information:

Robertson and Saad, 2011, Nutrient Inputs to the Laurentian Great Lakes
By Source and River Basin Estimated Using SPARROW Watershed Models, *This Issue.*

There are eight parts to this Supporting Information

S1. A description of the SPAtially Referenced Regression On Watershed attributes (SPARROW) model.

S2. Data Requirements/Specifications used in the Major River Basin number 3 (MRB3) SPARROW models (Table S1).

S3. Summary of measured (with Fluxmaster) and estimated (with SPARROW) loads for each monitoring site used in the calibration of the SPARROW models, with a summary of the data used to estimate the measured loads (Table S2).

S4. Description of the SPARROW input data.

S5. Figures demonstrating SPARROW model calibration for total phosphorus (TP) and total nitrogen (TN).

S6. Output from the MRB3 TP and TN SPARROW models for:

- Loads and yields each Great Lake and nearby large river basin (Tables S3 and S4);
- Loads and yield for and each HUC8 (Tables S5_TP and S5_TN);
- Loads and yield for each tributary > 150 km^2 to each Great Lake (Tables S6_TP and S6_TN). Tributaries to each Great Lake are ranked by relative loads and yields.

S7. Comparison of the yields and relative sources of P and N among Great Lakes watersheds with those from the Upper Mississippi River, Ohio River, and Red River watersheds.

S8. References for Supporting Information.

S1. Description of the SPARROW model

SPARROW is a GIS-based watershed model that uses a mass-balance approach to estimate nutrient sources, transport, and transformation in terrestrial and aquatic ecosystems of watersheds under long-term steady-state conditions (Smith *et al.*, 1997; Alexander *et al.*, 2008). SPARROW includes non-conservative transport, mass-balance constraints, and water flowpaths defined by topography, streams, and reservoirs, based on a stream-reach network with delineated reach catchments. The model-estimated flux leaving each reach (*i)* in SPARROW, F_i^*, is given by

$$F_i^* = \left(\sum_{j \in J(i)} F_j' \right) \delta_i T\left(\mathbf{Z}_i^S, \mathbf{Z}_i^R; \boldsymbol{\theta}_S, \boldsymbol{\theta}_R \right) + \left(\sum_{n=1}^{N_S} S_{n,i} \alpha_n D_n \left(\mathbf{Z}_i^D; \boldsymbol{\theta}_D \right) \right) T'\left(\mathbf{Z}_i^S, \mathbf{Z}_i^R; \boldsymbol{\theta}_S, \boldsymbol{\theta}_R \right). \qquad \text{(S1)}$$

The first summation term represents the flux (F_j^*) from all upstream confluent reaches $J(i)$ that are delivered downstream to reach *i*. δ_i is the fraction of upstream flux delivered to reach *i*; δ_i generally equals 1 unless the upstream end of reach *i* is the location of a diversion. $T(\cdot)$ is the stream transport function representing attenuation processes acting on the flux as it travels along the reach pathway (instream loss). This function defines the fraction of the flux entering reach *i* at the upstream node that is delivered to the reach's downstream node. The factor is a function of measured stream and reservoir characteristics, denoted by the vectors $\mathbf{Z}^S$ and $\mathbf{Z}^R$, with corresponding coefficient vectors $\boldsymbol{\theta}_S$ and $\boldsymbol{\theta}_R$.

The second summation term represents the incremental flux (that introduced to the stream network in reach *i*). This term is composed of the flux originating from specific sources, indexed by $n = 1, \ldots, N_S$. Associated with each source is a source variable, denoted S_n. Depending on the nature of the source, this variable could represent the mass of the source available for transport to streams, or it could be the area of a particular land use. The variable α_n is a source-specific

coefficient that converts source variable units to flux units. The function $D_n(\cdot)$ represents the land-to-water delivery factor. For sources associated with the landscape, this function along with the source-specific coefficient determines the amount of a constituent delivered to streams. The land-to-water delivery factor is a source-specific function of a vector of delivery variables, denoted by $\mathbf{Z}_i^D$, and an associated vector of coefficients $\boldsymbol{\theta}_D$. The last term in the equation, function $T'(\cdot)$, represents the fraction of flux originating in and delivered to downstream end of reach *i*. This term is similar in form to the stream delivery factor defined in the first summation term but is used to transport flux only from the midpoint of the reach to the outlet.

Parameter coefficients associated with the sources, land-to-water delivery factors, and instream- and reservoir-loss terms were statistically estimated using weighted nonlinear least squares regression, based on calibrations with long-term mean annual normalized loads at each monitored station throughout the study area. A more indepth description of the SPARROW model and its calibration is given in Schwarz *et al.* (2006).

S2. Data requirements/specifications used in the MRB3 SPARROW models

Table S1. Data requirements and specifications for data used in the MRB3 SPARROW models.

Supplemental Material to Robertson and Saad, 2011, Journal of the American Water Resources Association, Nutrient Inputs to the Laurentian Great Lakes By Source and River Basin Estimated Using SPARROW Watershed Models.

Category	Requirements/Specifications
**Water-quality sites**	
Time period for data	10/1/1970 - 9/30/2007
Time period covered by water-quality data	> 2 years
Data near 2002 base year	Data within 2 years of 2002 if < 5 years of data; data within 7 years of 2002 if > 5 years of data
Total number of samples	>25 samples
Total number of uncensored sample values	>25 samples
Total samples in each of four seasons	>1 sample for each season (winter: Dec.-Feb.; spring: Mar.-May; summer: June-Aug.; fall: Sept-Nov.)
Location of site	On enhanced RF1 stream coverage
**Coinciding stream gage**	
Time period for data	10/1/1970 - 9/30/2006
Water quality and flow data overlap	> 2 years
Drainage area ratio between water quality site and gaged site	0.5 - 2.0
Proximity between water quality site and gaged site	< 40 km
Proximity between water quality site and gaged site for large streams (>260 km^2)	Must be on the same stream network
**Load Computations**	
Program for load computation	Fluxmaster (Schwarz et al. 2006)
Variables included in Fluxmaster	logarithm of flow, sine, cosine, decimal time
Time period of data used in Fluxmaster calibration	10/1/1970 - 9/30/2007
Time period for load computation	10/1/1970 - 9/30/2006
Annual load computation period	Water year 10/1 - 9/30
Detrended to which year (base year)	2002
**Point Sources**	
Point sources not included for the following Standard Industrial Classification (SIC) codes	1389- Oil and gas injection wells; 3312, 3479, 3339 - Steel; and 4961- steam.
**Model Calibration and Accumulation Procedures**	
SPARROW version	V2_9
Coefficient Estimation	Nonlinear least square regression (NLLSR)
Confidence limits on coefficients	Compute coeffcients with NLLSR followed by application of Make_coef_ci.sas code.
Robustness of coefficients	200 nonparametric bootstrap iterations
Accumulation at a HUC8 scale	Not corrected for biases. Accumulated with Custom_predict_accumulator.sas; Confidence intervals computed with Sparrow_custom_predict.sas using 200 iterations of parametric bootstraps.
Accumulation for all tributaries > 150 km^2	Corrected for biases. Accumulated with Custom_predict_accumulator.sas; Confidence intervals computed with Sparrow_custom_predict.sas using 200 iterations of parametric bootstraps.
Accumulation for each Great Lake	Corrected for biases.Accumulated with Custom_predict_accumulator.sas; Confidence intervals computed with Sparrow_custom_predict.sas using 200 iterations of parametric bootstraps.

S3. Summary of measured (with Fluxmaster) and estimated (with SPARROW) loads for each monitored site

In Tables S2_TP and S2_TN (attached to the end of the Supporting information), we provide a summary of measured (with Fluxmaster) and estimated (with SPARROW) loads for each monitoring site used in the calibration of the SPARROW models. In this table we also provide a summary of the location of each of the sites and the concentration data used to estimate the loads with Fluxmaster.

For each of the sites, there is a description of its location and the concentration data used in Fluxmaster. For each site, midmonthly mean and median concentrations for each site were calculated using a subset of the data. The subset included only samples collected closest to the middle of the month for sites at which more than one sample per month was collected. This was done to reduce possible bias associated with the frequency of sampling at different sites (such as weekly or storm sampling events compared to the more common monthly or less than monthly sampling).

The long-term mean annual nutrient loads for each monitored site were computed with the rating curve/regression procedure in the Fluxmaster computer program (Schwarz *et al.*, 2006). This procedure combines water-quality data at a monitoring station with daily flow values to provide more accurate load estimates than can be obtained by using individual water-quality measurements alone. TP and TN loads were determined with log-linear water-quality regression models that related the logarithm of constituent concentration to the logarithm of daily flow, decimal time (to compensate for trends), and season of the year (expressed using trigonometric functions of the fraction of the year). Regression models were fit to data from each potential load site (sites with $\geq$ 25 samples and corresponding long-term flow data, see Saad *et al.*, 2011 [this issue] for a more complete description).

4. Description of SPARROW input data

Water flowpaths used in the SPARROW models were defined by streams and reservoirs included in the enhanced stream-reach file 1 (RF1; 1:500,000 scale) with incremental reach catchments delineated with 100-m digital elevation models (Brakebill *et al.*, 2011; this issue). The catchments were used to allocate spatial data on nutrient sources and landscape and aquatic characteristics to each reach (Wieczorek and Lamotte, 2011) Unless otherwise noted all spatial data in this paper are from this source).

Nutrient inputs

Nitrogen from atmospheric deposition

The atmospheric deposition estimates used as input to SPARROW are based on the use of wet deposition measurements at National Atmospheric Deposition Program (NADP) sites as a proxy for total (wet plus dry) inorganic nitrogen deposition. SPARROW estimates of the quantities of nitrogen deposition delivered to streams is expected to account for additional contributions from dry nitrogen deposition forms because the regional patterns of wet and dry deposition are generally correlated over large areas of the U.S. (Holland et al. 2005; Baumgardner et al. 2002). The SPARROW estimates of atmospheric nitrogen contributions to streams would also be expected to primarily reflect *regional* atmospheric nitrogen sources, given that NADP wet-deposition estimates generally reflect regional NOx emissions from stationary sources (Elliott et al., 2007). Local atmospheric nitrogen sources, such as those associated with vehicle emissions, are likely to be included in the SPARROW estimates of the nitrogen contributions from other modeled sources, especially urban sources (e.g., developed or impervious land). Data were compiled from a 1-km grid constructed from the original data (J. Gronberg, USGS, written commun., 2005). These data were derived from long-term mean annual measurements (1990 to 2005) at 186 stations in the U.S. Estimates were normalized to the

base year 2002. Inputs were generally highest through the center of the MRB3 study area and lowest in extreme north and southeast parts (Figure S1).

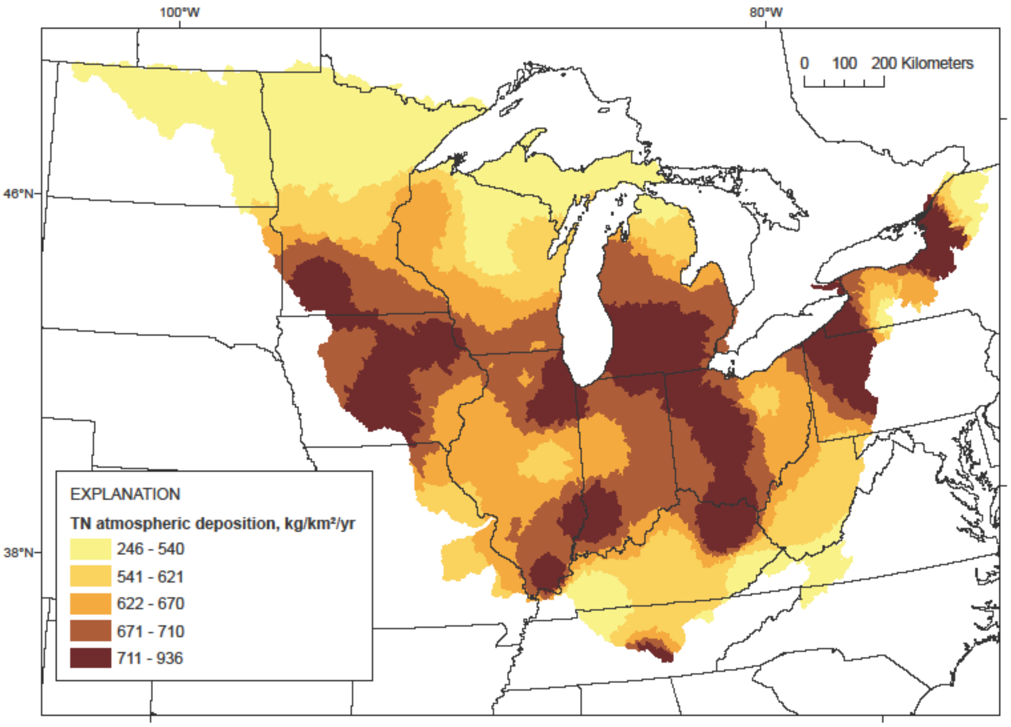

Figure S1. Atmospheric wet deposition rates for 2002 by SPARROW catchment, in kg/km^2. Intervals represent five equal quantiles (0-20%, 20-40%, 40-60%, 60-80%, 80-100%).

Point sources

Inputs from point sources (including sewerage treatment, commercial, and industrial effluent) were estimated by Maupin and Ivahnenko (2011; *this issue*) from data in the USEPA's PCS database supplemented with data obtained directly from the states of Wisconsin and Minnesota (J. Schmidt, Wisconsin Department of Natural Resources, and S. Weiss, Minnesota Pollution Control Agency, written commun., 2007). TP and TN effluent loads were computed for each point source location using methods and procedures described by McMahon *et al.* (2007) and Hoos *et al.* (2008). For MRB3 SPARROW models, inputs from the following oil and gas, steel, and stream Standard Industrial Classifications (SIC) were excluded: 1389, 3312, 3339,

3479, and 4961. The point source load estimates used in the model calibration were based on estimates for 2002. If the 2002 data were missing and 1997 data were available, then data from 1997 were used. If both 2002 and 1997 data were missing and 1992 were available, then 1992 data were used. The distribution of point source inputs of P and N were quite variable, but largest inputs were concentrated around major urban areas (Figure S2).

A.

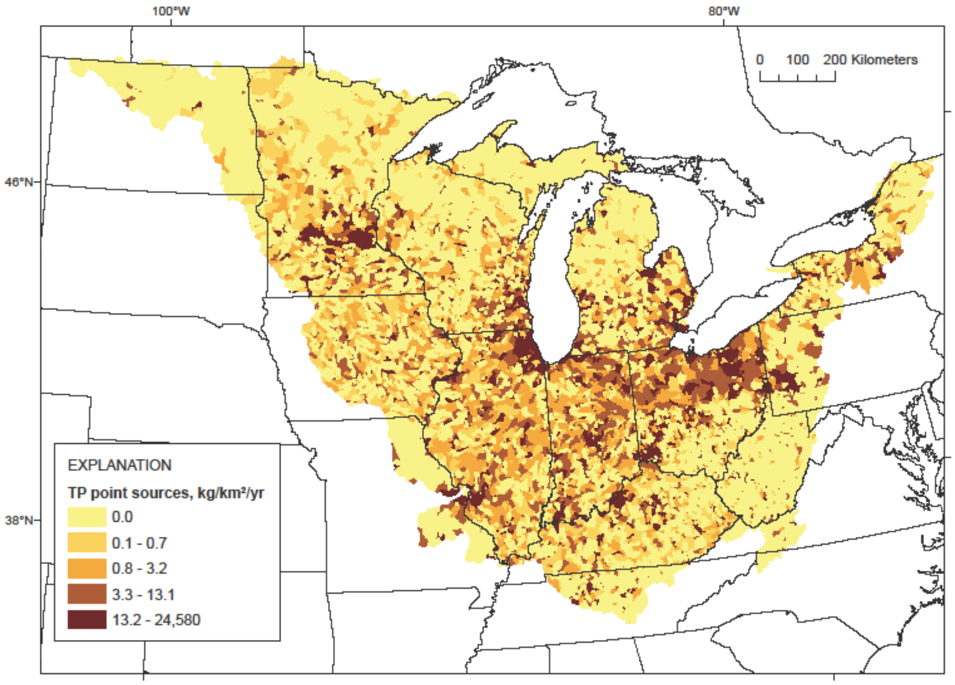

B.

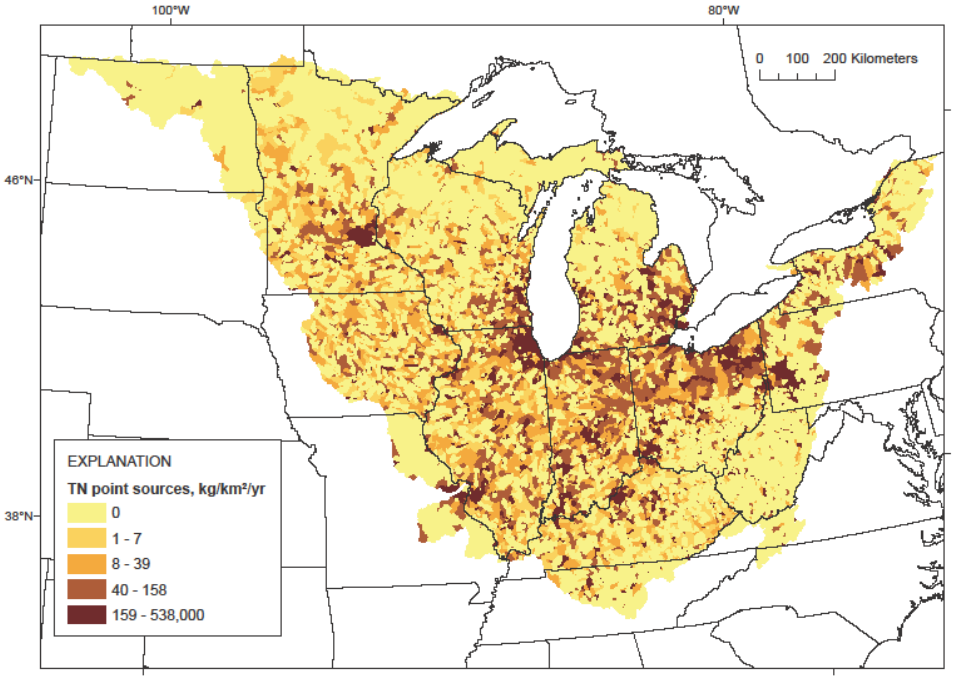

Figure S2. Point source input rates for TP (A) and TN (B) for 2002 by SPARROW catchment, in kg/km^2. Intervals represent five equal quantiles (0-20%, 20-40%, 40-60%, 60-80%, 80-100%).

Nutrients from farm fertilizer

Fertilizer inputs are based on county-level estimates from Ruddy *et al.* (2006). The P and N inputs from farm fertilizers were derived from 2002 county sales aggregated by state and allocated back to the individual counties based on their respective fertilizer expenditures (Association of American Plant Food Control Officials and the U.S. Census of Agriculture). The county data were then allocated to each SPARROW stream catchment by the fraction of the catchment's agricultural land. Farm N and P fertilizer inputs had the same pattern, and inputs were highest in the central and western parts of MRB3 (Figure S3). Generally, N inputs were about an order of magnitude greater than P inputs.

A.

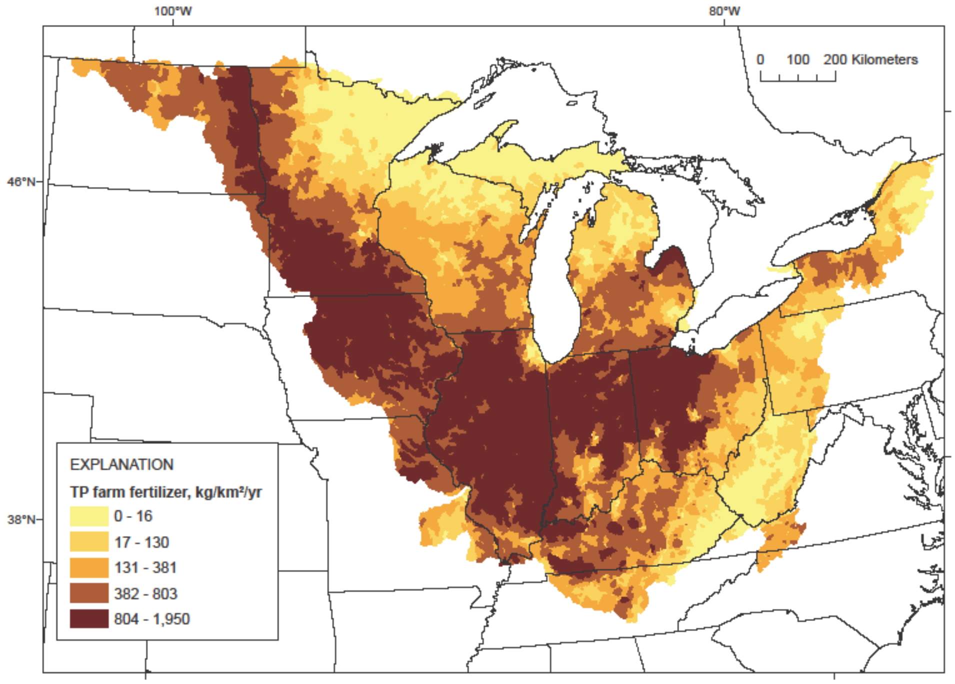

B.

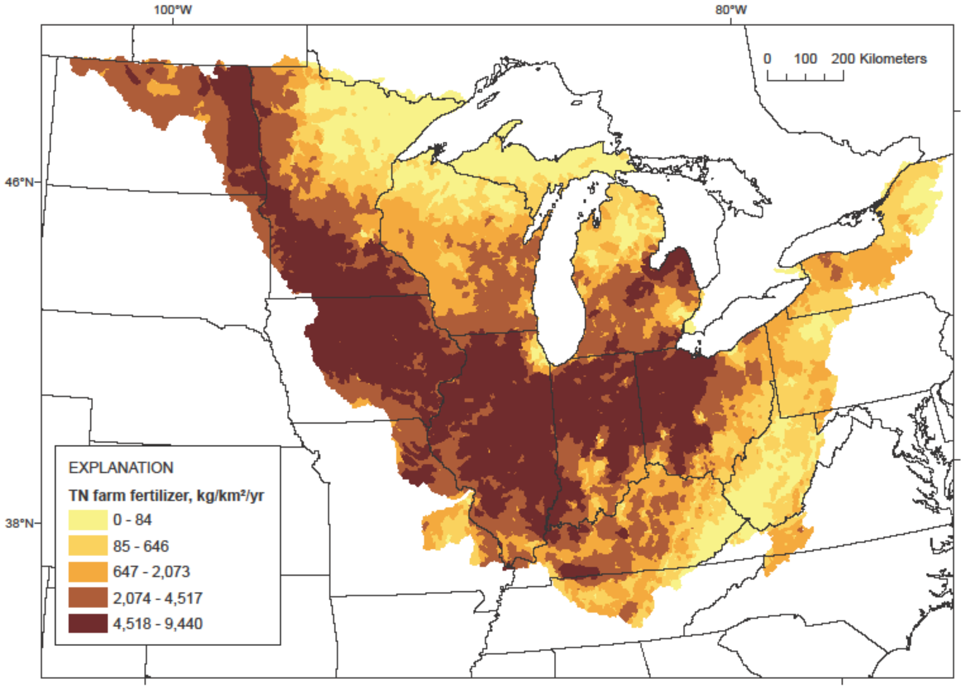

Figure S3. Fertilizer input rates for TP (A) and TN (B) for 2002 by SPARROW catchment, in kg/km^2. Intervals represent five equal quantiles (0-20%, 20-40%, 40-60%, 60-80%, 80-100%).

Nutrients from confined and unconfined manure

Manure inputs are based on county-level estimates of animal wastes from Ruddy *et al.* (2006). Nutrients associated with livestock wastes reflect contributions from the excreted wastes of *unconfined* animals on farms, pastures, and rangelands and from the excreted wastes of *confined* animals, including those in concentrated animal feeding operations. Confined animal wastes include recoverable manure that may be applied to nearby farmlands as well as unrecoverable manure that is lost during the collection, storage, and treatment of the waste. Manure inputs were derived from 2002 county livestock population data from the U.S. Census of Agriculture using species specific rates. The county data were then allocated to each SPARROW stream catchment by the fraction of the catchment's agricultural land and grasslands. Highest confined manure input rates for P occurred in southern Minnesota, Iowa, southwestern Wisconsin, and northern Indiana, whereas highest unconfined manure input rates for P occurred in eastern Iowa, western Kentucky, and Tennessee (Figure S4). The distribution of confined manure input rates for N is similar to that for P, highest inputs occurred in southern Minnesota, Iowa, southwestern Wisconsin, and northern Indiana (Figure S5).

A.

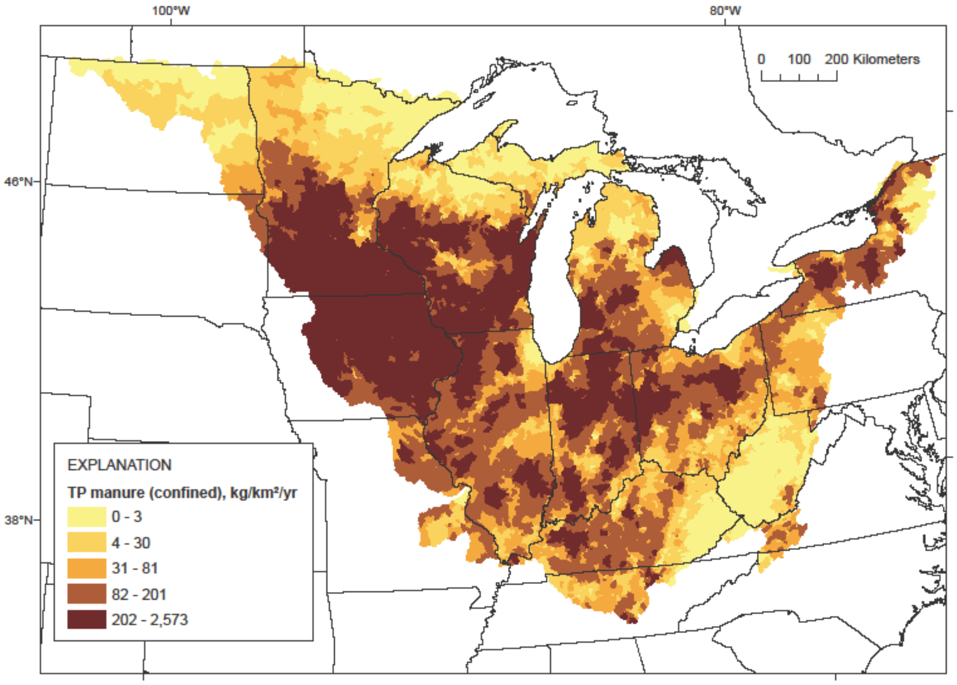

B.

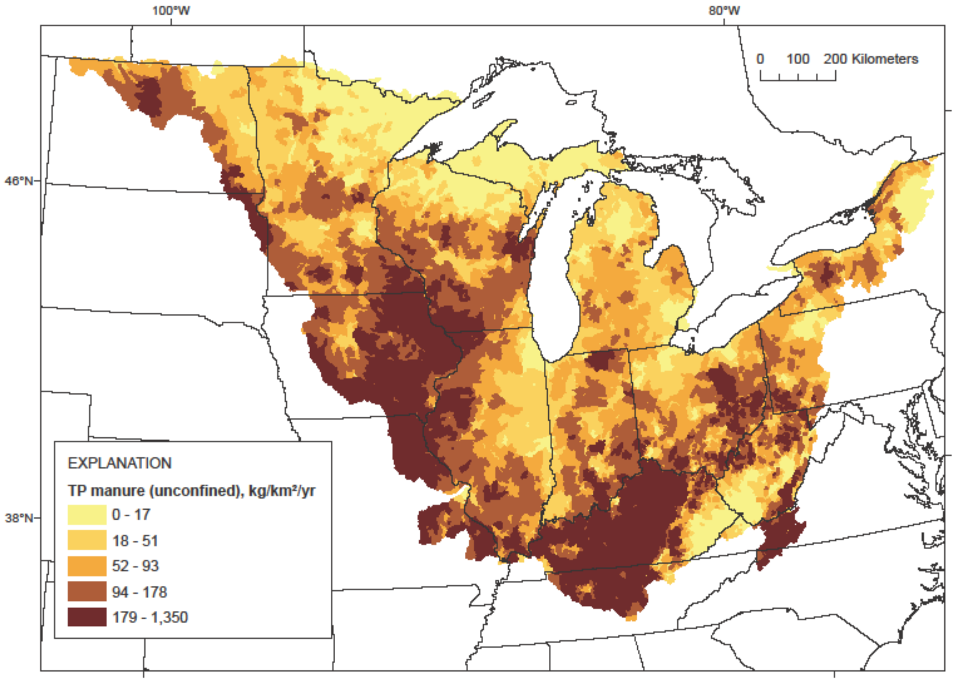

Figure S4. Manure input rates for TP for confined animals (A) and unconfined animals (B) for 2002 by SPARROW catchment, in kg/km^2. Intervals represent five equal quantiles (0-20%, 20-40%, 40-60%, 60-80%, 80-100%).

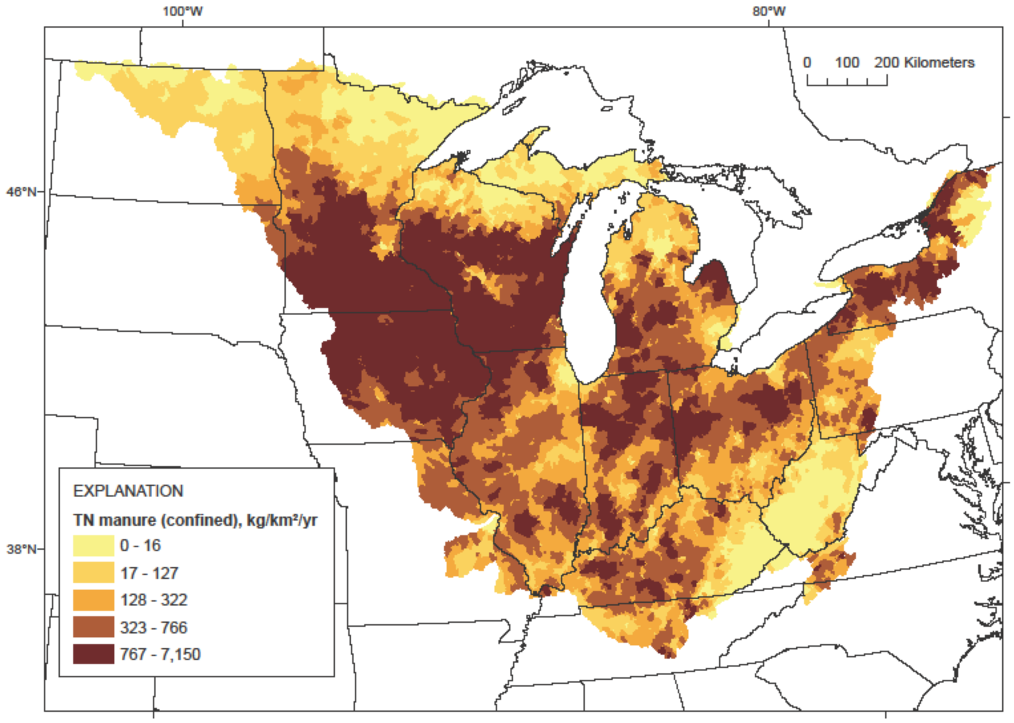

Figure S5. Manure input rates for TN for confined animals for 2002 by SPARROW catchment, in kg/km^2. Intervals represent five equal quantiles (0-20%, 20-40%, 40-60%, 60-80%, 80-100%).

Inputs from specific land-use/land-cover types

Land-use/land-cover related inputs (additional agricultural inputs from cultivated agricultural areas, urban inputs from urban and open areas, and natural inputs from forested areas) were based on the respective amount of area in each of these land types based on 2001 National Land Cover Data (USGS, 2000). The area consists primarily of forested areas in north and southeast, and agricultural areas in the west and central areas. Several major metropolitan areas are in the basin, such as Chicago, IL, Detroit MI, and Cleveland, OH (Figure S6).

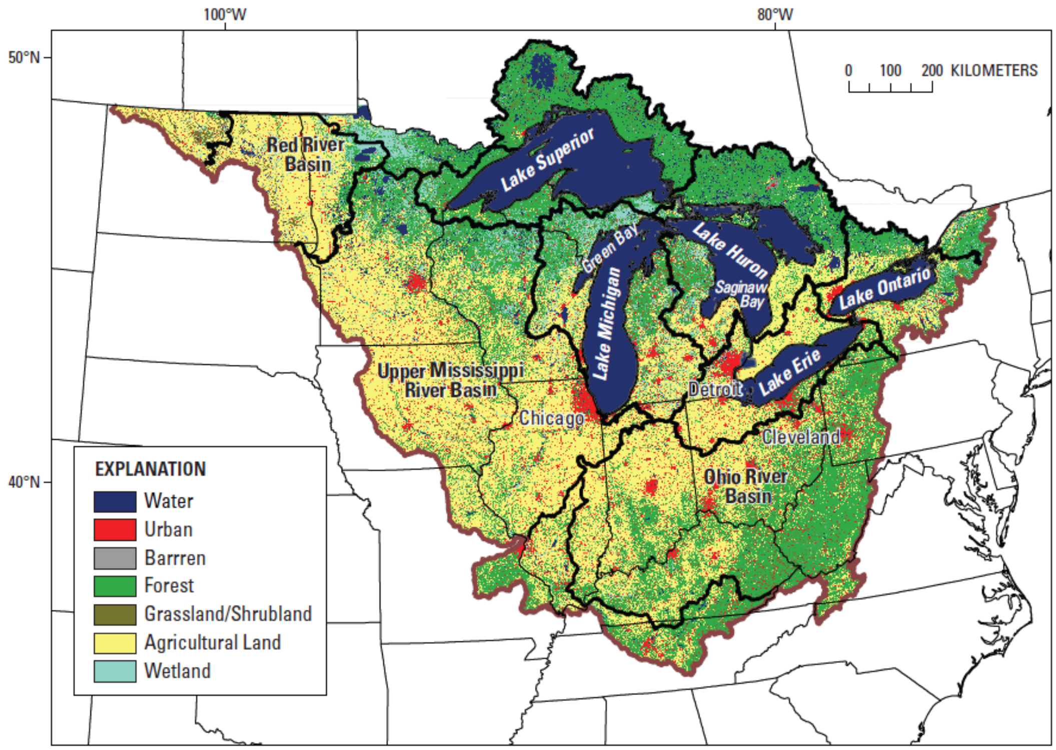

Figure S6. Land use and land cover across the Great Lakes Basin with selected metropolitan centers identified (U.S. drainage, U.S. Geological Survey, 2000; Canadian drainage, Geobase, 2009).

Environmental-setting information

Soil permeability

Average soil permeability was compiled from the STATSGO database (U.S. Department of Agriculture; http://water.usgs.gov/GIS/metadata/usgswrd/XML/muid.xml accessed July 2009) using methods described by Wolock (1997). Soils with relatively high permeability are in the northern part of MRB3 (eastern Minnesota, Wisconsin, and Michigan) and soils with relatively poor permeability are in the south central part of MB3 (Figure S7).

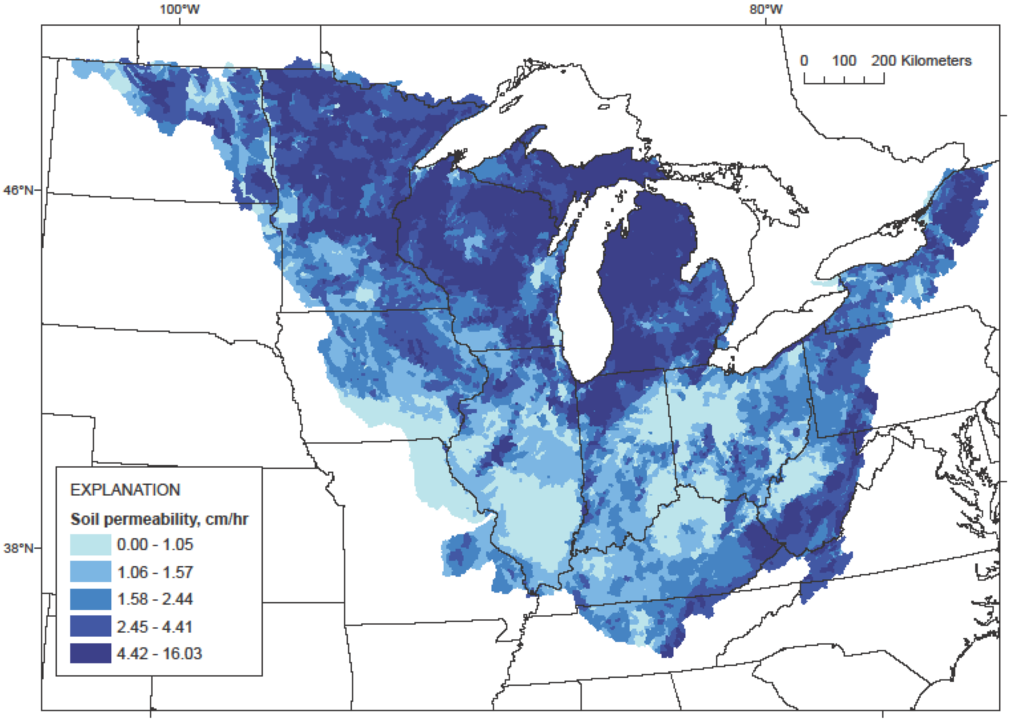

Figure S7. Average soil permeability for MRB3, by SPARROW catchment. Intervals represent five equal quantiles (0-20%, 20-40%, 40-60%, 60-80%, 80-100%).

Areas of tile drainage

Tile drain information was derived from the 1997 National Resources Inventory dataset compiled by the National Resource Conservation Service. The distribution of tile drains is inversely related to the permeability of the soil. The highest percentage of land with tile drains is found in the central part of MRB3 (southern Minnesota, Iowa, southeastern Wisconsin, central Illinois, southern Michigan northern Indiana, and northern Ohio), where the soils are least permeable (Figure S8).

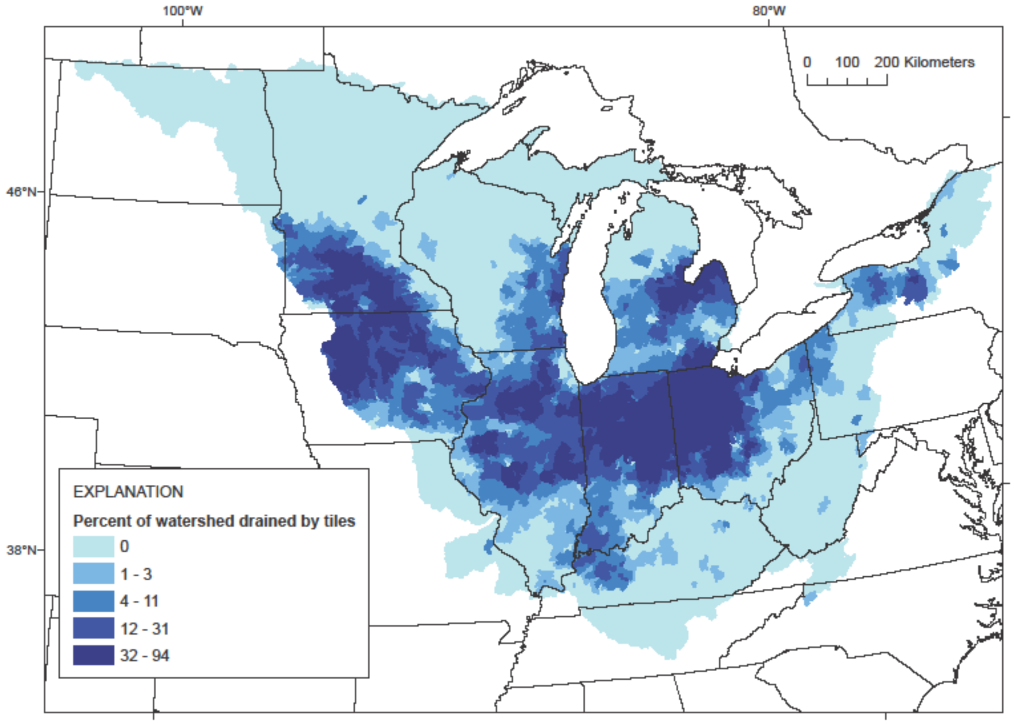

Figure S8. Percentage of the area drained by tiles for MRB3, by SPARROW catchment. Intervals represent five equal quantiles (0-20%, 20-40%, 40-60%, 60-80%, 80-100%).

Drainage density

The drainage density of each catchment was calculated as the total length of all stream reaches in a catchment divided by its area. Stream reaches were obtained from the enhanced stream-reach file 1 (RF1; 1:500,000 scale) (Brakebill *et al.*, 2011; this issue). The highest drainage density is in the southeast part of MRB3, which was partly caused by the increased precipitation in this area and the increased resolution in the enhanced RF1 coverage. Lowest drainage densities are in the northwest part of MRB3.

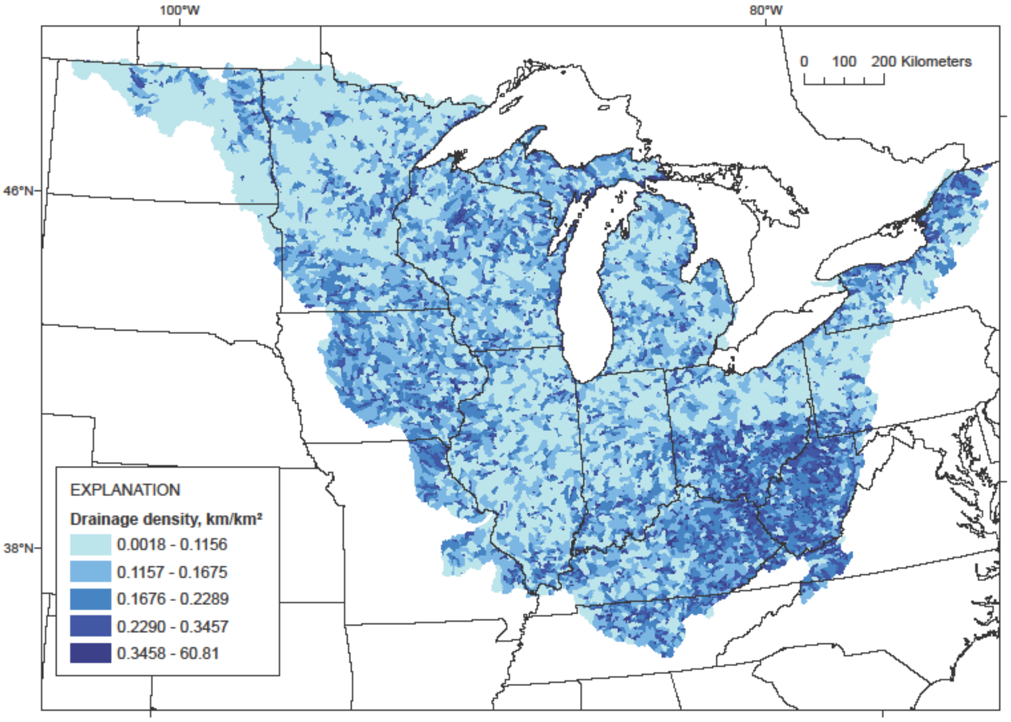

Figure S9. Drainage density (summed length of all stream reaches in each catchment divided by its area) for MRB3, by SPARROW catchment. Intervals represent five equal quantiles (0-20%, 20-40%, 40-60%, 60-80%, 80-100%).

Precipitation

Mean annual precipitation represent the average annual values over 30 years (1971 to 2000), and was obtained from the Parameter-elevation Regressions on Independent Slopes Model (PRISM) digital data network (http://www.prism.oregonstate.edu/). These data show a strong northwest to southeast gradient of increasing mean precipitation (Figure S10). Mean annual precipitation ranges from 380 to 1,700 mm.

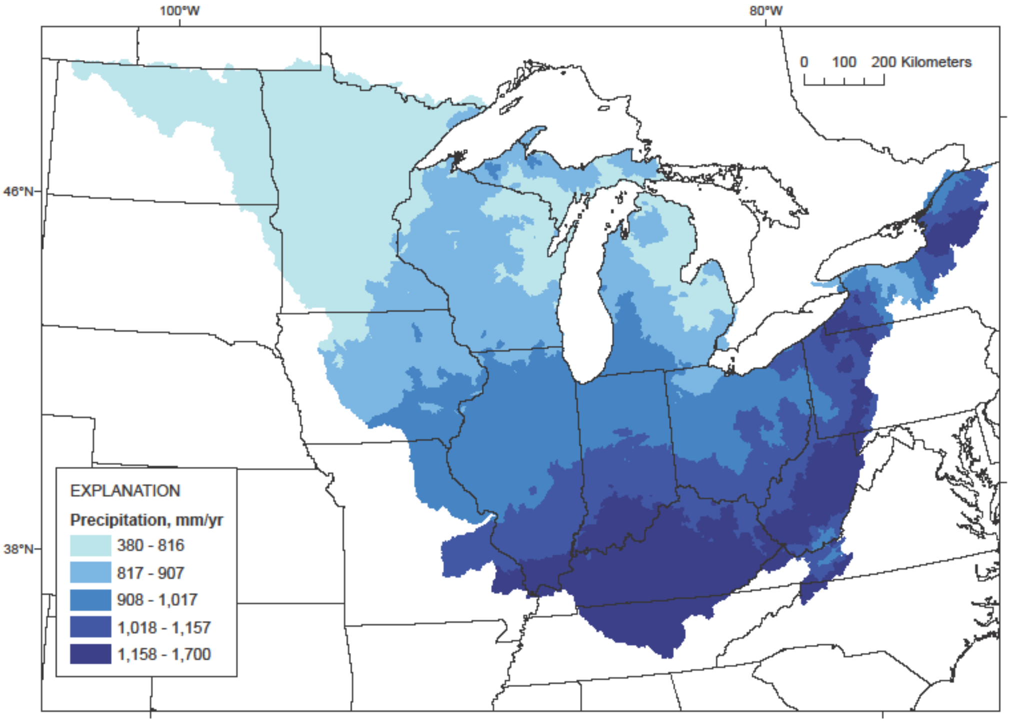

Figure S10. Mean annual precipitation (30 year average from 1971–2000) for MRB3, by SPARROW catchment. Intervals represent five equal quantiles (0-20%, 20-40%, 40-60%, 60-80%, 80-100%).

Air temperature

Air temperature data, averaged from minimum and maximum daily temperature values over 30 years (1971 to 2000), were obtained from the Parameter-elevation Regressions on Independent Slopes Model (PRISM) digital data network (http://www.prism.oregonstate.edu/). These data show a strong north to south gradient of increasing mean air temperature (Figure S11). Mean air temperatures range from 2.3 to 14.9 C.

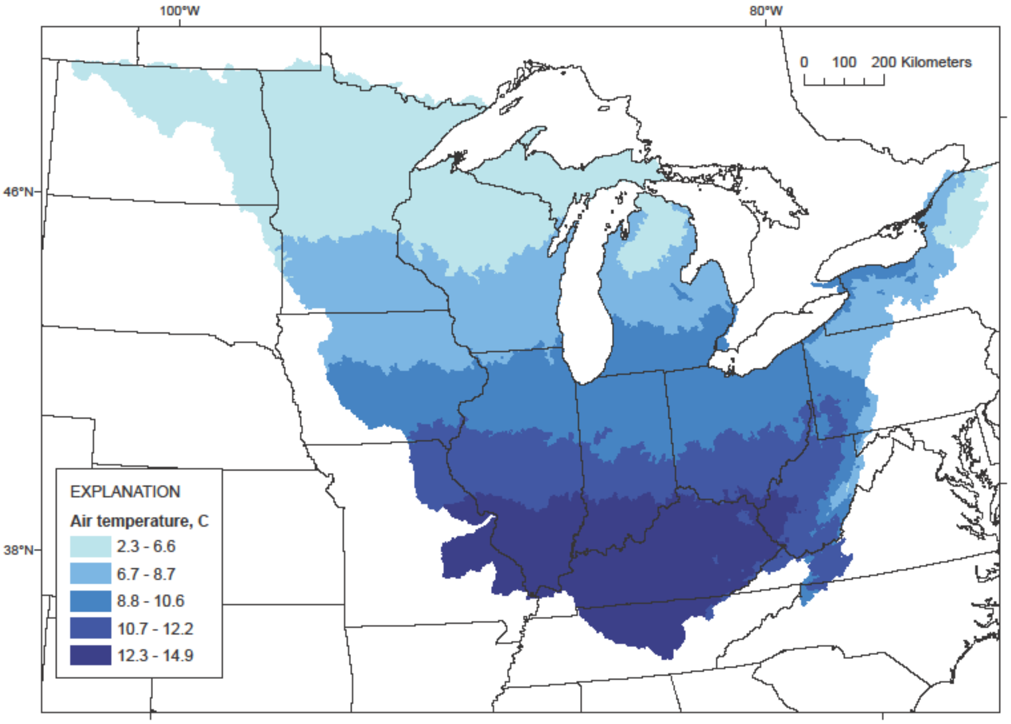

Figure S11. Mean annual air temperatures (30 year average from 1971–2000) for MRB3, by SPARROW catchment. Intervals represent five equal quantiles (0-20%, 20-40%, 40-60%, 60-80%, 80-100%).

Soil clay content

Soil clay content (percentage) was compiled from the STATSGO database (U.S. Department of Agriculture; http://water.usgs.gov/GIS/metadata/usgswrd/XML/muid.xml accessed July 2009) using methods described by Wolock (1997). Soils with the lowest clay content are in central Minnesota, northern Wisconsin, and Michigan. Soils with highest clay content occur are in the southern part of MRB3 (Figure S12). Soil clay content is inversely related to soil permeability.

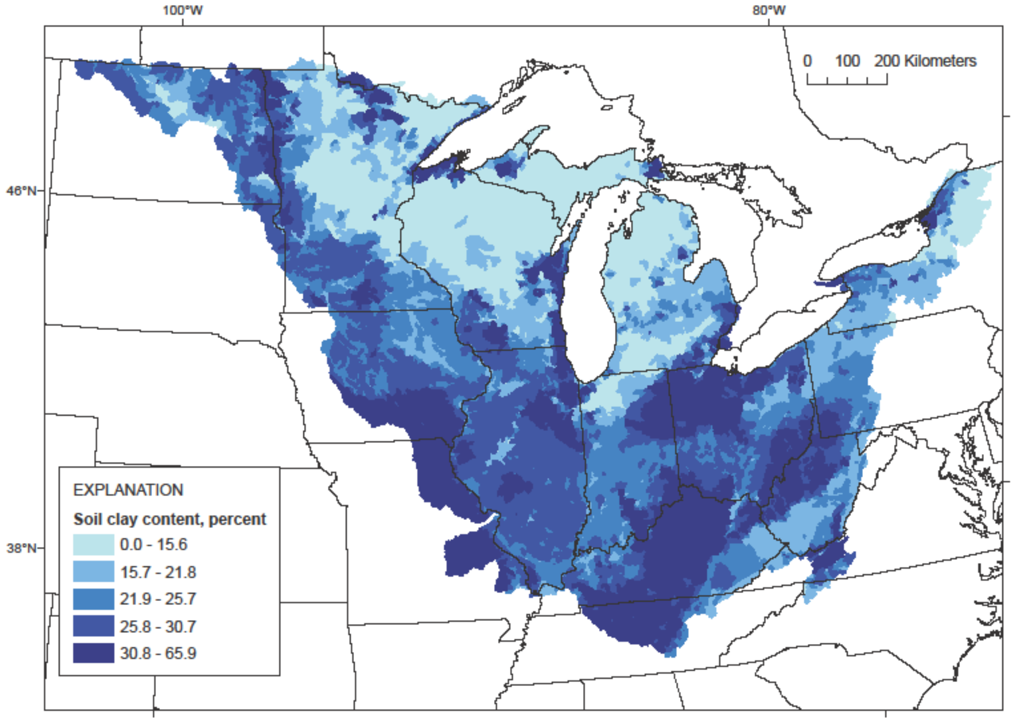

Figure S12. Average soil permeability for MRB3, by SPARROW catchment. Intervals represent five equal quantiles (0-20%, 20-40%, 40-60%, 60-80%, 80-100%).

Stream and reservoir information

Time of travel, based on stream velocity, was the factor used to describe nutrient removal (loss) in streams. Mean annual velocity estimates were estimated as a function of the mean annual flow and stream order (ERF1 reference). Mean annual flow for each reach was estimated from flows measured from 1975 to 2007 from gages throughout the country (D. Wolock, USGS, written commun., 2009). Discharges were subdivided into three categories to describe instream loss. The values classifying each category were determined in the calibration process for the SPARROW TP and TN models. For the SPARROW TP model, the three categories of streams were: small, flow <~1.4 m^3/s or <50 ft^3/s; medium, flow ~1.4–2.3 m^3/s or 50–80 ft^3/s; and large, flow >~2.3 m^3/s; 80 ft^3/s) (Figure S13). For the SPARROW TN model, the three categories of streams were: small, flow <~1.1 m^3/s or <40 ft^3/s; medium, flow ~1.1–2.0 m^3/s or 40–70 ft^3/s; and large, flow >~2.0 m^3/s; 70 ft^3/s) (Figure S14).

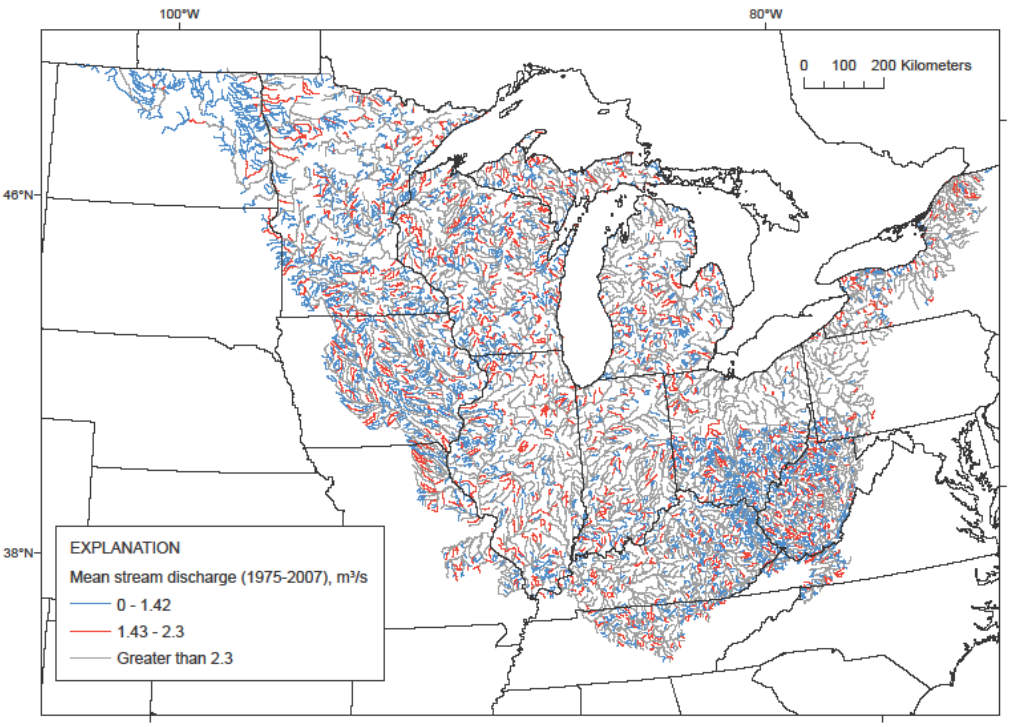

Figure S13. Stream discharge associated with reach loss variables in the MRB3 SPARROW TP model.

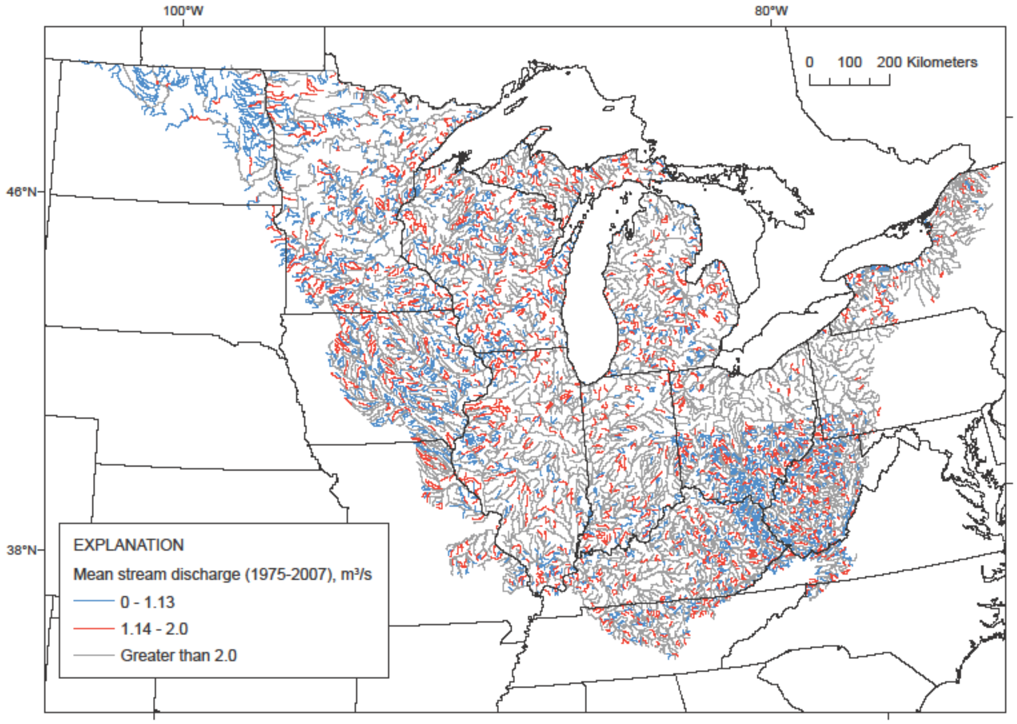

Figure S14. Stream discharge associated with reach loss variables in the MRB3 SPARROW TN model.

Hydraulic loading was used to describe nutrient removal in reservoirs (Schwarz *et al*. 2006). Hydraulic loading for each reservoir was calculated as average flow divided by reservoir surface area based on information from the National Inventory of Dams (USACE, 2007). The reservoirs used in the SPARROW models are shown in Figure S15. Nutrient losses in reservoirs that are not included in this coverage were assumed to occur as part of instream loss.

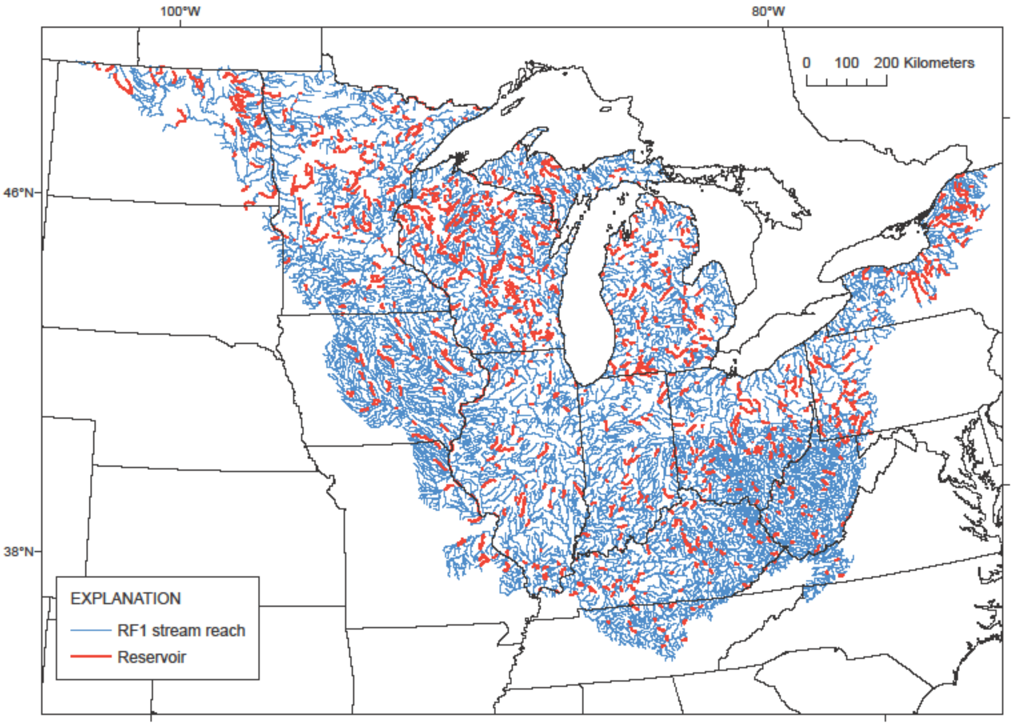

Figure S15. RF1 stream reaches and reservoirs used in the MRB3 SPARROW models.

5. SPARROW model calibration.

In the model calibration process, the studentized residuals (difference in the logarithmically transformed observed and simulated loads) were evaluated for evidence of local or regional biases based on visual inspections of residual maps (Figure S16). In the calibration process, the model used the monitored transport from each upstream basin rather than the model-estimated transport to represent the load leaving the upstream basin in an attempt to eliminate prediction errors from cascading downstream to other monitored sites. In Figure 2 of the main manuscript, the full model predictability is evaluated, because errors are allowed to cascade downstream to other monitored sites. In Figure 2 of the main manuscript, full prediction errors are reported; however, in Figure S16, the residuals in the calibration process are using studentized residuals are reported to enable comparisons in the calibration of other MRB SPARROW models.   Overall, the models showed little signs of regional biases.

A.

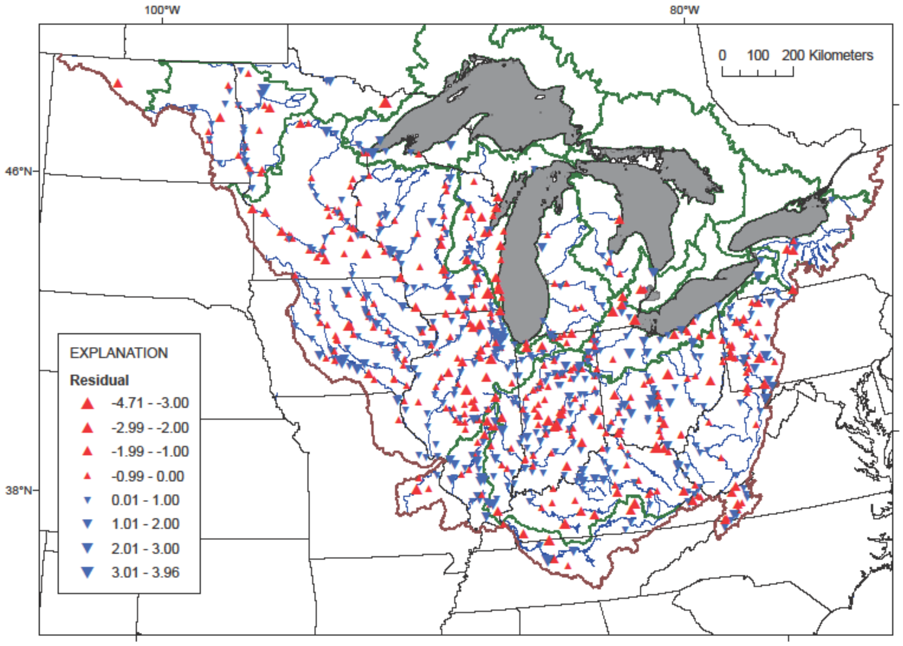

B.

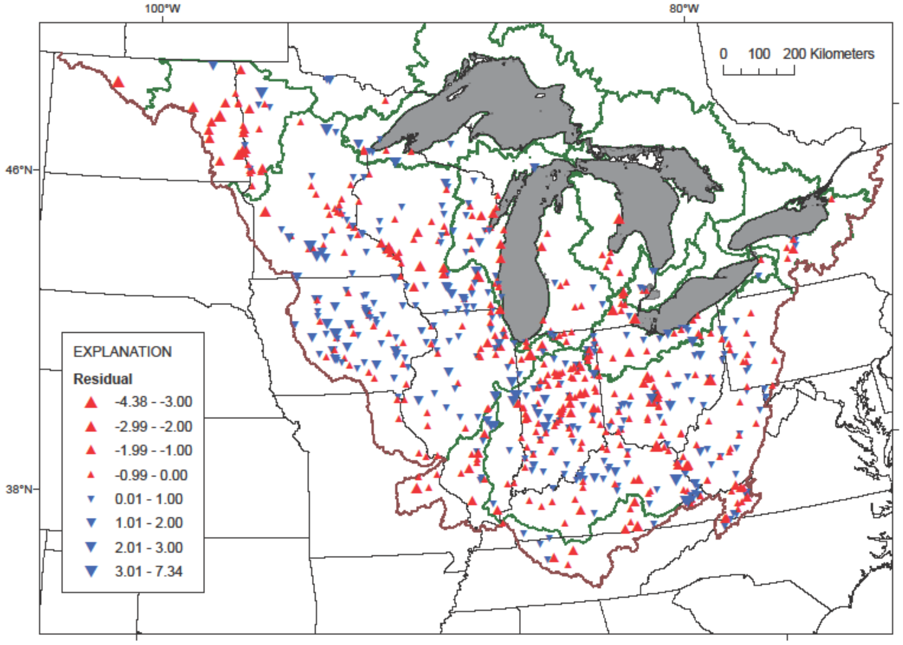

Figure S16. Model residuals for stations used to calibrate the final SPARROW models of (A) TP and (B) TN. The residuals are expressed in standardized (studentized) units for the standard normal distribution. The residuals are expressed as the difference in the logarithmically transformed observed and simulated loads: overpredictions are negative values and red triangles, and underpredictions are positive values and blue triangles.

6. Output from the MRB3 SPARROW TP and TN models for each Great Lake and nearby large river basin (Tables S3 and S4), each HUC8 (Table S5) and each tributary with a drainage basin greater than 150 km^2 (Table S6).

The total TP and TN load and yield to each Great Lake and nearby large river basin is provided in Tables S3 and S4. The percent contribution of each source of nutrients is given in each table.

Table S3. Total annual phosphorus loads and yields for each Great Lake and major river basin in Major River Basin 3, with the percent contribution by source.

All loads and yields from individual tributaries were adjusted to remove known spatial biases by only predicting areas that are not monitored.

Supplemental Material to Robertson and Saad, 2011, Journal of the American Water Resources Association, Nutrient Inputs to the Laurentian Great Lakes By Source and River Basin Estimated Using SPARROW Watershed Models.

[kg, kilogram; km^2, square kilometer; Yield is load per unit area of the watershed]

					Percent Contribution by Source					
Great Lake/Major River Basin	Drainage area (km^2)	Total annual load (Tonnes)	Annual Yield (kg/km^2)	Delivery Ratio	Forest, wetland, shrub	Point sources	Urban and open areas	Fertilizers (farm)	Manure (confined)	Manure (unconfined)
Superior	43,594	782	17.9	0.92	69.1	13.6	10.3	0.8	3.8	2.4
Michigan	116,395	3,430	29.5	0.86	11.7	31.4	15.4	12.7	25.1	3.7
Huron	41,369	927	22.4	0.91	13.5	35.4	18.4	17.1	13.0	2.6
Erie	55,488	4,610	83.1	0.96	4.3	42.1	10.0	26.3	15.4	1.9
Ontario	35,661	1,800	50.5	0.89	13.1	44.3	6.2	12.1	20.4	3.9
Red R.	84,508	1,940	23.0	0.62	5.5	6.5	7.2	66.9	8.6	5.4
Upper Miss. R.	446,475	31,400	70.3	0.79	4.5	21.0	6.3	30.0	30.6	7.6
Ohio R.	373,067	27,700	74.2	0.92	18.1	21.1	8.9	23.7	17.4	10.8

[a]Delivery ratio is computed as the delivered load divided by the total non-decayed load.

Table S4. Total annual nitrogen loads and yields for each Great Lake and major river basin in Major River Basin number 3 (MRB#3), with the percent contribution by source.

All loads and yields from individual tributaries were adjusted to remove known spatial biases by only predicting areas that are not monitored.

Supplemental Material to Robertson and Saad, 2011, Journal of the American Water Resources Association, Nutrient Inputs to the Laurentian Great Lakes By Source and River Basin Estimated Using SPARROW Watershed Models.

[kg, kilogram; km^2, square kilometer; Yield is load per unit area of the watershed]

					Percent Contribution by Source				
Great Lake/Major River Basin	Drainage area (km^2)	Total annual load (Tonnes)	Annual Yield (kg/km^2)	Delivery Ratio	Atmospheric deposition in watershed	Point sources	Additional agricultural sources	Fertilizers (farm)	Manure (confined)
Superior	43,594	10,900	250	0.91	77.8	15.6	2.4	1.1	3.0
Michigan	116,395	70,000	601	0.84	29.3	22.4	12.8	18.1	17.5
Huron	41,369	25,900	626	0.89	29.3	13.1	17.7	29.2	10.7
Erie	55,488	136,000	2,450	0.96	17.4	25.1	18.3	32.1	7.2
Ontario	35,661	32,800	920	0.86	32.7	34.2	7.5	8.6	17.1
Red R.	84,508	14,200	168	0.59	16.0	3.3	35.6	42.9	2.2
Upper Miss. R.	446,475	480,000	1,080	0.74	19.4	7.9	20.5	35.1	17.2
Ohio R.	373,067	357,000	957	0.90	35.2	12.8	14.3	27.3	10.4

[a]Delivery ratio is computed as the delivered load divided by the total non-decayed load.

The total TP and TN load and yield from each 8-digit Hydrologic Unit Code (HUC8; Seaber *et al.*, 1987) watershed throughout MRB3 and is provided in Table S5. The incremental nutrient load (load generated from within a HUC8 and delivered to its most downstream reach) was computed for each HUC8 watershed by summing the loads from each reach within the HUC8 watershed. The incremental yield was then computed by dividing the incremental load by the total area of the HUC8 watershed.

The lower and upper 95% confidence limits for the HUC8 loads and yields are also provided in Tables S5_TP and S5_TN (attached to the end of the Supporting information). The actual delivered fluxes computed in SPARROW are assumed to depend on a multiplicative error term that represents other sources and processes not included in the SPARROW analysis. Because of this residual term, and because the determination of the predicted flux depends on coefficients that are estimated via statistical methods, the delivered yields across HUC8 watersheds are subject to uncertainty. Because of the nonlinear manner in which the estimated coefficients enter the model, it was necessary to use bootstrap methods (Schwarz *et al.*, 2006) to assess the uncertainty. Bootstrap analyses were also used to correct for potential bias caused by log retransformations in the yield predictions. A brief summary of the bootstrap method is presented here; a full description of the bootstrap methodology is described by Robertson *et al.* (2009).

The bootstrapping method was implemented by performing 200 repeated calibrations of the SPARROW model using randomly selected integer weights (which sum to the total number of monitored reaches in both the models) applied to each of the squared residuals at monitored reaches, resulting in 200 realizations of the estimated coefficients, yields, delivered yields, and residuals. The distribution of the estimated delivered yields for each reach from the 200 iterations was used to estimate the standard errors in the yields from SPARROW. The estimated

confidence interval for delivered yields required explicit consideration of the distribution of residuals in the model, rather than just the summary statistical properties of the residuals. The bootstrap method for incorporating the distribution of the model residuals is based on the empirical distribution of the combined bootstrap-iteration estimate of the modeled component of delivered flux and a randomly selected weighted error from the original monitored values obtained in the original calibration of the model. The 95% confidence interval for the delivered incremental yield from each HUC8 was then estimated using a ratio formulation of the hybrid bootstrap confidence limit (see Shao and Tu, 1995, and Schwarz *et al.*, 2006).

The total load and yield of TN and TP from each tributary (> 150 km^2) to each Great Lake is provided in Tables S6_TP and S6_TN (attached to the end of the Supporting information). The lower and upper 95% confidence limits for the loads are also in Tables S6_TP and S6_TN. For each Great Lake, the tributaries are ranked on the basis of their respective loads and yields. It should be noted that SPARROW was only used to estimate the loads from the unmonitored part of each basin, and the confidence limits only represent the uncertainty in that portion of the load (model estimated load); no errors are assumed in the loads estimated with Fluxmaster. The percent contribution of each source of nutrients is also provided in Tables S6_TP and S6_TN.

7. Comparison in yields from the Great Lake Basins with those from nearby large river basins

Previous large-scale loading studies (Smith *et al.*, 1997) have demonstrated that highest nutrient yields in the U.S. were from watersheds in the Midwest (primarily Iowa, Illinois, Indiana, and western Ohio), part of which drain to the Great Lakes. Many actions have been taken to reduce P loading to the Great Lakes since the early 1970s. To see if these actions have resulted in lower yields compared with those from watersheds outside of the Great Lakes watershed, yields from Great Lake watersheds are compared with those draining away from the Great Lakes. Results from this study indicate that there are a few areas in the Great Lakes Basin that have yields similar to those found in the intensively agricultural areas of the Upper Midwest: western Ohio (identified in the previous studies), and areas in central Wisconsin and Michigan. To determine how yields from the individual lake basins compare with the nearby large river basins, yields to each Great Lake are compared with those from the Upper Mississippi, Ohio, and Red Rivers in Figure S17.

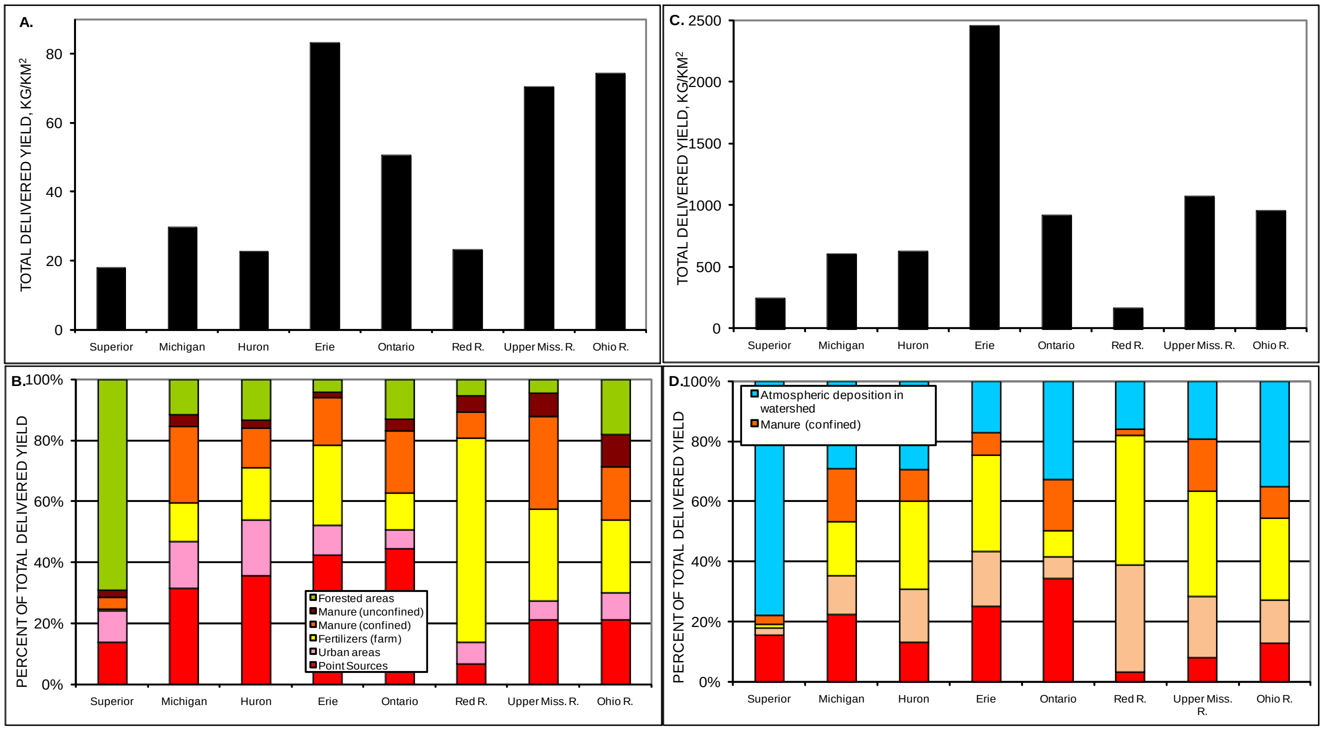

Figure S17. Total delivered yield from the watershed of each Great Lake and nearby large river basins for TP and TN, with the percent contributed by each source.

The TP yield from the Lake Erie Basin is slightly higher than those from the Upper Mississippi and Ohio River Basins primarily resulting from the Lake Erie Basin having more point-source inputs (Figure S17). In general, point sources were more important to almost all of the Great Lakes than to the large nearby rivers (7–21% for the large rivers compared to 31–44% for all of the Great Lakes except Lake Superior). Although the land uses vary, TP yields from the Lake Superior and Huron Basins are similar to that of the more intensively farmed Red River Basin. The low yields from the Red River Basin may have resulted from the lower runoff in northern Minnesota and North Dakota than around Lakes Superior and Huron, rather than lower nutrient concentrations in the streams. TP concentrations in streams in the Red River Basin have been shown to be as high as those as many of the intense agricultural areas around the other lakes (Lorenz *et al.*, 2009). The Red River Basin had the highest percentage of TP coming from fertilizer sources, but the lowest percentage coming from point sources.

TN yield from the Lake Erie Basin is almost twice those from the Upper Mississippi and Ohio River Basins, whereas TN yield from the Lake Ontario Basin is similar to those from the large river basins. The higher yield from the Lake Erie Basin was primarily due to it having more input from point sources and streams with shorter travel times and thus less time for losses, such as from denitrification. Point sources of N were more important to most of the Great Lakes than to the large nearby rivers (13–34% for the Great Lakes compared to 3–13% for these large rivers). TN yield from the Red River Basin was less than those from all of the Great Lake Basins. Low TN yields from the Red River Basin, again, may have been caused by less runoff in northern Minnesota and North Dakota than in the forested areas around Lakes Superior and Huron (Lorenz *et al.*, 2009). The Red River watershed had the highest percentage of N coming from agricultural sources, but the lowest percentage coming from point sources.

8. References to Supporting Information

Alexander, R.B., R.A. Smith, G.E. Schwarz, E.W. Boyer, J.V. Nolan, and J.W. Brakebill, 2008. Differences in phosphorus and nitrogen delivery to the Gulf of Mexico from the Mississippi River Basin. Environmental Science and Technology 42(3):822-830.

Baumgardner, R.E., T.F. Lavery, C.M. Rogers, and S.S. Isil, 2002. Estimates of the Atmospheric Deposition of Sulfur and Nitrogen Species: Clean Air Status and Trends Network, 1990-2000. Environ. Sci. Technol., 36:2614-2629.

Brakebill, J.W., D.M. Wolock, and S.E. Terziotti, this issue. Digital Hydrologic Networks Supporting Applications Related to Spatially Referenced Regression Modeling. Journal of the American Water Resources Association, doi: 10.1111/j.1752-1688.2011.00578.x.

Elliott, E.M., C. Kendall, S.D. Wankel, D.A. Burns, E.W. Boyer, K. Harlin, D.J. Bain, and T.J. Butler, 2007. Nitrogen Isotopes as Indicators of NOx Sources Contributions to Atmospheric Deposition across the Midwestern and northeastern United States, Environ. Sci. Technol., 41:7661-7667.

Geobase, 2009. Land cover, circa 2000-verctor (Canada). Available at http://www.geobase.ca/ geobase/en/data/landcover/index, accessed on September 28, 2009.

Holland, E.A., B.H. Braswell, J. Sulzman, and J.F. Lamarque, 2005. Nitrogen Deposition on the United States and Western Europe: Synthesis of Observations and Models, Ecological Applications, 15:38-57.

Lorenz, D.L., D.M. Robertson, D.W. Hall, and D.A. Saad. 2009. Trends in streamflow, and nutrient and suspended sediment concentrations and loads in the Upper Mississippi, Ohio, Red, and Great Lakes River Basins 1975–2004: U.S. Geological Survey Scientific Investigations Report 2008–5213, Reston, VA, 81 p.

Maupin, M.A. and T. Ivahnenko, this issue. Nutrient Loadings to Streams of the Continental United States From Municipal and Industrial Effluent. Journal of the American Water Resources Association, doi: 10.1111/j.1752-1688.2011.00576.x.

McMahon, G., L. Tervelt, and W. Donehoo, 2007. Methods for estimating annual wastewater nutrient loads in the southeastern United States: U.S. Geological Survey Open-File Report 2007–1040, Reston, VA, 81 p.

PRISM (Parameter-elevation Regressions on Independent Slopes Model) Climate Group, 2009, Oregon State University, Available at http://prism.oregonstate.edu, accessed on August 31, 2009.

Robertson, D.M., W.J. Rose, and P.F. Juckem, 2009, Water quality and hydrology of Whitefish Lake, Douglas County, Wisconsin, with special emphasis on the responses of an oligotrophic seepage lake to changes in phosphorus loading and water level. U.S. Geol. Survey Scientific Invest. Report 2009–5089, Reston, VA, 41 p.

Robertson, D.M., G.E. Schwarz, D.A. Saad, and R.B. Alexander, 2009. Incorporating uncertainty into the ranking of SPARROW model nutrient yields from Mississippi/Atchafalaya River basin watersheds. Jouranl of the American Water Resources Association 45(2):534–549.

Ruddy, B.C., D.L. Lorenz, and D.K. Mueller, 2006. County-Level Estimates of Nutrient Inputs to the Land Surface of the Conterminous United States, 1982–2001. U.S. Geological Survey Scientific Investigations Report 2006-5012, Reston, VA, 17 p.

Saad, D.A., G.E. Schwarz, D.M. Robertson, and N.L. Booth, this issue. A Multi-Agency Nutrient Dataset Used to Estimate Loads, Improve Monitoring Design, and Calibrate Regional Nutrient SPARROW Models. Journal of the American Water Resources Association, doi: 10.1111/j.1752-1688.2011.00575.x.

Schwarz, G.E., A.B. Hoos, R.B. Alexander, and R.A. Smith, 2006. The SPARROW Surface Water-Quality Model: Theory, Application and User Documentation, U.S. Geological Survey Techniques and Methods Report, Book 6, Chapter B3, Reston, VA, 2006.

Seaber, P.R., F.P. Kapinos, and G.L. Knapp, 1987. Hydrologic Unit maps: U.S. Geol. Surv. Water-Supply Paper 2294, 63 p. Available online at: http://water.usgs.gov/GIS/huc.html, accessed on June 14, 2011.

Shao, J. and D. Tu, 1995, The jackknife and bootstrap, New York, Springer, 516 p.

Smith, R.A., G.E. Schwarz, and R.B. Alexander, 1997. Regional interpretation of water-quality monitoring data. Water Resources Research 33:2781–2798.

U.S. Army Corps of Engineers (USACE), 2007. National Invetory of Dams (NID): U.S. Army Engineer Research and Development Center, Available at: http://www.agc.army.mil/fact_sheet/nid.pdf, accessed on August 25, 2009.

U.S. Department of Agriculture (USDA), 2010, NRCS Conservation Programs: Mississippi River Basin Healthy Watersheds Initiative, Available at: http://www.nrcs.usda.gov/programs/, accessed on July 14, 2010.

U.S. Geological Survey (USGS), 2000, National Land Cover Dataset: U.S. Geological Survey Fact Sheet 1008–00, Reston, VA, 1 p.

Wieczorek, M.E and A.E. Lamotte, 2011. SPARROW model variables for MRB_E2RF1 catchments, Attributes for MRB-E2RF1 Catchments by major river basins in the conterminous United States: (DS-491-XX), http://water.usgs.gov/nawqa/modeling/rf1attributes.html, accessed June 14, 2011.

Wolock, D.M., 1997. STATSGO Soil Characteristics for the Conterminous United States: U.S. Geological Survey Open-File Report-656, Reston, VA.

Zucker, L.A. and L.C. Brown (Editors). 1998. Agricultural Drainage: Water Quality Impacts and Subsurface Drainage Studies in the Midwest. Ohio State University Extension Bulletin 871. The Ohio State University, Columbus, OH.

Table S2_TP. Summary of measured (with Fluxmaster) and estimated (with SPARROW) loads for each monitoring site used in the calibration of the SPARROW models, with a summary of the measured loads. [Not all water-water quality used in Fluxmaster calculations if outside of year range specified in Table S1.]

Supplemental Material to Robertson and Saad, 2011, Journal of the American Water Resources Association, Nutrient Inputs to the Laurentian Great Lakes By Source and River Basin Estimated Using SPARROW Watershed Models.

[kg, kilogram; km^2, square kilometer; mg/L, milligrams per liter; kg/yr; kilograms per year]
HUC8's are based on Seaber, P.R., F.P. Kapinos, and G.L. Knapp, 1987. Hydrologic Unit maps: U.S. Geol. Surv. Water-Supply Paper 2294, 63 p. Available online at: http://water.usgs.gov/GIS/huc.html

Identification and Location Information							Total Phosphorus Concentration and Load Data							
Station Identification Number	Name	HUC8	State	Latitude (decimal)	Longitude (decimal)	Area (km^2)	Water Quality Start Date	Water Quality End Date	Number of Observations	Midmonthly Mean (mg/L)	Midmonthly Median (mg/L)	Fluxmaster Load (kg/yr)	SPARROW Estimated Load (kg/yr)	Standard Deviation in Fluxmaster Loads (kg/yr)
000755	CUMBERLAND R 262.9 @ HWY 231 QUAD 313SE	5130201	TN	36.29917	-86.2631	28,282.3	11/7/1977	12/7/1998	116	0.101	0.060	1,798,719	1,146,849	359,408
000770	CUMBERLAND R 174.5 U/S FROM CLEESE'S FERRY	5130202	TN	36.14842	-86.8902	33,040.0	8/8/1972	10/22/1998	163	0.162	0.130	1,119,119	1,870,025	227,146
002205	RED RIVER 8.4 QUAD 301SE	5130206	TN	36.52833	-87.2783	2,352.1	7/29/1982	7/8/1998	36	0.139	0.100	205,092	260,758	57,636
010	MISSISSIPPI R BR ON CR-441 1 MI SW OF BLACKBERRY	7010103	MN	47.17447	-93.4206	9,310.1	5/16/1974	9/27/1994	149	0.071	0.048	79,610	51,386	6,046
018	KNIFE RIVER UPSTREAM OF US-61 AT KNIFE RIVER	4010102	MN	46.94698	-91.7951	225.8	7/10/1974	9/22/1998	74	0.046	0.030	7,395	4,245	1,807
020051	DRUMMOND BOG DISCHARGE 1MI NE DRUMMOND,WI	4010302	WI	46.34888	-91.2584	88.9								
03011800	Kinzua Creek near Guffey, PA	5010001	PA	41.76673	-78.7187	100.5	3/7/1973	8/16/1995	162	0.039	0.020	2,560	1,270	361
03012600	Allegheny River at Warren, PA	5010001	PA	41.8245	-79.1189	5,757.6	1/19/1988	9/7/2005	150	0.027	0.030	70,656	129,379	7,693
03015000	Conewango Creek at Russell, PA	5010002	PA	41.93811	-79.1331	2,113.4	12/23/1970	9/7/2005	315	0.104	0.080	74,784	78,340	5,003
03015500	Brokenstraw Creek at Youngsville, PA	5010001	PA	41.85447	-79.3164	831.4	12/23/1970	9/7/2005	220	0.057	0.030	17,367	26,866	1,873
03016000	Allegheny River at West Hickory, PA	5010003	PA	41.56971	-79.4056	9,479.4	3/5/1972	9/6/2005	279	0.041	0.037	209,150	254,947	16,793
03020500	Oil Creek at Rouseville, PA	5010003	PA	41.48096	-79.6953	777.0	12/21/1970	9/21/2005	173	0.066	0.040	14,839	28,597	1,683
03024000	French Creek at Utica, PA	5010004	PA	41.43792	-79.9554	2,662.5	12/21/1970	11/20/2002	102	0.084	0.050	48,650	92,062	8,633
03029500	Clarion River at Cooksburg, PA	5010005	PA	41.3314	-79.209	2,090.1	12/24/1970	9/6/2005	217	0.036	0.030	40,218	50,219	4,789
03031500	Allegheny River at Parker, PA	5010006	PA	41.10086	-79.6794	19,867.9	7/7/1971	1/26/2005	169	0.082	0.040	393,586	522,907	64,528
03032500	Redbank Creek at St. Charles, PA	5010006	PA	40.99482	-79.3952	1,367.5	9/13/1971	9/22/2005	303	0.041	0.030	25,954	43,301	3,250
03040000	Stonycreek River at Ferndale, PA	5010007	PA	40.28592	-78.9203	1,168.1	10/21/1975	9/28/1998	43	0.033	0.026	26,297	45,713	7,750
03042000	Blacklick Creek at Josephine, PA	5010007	PA	40.47345	-79.1834	497.3	8/2/1972	6/6/1995	211	0.077	0.040	23,836	9,312	2,619
03044000	Conemaugh River at Tunnelton, PA	5010007	PA	40.45451	-79.3909	3,517.2	6/28/1971	9/28/2005	199	0.045	0.020	37,532	115,027	6,239
03049625	Allegheny River at New Kensington, PA	5010009	PA	40.56451	-79.7726	29,904.1	5/21/1973	8/10/2004	209	0.044	0.027	620,789	833,233	92,048
03050001	TYGART VALLEY RIVER NEAR DAILEY, WV	5020001	WV	38.80901	-79.8822	479.2	6/18/1974	3/12/1996	92	0.042	0.026	6,346	11,094	641
03061000	WEST FORK RIVER AT ENTERPRISE, WV	5020002	WV	39.42209	-80.2759	1,965.8	7/16/1974	2/15/2005	284	0.100	0.068	100,165	73,890	15,605
03063000	Monongahela R at Lock & Dam 8, at Pnt Marion, PA	5020003	PA	39.72702	-79.9115	7,044.8	5/23/1973	9/26/2005	31	0.257	0.029	273,701	233,510	91,692
03072000	Dunkard Creek at Shannopin, PA	5020005	PA	39.75916	-79.9703	593.1	7/17/1973	9/26/2005	322	0.049	0.030	7,548	15,441	944
03077500	Youghiogheny River at Youghiogheny River Dam, PA	5020006	PA	39.80567	-79.3647	1,129.2	8/16/1972	9/27/2005	184	0.038	0.020	11,704	21,961	1,462
03079000	Casselman River at Markleton, PA	5020006	PA	39.85987	-79.2283	989.4	8/16/1972	8/17/1995	152	0.085	0.040	63,969	40,318	9,210
03083250	Sewickley Creek at Hunker, PA	5020006	PA	40.20642	-79.623	230.0	1/5/1971	6/5/1995	235	0.347	0.160	34,619	15,812	3,117
03083500	Youghiogheny River at Sutersville, PA	5020006	PA	40.24003	-79.8063	4,441.9	12/28/1972	9/14/2005	330	0.106	0.040	158,652	143,534	23,834
03085000	Monongahela River at Braddock, PA	5020005	PA	40.39043	-79.8586	19,002.8	2/13/1973	8/10/2004	232	0.068	0.050	472,769	597,878	60,218
03086000	Ohio River at Sewickley, PA	5030101	PA	40.55023	-80.204	50,505.0	5/23/1973	9/1/2005	84	0.089	0.045	2,312,488	1,776,312	172,122
03099500	Mahoning River at Lowellville OH	5030103	OH	41.03598	-80.5368	2,779.1	10/26/1987	9/22/2000	141	0.490	0.345	160,934	227,271	19,679
03099600	Mahoning River at North Edinburg, PA	5030103	PA	41.01839	-80.4406	2,846.4	10/20/1987	9/13/2005	160	0.356	0.310	175,038	231,119	18,735
03103500	Shenango River at Sharpsville, PA	5030102	PA	41.26617	-80.4726	1,512.6	10/8/1970	9/19/2005	319	0.085	0.070	44,020	43,470	3,248
03105500	Beaver River at Wampum, PA	5030104	PA	40.88894	-80.3366	5,788.7	12/23/1970	9/15/2003	333	0.266	0.240	287,380	365,864	32,532
03106000	Connoquenessing Creek near Zelienople, PA	5030105	PA	40.81673	-80.2426	922.0	10/5/1971	9/1/2005	295	0.137	0.100	21,434	37,346	2,202
03106500	Slippery Rock Creek at Wurtemburg, PA	5030105	PA	40.88412	-80.2338	1,030.8	3/6/1973	9/1/2005	316	0.059	0.040	26,261	32,206	2,482
03107500	Beaver River at Beaver Falls, PA	5030104	PA	40.76298	-80.3159	8,044.5	10/5/1971	7/5/2006	665	0.199	0.160	426,095	586,020	21,159
03108000	Raccoon Creek at Moffatts Mill, PA	5030101	PA	40.62784	-80.3376	461.0	10/5/1971	9/1/2005	294	0.106	0.070	7,795	15,540	853
03109500	Little Beaver Creek near East Liverpool OH	5030101	OH	40.67576	-80.5404	1,284.6	8/19/1971	12/22/1998	272	0.122	0.070	71,625	68,963	12,793
03146500	Licking River near Newark OH	5040006	OH	40.05923	-82.3383	1,390.8	8/21/1974	1/17/1997	280	0.351	0.210	110,954	106,482	7,713
03150000	Muskingum River at McConnelsville OH	5040004	OH	39.64409	-81.8506	19,223.0	11/27/1970	12/5/2004	1343	0.138	0.116	1,342,000	1,429,045	17,529
03155000	LITTLE KANAWHA RIVER AT PALESTINE, WV	5030203	WV	39.05893	-81.3893	3,926.4	1/29/1974	5/9/1995	239	0.050	0.033	115,303	136,002	11,106
03157000	Clear Creek near Rockbridge OH	5030204	OH	39.58835	-82.5785	230.5	10/12/1971	9/20/1995	46	0.056	0.050			
03164000	NEW RIVER NEAR GALAX, VA	5050001	VA	36.64646	-80.9777	2,929.3	7/11/1979	12/17/1998	200	0.090	0.100	146,779	86,084	18,221
03167000	REED CREEK AT GRAHAMS FORGE, VA	5050001	VA	36.93946	-80.887	639.7	1/23/1979	11/17/1998	190	0.091	0.100	14,662	34,060	1,255
03170000	LITTLE RIVER AT GRAYSONTOWN, VA	5050001	VA	37.03733	-80.5567	777.0	7/25/1979	9/4/1998	129	0.097	0.100	40,212	30,422	16,499

03171500	NEW RIVER AT EGGLESTON, VA	5050002	VA	37.29008	-80.6161	7,617.2	9/11/1973	12/8/1998	121	0.097	0.100	440,346	288,534	51,153
03176500	NEW RIVER AT GLEN LYN, VA	5050002	VA	37.37221	-80.8613	9,759.1	1/17/1979	12/8/1998	320	0.090	0.100	273,860	338,975	15,510
03184000	GREENBRIER RIVER AT HILLDALE, WV	5050003	WV	37.64012	-80.8047	4,193.2	12/4/1973	5/4/1995	242	0.037	0.024	89,237	99,595	9,325
03189100	GAULEY RIVER NEAR CRAIGSVILLE, WV	5050005	WV	38.29094	-80.6409	1,370.1	6/25/1974	5/28/1998	69	0.028	0.010			
03189600	GAULEY RIVER BELOW SUMMERSVILLE, WV	5050005	WV	38.21511	-80.8882	2,087.5	6/25/1974	5/28/1998	62	0.015	0.010			
03193000	KANAWHA RIVER AT KANAWHA FALLS, WV	5050006	WV	38.13774	-81.2135	21,680.9	12/17/1973	9/3/1998	38	0.094	0.050			
03194700	ELK RIVER BELOW WEBSTER SPRINGS, WV	5050007	WV	38.59741	-80.4905	688.9	9/11/1973	5/27/1998	67	0.021	0.010			
03198350	CLEAR FORK AT WHITESVILLE, WV	5050009	WV	37.96624	-81.5241	162.7	10/22/1996	3/8/2000	49	0.035	0.050			
03201300	KANAWHA RIVER AT WINFIELD, WV	5050008	WV	38.52657	-81.9116	30,585.3	2/15/1974	7/12/2006	645	0.096	0.067	903,608	831,157	68,710
03202000	Raccoon Creek at Adamsville OH	5090101	OH	38.85896	-82.3618	1,515.2	9/16/1974	9/1/1998	60	0.085	0.050	30,379	53,948	7,074
03209300	RUSSELL FORK AT ELKHORN CITY, KY	5070202	KY	37.30401	-82.3428	1,434.9	8/25/1971	12/12/1995	42	0.036	0.020			
03209500	LEVISA FORK AT PIKEVILLE, KY	5070203	KY	37.46425	-82.5263	3,190.9	3/27/1979	11/16/2006	257	0.031	0.018	24,522	46,610	5,268
03215000	BIG SANDY R AT LOUISA, KY	5070204	WV	38.1708	-82.6347	10,093.2	8/31/1971	7/11/2006	453	0.112	0.069	348,496	153,810	47,840
03216600	OHIO RIVER AT GREENUP DAM NEAR GREENUP, KY	5090103	KY	38.64684	-82.8588	160,580.0	10/22/1974	7/25/2006	165	0.089	0.066	10,790,848	6,378,772	989,683
03219500	Scioto River near Prospect OH	5060001	OH	40.4195	-83.1971	1,468.5	8/31/1971	10/2/1995	135	0.450	0.290	160,831	131,569	18,992
03225500	Olentangy River near Delaware OH	5060001	OH	40.35499	-83.0678	1,017.9	12/15/1970	3/28/1995	84	0.105	0.081	48,195	84,221	11,828
03229000	Alum Creek at Columbus OH	5060001	OH	39.94484	-82.9413	489.5	10/23/1970	9/19/1996	64	0.155	0.080	13,394	39,555	3,367
03229500	Big Walnut Creek at Rees OH	5060001	OH	39.85673	-82.9571	1,409.0	10/28/1970	9/3/1991	37	0.231	0.210	85,233	87,675	28,755
03230450	Hellbranch Run near Harrisburg OH	5060001	OH	39.83063	-83.1598	95.8	1/4/1993	9/27/2005	172	0.131	0.063	20,211	6,844	2,910
03230500	Big Darby Creek at Darbyville OH	5060001	OH	39.70097	-83.1096	1,383.1	10/30/1970	12/12/1996	294	0.184	0.120	97,407	93,444	8,709
03230800	Deer Creek at Mount Sterling OH	5060002	OH	39.71514	-83.2574	590.5	10/29/1970	12/16/1996	71	0.266	0.076	69,306	33,190	25,433
03231500	Scioto River at Chillicothe OH	5060002	OH	39.34217	-82.9713	9,968.9	6/8/1973	12/6/2004	3116	0.457	0.370	1,097,308	979,249	13,820
03232000	Paint Creek near Greenfield OH	5060003	OH	39.37929	-83.3754	644.9	12/10/1970	1/10/1997	52	0.468	0.250	40,525	40,306	9,633
03234500	Scioto River at Higby OH	5060002	OH	39.21192	-82.8644	13,289.3	6/8/1973	12/14/1998	470	0.391	0.330	893,053	1,181,137	70,431
03237280	Upper Twin Creek at McGaw OH	5090201	OH	38.64375	-83.2155	31.6	10/22/1970	8/29/2001	264	0.023	0.010	70	582	19
03240000	Little Miami River near Oldtown OH	5090202	OH	39.74792	-83.9314	334.1	9/17/1971	12/17/1998	282	0.165	0.100	10,066	15,342	1,539
03242200	Anderson Fork near New Burlington OH	5090202	OH	39.56638	-83.9028	201.5	10/20/1970	9/1/1998	36	0.126	0.060	15,999	8,680	7,648
03245500	Little Miami River at Milford OH	5090202	OH	39.17138	-84.2985	3,115.8	10/28/1970	9/13/2000	245	0.466	0.380	362,831	330,538	33,842
03248100	Little Miami River at Cincinnati OH	5090202	OH	39.10921	-84.4016	4,517.0	10/1/1973	7/26/2006	266	0.401	0.310	297,246	476,722	32,374
03265000	Stillwater River at Pleasant Hill OH	5080001	OH	40.05779	-84.356	1,302.8	8/12/1971	12/17/1998	67	0.208	0.160	84,106	193,796	18,860
03267900	Mad River at St Paris Pike at Eagle City OH	5080001	OH	39.96415	-83.8314	802.9	10/15/1970	10/13/2004	331	0.120	0.060	34,947	45,576	3,727
03270000	Mad River near Dayton OH	5080001	OH	39.79702	-84.0916	1,644.7	10/7/1970	12/27/1990	180	0.341	0.300	196,442	119,566	8,562
03271601	Great Miami River below Miamisburg OH	5080002	OH	39.60687	-84.2865	7,031.9	4/22/1996	12/6/2004	2785	0.371	0.322	925,079	759,240	12,873
03274000	Great Miami River at Hamilton OH	5080002	OH	39.39116	-84.5718	9,401.7	9/21/1973	9/18/2001	153	0.513	0.404	1,069,294	1,010,896	117,671
03274600	Great Miami River at New Baltimore OH	5080002	OH	39.26318	-84.6677	9,878.3	12/8/1972	9/20/1995	209	0.473	0.420	1,564,936	1,070,607	76,449
03275000	WHITEWATER RIVER NEAR ALPINE, IN	5080003	IN	39.57944	-85.1574	1,352.0	2/18/1987	9/13/2000	70	0.091	0.070	93,332	79,826	27,765
03275600	EAST FORK WHITEWATER RIVER AT ABINGTON, IND.	5080003	IN	39.73251	-84.9596	518.0	3/11/1971	2/14/2006	249	0.284	0.120	19,267	49,665	2,059
03277200	OHIO RIVER AT MARKLAND DAM NEAR WARSAW, KY	5090203	KY	38.77632	-84.9642	215,410.3	10/30/1974	7/13/2006	367	0.152	0.100	12,174,237	11,205,103	1,165,267
03281000	MIDDLE FORK KENTUCKY RIVER AT TALLEGA, KY	5100202	KY	37.55533	-83.5938	1,390.8	9/7/1971	7/19/2006	276	0.031	0.016	19,785	24,329	3,259
03281500	SOUTH FORK KENTUCKY RIVER AT BOONEVILLE, KY	5100203	KY	37.47928	-83.6751	1,870.0	10/20/1971	7/19/2006	271	0.027	0.013	28,338	38,519	5,977
03285000	DIX RIVER NEAR DANVILLE, KY	5100205	KY	37.64208	-84.6609	823.6	10/7/1970	10/3/2006	200	0.085	0.063	40,576	106,690	5,926
03289500	ELKHORN CREEK NEAR FRANKFORT, KY	5100205	KY	38.26859	-84.8146	1,225.1	10/2/1970	11/17/2006	111	0.647	0.510	278,699	218,700	36,723
03290500	KENTUCKY RIVER AT LOCK 2 AT LOCKPORT, KY	5100205	KY	38.43867	-84.9625	16,006.2	2/7/1973	6/6/1995	217	0.182	0.150	1,594,137	1,079,742	93,070
03291500	EAGLE CREEK AT GLENCOE, KY	5100205	KY	38.70589	-84.8252	1,131.8	10/6/1970	11/17/2006	201	0.132	0.079	117,777	121,864	15,956
03294600	OHIO RIVER AT KOSMOSDALE, KY	5140101	KY	38.03519	-85.9175	236,208.0	10/12/1976	7/26/2006	315	0.264	0.180	26,182,650	12,917,495	2,779,600
03298500	SALT RIVER AT SHEPHERDSVILLE, KY	5140102	KY	37.98519	-85.7174	3,100.2	10/5/1970	12/19/2006	374	0.277	0.216	495,989	355,897	36,124
03301630	ROLLING FORK NEAR LEBANON JUNCTION, KY	5140103	KY	37.82258	-85.7477	3,561.3	10/8/1974	12/19/2006	284	0.211	0.154	434,228	406,004	39,914
03303280	OHIO RIVER AT CANNELTON DAM AT CANNELTON, IN	5140201	KY	37.89848	-86.7042	251,230.0	10/1/1975	7/27/2006	483	0.159	0.100	22,258,297	14,074,204	1,348,503
03308500	GREEN RIVER AT MUNFORDVILLE, KY	5110001	KY	37.26779	-85.8857	4,333.1	8/25/1971	12/14/2006	312	0.064	0.042	109,649	332,872	10,483
03314500	BARREN RIVER AT BOWLING GREEN, KY	5110002	KY	37.0014	-86.4305	4,788.9	10/26/1970	9/12/1994	177	0.044	0.031	117,856	489,532	9,870
03322500	WABASH RIVER NEAR NEW CORYDON, IND	5120101	IN	40.56323	-84.8028	678.6	12/22/1970	2/13/2006	42	0.568	0.415	165,220	142,824	27,577
03325500	MISSISSINEWA RIVER NEAR RIDGEVILLE, IND.	5120103	IN	40.28015	-84.9958	344.5	6/5/1979	2/13/2006	300	0.263	0.170	36,470	42,988	3,932
03328500	EEL RIVER NEAR LOGANSPORT, IN	5120104	IN	40.78212	-86.2646	2,043.5	1/22/1976	2/22/2006	343	0.139	0.100	174,205	116,704	12,665
03329700	DEER CREEK NEAR DELPHI, IND.	5120105	IN	40.59032	-86.6217	709.7	3/25/1998	2/22/2006	92	0.086	0.060	55,162	61,818	12,408
03335500	WABASH RIVER AT LAFAYETTE IND	5120108	IN	40.42193	-86.8963	18,821.5	2/26/1971	12/6/1995	244	0.224	0.140	1,741,601	1,380,426	127,904
03336645	MIDDLE FORK VERMILION RIVER ABOVE OAKWOOD, IL	5120109	IL	40.13627	-87.7462	1,118.9	10/19/1978	12/8/1998	150	0.100	0.050	110,859	111,802	19,015
03338780	NORTH FORK VERMILION RIVER NEAR BISMARCK, IL	5120109	IL	40.26517	-87.6438	678.6	11/10/1988	12/30/1998	90	0.145	0.100	52,819	43,559	8,734
03339000	VERMILION RIVER NEAR DANVILLE, IL	5120109	IL	40.0874	-87.597	3,341.1	4/11/1978	12/10/1998	167	0.498	0.310	314,356	288,176	20,327
03341910	WABASH RIVER AT HUTSONVILLE, IL	5120111	IN	39.11028	-87.655	33,715.1	7/20/1972	12/15/1998	146	0.229	0.200	4,084,683	2,400,253	382,342
03341920	TURMAN CREEK NR FARMERSBURG, IN	5120111	IN	39.24448	-87.4078	33.7	7/20/1972	4/14/1997	125	0.232	0.200	4,319	2,137	431

03343395	EMBARRAS RIVER AT CAMARGO, IL	5120112	IL	39.79916	-88.1701	466.2	3/14/1984	12/21/1998	129	0.120	0.090	13,970	25,924	1,465
03345500	EMBARRAS RIVER AT STE. MARIE, IL	5120112	IL	38.93536	-88.0146	3,926.4	2/29/1972	12/16/1998	192	0.270	0.190	350,466	355,911	36,575
03346000	NORTH FORK EMBARRAS RIVER NEAR OBLONG, IL	5120112	IL	39.00893	-87.9461	823.6	10/5/1971	12/16/1998	190	0.226	0.160	72,891	97,894	8,986
03351500	FALL CREEK NEAR FORTVILLE, IND.	5120201	IN	39.95456	-85.8676	437.7	10/26/1972	2/15/2006	101	0.083	0.070	19,290	19,549	2,006
03354000	WHITE RIVER NEAR CENTERTON, IN	5120201	IN	39.49722	-86.4009	6,330.0	3/17/1971	8/17/1995	246	0.907	0.610	1,383,891	771,725	76,525
03357500	BIG WALNUT CREEK NEAR REELSVILLE, IN	5120203	IN	39.53572	-86.9763	844.3	2/27/1996	11/13/2003	61	0.096	0.080	36,259	56,109	5,290
03358000	MILL CREEK NEAR CATARACT, IND.	5120203	IN	39.43349	-86.7634	634.6	7/26/1971	2/14/2006	355	0.108	0.070	37,119	42,881	4,273
03362500	SUGAR CREEK NEAR EDINBURGH, IN	5120204	IN	39.36097	-85.9982	1,227.7	2/6/1986	2/9/2006	134	0.124	0.110	77,366	90,227	7,147
03366500	MUSCATATUCK RIVER NEAR DEPUTY, IN	5120207	IN	38.80426	-85.6738	758.9	4/5/1993	4/18/1995	28	0.103	0.070	61,599	58,543	16,598
03374100	WHITE RIVER AT HAZLETON, IN	5120202	IN	38.48977	-87.55	29,280.0	2/21/1973	2/13/2006	524	0.207	0.180	2,886,170	2,305,666	119,242
03378000	BONPAS CREEK AT BROWNS, IL	5120113	IL	38.38246	-87.9769	590.5	2/16/1984	12/16/1998	124	0.248	0.190	85,778	79,928	7,789
03378500	WABASH RIVER AT NEW HARMONY, IND.	5120113	IN	38.1314	-87.9429	75,716.1	11/7/1974	7/13/2006	333	0.223	0.200	7,717,761	5,946,186	375,691
03378635	LITTLE WABASH RIVER NEAR EFFINGHAM, IL	5120114	IL	39.10344	-88.5926	621.6	5/29/1979	12/21/1998	129	0.214	0.170	48,101	69,629	5,208
03378900	LITTLE WABASH RIVER AT LOUISVILLE, IL	5120114	IL	38.77291	-88.497	1,929.6	7/11/1973	12/8/1998	171	0.289	0.210	415,709	300,266	46,759
03379500	LITTLE WABASH RIVER BELOW CLAY CITY, IL	5120114	IL	38.63345	-88.2953	2,929.3	3/15/1984	12/8/1998	134	0.296	0.230	515,986	479,414	42,249
03379600	LITTLE WABASH RIVER AT BLOOD, IL	5120114	IL	38.51894	-88.1319	3,577.7	10/13/1977	12/7/1998	135	0.293	0.250	580,606	605,053	39,669
03380500	SKILLET FORK AT WAYNE CITY, IL	5120115	IL	38.35867	-88.5848	1,201.8	2/16/1984	12/7/1998	132	0.154	0.120	130,005	103,315	11,688
03381495	LITTLE WABASH RIVER AT MAIN ST AT CARMI, IL	5120114	IL	38.09232	-88.1561	7,997.9	10/29/1979	12/16/1998	180	0.255	0.220	1,059,437	1,065,232	66,809
03382090	SUGAR CREEK NEAR STONEFORT, IL	5140204	IL	37.65533	-88.7633	90.7	10/25/1977	11/19/1998	121	0.625	0.140	4,054	5,111	735
03382100	SOUTH FORK SALINE RIVER NR CARRIER MILLS, IL	5140204	IL	37.63643	-88.6772	380.7	12/14/1977	12/10/1998	194	0.061	0.010	68,528	17,632	18,391
03382185	BANKSTON FORK NEAR DORRIS HEIGHTS, IL	5140204	IL	37.76754	-88.5404	201.2	2/2/1984	11/23/1998	135	0.053	0.020	13,057	10,894	2,849
03384450	LUSK CREEK NEAR EDDYVILLE, IL	5140203	IL	37.47253	-88.5476	111.1	10/31/1977	12/23/1998	174	0.020	0.010	1,441	3,551	346
03404500	CUMBERLAND RIVER AT CUMBERLAND FALLS, KY	5130101	KY	36.83694	-84.3437	5,120.4	10/7/1970	12/11/2006	277	0.038	0.024	118,417	120,274	22,939
03428500	WEST FORK STONES RIVER NEAR SMYRNA, TN	5130203	TN	35.94027	-86.4649	613.8	9/29/1982	12/7/1998	31	0.541	0.210	70,317	85,234	11,204
03434500	HARPETH RIVER NEAR KINGSTON SPRINGS, TN	5130204	TN	36.122	-87.0989	1,763.8	6/25/1975	10/22/1998	51	0.383	0.310	412,657	99,479	93,143
03612000	CACHE RIVER AT FORMAN, IL	5140206	IL	37.3352	-88.9258	632.0	10/26/1977	11/16/1998	134	0.174	0.140	71,540	35,136	4,677
04024000	ST. LOUIS RIVER AT SCANLON, MN	4010201	MN	46.70322	-92.4178	8,883.7	11/17/1970	11/20/1996	347	0.050	0.040	114,580	110,771	5,697
04024430	NEMADJI RIVER NEAR SOUTH SUPERIOR, WI	4010301	WI	46.63326	-92.0943	1,087.8	1/28/1974	3/19/1998	289	0.093	0.040	66,454	17,229	4,315
04027595	BAD RIVER AT ODANAH, WI	4010302	WI	46.61028	-90.6869	2,512.3	11/30/1970	2/7/2005	241	0.050	0.040	74,004	55,058	9,740
04040000	ONTONAGON RIVER NEAR ROCKLAND, MI	4020102	MI	46.7207	-89.2075	3,470.6	10/3/1974	8/29/1995	142	0.052	0.040	81,788	42,456	7,368
04057005	MANISTIQUE RIVER AT MANISTIQUE, MI	4060106	MI	45.95164	-86.2485	3,755.5	10/20/1970	10/26/1995	243	0.029	0.027	41,285	31,333	2,638
04063700	POPPLE RIVER NEAR FENCE, WI	4030108	WI	45.76347	-88.4632	360.0	10/20/1970	9/20/2007	336	0.026	0.020	2,265	3,668	203
04067500	MENOMINEE RIVER NEAR MC ALLISTER, WI	4030108	MI	45.32572	-87.6621	10,178.7	10/24/1979	8/8/2007	158	0.027	0.024	73,721	146,494	7,513
04071000	OCONTO RIVER NEAR GILLETT, WI	4030104	WI	44.86465	-88.3001	1,826.0	1/29/1973	10/13/2003	207	0.037	0.027	11,008	24,131	1,778
04072050	DUCK CREEK AT SEMINARY ROAD NEAR ONEIDA, WI	4030103	WI	44.46521	-88.2187	247.3	4/7/1993	10/20/2004	108	0.216	0.189	9,059	25,511	863
04072150	DUCK CREEK NEAR HOWARD, WI	4030103	WI	44.53371	-88.1291	279.7	9/21/1988	9/30/2005	358	0.199	0.170	17,295	25,698	1,293
040734644	SILVER CREEK AT SOUTH KORO ROAD NEAR RIPON, WI	4030201	WI	43.8581	-88.87	93.8	3/18/1987	10/1/1996	624	0.335	0.240	8,516	8,589	325
04073468	GREEN LAKE INLET AT CT HIGHWAY A NR GREEN LAKE, WI	4030201	WI	43.82369	-88.9269	138.6	3/5/1987	9/20/2005	1038	0.150	0.130	5,557	10,947	177
04073470	PUCHYAN RIVER AT GREEN LAKE, WI	4030201	WI	43.84665	-88.9601	266.8	2/19/1997	9/20/2005	122	0.034	0.031	2,208	3,806	151
04075050	WOLF RIVER AT HIGHWAY M NEAR LANGLADE, WI	4030202	WI	45.12632	-88.6635	1,266.5	1/3/1990	8/28/2001	63	0.027	0.020	4,491	13,954	1,542
04077100	WOLF RIVER AT KESHENA, WI	4030202	WI	44.88332	-88.6348	2,297.8	5/31/1995	8/27/2001	27	0.025	0.020			
04077630	RED RIVER AT MORGAN ROAD NEAR MORGAN, WI	4030202	WI	44.89814	-88.8441	295.3	11/4/1992	7/2/1998	171	0.028	0.020	3,076	5,129	520
04080798	TOMORROW RIVER NEAR NELSONVILLE, WI	4030202	WI	44.52432	-89.3381	114.0	4/9/1993	10/17/2002	38	0.032	0.020			
04085139	FOX RIVER AT MOUTH AT GREEN BAY, WI	4030103	WI	44.53949	-88.0049	16,394.7	10/5/1988	9/15/2005	153	0.131	0.130	634,948	462,782	58,838
04085427	MANITOWOC RIVER AT MANITOWOC, WI	4030101	WI	44.10708	-87.7151	1,362.3	1/24/1977	9/20/2007	227	0.215	0.190	50,813	109,088	3,572
04086500	CEDAR CREEK NEAR CEDARBURG, WI	4040003	WI	43.32308	-87.9786	310.8	1/25/1977	11/8/2004	108	0.220	0.170	5,302	16,002	732
04086600	MILWAUKEE RIVER NEAR CEDARBURG, WI	4040003	WI	43.27999	-87.9421	1,572.1	9/28/1994	9/23/2005	75	0.126	0.108	50,493	73,940	7,971
04087000	MILWAUKEE RIVER AT MILWAUKEE, WI	4040003	WI	43.10001	-87.909	1,802.6	1/25/1973	9/26/2007	635	0.136	0.120	52,703	87,147	1,738
04087170	MILWAUKEE RIVER AT MOUTH AT MILWAUKEE, WI	4060200	WI	43.02505	-87.8985	2,258.5	3/29/1994	8/29/2005	47	0.061	0.058	47,267	186,977	3,726
04101500	ST. JOSEPH RIVER AT NILES, MI	4050001	MI	41.82928	-86.2596	9,494.9	2/12/1979	9/22/1995	103	0.074	0.060	242,948	432,433	16,940
04102533	ST. JOSEPH RIVER AT ST. JOSEPH, MI	4050001	MI	42.11318	-86.4853	12,095.3	10/22/1970	10/27/1995	278	0.094	0.080	385,328	532,721	12,703
04119300	GRAND RIVER AT EASTMANVILLE, MI	4050006	MI	43.01511	-85.9559	13,545.7	2/14/1979	10/7/1994	98	0.107	0.100	438,156	623,305	34,114
04122500	PERE MARQUETTE RIVER AT SCOTTVILLE, MI	4060101	MI	43.94469	-86.2792	1,763.8	12/15/1970	10/18/1995	63	0.038	0.030	27,369	18,661	2,931
04137500	AU SABLE RIVER NEAR AU SABLE, MI	4070007	MI	44.43622	-83.4344	4,504.0	6/6/1978	9/28/1998	129	0.015	0.010	14,514	40,916	2,383
04156100	TITTABAWASSEE RIVER NR MIDLAND, MI	4080201	MI	43.56864	-84.1936	6,345.5	7/24/1980	9/23/1996	220	0.074	0.066	134,708	120,179	5,927
04157000	SAGINAW RIVER AT SAGINAW, MI	4080206	MI	43.41322	-83.9642	15,695.4	3/12/1976	7/21/2005	66	0.152	0.126	278,735	424,307	33,411
04161820	CLINTON RIVER AT STERLING HEIGHTS, MI	4090003	MI	42.61452	-83.0262	800.3	4/4/1996	8/30/2005	81	0.068	0.059	16,223	31,941	1,942
04165500	CLINTON RIVER AT MORAVIAN DRIVE AT MT. CLEMENS, MI	4090003	MI	42.59572	-82.9093	1,901.1	10/8/1974	8/24/1995	139	0.207	0.170	98,659	151,282	6,201
04170000	HURON RIVER AT MILFORD, MI	4090005	MI	42.57892	-83.6276	341.9	4/6/1984	9/8/2003	46	0.049	0.043	1,578	15,956	463
04170500	HURON RIVER NEAR NEW HUDSON, MI	4090005	MI	42.51253	-83.6763	383.3	4/6/1984	9/8/2003	44	0.042	0.040	885	14,356	386

04175600	RIVER RAISIN NEAR MANCHESTER, MI	4100002	MI	42.16796	-84.0765	341.9	4/2/1996	8/26/2004	61	0.020	0.018	1,930	6,054	367
04176500	RIVER RAISIN NEAR MONROE, MI	4100002	MI	41.96033	-83.5311	2,698.8	1/19/1978	9/30/2004	6712	0.135	0.114	110,680	96,475	2,169
04178000	ST. JOSEPH RIVER NEAR NEWVILLE, IN	4100003	IN	41.38571	-84.8019	1,579.9	3/7/1996	10/25/2004	89	0.155	0.128	110,498	64,322	12,415
04186500	Auglaize River near Fort Jennings OH	4100007	OH	40.94899	-84.2665	859.9	8/27/1974	8/9/2005	91	0.219	0.162	66,552	79,806	7,621
04189000	Blanchard River near Findlay OH	4100008	OH	41.05609	-83.6879	896.1	5/14/1973	9/19/1996	135	1.337	0.670	153,988	55,908	25,789
04193500	Maumee River at Waterville OH	4100009	OH	41.50016	-83.7139	16,394.7	10/12/1970	8/10/2005	9221	0.249	0.209	1,481,659	1,108,680	17,352
04197100	Honey Creek at Melmore OH	4100011	OH	41.02214	-83.1096	385.9	2/2/1976	9/30/2004	9883	0.178	0.120	33,845	23,385	910
04198000	Sandusky River near Fremont OH	4100011	OH	41.30779	-83.1584	3,240.1	4/12/1973	9/30/2004	8982	0.190	0.124	360,004	233,945	7,463
04199000	Huron River at Milan OH	4100012	OH	41.30077	-82.6088	960.9	10/2/1973	8/20/1998	2587	0.278	0.186	107,714	90,937	5,406
04199500	Vermilion River near Vermilion OH	4100012	OH	41.38199	-82.3169	678.6	4/12/1973	9/30/2004	1462	0.146	0.060	96,328	72,433	8,717
04200500	Black River at Elyria OH	4110001	OH	41.37967	-82.1047	1,025.6	6/20/1973	12/9/1998	318	0.427	0.210	33,601	67,389	5,231
04206000	Cuyahoga River at Old Portage OH	4110002	OH	41.13559	-81.5472	1,046.4	9/10/1974	7/15/2002	31	0.167	0.100	77,981	52,740	12,753
04208000	Cuyahoga River at Independence OH	4110002	OH	41.39532	-81.6294	1,831.1	9/11/1973	9/30/2004	7652	0.316	0.240	185,992	182,041	2,877
04209000	Chagrin River at Willoughby OH	4110003	OH	41.63136	-81.4027	637.1	8/23/1971	12/1/1998	243	0.132	0.070	26,265	36,126	4,961
04212100	Grand River near Painesville OH	4110004	OH	41.7188	-81.2278	1,774.2	11/26/1975	9/30/2004	5196	0.092	0.050	91,204	101,219	2,276
04213500	CATTARAUGUS CREEK AT GOWANDA NY	4120102	NY	42.46389	-78.9352	1,129.2	7/16/1975	2/26/1998	158	0.086	0.022	136,763	42,311	32,839
04214500	BUFFALO CREEK AT GARDENVILLE NY	4120103	NY	42.85478	-78.755	367.8	10/22/1970	11/6/2006	63	0.049	0.020	23,993	20,824	8,312
04227000	CANASERAGA CREEK AT SHAKERS CROSSING NY	4130002	NY	42.7371	-77.8404	867.7	3/21/1990	11/8/2005	20	0.116	0.080			
04229500	HONEOYE CREEK AT HONEOYE FALLS NY	4130003	NY	42.95681	-77.589	507.6	3/30/1998	4/6/2005	554	0.093	0.045	9,750	23,958	491
04230500	OATKA CREEK AT GARBUTT NY	4130003	NY	43.00998	-77.7914	518.0	3/20/1990	9/16/2005	682	0.055	0.035	18,003	49,557	841
04232034	IRONDEQUOIT CR AT RAILROAD MILLS, NR FISHERS NY	4140101	NY	43.02746	-77.4784	101.5	11/27/1991	9/16/2005	604	0.122	0.060	3,679	2,017	263
0423205010	IRONDEQUOIT CR ABV BLOSSOM RD NR ROCHESTER NY	4140101	NY	43.14506	-77.5119	367.8	12/1/1980	8/31/2005	2987	0.114	0.080	21,438	12,102	868
0423205025	IRONDEQUOIT CREEK AT EMPIRE BLVD, ROCHESTER NY	4140101	NY	43.17567	-77.5266	391.1	6/28/1990	12/30/2002	1675	0.130	0.100	13,223	12,773	449
04260500	BLACK RIVER AT WATERTOWN NY	4150101	NY	43.98543	-75.9251	4,827.8	10/8/1970	8/9/1994	210	0.032	0.030	136,734	129,150	9,327
05046000	OTTER TAIL RIVER BL ORWELL D NR FERGUS FALLS, MN	9020103	MN	46.20961	-96.1849	4,506.6	4/18/1985	6/16/2003	66	0.053	0.042	32,095	70,214	4,545
05046450	OTTER TAIL RIVER AB BRECKENRIDGE, MN	9020103	MN	46.26163	-96.5462	4,382.1	5/20/1997	6/17/2003	48	0.090	0.080	65,764	113,048	20,661
05046502	OTTER TAIL RIVER AT 11TH ST IN BRECKENRIDGE, MN	9020103	MN	46.2744	-96.5801	4,416.0	9/1/2001	9/10/2007	130	0.102	0.092	59,461	117,277	5,726
05049000	MUSTINKA RIVER AB WHEATON, MN	9020102	MN	45.82079	-96.4906	1,731.8	6/1/1994	9/4/2003	31	0.210	0.190	178,436	146,997	27,348
05051300	BOIS DE SIOUX RIVER NEAR DORAN, MN	9020101	ND	46.1523	-96.5794	4,869.2	3/27/1993	5/24/2005	46	0.298	0.250	121,580	95,440	38,031
05051510	RED RIVER OF THE NORTH BELOW WAHPETON, ND	9020104	MN	46.37444	-96.658	10,411.8	10/3/1970	11/6/2006	294	0.202	0.159	181,760	232,060	14,908
05051522	RED RIVER OF THE NORTH AT HICKSON, ND	9020104	ND	46.65953	-96.7974	11,137.0	11/3/1975	5/12/2004	103	0.195	0.175	248,151	269,210	30,909
05053000	WILD RICE RIVER NR ABERCROMBIE, ND	9020105	ND	46.46758	-96.7837	5,387.2	4/6/1993	11/6/2006	130	0.268	0.222	300,842	209,347	28,712
05053800	RED RIVER OF THE NORTH ABOVE FARGO, ND	9020104	ND	46.80385	-96.7968	14,660.2	4/4/1994	5/12/2004	45	0.220	0.210	600,658	537,061	135,783
05054000	RED RIVER OF THE NORTH AT FARGO, ND	9020104	ND	46.86106	-96.7837	17,612.0	5/8/2003	8/23/2005	30	0.247	0.203	700,335	541,126	176,613
05056000	SHEYENNE RIVER NR WARWICK, ND	9020203	ND	47.80515	-98.7165	5,361.3	7/14/1993	10/3/2006	47	0.217	0.198	34,058	25,234	4,633
05057000	SHEYENNE RIVER NR COOPERSTOWN, ND	9020203	ND	47.43274	-98.0277	16,757.3	3/6/1979	8/22/2005	83	0.229	0.220	86,083	81,965	15,762
05057200	BALDHILL CREEK NR DAZEY, ND	9020203	ND	47.22916	-98.1249	1,789.7	3/21/1979	8/8/1995	48	0.155	0.110	18,772	25,824	3,377
05058000	SHEYENNE RIVER BELOW BALDHILL DAM, ND	9020204	ND	47.03367	-98.0832	19,347.3	3/21/1979	8/22/2005	45	0.193	0.190	76,599	99,852	10,049
05058700	SHEYENNE RIVER AT LISBON, ND	9020204	ND	46.44673	-97.6795	21,212.1	2/9/1993	5/10/2005	30	0.252	0.220	150,079	146,796	33,254
05059000	SHEYENNE RIVER NEAR KINDRED, ND	9020204	ND	46.62888	-97.0042	22,792.0	7/27/1976	11/6/2006	308	0.192	0.164	145,586	198,813	8,445
05062500	WILD RICE RIVER AT TWIN VALLEY, MN	9020108	MN	47.26558	-96.2473	2,419.1	9/24/1974	4/30/1997	176	0.059	0.040	40,150	49,182	3,628
05064000	WILD RICE RIVER AT HENDRUM, MN	9020108	MN	47.26754	-96.7979	4,040.4	4/6/1976	5/12/2004	115	0.104	0.060	125,819	99,968	53,375
05064500	RED RIVER OF THE NORTH AT HALSTAD, MN	9020107	ND	47.35182	-96.8439	56,462.0	1/27/1978	8/23/2005	125	0.332	0.280	1,112,339	1,260,133	169,498
05064900	BEAVER CREEK NR FINLEY, ND	9020109	ND	47.59452	-97.7094	414.4	4/10/1974	8/5/1996	83	0.209	0.175	7,346	19,223	1,235
05076000	THIEF RIVER NEAR THIEF RIVER FALLS, MN	9020304	MN	48.18555	-96.1701	2,551.2	7/20/1992	9/13/2007	91	0.246	0.072	68,463	13,441	11,467
05079000	RED LAKE RIVER AT CROOKSTON, MN	9020303	MN	47.77596	-96.6099	13,649.3	11/17/1972	8/14/2007	236	0.111	0.060	190,233	121,658	24,234
05082500	RED RIVER OF THE NORTH AT GRAND FORKS, ND	9020301	ND	47.92705	-97.0287	77,959.0	2/8/1993	2/8/2006	70	0.193	0.160	1,521,901	1,585,051	148,294
05082625	TURTLE RIVER AT TURTLE R STATE PARK NR ARVILLA, ND	9020307	ND	47.93195	-97.5148	805.5	2/8/1993	8/10/2000	85	0.148	0.080	15,030	12,587	2,972
05083500	RED RIVER OF THE NORTH AT OSLO, MN	9020306	ND	48.19419	-97.1411	80,808.0	3/15/1973	5/12/2004	56	0.331	0.230			
05085000	FOREST RIVER AT MINTO, ND	9020308	ND	48.28611	-97.3679	1,916.6	4/19/1994	2/8/2006	41	0.146	0.117	26,596	28,958	8,658
05090000	PARK RIVER AT GRAFTON, ND	9020310	ND	48.4247	-97.412	1,800.1	4/19/1994	2/8/2006	36	0.182	0.142	36,107	50,451	10,384
05099400	LITTLE SOUTH PEMBINA RIVER NR WALHALLA, ND	9020313	ND	48.86517	-98.0068	471.4				0.332	0.320			
05116500	DES LACS RIVER AT FOXHOLM, ND	9010002	ND	48.3705	-101.57	2432+G33.	11/5/1981	11/1/2006	116	0.387	0.301	5,370	12,911	619
05124480	KAWISHIWI RIVER NEAR ELY, MN	9030001	MN	47.92327	-91.5342	657.9	10/21/1970	5/15/2001	108	0.020	0.010	1,442	9,123	411
052	MISSISSIPPI R SHIELY CO. DOCK, GREY CLOUD ISLAND	7010206	MN	44.80278	-93.0139	90,034.4	3/7/1975	2/11/1998	263	0.305	0.264	3,744,481	5,663,521	119,884
05267000	MISSISSIPPI RIVER NEAR ROYALTON, MN	7010201	MN	45.82612	-94.3566	30,044.0	1/22/1975	8/5/1998	173	0.044	0.040	243,125	359,450	18,369
05280000	CROW RIVER AT ROCKFORD, MN	7010204	MN	45.08659	-93.7341	6,837.6	4/28/1971	9/18/2006	108	0.324	0.320	423,981	515,597	20,950
05283500	MISSISSIPPI RIVER AT ANOKA, MN	7010206	MN	45.19163	-93.3947	44,755.2	8/6/1998	8/6/1998	1	0.113	0.113			
05286000	RUM RIVER NEAR ST. FRANCIS, MN	7010207	MN	45.32757	-93.3727	3,522.4	4/2/1997	4/2/1997	1	0.190	0.190			
05287890	ELM CREEK NR CHAMPLIN, MN	7010206	MN	45.1633	-93.4365	222.7	2/26/1988	9/8/2005	321	0.177	0.140	10,398	10,469	709

05288500	MISSISSIPPI RIVER NEAR ANOKA, MN	7010206	MN	45.12671	-93.2964	49,469.0	4/4/1996	8/7/1998	25	0.071	0.069			
05291000	WHETSTONE RIVER NEAR BIG STONE CITY, SD	7020001	SD	45.29189	-96.4872	1,030.8	4/11/1983	1/13/2005	112	0.208	0.168	15,560	38,381	2,179
05294000	POMME DE TERRE RIVER AT APPLETON, MN	7020002	MN	45.20355	-96.0195	2,344.0	8/11/1971	9/27/2007	325	0.203	0.177	52,138	132,420	3,268
05316500	REDWOOD RIVER NEAR REDWOOD FALLS, MN	7020006	MN	44.52366	-95.1717	1,629.1	6/20/1990	9/6/2007	206	0.525	0.397	169,966	181,631	14,027
05317000	COTTONWOOD RIVER NEAR NEW ULM, MN	7020008	MN	44.29119	-94.4405	3,367.0	8/23/1989	9/6/2007	201	0.176	0.107	369,055	263,462	43,666
05319500	WATONWAN RIVER NEAR GARDEN CITY, MN	7020010	MN	44.0465	-94.1948	2,204.1	9/14/1976	9/27/2007	445	0.283	0.238	151,505	214,172	5,505
05320270	LITTLE COBB RIVER NEAR BEAUFORD, MN	7020011	MN	43.99641	-93.9087	336.7	4/24/1996	9/7/2005	98	0.203	0.166	13,433	31,197	1,949
05322000	BLUE EARTH RIVER AT MOUTH AT MANKATO, MN	7020007	MN	44.1633	-94.0369	9,056.9	10/6/1970	9/21/2006	528	0.237	0.198	463,675	1,075,196	29,968
05325000	MINNESOTA RIVER AT MANKATO, MN	7020007	MN	44.16966	-94.0031	38,591.0	3/4/1994	8/27/1997	93	0.206	0.220	1,805,372	2,758,729	283,361
05330000	MINNESOTA RIVER NEAR JORDAN, MN	7020012	MN	44.69314	-93.6419	41,958.0	10/20/1971	9/3/1998	229	0.230	0.200	1,319,204	2,978,117	102,350
05331000	MISSISSIPPI RIVER AT ST. PAUL, MN	7010206	MN	44.93309	-93.1046	95,312.0	10/4/1972	8/22/1996	329	0.217	0.180	3,695,044	4,651,807	137,344
05331570	MISSISSIPPI RIVER AT NININGER, MN	7010206	MN	44.77583	-92.8984	95,830.0	10/18/1977	9/8/1995	95	0.234	0.220	3,110,157	5,675,694	158,813
05331580	MISSISSIPPI RIVER BELOW L&D #2 AT HASTINGS, MN	7010206	MN	44.74663	-92.8524	96,089.0	10/2/1995	8/13/2004	96	0.203	0.196	2,596,869	5,696,120	162,930
05333500	ST. CROIX RIVER NEAR DANBURY, WI	7030001	WI	46.07524	-92.2477	4,092.2	10/23/1975	11/16/2004	121	0.023	0.020	25,933	33,952	2,905
05338500	SNAKE RIVER NEAR PINE CITY, MN	7030004	MN	45.84057	-92.9319	2,522.7	8/4/1971	10/10/2006	164	0.106	0.080	49,755	62,447	3,157
05340500	ST. CROIX RIVER AT ST. CROIX FALLS, WI	7030005	MN	45.40688	-92.6482	16,161.6	10/2/1974	5/8/2007	197	0.040	0.036	196,464	262,216	16,930
05355200	CANNON RIVER AT WELCH, MN	7040002	MN	44.5645	-92.7316	3,470.6	10/30/1991	8/21/2007	78	0.274	0.212	198,532	236,461	18,183
05367500	RED CEDAR RIVER NEAR COLFAX, WI	7050007	WI	45.05308	-91.7119	2,823.1	10/2/1989	10/15/2003	125	0.181	0.135	136,511	77,747	16,086
05368000	HAY RIVER AT WHEELER, WI	7050007	WI	45.04785	-91.911	1,082.6	10/2/1989	10/17/2002	118	0.140	0.094	50,248	45,234	6,382
05369500	CHIPPEWA RIVER AT DURAND, WI	7050005	WI	44.62891	-91.9702	23,335.9	10/2/1974	9/5/2007	328	0.098	0.086	670,992	511,878	24,950
05378183	JOOS VALLEY CREEK NEAR FOUNTAIN CITY, WI	7040003	WI	44.21479	-91.6651	15.3	8/8/1990	9/30/2007	399	0.544	0.054	5,206	862	2,578
05378185	EAGLE CREEK AT CT HIGHWAY G NEAR FOUNTAIN CITY, WI	7040003	WI	44.2094	-91.6784	37.0	8/8/1990	9/25/2005	340	0.554	0.070	9,442	2,052	3,978
05378500	MISSISSIPPI RIVER AT WINONA, MN	7040003	MN	44.05672	-91.6367	153,328.0	12/3/1970	8/20/1986	112	0.151	0.150	4,993,905	7,242,983	209,712
05379500	TREMPEALEAU RIVER AT DODGE, WI	7040005	WI	44.1316	-91.5531	1,665.4	1/24/1977	9/18/2007	128	0.389	0.362	269,058	76,532	15,263
05382000	BLACK RIVER NEAR GALESVILLE, WI	7040007	WI	44.06063	-91.2872	5,387.2	10/6/1970	9/18/2007	264	0.149	0.140	297,770	205,199	13,895
05388250	Upper Iowa River near Dorchester, IA	7060002	IA	43.42111	-91.5088	1,994.3	4/21/1999	9/12/2007	81	0.256	0.140	235,284	188,333	38,675
054	MISSISSIPPI RIVER AT BR ON MN-25 AT MONTICELLO	7010203	MN	45.30886	-93.7919	34,018.7	9/30/1988	9/7/1994	55	0.084	0.078	409,638	663,682	38,988
05401050	TENMILE CREEK NEAR NEKOOSA, WI	7070003	WI	44.26262	-89.8106	189.8	11/15/1973	10/17/2002	43	0.034	0.030	2,261	5,495	416
05405000	BARABOO RIVER NEAR BARABOO, WI	7070004	WI	43.48174	-89.6365	1,577.3	2/28/1977	9/17/2007	91	0.214	0.200	104,956	87,170	6,448
05406491	GARFOOT CREEK NEAR CROSS PLAINS, WI	7070005	WI	43.10998	-89.6794	14.0	10/16/1984	6/15/2001	359	0.408	0.140	1,378	647	181
05406500	BLACK EARTH CREEK AT BLACK EARTH, WI	7070005	WI	43.13388	-89.7325	118.1	4/28/1976	8/14/2003	84	0.233	0.100	6,435	5,616	1,287
05407000	WISCONSIN RIVER AT MUSCODA, WI	7070005	WI	43.1995	-90.4433	26,936.0	10/24/1974	9/17/2007	225	0.086	0.080	703,576	1,280,826	35,425
05407500	KICKAPOO RIVER AT ONTARIO, WI	7070006	WI	43.71476	-90.5869	388.5	1/17/1974	12/2/1998	221	0.149	0.120	19,578	27,289	2,838
05412500	Turkey River at Garber, IA	7060004	IA	42.73972	-91.2616	4,001.6	10/30/1979	9/12/2007	134	0.429	0.128	769,794	406,452	141,964
05413500	GRANT RIVER AT BURTON, WI	7060003	WI	42.72037	-90.8195	696.7	11/19/1986	10/16/2003	53	0.206	0.170	29,602	77,294	6,123
05418500	Maquoketa River near Maquoketa, IA	7060006	IA	42.08363	-90.6325	4,022.3	12/9/1970	6/5/2002	64	0.402	0.220	506,464	404,612	220,308
05418950	APPLE RIVER NEAR ELIZABETH, IL	7060005	IL	42.31862	-90.2543	536.1	3/20/1978	12/9/1998	142	0.223	0.145	49,873	42,833	7,412
05420500	Mississippi River at Clinton, IA	7080101	IL	41.77928	-90.2511	221,704.0	2/11/1974	9/7/2005	284	0.178	0.168	8,678,151	11,044,731	356,110
05420680	Wapsipinicon River near Tripoli, IA	7080102	IA	42.83627	-92.2571	896.1	4/18/1996	8/3/2004	73	0.131	0.096	48,007	54,930	8,038
05422000	Wapsipinicon River near De Witt, IA	7080103	IA	41.76649	-90.5347	6,050.2	3/14/1996	9/5/2007	147	0.251	0.209	587,571	361,295	45,422
05423000	WEST BRANCH ROCK R NEAR WAUPUN, WI	7090001	WI	43.66803	-88.6524	105.4	3/30/1998	12/12/2000	81	1.161	1.210	15,476	9,373	1,013
05423510	WEST BRANCH ROCK R @ STATE HWY 49 NR WAUPUN,WI	7090001	WI	43.63383	-88.6846	292.7	11/19/1997	10/14/2002	159	1.666	1.290	42,001	55,144	5,342
05424057	ROCK RIVER AT HORICON, WI	7090001	WI	43.4501	-88.6323	1,181.0	11/19/1997	12/5/2002	242	0.399	0.370	202,270	109,754	14,795
05424082	ROCK RIVER AT HUSTISFORD, WI	7090001	WI	43.34537	-88.598	1,323.5	4/6/1978	11/19/2002	161	0.437	0.380	108,016	107,847	4,575
05425500	ROCK RIVER AT WATERTOWN, WI	7090001	WI	43.18806	-88.7265	2,509.7	9/1/1998	11/30/2004	95	0.240	0.259	136,687	162,969	6,656
05426000	CRAWFISH RIVER AT MILFORD, WI	7090002	WI	43.09992	-88.8497	1,973.6	2/11/1977	10/14/2003	180	0.384	0.330	129,704	103,730	5,202
05427085	ROCK RIVER AT ROBERT STREET AT FORT ATKINSON, WI	7090001	WI	42.92751	-88.8429	5,801.6	10/6/1998	10/14/2003	66	0.300	0.306	361,048	390,955	32,674
05427570	ROCK RIVER AT INDIANFORD, WI	7090001	WI	42.80392	-89.0892	6,811.7	1/27/1977	9/12/2000	279	0.299	0.260	301,809	402,181	18,455
05430175	YAHARA RIVER NEAR FULTON, WI	7090001	WI	42.82649	-89.1725	1,341.6	7/26/1977	10/14/2003	96	0.707	0.293	67,337	36,134	6,564
05430500	ROCK RIVER AT AFTON, WI	7090001	WI	42.60937	-89.0704	8,650.6	10/28/1970	9/19/2007	269	0.371	0.320	390,633	451,527	14,774
05431486	TURTLE CK AT CARVERS ROCK ROAD NEAR CLINTON, WI	7090001	WI	42.59752	-88.829	515.4	9/2/1998	10/17/2002	67	0.180	0.140	18,881	25,832	4,332
05434500	PECATONICA RIVER AT MARTINTOWN, WI	7090003	WI	42.50961	-89.7993	2,678.1	12/8/1970	9/18/2007	93	0.211	0.190	181,948	247,602	15,825
05435500	PECATONICA RIVER AT FREEPORT, IL	7090003	IL	42.30304	-89.619	3,434.3	10/11/1977	11/18/1998	139	0.269	0.260	285,035	343,636	16,495
05435800	PECATONICA RIVER AT HARRISON, IL	7090003	IL	42.42751	-89.1957	4,630.9	12/20/1977	11/17/1998	143	0.288	0.270	416,180	499,870	20,515
05437500	ROCK RIVER AT ROCKTON, IL	7090005	IL	42.44956	-89.0697	16,480.2	12/20/1977	9/12/2000	243	0.298	0.260	1,067,583	1,170,911	59,478
05438250	COON CREEK AT RILEY, IL	7090006	IL	42.1828	-88.6413	220.4	5/9/1983	12/11/1998	141	0.105	0.070	3,624	12,217	318
05438600	KISHWAUKEE R AB SOUTH BRANCH NR PERRYVILLE, IL	7090006	IL	42.2018	-88.9792	1,696.5	12/20/1977	12/10/1998	147	0.148	0.120	59,108	99,950	3,411
05439500	SOUTH BRANCH KISHWAUKEE RIVER NR FAIRDALE IL	7090006	IL	42.11032	-88.9008	1,002.3	12/21/1977	12/10/1998	148	0.342	0.270	74,709	117,675	5,167
05440000	KISHWAUKEE RIVER NEAR PERRYVILLE, IL	7090006	IL	42.19379	-89.0002	2,846.4	3/16/1978	11/6/1998	142	0.191	0.150	142,212	227,204	9,318
05440700	ROCK RIVER AT BYRON, IL	7090005	IL	42.12273	-89.2557	19,456.8	12/21/1977	12/16/1998	183	0.302	0.290	1,638,310	1,536,564	52,640

05442200	ROCK RIVER AT GRAND DETOUR, IL	7090005	IL	41.88998	-89.4206	22,020.2	12/21/1977	12/16/1998	165	0.299	0.280	1,880,722	1,633,532	70,483
05443500	ROCK RIVER AT COMO, IL	7090005	IL	41.78184	-89.7504	22,670.3	10/17/1977	12/16/1998	184	0.312	0.290	2,021,341	1,634,115	61,518
05444000	ELKHORN CREEK NEAR PENROSE, IL	7090005	IL	41.90255	-89.6967	378.1	7/7/1983	12/17/1998	141	0.237	0.150	31,373	45,565	4,621
05446500	ROCK RIVER NEAR JOSLIN, IL	7090005	IL	41.55601	-90.1853	24,731.9	11/13/1974	12/16/1998	241	0.311	0.290	1,689,196	1,813,539	124,230
05447500	GREEN RIVER NEAR GENESEO, IL	7090007	IL	41.48886	-90.1578	2,597.8	12/27/1977	12/16/1998	176	0.215	0.120	154,287	141,485	13,553
05449500	Iowa River near Rowan, IA	7080207	IA	42.75993	-93.6223	1,082.6	3/28/1983	11/7/2006	169	0.213	0.181	91,935	102,625	7,715
05451210	South Fork Iowa River NE of New Providence, IA	7080207	IA	42.31483	-93.1524	580.2	3/19/1996	6/2/2005	104	0.173	0.065	29,029	95,328	6,168
05453100	Iowa River at Marengo, IA	7080208	IA	41.81218	-92.0643	7,236.5	5/23/1985	9/4/1998	42	0.421	0.343	787,093	772,510	80,607
05455100	Old Mans Creek near Iowa City, IA	7080209	IA	41.60642	-91.6157	520.6	6/14/1995	9/4/2007	213	0.311	0.170	83,306	70,329	10,229
05458000	Little Cedar River near Ionia, IA	7080201	IA	43.03352	-92.5037	792.5	4/19/2001	12/8/2004	37	0.371	0.150	131,375	61,847	45,569
05458900	West Fork Cedar River at Finchford, IA	7080204	IA	42.62936	-92.5432	2,191.1	1/12/1978	9/4/2007	243	0.186	0.130	170,572	157,909	11,261
05463050	Cedar River at Cedar Falls, IA	7080205	IA	42.53935	-92.4497	12,261.1	7/8/1974	9/4/2007	193	0.200	0.170	766,540	835,193	53,805
05464020	Cedar River at Gilbertville, IA	7080205	IA	42.41582	-92.2188	13,571.6	10/6/1970	9/4/2007	207	0.299	0.250	864,621	954,372	58,750
05464220	Wolf Creek near Dysart, IA	7080205	IA	42.2517	-92.2988	774.4	3/21/1996	5/24/2004	56	0.255	0.130	54,051	76,999	15,827
05464760	Cedar River near Bertram, IA	7080206	IA	41.92572	-91.5502	18,013.5	7/8/1974	9/5/2007	194	0.365	0.330	1,441,426	1,262,778	105,810
05465500	Iowa River at Wapello, IA	7080209	IA	41.17835	-91.181	32,375.0	11/1/1983	9/1/2005	194	0.310	0.280	2,823,764	2,747,743	164,947
05466500	EDWARDS RIVER NEAR NEW BOSTON, IL	7080104	IL	41.18696	-90.9676	1,152.6	1/18/1978	12/16/1998	138	0.295	0.164	137,844	103,623	16,048
05469000	HENDERSON CREEK NEAR OQUAWKA, IL	7080104	IL	41.00188	-90.8543	1,118.9	2/8/1984	12/16/1998	125	0.712	0.485	116,139	117,825	8,668
05471500	South Skunk River near Oskaloosa, IA	7080105	IA	41.35524	-92.6571	4,234.7	10/11/1999	9/11/2007	93	0.405	0.290	641,787	427,950	86,151
05474000	Skunk River at Augusta, IA	7080107	IA	40.75317	-91.2761	11,168.1	11/9/1977	9/7/2005	195	0.345	0.240	1,247,269	1,297,934	107,353
05474500	Mississippi River at Keokuk, IA	7080104	IL	40.39247	-91.3718	308,210.0	3/17/1975	7/22/1999	142	0.237	0.200	14,511,018	17,976,380	1,021,564
05476000	DES MOINES RIVER AT JACKSON, MN	7100002	MN	43.61937	-94.9843	3,237.5	10/12/1972	6/29/1976	39	0.284	0.260	135,251	227,147	19,705
05481650	Des Moines River near Saylorville, IA	7100004	IA	41.68033	-93.6688	15,128.2	7/14/1975	9/6/2007	118	0.190	0.140	533,705	1,140,303	48,477
05484500	Raccoon River at Van Meter, IA	7100006	IA	41.53414	-93.9499	8,912.2	7/14/1975	9/6/2007	176	0.273	0.185	703,714	752,322	66,391
05486000	North River near Norwalk, IA	7100008	IA	41.45784	-93.655	903.9	7/15/1987	9/6/2007	235	0.413	0.300	55,579	72,838	6,796
05490600	Des Moines River at St. Francisville, MO	7100009	IA	40.46211	-91.5671	37,037.0	10/19/1970	11/2/2006	275	0.266	0.220	2,619,575	2,961,891	184,432
05495000	Fox River at Wayland, MO	7110001	MO	40.39215	-91.5981	1,036.0	10/19/1970	9/7/2005	57	0.257	0.120	176,411	117,454	46,034
05495500	BEAR CREEK NEAR MARCELLINE, IL	7110001	IL	40.14358	-91.3415	903.9	2/2/1984	12/22/1998	136	0.266	0.150	151,377	116,703	23,264
05500000	South Fabius River near Taylor, MO	7110003	MO	39.89678	-91.5799	1,605.8	7/18/1972	9/7/2005	260	0.154	0.095	124,810	158,098	13,111
05514500	Cuivre River near Troy, MO	7110008	MO	39.00937	-90.9764	2,338.8	7/19/1972	9/7/2005	160	0.154	0.095	313,064	341,205	42,665
05518000	KANKAKEE RIVER AT SHELBY, IN	7120001	IN	41.18248	-87.3435	4,607.6	1/6/1976	2/15/2006	327	0.149	0.080	154,480	179,914	9,635
05520500	KANKAKEE RIVER AT MOMENCE, IL	7120001	IL	41.15944	-87.6682	5,941.5	1/19/1978	2/21/2001	232	0.093	0.070	210,048	220,877	17,127
05525000	IROQUOIS RIVER AT IROQUOIS, IL	7120002	IL	40.82321	-87.5818	1,776.7	4/10/1984	12/9/1998	135	0.136	0.130	77,497	76,418	5,679
05525500	SUGAR CREEK AT MILFORD, IL	7120002	IL	40.63164	-87.7243	1,155.1	7/30/1981	12/15/2004	208	0.137	0.084	86,209	103,428	10,367
05526000	IROQUOIS RIVER NEAR CHEBANSE, IL	7120002	IL	41.00908	-87.8253	5,415.7	4/21/1978	2/21/2001	214	0.147	0.120	390,669	320,874	36,554
05527500	KANKAKEE RIVER NEAR WILMINGTON, IL	7120001	IL	41.34607	-88.1881	13,338.5	12/14/1977	12/2/1998	207	0.119	0.090	720,763	658,735	57,770
05527800	DES PLAINES RIVER AT RUSSELL, IL	7120004	IL	42.48755	-87.9255	318.6	12/29/1977	8/9/2001	190	0.186	0.160	11,637	22,221	850
05528000	DES PLAINES RIVER NEAR GURNEE, IL	7120004	IL	42.34398	-87.9406	600.9	12/20/1977	12/1/1998	179	0.767	0.590	85,857	57,673	3,446
05529000	DES PLAINES RIVER NEAR DES PLAINES, IL	7120004	IL	42.08194	-87.8903	932.4	1/18/1978	12/1/1998	150	0.704	0.610	139,896	100,761	5,192
05530590	DES PLAINES RIVER NEAR SCHILLER PARK, IL	7120004	IL	41.95333	-87.8546	1,150.0	9/19/1978	11/30/1998	183	0.681	0.590	190,196	148,698	7,719
05531500	SALT CREEK AT WESTERN SPRINGS, IL	7120004	IL	41.82604	-87.9	297.9	12/14/1977	8/17/2005	272	1.595	1.500	181,607	68,549	6,613
05532500	DES PLAINES RIVER AT RIVERSIDE, IL	7120004	IL	41.82278	-87.8201	1,631.7	4/9/1987	8/18/2004	190	0.871	0.765	457,766	242,798	21,884
05534050	DES PLAINES RIVER AT LOCKPORT, IL	7120004	IL	41.59642	-88.0686	1,813.0	1/19/1978	12/3/1998	167	0.761	0.680	418,734	1,029,361	14,937
05536195	LITTLE CALUMET RIVER AT MUNSTER, IND.	7120003	IN	41.57715	-87.5223	233.1	2/24/1971	2/6/2006	511	0.783	0.540	20,510	16,489	1,020
05536995	CHICAGO SANITARY AND SHIP CANAL AT ROMEOVILLE, IL	7120004	IL	41.64088	-88.06	1,914.0	4/9/1987	2/21/2001	92	1.014	0.864	3,802,886	742,978	374,169
05539000	HICKORY CREEK AT JOLIET, IL	7120004	IL	41.51445	-88.0741	278.4	6/14/1979	12/3/1998	147	0.444	0.390	37,017	26,030	2,663
05539900	WEST BRANCH DU PAGE RIVER NEAR WEST CHICAGO, IL	7120004	IL	41.91086	-88.1792	73.8	6/18/1979	12/2/1998	147	1.537	1.430	43,523	18,445	1,020
05540095	WEST BRANCH DU PAGE RIVER NEAR WARRENVILLE, IL	7120004	IL	41.82189	-88.1724	234.1	12/19/1977	12/2/1998	174	1.265	1.200	87,719	66,099	1,988
05540210	EAST BRANCH DU PAGE RIVER AT ROUTE 34 AT LISLE, IL	7120004	IL	41.80059	-88.0815	133.1	11/14/1977	12/2/1998	168	1.431	1.248	55,051	48,434	1,838
05540290	DU PAGE RIVER NEAR NAPERVILLE, IL	7120004	IL	41.69003	-88.1662	569.8	10/7/1988	12/8/1998	91	1.206	1.115	197,083	161,737	5,611
05540500	DU PAGE RIVER AT SHOREWOOD, IL	7120004	IL	41.5223	-88.1932	839.2	1/19/1978	8/20/1993	127	0.992	0.960	268,549	180,488	14,568
05542000	MAZON RIVER NEAR COAL CITY, IL	7120005	IL	41.28711	-88.3606	1,178.5	5/2/1978	12/2/1998	117	0.096	0.070	23,753	96,566	2,698
05543500	ILLINOIS RIVER AT MARSEILLES, IL	7120005	IL	41.3259	-88.7181	21,390.8	11/13/1974	12/15/1998	294	0.488	0.450	3,421,828	2,907,198	164,519
055451345	WHITE RIVER AT CENTER STREET AT LAKE GENEVA, WI	7120006	WI	42.59057	-88.4337	75.1	10/1/1997	7/7/2005	125	0.023	0.012	243	395	62
05546700	FOX RIVER NEAR CHANNEL LAKE, IL	7120006	IL	42.47919	-88.1783	2,255.9	11/8/1977	12/1/1998	198	0.227	0.120	97,199	145,017	6,426
05548280	NIPPERSINK CREEK NEAR SPRING GROVE, IL	7120006	IL	42.44352	-88.2472	497.3	12/29/1977	12/1/1998	197	0.149	0.120	18,843	24,344	1,022
05549600	FOX RIVER AT BURTONS BRIDGE, IL	7120006	IL	42.27948	-88.2271	3,232.1	7/26/1979	12/17/1998	176	0.132	0.120	104,538	177,220	3,728
05550000	FOX RIVER AT ALGONQUIN, IL	7120006	IL	42.16596	-88.2904	3,633.8	11/8/1977	12/7/1998	212	0.173	0.140	142,700	209,266	5,010
05551000	FOX RIVER AT SOUTH ELGIN, IL	7120007	IL	41.9962	-88.2941	4,030.0	11/10/1977	12/7/1998	198	0.245	0.200	216,376	249,370	6,895
05551700	BLACKBERRY CREEK NEAR YORKVILLE, IL	7120007	IL	41.67123	-88.4462	181.8	12/14/1977	12/10/1998	201	0.131	0.100	7,210	8,248	525

05551995	SOMONAUK CREEK AT SHERIDAN, IL	7120007	IL	41.54361	-88.6867	215.7	7/9/1979	12/10/1998	182	0.138	0.070	9,584	23,943	1,283
05552500	FOX RIVER AT DAYTON, IL	7120007	IL	41.38325	-88.7909	6,843.4	8/30/1971	1/13/1998	224	0.322	0.270	530,684	505,763	25,976
05553500	ILLINOIS RIVER AT OTTAWA, IL	7130001	IL	41.34199	-88.8467	28,357.9	3/8/1972	8/4/2004	148	0.705	0.576	5,959,924	3,420,271	351,291
05554490	VERMILION RIVER AT MC DOWELL, IL	7130002	IL	40.83059	-88.5748	1,427.1	9/18/1978	12/11/1998	134	0.120	0.090	43,923	131,430	4,681
05555300	VERMILION RIVER NEAR LEONORE, IL	7130002	IL	41.20959	-88.9373	3,240.1	1/23/1978	12/11/1998	137	0.288	0.210	202,902	284,336	18,657
05556200	ILLINOIS RIVER AT HENNEPIN	7130001	IL	41.25788	-89.3488	33,143.2	3/12/1984	12/15/1998	131	0.362	0.335	3,600,962	3,803,678	100,969
05556500	BIG BUREAU CREEK AT PRINCETON, IL	7130001	IL	41.36494	-89.4976	507.6	1/23/1978	12/14/1998	143	0.449	0.194	23,930	34,481	2,124
05558995	ILLINOIS RIVER AT LACON, IL	7130001	IL	41.02504	-89.4173	35,394.9	3/12/1984	12/15/1998	129	0.352	0.340	3,754,633	3,839,947	106,843
05559900	ILLINOIS RIVER AT WATER COMPANY AT PEORIA, IL	7130001	IL	40.72493	-89.5473	36,001.0	11/17/1970	12/15/1998	215	0.372	0.340	3,632,357	3,857,314	187,095
05563800	ILLINOIS RIVER AT PEKIN, IL	7130003	IL	40.57309	-89.6548	36,555.1	1/21/1980	12/14/1998	181	0.373	0.360	4,372,899	3,058,054	130,543
05567510	MACKINAW RIVER BELOW CONGERVILLE, IL	7130004	IL	40.58672	-89.2785	2,009.8	3/7/1984	12/15/1998	136	0.102	0.080	52,327	226,501	5,236
05568005	MACKINAW RIVER BELOW GREEN VALLEY, IL	7130004	IL	40.44754	-89.6912	2,828.3	8/1/1988	12/14/1998	95	0.163	0.100	183,911	308,427	20,800
05568800	INDIAN CREEK NEAR WYOMING, IL	7130005	IL	41.01846	-89.8359	162.4	1/23/1978	12/17/1998	164	0.177	0.130	6,575	13,882	639
05568915	SPOON RIVER NEAR DAHINDA, IL	7130005	IL	40.90791	-90.0868	1,976.4	2/9/1984	12/17/1998	134	0.192	0.128	107,499	178,080	10,209
05569500	SPOON RIVER AT LONDON MILLS, IL	7130005	IL	40.70676	-90.2829	2,776.5	2/8/1984	12/16/1998	135	0.226	0.140	180,585	265,696	19,368
05570000	SPOON RIVER AT SEVILLE, IL	7130005	IL	40.48497	-90.3422	4,236.7	6/17/1981	12/28/1998	141	0.276	0.150	369,019	387,141	41,931
05570520	ILLINOIS RIVER AT POWER COMPANY AT HAVANA, IL	7130003	IL	40.28032	-90.0815	47,397.0	4/24/1978	11/30/1998	141	0.349	0.340	5,117,590	3,895,850	132,481
05570910	SANGAMON RIVER AT FISHER, IL	7130006	IL	40.31119	-88.3227	621.6	1/17/1979	11/23/1998	184	0.139	0.110	27,097	53,524	2,644
05572000	SANGAMON RIVER AT MONTICELLO, IL	7130006	IL	40.02573	-88.5891	1,424.5	1/27/1972	8/16/2005	160	0.205	0.150	67,489	143,505	8,282
05572125	SANGAMON R AT ALLERTON PARK NEAR MONTICELLO, IL	7130006	IL	40.00226	-88.6353	1,489.4	10/19/1978	12/21/1998	174	0.203	0.170	70,759	146,027	5,445
05573504	SANGAMON R. AT L DECATUR WATER INTAKE AT DECATUR	7130006	IL	39.82893	-88.9597	2,403.6	11/4/1982	12/1/1998	137	0.143	0.119	83,308	219,089	6,328
05573540	SANGAMON RIVER AT ROUTE 48 AT DECATUR, IL	7130006	IL	39.83094	-88.9763	2,429.4	5/26/1983	12/1/1998	132	0.215	0.130	86,658	221,702	7,735
05573650	SANGAMON RIVER NEAR NIANTIC, IL	7130006	IL	39.79723	-89.1054	2,737.8	5/26/1983	12/1/1998	137	1.540	0.625	270,900	280,726	18,658
05573800	SANGAMON RIVER AT ROBY, IL	7130006	IL	39.74227	-89.3992	3,273.8	6/6/1983	11/24/1998	129	1.132	0.460	286,180	319,687	21,421
05575500	SOUTH FORK SANGAMON RIVER AT KINCAID, IL	7130007	IL	39.57884	-89.3925	1,455.6	5/3/1978	11/24/1998	184	0.349	0.250	92,296	148,050	7,216
05576022	SOUTH FORK SANGAMON RIVER BELOW ROCHESTER, IL	7130007	IL	39.75411	-89.5662	2,253.3	3/17/1981	12/2/1998	142	0.279	0.220	105,469	196,225	7,945
05576500	SANGAMON RIVER AT RIVERTON, IL	7130008	IL	39.84754	-89.55	6,780.6	1/9/1986	12/2/1998	116	0.871	0.440	521,784	603,864	32,882
05577505	SPRING CR AT BURNS LANE BRIDGE AT SPRINGFIELD,IL	7130008	IL	39.82055	-89.6877	282.3	4/19/1979	12/2/1998	140	0.298	0.190	10,284	15,855	990
05578000	SANGAMON RIVER AT PETERSBURG, IL	7130008	IL	40.01102	-89.844	7,933.2	5/1/1978	11/30/1998	145	0.723	0.450	798,619	701,245	37,078
05578500	SALT CREEK NEAR ROWELL, IL	7130009	IL	40.11457	-89.0505	867.7	7/20/1979	12/29/1998	151	0.218	0.140	25,404	61,414	1,565
05579500	LAKE FORK NEAR CORNLAND, IL	7130009	IL	39.95123	-89.3841	554.3	5/4/1978	12/29/1998	152	0.158	0.090	30,703	41,847	3,295
05580000	KICKAPOO CREEK AT WAYNESVILLE, IL	7130009	IL	40.25517	-89.1283	587.9	5/11/1981	12/29/1998	145	0.134	0.070	28,979	50,008	4,393
05580500	KICKAPOO CREEK NEAR LINCOLN, IL	7130009	IL	40.1864	-89.3668	792.5	3/27/1979	12/7/1998	150	0.146	0.080	41,857	73,206	5,936
05582000	SALT CREEK NEAR GREENVIEW, IL	7130009	IL	40.13304	-89.7367	4,672.4	5/1/1978	12/7/1998	154	0.262	0.210	302,202	408,778	19,417
05583000	SANGAMON RIVER NEAR OAKFORD, IL	7130008	IL	40.12302	-89.9852	13,190.9	12/20/1977	11/30/1998	225	0.419	0.348	1,084,438	1,145,867	46,533
05584500	LA MOINE RIVER AT COLMAR, IL	7130010	IL	40.33088	-90.896	1,696.5	2/17/1975	12/16/1998	358	0.273	0.170	119,915	139,324	11,552
05585000	LA MOINE RIVER AT RIPLEY, IL	7130010	IL	40.02384	-90.6308	3,348.9	7/16/1975	12/14/1998	285	0.286	0.180	336,372	267,533	24,910
05586100	ILLINOIS RIVER AT VALLEY CITY, IL	7130011	IL	39.70341	-90.6462	69,267.0	12/12/1974	8/18/2005	377	0.391	0.360	7,436,764	5,459,811	284,665
05587000	MACOUPIN CREEK NEAR KANE, IL	7130012	IL	39.23422	-90.3946	2,248.1	6/28/1978	11/22/1998	139	0.325	0.210	292,897	257,172	27,870
05587455	MISSISSIPPI RIVER BELOW GRAFTON, IL	7110009	IL	38.95033	-90.3715	443,667.0	5/4/1989	9/12/2005	183	0.242	0.203	31,359,903	29,524,914	1,762,241
05587900	CAHOKIA CREEK AT EDWARDSVILLE, IL	7140101	IL	38.82447	-89.975	549.1	2/14/1984	12/15/1998	133	0.381	0.200	78,741	52,120	11,678
05591200	KASKASKIA RIVER AT COOKS MILLS, IL	7140201	IL	39.58315	-88.4132	1,225.1	12/1/1977	12/3/1998	194	0.270	0.200	74,259	85,212	6,444
05591300	KASKASKIA RIVER AT ALLENVILLE, IL	7140201	IL	39.57281	-88.5322	1,310.5	1/16/1980	12/3/1998	175	0.231	0.180	75,815	89,178	5,581
05591400	JONATHAN CREEK NEAR SULLIVAN, IL	7140201	IL	39.60111	-88.5461	141.7	3/3/1980	12/7/1998	154	0.152	0.095	3,887	5,490	479
05591700	WEST OKAW RIVER NEAR LOVINGTON, IL	7140201	IL	39.73133	-88.6623	290.1	4/7/1980	12/7/1998	153	0.126	0.070	10,012	22,362	1,144
05592000	KASKASKIA RIVER AT SHELBYVILLE, IL	7140201	IL	39.39884	-88.7881	2,729.9	10/31/1977	12/15/1998	200	0.057	0.030	49,635	134,203	4,250
05592100	KASKASKIA RIVER NEAR COWDEN, IL	7140201	IL	39.22962	-88.8449	3,444.7	10/17/1977	12/21/1998	200	0.135	0.090	127,641	215,456	9,554
05592195	BECK CREEK AT HERRICK, IL	7140201	IL	39.21639	-89.0206	251.2	10/22/1979	12/21/1998	173	0.229	0.192	19,111	25,803	1,773
05592500	KASKASKIA RIVER AT VANDALIA, IL	7140202	IL	38.95644	-89.0877	5,024.6	10/3/1977	12/22/1998	194	0.202	0.160	293,963	417,422	18,397
05592600	HICKORY CREEK NEAR BLUFF CITY, IL	7140202	IL	38.92525	-89.0393	201.5	1/19/1989	12/22/1998	74	0.188	0.130	20,157	20,553	5,603
05592800	HURRICANE CREEK NEAR MULBERRY GROVE, IL	7140202	IL	38.91851	-89.2428	393.7	5/3/1978	12/22/1998	191	0.221	0.170	53,942	50,585	7,099
05592900	EAST FORK KASKASKIA RIVER NEAR SANDOVAL, IL	7140202	IL	38.69236	-89.0999	292.7	12/12/1979	12/17/1998	168	0.245	0.200	36,749	31,258	4,185
05592930	NORTH FORK KASKASKIA RIVER NEAR PATOKA, IL	7140202	IL	38.77361	-89.0861	101.3	1/19/1989	12/17/1998	75	0.355	0.290	13,616	9,302	3,139
05593010	KASKASKIA RIVER BELOW CARLYLE, IL	7140202	IL	38.57449	-89.3693	7,081.1	10/28/1977	12/15/1998	197	0.171	0.150	300,038	540,964	14,124
05593505	CROOKED CREEK NEAR ODIN, IL	7140202	IL	38.56389	-89.0503	231.0	4/20/1982	12/17/1998	136	0.510	0.305	28,790	20,526	3,800
05594000	SHOAL CREEK NEAR BREESE, IL	7140203	IL	38.60959	-89.4945	1,903.7	4/28/1982	11/17/1998	142	0.275	0.220	257,632	220,870	22,558
05594100	KASKASKIA RIVER NEAR VENEDY STATION, IL	7140204	IL	38.44995	-89.6298	11,377.9	11/12/1974	11/18/1998	236	0.267	0.220	939,056	1,216,582	55,281
05594450	SILVER CREEK NEAR TROY, IL	7140204	IL	38.7152	-89.8304	398.9	4/21/1982	12/15/1998	143	1.031	0.660	92,938	50,072	9,506
05594800	SILVER CREEK NEAR FREEBURG, IL	7140204	IL	38.39653	-89.873	1,201.8	4/27/1982	12/9/1998	145	0.664	0.540	241,572	143,850	19,081
05595200	RICHLAND CREEK NEAR HECKER, IL	7140204	IL	38.32375	-89.9711	334.1	8/28/1978	12/9/1998	147	1.209	0.890	83,719	54,062	9,083

05595700	BIG MUDDY RIVER NEAR MT. VERNON, IL	7140106	IL	38.30935	-88.9887	186.2	10/2/1985	12/2/1998	118	0.196	0.140	34,573	12,937	6,611
05595730	RAYSE CREEK NEAR WALTONVILLE, IL	7140106	IL	38.25381	-89.0399	227.9	12/20/1979	12/2/1998	161	0.233	0.140	38,207	20,006	5,817
05595830	CASEY FORK AT ROUTE 37 NEAR MT. VERNON, IL	7140106	IL	38.26947	-88.8989	227.1	12/18/1979	12/2/1998	162	0.516	0.230	25,342	13,855	4,071
05597000	BIG MUDDY RIVER AT PLUMFIELD, IL	7140106	IL	37.90148	-89.0143	2,056.5	11/17/1981	11/24/1998	135	0.173	0.160	112,025	131,200	8,041
05597500	CRAB ORCHARD CREEK NEAR MARION, IL	7140106	IL	37.73092	-88.8895	82.1	10/20/1977	11/16/1998	154	0.107	0.065	7,529	6,557	1,206
05599500	BIG MUDDY RIVER AT MURPHYSBORO, IL	7140106	IL	37.74924	-89.3471	5,617.7	11/11/1974	12/1/1998	235	0.215	0.200	412,380	437,640	36,454
07014500	Meramec River near Sullivan, MO	7140102	MO	38.15847	-91.1086	3,820.3	10/22/1970	9/1/2005	357	0.051	0.030	38,488	147,671	7,017
07016400	Bourbeuse River above Union, MO	7140103	MO	38.432	-91.0199	2,092.7	10/22/1970	9/7/2005	197	0.062	0.050	83,874	105,561	13,589
07018100	Big River near Richwoods, MO	7140104	MO	38.15988	-90.7055	1,903.7	12/20/1983	9/1/2005	118	0.041	0.030	54,406	76,839	11,960
07019280	Meramec River at Paulina Hills, MO	7140102	MO	38.46283	-90.4148	10,152.8	10/22/1970	9/8/2005	323	0.108	0.097	312,814	455,338	19,766
090162	SAGINAW R AT MIDLAND ST BR; BANGOR TWP, SEC 21	4080206	MI	43.60139	-83.8919	15,845.6	1/29/1974	6/18/1996	113	0.165	0.147	445,728	515,695	24,204
093051	CHIPPEWA R @ NSP HOLCOMBE DM TAILRACE	7050005	WI	45.22441	-91.1281	11,923.6	2/10/1977	6/6/2001	149	0.045	0.040	189,800	180,906	11,535
10070001	Beaver Creek near Cedar Falls	7080205	IA	42.5732	-92.5065	1,009.2	10/26/1998	9/4/2007	90	0.191	0.120	113,206	110,634	15,457
10070002	Wolf Creek at La Porte City	7080205	IA	42.3158	-92.1936	847.6	6/5/2001	9/4/2007	75	0.244	0.130	108,120	84,288	20,699
10070004	Black Hawk Creek at Waterloo	7080205	IA	42.4695	-92.4134	848.1	10/2/2001	9/4/2007	76	0.198	0.140	74,727	73,345	11,927
10090001	Cedar River near Janesville	7080201	IA	42.6496	-92.4664	4,287.4	10/26/1998	9/10/2007	88	0.190	0.170	305,893	286,650	23,862
10120001	Shell Rock River at Shell Rock	7080202	IA	42.7116	-92.5812	4,427.4	11/9/1999	9/10/2007	105	0.232	0.180	383,282	271,544	26,734
10160001	Cedar River at Cedar Bluff	7080206	IA	41.78801	-91.3124	18,117.6	12/6/1999	7/5/2006	79	0.332	0.320	1,598,518	1,283,470	139,486
10170002	Winnebago River Upstream of Mason City	7080203	IA	43.1929	-93.2103	1,162.4	11/11/1999	9/10/2007	85	0.214	0.150	119,852	63,265	14,619
10170003	Winnebago River Downstream of Mason City	7080203	IA	43.1257	-93.1231	1,595.9	11/11/1999	9/10/2007	86	0.302	0.295	127,998	99,110	11,479
10220002	Volga River near Elkport	7060004	IA	42.7497	-91.2766	1,042.7	2/23/2000	9/12/2007	85	0.513	0.100	242,941	104,064	81,937
10250001	South Raccoon River near Redfield	7100007	IA	41.5798	-94.1827	2,512.2	3/1/2000	9/6/2007	84	0.305	0.145	403,219	223,393	89,119
103094	BLACK RIVER	7040007	WI	44.57076	-90.5952	1,717.2	3/30/1978	9/3/2003	154	0.170	0.159	87,829	127,121	7,783
10340001	Cedar River near Charles City	7080201	IA	43.00726	-92.6029	2,833.8	1/12/1971	9/10/2007	305	0.247	0.200	177,025	196,149	11,383
10370001	North Raccoon River near Jefferson	7100006	IA	42.0119	-94.4286	4,091.6	1/11/2000	11/13/2006	87	0.332	0.250	439,122	404,441	68,635
10400001	Boone River near Stratford	7100005	IA	42.3411	-93.892	2,286.1	10/18/1999	9/10/2007	93	0.249	0.190	182,975	246,118	27,131
10420001	Iowa River near Gifford	7080207	IA	42.3102	-93.0755	2,039.0	10/7/1999	9/12/2007	87	0.205	0.150	157,325	228,315	21,236
10440001	Cedar Creek near Oakland Mills	7080107	IA	40.9254	-91.6735	1,385.9	10/12/1998	9/4/2007	98	0.303	0.200	128,223	161,444	17,000
10460001	West Fork Des Moines River near Humboldt	7100002	IA	42.6744	-94.2069	5,926.7	4/30/1981	9/11/2007	107	0.221	0.200	323,837	472,603	24,371
10490001	North Fork Maquoketa River near Hurstville	7060006	IA	42.09025	-90.6723	1,527.3	10/13/1998	9/5/2007	100	0.360	0.205	510,462	188,551	203,391
10490002	Maquoketa River near Maquoketa	7060006	IA	42.0757	-90.6823	2,470.8	3/1/2000	9/5/2007	83	0.200	0.160	218,950	203,920	27,287
10500001	Indian Creek near Colfax	7080105	IA	41.74621	-93.2264	1,038.0	1/10/2000	9/12/2007	90	0.292	0.185	102,848	77,799	16,681
10520002	Iowa River Upstream of Iowa City	7080209	IA	41.6937	-91.5466	8,122.5	11/30/1999	9/5/2007	82	0.191	0.180	407,626	781,465	31,305
10520003	Iowa River Downstream of Iowa City	7080209	IA	41.5547	-91.5186	8,565.0	11/30/1999	9/5/2007	86	0.249	0.220	526,549	821,717	35,781
10540001	North Skunk River	7080106	IA	41.28387	-92.2574	1,649.6	10/12/1998	9/11/2007	90	0.339	0.200	232,491	161,631	37,337
10550001	East Fork of The Des Moines River near St. Joseph	7100003	IA	42.95138	-94.2335	2,432.9	10/12/1998	9/11/2007	99	0.243	0.210	137,122	218,230	11,730
10570002	Cedar River Upstream of Cedar Rapids	7080205	IA	42.0692	-91.7847	16,245.2	5/9/2000	9/5/2007	80	0.224	0.200	1,237,685	1,155,266	124,788
10630001	Whitebreast Creek near Knoxville	7100008	IA	41.3026	-93.1713	917.6	3/6/2000	9/5/2007	84	0.272	0.125	118,361	85,241	30,465
10630002	Cedar Creek near Bussey	7100009	IA	41.2191	-92.9077	965.0	4/3/2000	9/5/2007	95	0.517	0.110	315,183	108,922	79,000
10640002	Iowa River Downstream of Marshalltown	7080208	IA	42.0508	-92.8464	4,254.3	1/27/1976	9/12/2007	118	0.389	0.315	361,020	534,620	28,419
10640003	Iowa River Upstream of Marshalltown	7080208	IA	42.0917	-93.0002	3,817.6	4/17/2000	9/12/2007	77	0.182	0.130	299,271	487,287	37,820
10700001	Cedar River near Conesville	7080206	IA	41.4093	-91.29	19,949.4	10/14/1999	9/4/2007	93	0.352	0.300	1,656,927	1,428,639	144,434
10770001	Beaver Creek near Grimes	7100004	IA	41.6883	-93.7353	969.3	10/11/1999	9/6/2007	92	0.332	0.200	80,548	52,258	13,284
10770003	Des Moines River Downstream of Des Moines	7100008	IA	41.4877	-93.3384	29,882.3	11/15/1999	9/6/2007	85	0.514	0.380	3,301,192	2,401,950	400,796
10850003	South Skunk River Upstream of Ames	7080105	IA	42.06631	-93.6207	822.3	10/7/1999	9/12/2007	80	0.203	0.150	79,659	115,959	14,114
10900002	Des Moines River Downstream of Ottumwa	7100009	IA	40.95677	-92.34	34,441.8	11/2/1999	9/11/2007	84	0.352	0.320	2,110,196	2,732,401	188,404
10900003	Des Moines River Upstream of Ottumwa	7100009	IA	41.0874	-92.5258	33,982.4	11/2/1999	9/11/2007	86	0.353	0.295	2,191,253	2,691,456	195,338
10910001	Middle River near Indianola	7100008	IA	41.4247	-93.5871	1,269.3	10/15/1998	9/6/2007	88	0.452	0.200	311,965	126,838	60,926
10940002	Des Moines River Upstream of Fort Dodge	7100004	IA	42.5436	-94.2163	9,531.0	6/12/2000	9/10/2007	82	0.189	0.170	657,744	783,200	69,995
123016	Mississippi R. at LD 9	7060001	WI	43.21167	-91.1025	166,212.4	2/3/1977	9/4/2007	314	0.155	0.150	5,650,573	8,111,003	163,946
123017	Kickapoo R. at Stueben	7070006	WI	43.18214	-90.8506	1,773.6	2/3/1977	9/20/2007	281	0.158	0.120	89,750	95,906	5,826
130001	Maquoketa River	7060006	IA	42.18667	-90.3106	4,818.0	5/5/1993	11/8/2005	281	0.312	0.188	501,307	465,400	58,313
130003	APPLE RIVER NEAR HANOVER, IL	7060005	IL	42.18718	-90.236	657.4	5/3/1993	11/8/2005	238	0.174	0.135	48,486	55,835	7,946
130202	BATTLE CREEK R@ 9 MILE RD; PENNFIELD TWP SEC 21	4050003	MI	42.36417	-85.1222	536.5	10/8/1979	3/1/1994	170	0.073	0.067	12,053	19,499	348
133416	SUGAR RIVER AT STH 69 DWNSTM BRIDGE	7090004	WI	42.94904	-89.5445	212.4	5/27/1997	12/5/2000	49	0.083	0.080	10,469	16,257	1,384
133417	SUGAR RIVER AT VALLEY RD.	7090004	WI	42.9725	-89.5667	120.7	5/27/1997	12/5/2000	50	0.073	0.068	3,159	10,305	245
140001	Wapsipinicon River	7080103	IA	41.7273	-90.3646	6,524.4	5/5/1993	11/8/2005	282	0.256	0.189	469,652	397,124	36,373
160376	E BR DUPAGE R .5 MI S NAPERVILLE T37N R10E NE7	7120004	IL	41.71111	-88.1278	194.2	10/22/1970	12/3/1997	70	2.624	2.400	140,998	64,919	7,378
160383	E BR DUPAGE R MAPLE AV LISLE T38N R10E SE10	7120004	IL	41.78571	-88.0792	136.9	10/22/1970	9/17/1997	66	3.312	2.705	108,141	47,918	4,834
163001	ST. LOUIS RIVER AT OLIVER	4010201	WI	46.65669	-92.2004	9,114.0	7/11/1972	11/20/1996	271	0.053	0.046	116,928	119,226	4,857

163002	BOIS BRULE RIVER	4010301	WI	46.67936	-91.5949	466.6	7/31/1973	7/24/2007	175	0.039	0.030	7,765	5,524	781
171408	MISSISSINEWA RIVER AT PERU MILE POINT 0.8	5120103	IN	40.75389	-86.0231	2,107.3	2/9/1971	12/6/1995	266	0.139	0.090	140,947	159,085	15,567
171420	ST. MARY'S RIVER NEAR FT WAYNE AT MI PT 10.83	4100004	IN	40.988	-85.099	1,800.1	2/27/1973	12/12/1995	240	0.370	0.330	239,529	258,427	16,025
171433	WABASH R AT US 27 BRIDGE N OF GENEVA, MI PT451.9	5120101	IN	40.61667	-84.9542	1,211.3	6/5/1979	12/14/1995	173	0.396	0.340	177,198	203,183	13,842
173051	RED CEDAR R @ CTH Y BRIDGE	7050007	WI	44.71927	-91.9034	4,852.4	2/10/1977	8/5/1999	27	0.138	0.120	192,414	140,259	10,703
173208	Red Cedar R. at Menomonie	7050007	WI	44.88336	-91.9319	4,627.8	8/4/1987	9/5/2007	175	0.119	0.100	170,311	129,496	6,336
173993	WABASH RIVER AT VINCENNES US 50 AT MI PT 129.8	5120111	IN	38.70722	-87.5192	35,578.4	2/9/1971	11/29/1995	245	0.242	0.180	3,229,014	2,522,099	171,066
174000	WABASH R NR HUNTINGTON IND AT MILE POINT 409.05	5120101	IN	40.85479	-85.4983	1,993.2	1/12/1971	12/12/1995	251	0.242	0.190	188,782	251,405	11,526
174001	WHITE RIVER NEAR HAZELTON.	5120202	IN	38.49083	-87.5653	29,382.0	2/16/1971	11/29/1995	29	0.269	0.190	3,278,249	2,305,980	755,904
174035	WHITE R AT PERKINSVILLE MILE POINT 278.610	5120201	IN	40.14167	-85.88	1,889.5	3/11/1971	12/14/1995	269	0.601	0.380	375,229	137,322	35,454
174303	BLUE R ON INTERSTATE 70 AT MILE POINT 63.76	5120204	IN	39.85401	-85.4773	242.3	3/24/1971	12/13/1995	104	0.369	0.180	20,290	20,632	3,929
174317	WHITE RIVER AT EDWARDSPORT, MILE POINT 80.98	5120202	IN	38.80696	-87.2459	12,931.6	2/8/1971	11/29/1995	267	0.454	0.290	1,839,890	1,143,928	111,224
174319	WHITE RIVER AT SPENCER MILE POINT 162.22	5120202	IN	39.28778	-86.7458	7,751.8	2/16/1971	11/29/1995	243	0.596	0.420	1,264,214	827,235	76,538
174350	TIPPECANOE R-ROCHESTER US 31 N OF TOWN MI PT 107	5120106	IN	41.10603	-86.2204	1,485.7	1/13/1986	12/21/1995	106	0.230	0.120	102,707	68,534	12,709
174554	WHITE R-W FORK-WAVERLY S.R. 144 BR MI PT 210	5120201	IN	39.55972	-86.2744	5,275.6	1/21/1986	11/30/1995	113	0.771	0.440	863,079	536,885	79,041
1BBFW0001	BEECH FORK LAKE	5090102	WV	38.29936	-82.3967	146.3	8/17/1973	8/2/1995	46	0.031	0.020			
1DEWW0001	JOHNS CR OF LEVISA FORK KY	5070203	KY	37.73908	-82.7313	4,733.8	5/28/1974	7/30/1996	56	0.053	0.026			
1DILW0003	LICKING CK AT TOBOSO GAGING STA	5040006	OH	40.05694	-82.22	1,743.9	8/29/1973	8/27/1996	51	0.128	0.060			
1ELTW0031	East Fork Twelvepole Ck	5090102	WV	38.05222	-82.3081	127.9	6/15/1976	11/21/1997	74	0.027	0.010	1,619	1,685	789
1ELTW0032	Kiah Creek	5090102	WV	38.06098	-82.266	75.2	5/16/1977	11/21/1997	72	0.028	0.010			
1FRLW0001	LEVISA FORK KY	5070202	KY	37.42494	-82.4121	1,019.3	6/25/1974	3/29/1994	82	0.036	0.015	20,900	16,517	9,876
1JWFW0001	POUND RIVER VA	5070202	VA	37.23734	-82.3433	574.7	6/26/1974	12/12/1995	75	0.018	0.010			
1JWFW0002	POUND RIVER VA	5070202	VA	37.16594	-82.5233	215.4	6/26/1974	12/13/1995	73	0.106	0.020			
1JWFW0003	CRANES NEST R OF POUND RIVER VA	5070202	VA	37.12397	-82.4385	179.7	6/25/1974	12/12/1995	74	0.030	0.010			
1PCSW0002	Paint Creek, Main Inflow to Lake	5060003	OH	39.32166	-83.3866	706.7	7/26/1973	12/16/1996	61	0.415	0.230	52,764	45,482	9,664
1PESW0003	STILLWATER CREEK ABOVE POOL	5040001	OH	40.10192	-81.1461	130.0	6/14/1983	10/14/1996	44	0.054	0.031			
1PESW0008	STILLWATER CREEK	5040001	OH	40.10259	-81.1311	130.0	5/14/1987	11/6/1997	45	0.044	0.020	3,031	5,889	747
1RDBW0012	Guyandot River	5070101	WV	37.61083	-81.7342	1,291.5	1/29/1980	12/29/1997	53	0.032	0.020			
1RDBW0015	Clear Fork of Guyandot River	5070101	WV	37.60917	-81.7242	333.5	1/29/1980	12/29/1997	83	0.021	0.010			
1SUMW0004	MUDDLETY CREEK OF GAULEY RIVER	5050005	WV	38.32675	-80.8326	128.0	10/11/1979	5/27/1998	31	0.018	0.010			
1SUTW0008	RIGHT FK HOLLY RIVER WV	5050007	WV	38.63576	-80.4663	142.2	6/2/1976	5/27/1998	61	0.022	0.010			
230028	GRAND RIVER AT WEBSTER RD BR; DELTA TWP SEC 3	4050004	MI	42.76111	-84.6496	3,251.8	4/3/1973	3/30/1994	337	0.197	0.156	153,602	148,604	5,192
233001	Sugar R. at Broadhead	7090004	WI	42.55023	-89.3663	1,551.8	12/8/1970	9/18/2007	148	0.188	0.174	75,167	99,054	4,586
243020	Fox R. at Berlin	4030201	WI	43.96667	-88.9502	3,426.7	1/20/1977	7/10/2007	204	0.085	0.072	83,056	70,829	4,921
250098	FLINT R AT CARPENTER RD BR; CITY OF FLINT	4080204	MI	43.07639	-83.6542	1,591.4	9/18/1973	9/23/1996	233	0.066	0.060	28,892	41,584	1,245
260001	Cuivre River	7110008	MO	38.92417	-90.7489	3,177.5	5/3/1993	11/8/2005	201	0.160	0.116	487,025	421,745	83,672
263001	MONTREAL RIVER NEAR SAXON, WI	4010302	WI	46.55658	-90.4141	689.3	10/21/1970	3/19/1998	50	0.062	0.050	11,076	15,072	629
273038	BLACK RIVER AT BYPASS CHANNEL @ K	7040007	WI	44.38942	-90.7601	3,299.2	1/13/1994	6/5/2001	77	0.112	0.100	136,285	169,956	17,418
303066	Fox (IL) R. near New Munster	7120006	WI	42.61085	-88.2254	2,040.9	3/13/1997	7/30/2007	42	0.116	0.106	59,935	139,471	4,316
313038	KEWAUNEE RIVER NEAR KEWAUNEE, WI	4030102	WI	44.45745	-87.5557	343.4	1/24/1977	9/18/2007	246	0.137	0.100	16,075	25,134	1,793
320540	Iowa River HWY 92 BR AT COLUMBUS JUCTION	7080209	IA	41.27885	-91.3447	31,516.5	9/22/1976	8/2/2006	219	0.350	0.300	2,463,978	2,664,648	119,258
324015	CEDAR RIVER AT LEWIS ACCESS, 6.5 MI N OF PALO	7080205	IA	42.16135	-91.8107	15,903.7	5/12/1971	12/16/1998	519	0.403	0.300	942,259	1,135,494	61,394
343033	Wolf R. at Langlade	4030202	WI	45.18998	-88.7337	1,204.9	1/25/1977	9/20/2007	101	0.031	0.028	12,527	13,041	827
353068	Wisconsin R. at Merril	7070002	WI	45.17822	-89.6862	7,122.0	1/25/1977	9/20/2007	248	0.056	0.051	121,160	108,747	4,406
373001	WISCONSIN RIVER AT WAUSAU	7070002	WI	44.95773	-89.6358	7,829.1	11/19/1970	6/19/2001	264	0.068	0.060	168,275	165,168	8,533
373325	BIG EAU PLEINE RIVER AT STRATFORD, WI	7070002	WI	44.82076	-90.0788	632.0	2/2/1976	9/12/2007	91	0.232	0.220	90,697	53,883	12,493
380031x	GRAND R AT MAPLE GROVE RD; RIVES TWP, SEC 35	4050004	MI	42.34167	-84.4016	932.4	8/30/1971	9/29/1982	125	0.165	0.122	37,858	28,732	1,682
380153	SHEYENNE RIVER - BALHILL DAM	9020204	ND	47.02735	-98.0862	9,847.5	4/20/1994	11/7/2006	148	0.276	0.262	44,380	71,962	2,386
3822350602001	HOCKING R. DST LANCASTER - 1ST U.S. RT 33 BRIDGE	5030204	OH	39.68595	-82.5738	179.7	7/10/1974	1/8/1997	266	0.664	0.525	28,271	12,794	2,104
383001	PESHTIGO RIVER AT PESHTIGO, WI	4030105	WI	45.05381	-87.7471	2,794.6	12/9/1970	8/8/2007	294	0.036	0.030	18,779	39,316	1,731
390057	KALAMAZOO R AT "G" AVE BRIDGE; CITY OF AUGUSTA	4050003	MI	42.33528	-85.3453	2,356.1	7/27/1971	3/1/1994	213	0.096	0.088	67,389	91,368	2,148
390553	South Skunk River CO RD BR E57	7080105	IA	41.97322	-93.5806	1,480.0	11/8/1979	11/9/1998	67	1.385	0.460	135,956	183,894	30,534
390566	SOUTH SKUNK RIVER APPROX 3.5 MI NNW OF CAMBRIDGE	7080105	IA	41.95035	-93.5511	1,523.8	10/8/1991	9/12/2007	185	0.793	0.400	128,169	185,076	12,880
391732085414401	CLIFTY CREEK AT CO RD 1150 E NEAR HARTSVILLE, IN	5120206	IN	39.29227	-85.6955	227.7	3/26/1993	8/10/2004	33	0.211	0.070			
394340085524601	SUGAR CREEK AT CO RD 400 S AT NEW PALESTINE, IN	5120204	IN	39.72782	-85.8794	239.8	5/7/1992	8/30/2005	271	0.086	0.060	13,564	12,430	1,090
40007	Cannon River	7040002	MN	44.52437	-92.5628	3,835.2	10/1/1991	11/8/2005	251	0.206	0.161	212,965	262,802	18,194
410050	GRAND RIVER AT KNAPP STREET; ADA TWP, SEC 7	4050006	MI	43.00611	-85.5425	11,577.7	8/30/1971	3/2/1994	225	0.093	0.091	334,229	461,681	9,992
410052	GRAND R AT M-11 BRIDGE; CITY OF GRANDVILLE	4050006	MI	42.91524	-85.7667	12,790.2	8/30/1971	3/2/1994	261	0.141	0.120	494,823	577,501	17,430
413002	MILWAUKEE RIVER @ BROWN DEER ROAD	4040003	WI	43.17769	-87.9561	1,632.4	12/16/1970	4/18/2001	44	0.283	0.225			
423014	North Raccoon River CO RD BR D46 SEC 13/24 AT SAC	7100006	IA	42.33971	-94.9859	1,834.5	10/9/1979	9/10/2007	344	0.501	0.330	192,224	213,035	13,525

426046	South River BRIDGE HIWAY 92	7100008	IA	41.37214	-93.4309	1,230.8	11/25/1991	9/6/2007	82	0.279	0.135	154,632	88,409	35,593
430380	Des Moines River CO RD E26 NW OF BOONE	7100004	IA	42.08029	-93.9382	14,065.6								
433002	OCONTO RIVER NEAR OCONTO, WI	4030104	WI	44.88271	-87.8843	2,504.5	10/26/1988	7/11/2007	47	0.052	0.040	30,119	56,626	3,227
443001	WISCONSIN RIVER AT RHINELANDER	7070001	WI	45.64094	-89.4205	2,221.8	4/17/1974	12/11/2001	115	0.041	0.040	21,175	14,299	1,110
443002	WISCONSIN R AT HAT RAPIDS DAM	7070001	WI	45.5742	-89.4789	2,929.2	4/17/1974	6/30/1999	210	0.052	0.044	34,437	34,340	2,276
443003	WISCONSIN RIVER BRIDGE AT MCNAUGHTON	7070001	WI	45.73376	-89.5133	2,015.8	3/27/1979	11/29/2001	174	0.033	0.030	16,050	13,509	897
443015	East Fk Des Moines River HWY 169 BR 1 MI N ST JOSE	7100003	IA	42.93537	-94.2271	2,439.4	7/7/1987	12/7/1998	136	0.356	0.300	150,994	219,531	14,626
443704	Wapsipinicon River CO RD BR 0.5 MI W. OF D16 N. OF	7080102	IA	42.51197	-91.9746	2,386.1	10/30/1979	9/10/2007	126	0.153	0.120	132,255	129,587	13,615
444066	Des Moines River CO RD BR P59 AT KALO S. OF FT DOD	7100004	IA	42.43159	-94.1325	10,842.0	11/16/1979	9/10/2007	111	0.240	0.200	567,152	852,158	46,482
46021	DUPAGE R OLD RT 6 S CHANNAHON T34N R9E NW20	7120004	IL	41.41944	-88.2278	954.2	10/6/1970	2/10/1984	54	1.358	1.200	430,050	188,179	50,625
47018	W BR DUPAGE R 2 MI S NAPERVILLE T37N R10E NE6	7120004	IL	41.72	-88.1322	319.9	10/15/1970	12/3/1997	66	1.725	1.400	155,607	70,964	7,927
47041	HICKORY CR 1 MI NE NEW LENOX T35N R11E NE14	7120004	IL	41.51611	-87.973	198.8	10/7/1970	12/3/1997	53	0.561	0.490	20,411	19,093	2,823
47087	Kaskaskia River RT 133 BRIDGE IN CHESTERVILLE.	7140201	IL	39.70266	-88.3871	931.1	10/21/1971	12/16/1997	27	0.332	0.240	97,580	66,801	19,589
48046	FOX R AT WEDRON T34N R4E NE9	7120007	IL	41.4319	-88.7726	6,602.5	8/30/1971	8/30/1996	56	0.429	0.400	686,368	498,242	53,199
48246	SALT CR YORK RD HINSDALE T39N R11E SE36	7120004	IL	41.82083	-87.9271	306.0	10/20/1970	10/2/1995	54	1.983	1.600	162,230	66,290	7,748
48247	SALT CR RT 51 ELMHURST T39N R11E SW14	7120004	IL	41.86592	-87.9517	253.7	7/2/1971	10/2/1995	51	2.013	1.566	138,599	64,037	8,257
48278	S BR KISHWAUKEE R 1.5 MI W SYCAMORE T41NR4ESE25	7090006	IL	41.99662	-88.7222	240.5	12/14/1971	11/13/1997	57	1.745	0.800	50,467	31,807	3,898
483026	MISS R.LOCK+DAM # 2 HASTINGS,MN.	7010206	MN	44.76058	-92.8674	90,233.2	7/19/1971	9/15/1994	305	0.299	0.260	4,217,109	5,695,605	153,384
483027	Mississippi R. Above LD 3	7040001	WI	44.61125	-92.611	109,835.0	10/6/1970	12/1/1998	300	0.197	0.190	3,254,815	6,086,895	119,451
48343	KICKAPOO CR 1 MI SSW CHARLESTON T12N R9E SW22	5120112	IL	39.46311	-88.1934	253.8	1/20/1972	9/10/1996	46	1.007	0.560	58,475	20,990	18,968
48414	KICKAPOO CR RT 136 BR 1 M W HEYWORTH T22NR2ESW32	7130009	IL	40.31209	-89.0144	306.9	4/19/1972	9/24/1997	47	0.340	0.110	18,446	25,358	6,204
48494	HURRICANE CR 4 MI WNW HERRIN T8SR1ESE16	7140106	IL	37.82277	-89.095	62.0	1/25/1972	2/29/1996	64	0.872	0.475	17,156	4,013	8,566
48525	PECATONICA R E EDGE WINSLOW T29N R6E SE22	7090003	IL	42.49312	-89.7897	2,676.1	1/7/1972	11/18/1998	52	0.278	0.245	138,355	255,043	59,355
4CRR11005	CHEAT RIVER RM 75.6	5020004	WV	39.12125	-79.6756	1,850.2	5/15/1973	3/12/1996	115	0.016	0.009	31,962	31,097	8,363
500233	CLINTON R@ NO. BOUND GRATIOT AVE IN MT. CLEMENS	4090003	MI	42.58417	-82.8828	1,951.2	1/17/1974	10/8/1998	342	0.235	0.190	118,277	155,049	4,222
501790	ROCKY R. AT LAKEWOOD - PARK BLVD. (RM 3.0)	4110001	OH	41.46944	-81.8317	746.3	11/19/1980	12/9/1998	229	0.261	0.150	87,383	64,597	12,929
501810	ROCKY R. AT FAIRVIEW PARK - PURITAS RD (RM 8.18)	4110001	OH	41.43778	-81.8456	728.6	5/22/1974	9/3/1997	32	0.855	0.800	127,752	63,674	44,967
502070	CUYAHOGA R AT SHALERSVILLE - DIAGONAL RD-SR 303	4110002	OH	41.23806	-81.3031	488.7	8/8/1973	12/10/1998	69	0.101	0.060	12,145	19,339	2,695
502080	L. CUYAHOGA R. AT AKRON - OTTO ST GAGE (RM 1.85)	4110002	OH	41.09444	-81.5217	160.5	8/8/1973	9/4/1996	63	0.234	0.160	15,903	7,400	3,714
502130	CUYAHOGA R. AT CLEVELAND - LOWER HARVARD AVE.	4110002	OH	41.44778	-81.685	2,032.8	10/23/1991	12/10/1998	82	0.344	0.250	264,052	320,539	24,497
503003	WISCONSIN RIVER ABOVE DUBAY DAM	7070002	WI	44.6649	-89.6505	12,483.3	8/17/1976	4/5/2001	195	0.097	0.080	315,893	407,145	24,642
503059	WISCONSIN RIVER ABOVE STEVENS POINT DAM	7070003	WI	44.51641	-89.5864	12,686.8	8/18/1976	6/19/2001	97	0.093	0.083	341,585	403,613	23,564
510017	MANISTEE R. AT HIGH BR RD; DICKSON TWP, SEC 33	4060103	MI	44.2675	-86.0139	3,621.4	4/8/1975	11/26/1990	77	0.019	0.015	28,024	32,891	5,253
523061	Root River	4040002	WI	42.78528	-87.8299	478.9	1/26/1977	9/18/2007	259	0.270	0.179	15,482	27,065	1,204
53210	Fox R. at DePere	4030204	WI	44.46111	-88.0591	16,171.5	10/17/1988	2/16/2005	173	0.126	0.103	448,908	393,152	25,100
543056	YAHARA R AT CTH M AT FULTON	7090001	WI	42.80806	-89.1255	1,231.4	7/18/1977	10/26/1997	72	1.357	1.175	289,459	38,046	23,485
550353	Tug Fork at Fort Gay, W.Va.	5070201	WV	38.11694	-82.5983	4,030.6	3/5/1985	1/20/2005	140	0.084	0.055	171,738	62,629	28,310
550446	Monongahela River below Morgantown, W. Va.	5020003	WV	39.65806	-79.9931	6,920.1	7/16/1974	2/17/2005	256	0.072	0.042	176,303	229,133	26,828
550476	Coal River at Tornado, W. Va.	5050009	WV	38.33889	-81.8408	2,223.3	11/13/1973	1/7/2005	252	0.045	0.029	29,524	26,766	4,503
550500	New River at C&O Railroad Bridge	5050004	WV	38.15092	-81.1799	17,993.3	11/19/1973	1/10/2005	250	0.061	0.046	311,302	538,070	32,522
550544	Elk River at Coonskin Park, above Charleston, WV	5050007	WV	38.38531	-81.586	3,917.8	11/13/1973	1/5/2005	211	0.044	0.030	82,883	79,164	11,750
573052	Wisconsin R. at Wisconsin Del	7070003	WI	43.62734	-89.7808	20,668.3	2/28/1977	9/17/2007	245	0.078	0.080	433,567	1,036,639	11,904
573081	BARABOO RIVER 1994 MILWAUKEE BRIDGE	7070004	WI	43.47032	-89.7689	1,433.7	9/23/1997	12/3/2001	53	0.210	0.217	71,713	81,263	5,146
573082	BARABOO RIVER AT HWY 113	7070004	WI	43.45833	-89.7165	1,455.5	9/23/1997	12/3/2001	53	0.208	0.209	70,081	83,969	4,973
580046	RAISIN R. AT ERA DOCK NEAR MOUTH; CITY OF MONROE	4100002	MI	41.90056	-83.3544	2,743.0	10/12/1970	10/8/1998	281	0.177	0.133	39,377	116,046	3,688
600150	GREAT MIAMI R. AT MIAMISBURG - S.R. 725	5080002	OH	39.64444	-84.2897	7,015.9	7/28/1994	12/17/1998	38	0.587	0.460	1,029,081	753,083	325,539
600810	SCIOTO R. AT SHADEVILLE - S.R. 665	5060001	OH	39.8325	-83.0083	4,387.8	1/4/1971	10/28/1996	901	1.407	1.100	990,851	506,806	27,821
600850	SCIOTO R. AT COLUMBUS - MOUND ST.	5060001	OH	39.95222	-83.0125	4,183.1	1/4/1971	6/3/1996	577	0.419	0.300	643,934	386,623	31,483
600870	SCIOTO R. AT COLUMBUS - FRANK RD.	5060001	OH	39.91667	-83.0097	4,205.8	1/4/1971	10/15/1996	651	0.365	0.300	668,263	387,647	34,169
600880	SCIOTO R. AT COLUMBUS - I-270 S.	5060001	OH	39.88062	-83.0179	4,232.5	1/4/1971	10/28/1996	920	1.459	1.100	949,243	503,708	23,748
600900	SCIOTO R. NR COMMERCIAL POINT - S.R. 762	5060001	OH	39.77417	-83.0075	5,879.7	1/4/1971	10/28/1996	943	1.300	1.025	1,356,721	710,542	39,571
600910	SCIOTO R. NR SOUTH BLOOMFIELD - S.R. 316	5060001	OH	39.71944	-83.0125	5,987.4	1/4/1971	10/7/1996	846	1.272	1.000	1,518,460	714,732	49,846
600920	SCIOTO R. DST CHILLICOTHE - U.S. RT. 35	5060002	OH	39.30556	-82.9203	10,010.7	6/8/1973	9/23/1997	222	0.535	0.460	1,573,257	995,449	95,212
600960	SCIOTO R. AT CIRCLEVILLE - U.S. RT. 22	5060002	OH	39.60139	-82.9553	8,331.7	1/22/1976	9/16/1996	82	0.689	0.500	1,253,957	870,411	179,615
601000	SCIOTO R. AT PIKETON - U.S. RT. 23 (RM 34.50)	5060002	OH	39.07222	-83.0056	15,114.5	1/4/1973	9/23/1997	273	0.308	0.220	1,169,174	1,227,124	37,551
601010	SCIOTO R. AT JASPER - S.R. 124 (RM 30.08)	5060002	OH	39.04716	-83.05	15,135.1	1/4/1973	9/23/1997	269	0.289	0.220	1,099,036	1,233,078	32,222
601290	OLENTANGY R. NR WORTHINGTON - I-270 N.	5060001	OH	40.11091	-83.0329	1,284.0	2/4/1976	1/23/1997	114	0.188	0.157	83,610	101,586	9,783
601340	SCIOTO R. UPST CIRCLEVILLE - FLORENCE CHAPEL RD.	5060001	OH	39.63278	-82.9625	6,830.3	10/23/1975	1/8/1997	255	0.670	0.470	876,946	766,235	49,814
601350	MILL CREEK OFF WALDO ROAD - MARYSVILLE (RM 16.8)	5060001	OH	40.25583	-83.3453	235.4	5/17/1977	1/23/1997	237	1.777	0.940	34,304	46,722	2,778
601400	RACCOON CREEK AT ADAMSVILLE - U.S. RT. 35	5090101	OH	38.87361	-82.3561	1,518.7	10/25/1977	12/14/1998	129	0.072	0.050	32,906	52,403	6,577

601930	TUSCARAWAS R. AT MASSILLON - WARMINGTON ST.	5040001	OH	40.75222	-81.5292	1,343.7	3/31/1976	12/22/1998	269	0.471	0.400	161,268	168,634	7,094
601940	NIMISHILLEN CRK DST N. INDUSTRY - HOWENSTINE RD.	5040001	OH	40.71718	-81.3473	463.2	4/2/1976	12/22/1998	262	0.788	0.660	137,352	137,054	5,556
602280	MAHONING R. AT LEAVITTSBURG - LEAVITT RD.	5030103	OH	41.23944	-80.8811	1,487.5	1/8/1971	12/22/1998	402	0.167	0.090	28,686	72,614	3,482
603095	Sheboygan R. at Esslingen Par	4030101	WI	43.74053	-87.7512	1,103.7	2/23/1977	9/10/2007	321	0.197	0.160	46,029	78,662	2,604
610060	GREAT MIAMI R. AT DAYTON - MONUMENT AVE.	5080002	OH	39.76334	-84.2021	6,491.1	10/12/1978	12/17/1998	233	0.266	0.240	612,828	527,885	37,778
610520	L. MIAMI R. NR MORROW - STUBBS MILL RD.	5090202	OH	39.3634	-84.1735	2,498.5	4/7/1976	9/1/1998	72	0.410	0.320	272,859	215,185	36,419
610530	E FK L MIAMI R NR MILFORD - NR TERRACE PARK C.C.	5090202	OH	39.15526	-84.2898	1,292.6	2/28/1978	9/8/1998	62	0.460	0.330	165,304	137,900	23,936
611740	MUSKINGUM R. DST COSHOCTON - S.R. 83	5040004	OH	40.23547	-81.8713	12,562.8	4/7/1976	12/16/1998	213	0.197	0.140	882,084	1,093,357	57,952
611790	TUSCARAWAS R. AT NEWCOMERSTOWN - RIVER ST.	5040001	OH	40.26139	-81.6092	6,322.3	10/28/1977	12/16/1998	239	0.203	0.150	473,561	541,810	26,865
63029	Mississippi R. Above LD 4	7040003	WI	44.32495	-91.9227	141,590.8	1/24/1977	12/1/1998	265	0.142	0.126	4,123,077	6,876,536	88,569
643509	Wis R Tailwater - Otter RPS Dam	7070001	WI	45.90361	-89.3214	1,427.1	8/10/1999	11/29/2001	29	0.039	0.037	14,632	9,758	987
683096	Fox (IL) R. below Waukesha	7120006	WI	42.93375	-88.2928	475.2	1/26/1977	1/30/2007	221	0.214	0.180	16,872	117,656	1,066
693035	Wolf R. at New London	4030202	WI	44.3917	-88.7496	6,123.2	1/19/1977	9/5/2007	302	0.075	0.065	129,575	177,282	6,357
6ACNR009.17	Cranes Nest River N.E. FROM ROUTE 83	5070202	VA	37.15083	-82.4108	198.6	4/13/1982	11/2/1998	124	0.107	0.100	5,276	2,390	1,111
6ADIS001.24	Dismal Creek, RT. 666 BRIDGE	5070202	VA	37.24278	-82.0275	223.0	7/21/1979	12/14/1998	150	0.082	0.100	2,862	4,058	777
6AKOX008.11	Knox Creek RT. 697 AT STATE LINE	5070201	KY	37.47064	-82.0624	228.9	7/31/1979	12/14/1998	148	0.090	0.100	6,336	3,272	1,330
6ALEV131.52	Levisa Fork WELLMORE COAL CO.DOCK #14 BR OFF 460 .	5070202	VA	37.34833	-82.2056	770.1	6/17/1992	12/14/1998	73	0.022	0.020	10,310	13,593	2,369
6ALEV143.86	Levisa Fork TEEL BR.ON RAILROAD AVE OFF RT 83, .1	5070202	VA	37.27694	-82.1008	505.9	6/17/1992	12/14/1998	73	0.024	0.020	6,122	8,933	1,388
6AMCR007.46	McClure River RT. 63 BR N OF CLINCH	5070202	VA	37.16833	-82.361	251.0	8/29/1979	12/7/1998	150	0.092	0.100	4,561	3,597	936
6ARSS026.98	Russell Fork UPSTREAM OF LAZARUS BR & DOWNSTR RT 7	5070202	VA	37.1925	-82.2878	389.9	5/14/1992	12/7/1998	42	0.018	0.020	3,037	6,130	1,054
6ASAT000.03	Slate Creek RT. 460 BRIDGE IN GRUNDY	5070202	VA	37.27889	-82.0994	106.2	7/31/1979	9/11/1986	75	0.087	0.100			
70005	BLACK RIVER NEAR GALESVILLE, WI	7040007	WI	43.96192	-91.3371	5,868.2	5/6/1993	11/9/2005	240	0.147	0.130	328,883	222,994	22,016
713001	FOX RIVER AT OMRO	4030201	WI	44.04014	-88.7446	3,829.4	12/15/1970	2/13/1996	103	0.113	0.100	135,490	87,016	7,857
713002	Fox R. at Neenah and Menasha	4030203	WI	44.18647	-88.4565	15,567.1	7/17/1986	9/5/2007	269	0.086	0.074	351,236	242,713	17,641
723002	Wisconsin R. at Biron	7070003	WI	44.43405	-89.7764	13,944.2	8/17/1976	9/19/2007	273	0.105	0.100	365,244	467,860	13,071
723020	WISCONSIN RIVER AT STH 73 IN NEKOOSA	7070003	WI	44.31438	-89.8884	14,230.6	2/8/1978	4/5/2001	98	0.134	0.120	467,337	563,903	29,958
730023	SHIAWASSEE R@ FERGUS RD; ST. CHARLES TWP SEC 22	4080203	MI	43.25472	-84.1056	1,576.2	8/31/1971	3/8/1994	72	0.118	0.082	54,605	47,146	5,663
730150	SAGINAW R OFF CENTER ST BR; SAGINAW TWP, SEC 35	4080206	MI	43.40222	-83.9661	15,322.4	1/29/1974	6/18/1996	96	0.165	0.142	384,704	423,842	22,759
740144	BLACK R AT 10TH AVE. BRIDGE; CITY OF PORT HURON	4090001	MI	42.98083	-82.4336	1,836.3	5/21/1973	6/18/1978	56	0.097	0.076	106,279	67,593	12,078
740153	BLACK R AT WADHAMS RD BRIDGE; KIMBALL TWP, SEC 2	4090001	MI	42.98981	-82.5378	1,759.8	5/22/1973	3/29/1994	260	0.100	0.078	103,424	65,655	5,551
784016	ENGLISH RIVER AT BRIDGE CO RD W61 AT RIVERSIDE	7080209	IA	41.47548	-91.5802	1,626.8	7/9/1987	9/11/2007	330	0.394	0.200	220,047	225,881	19,155
80001	LaCrosse River	7040006	WI	43.81882	-91.2535	1,234.4	10/4/1999	9/18/2007	125	0.170	0.150	48,353	42,345	2,208
810006	SALINE R AT MAPLE RD BRIDGE; SALINE TWP, SEC 13	4100002	MI	42.13	-83.7756	247.6	9/1/1971	10/8/1980	40	0.318	0.170	14,445	9,033	3,265
810042	HURON R AT SUPERIOR RD BR; SUPERIOR TWP, SEC 32	4090005	MI	42.25954	-83.6394	2,060.6	9/1/1971	3/30/1994	262	0.093	0.080	40,209	74,535	1,408
810242	HURON R AT HURON BR PARK; ANN ARBOR TWP., SEC 17	4090005	MI	42.30333	-83.7467	1,892.3	4/16/1975	3/30/1994	224	0.030	0.029	14,328	55,789	717
821004	CEDAR CREEK AT BRIDGE 3 MI WNW OF OAKLAND MILLS	7080107	IA	40.94456	-91.6783	1,383.1	10/20/1986	12/1/1998	137	0.329	0.200	111,964	162,434	13,894
9-CRK003.00	Crooked Creek, RT. 635 BRIDGE AT INTERSECTION WITH	5050001	VA	36.76861	-80.9078	185.7	6/18/1992	11/30/1998	38	0.044	0.030	5,504	6,624	1,445
9-EKC003.78	AT MT CARMEL CHURCH ON RT 650, 1.8 MI OFF RT 660	5050001	VA	36.69806	-81.0575	164.4	5/21/1992	12/17/1998	40	0.072	0.060	8,812	5,922	2,657
9-FXC000.84	LOW WATER BRIDGE OFF RT 711,APPROX .75 MI W. FOX	5050001	VA	36.61444	-81.3056	197.9	5/21/1992	11/30/1998	38	0.027	0.020	3,136	4,062	804
9-LRV000.34	Little River RT. 605 BRIDGE, S OF RADFORD	5050001	VA	37.07861	-80.5753	910.7	9/25/1997	11/4/1998	6	0.100	0.100			
9-NEW081.72	New RIver RT. 11 BRIDGE AT RADFORD	5050001	VA	37.13861	-80.575	7,151.9	7/25/1979	12/8/1998	216	0.105	0.100	307,115	250,765	7,341
9-NEW107.51	New River STA #25 ALLSONIA AT GAGE (PULASKI COUNTY	5050001	VA	36.93639	-80.7461	5,726.1	11/29/1993	12/7/1998	57	0.041	0.030	179,648	193,974	31,452
9-NEW187.46	New River OFF RT. 58 AT MOUTH OF WILSON OR RT. 93	5050001	VA	36.58531	-81.3142	1,744.4	7/11/1979	12/17/1998	197	0.086	0.100	67,492	45,553	7,432
9-WFC016.45	Wolf Creek at PRIVATELY OWNED LOW WATER BR OFF RT	5050002	VA	37.25641	-81.0143	505.6	6/15/1992	11/3/1998	32	0.020	0.020	4,148	10,253	1,221
9-WLK004.34	Walker Creek RT. 622 BRIDGE, BELOW PEARISBURG	5050002	VA	37.29651	-80.7063	791.9	8/25/1997	11/17/1998	6	0.100	0.100			
9-WLS002.57	Wilscon Creek, RT 721 BRIDGE OFF RT 56/16, APPROX	5050001	VA	36.59972	-81.3547	75.6	6/10/1992	11/30/1998	38	0.029	0.020	1,040	2,025	285
90003	Upper Iowa River	7060002	IA	43.47444	-91.2944	2,568.5	10/26/1979	9/20/2004	387	0.174	0.127	135,285	240,215	16,354
93001	Chippewa R. at Chippewa Falls	7050005	WI	44.93001	-91.397	14,346.7	10/6/1970	1/11/2005	136	0.051	0.048	243,751	253,811	11,468
AR01	Alllegheny River at Aspinwall, PA (rmi -7.4)	5010009	PA	40.48684	-79.9049	30,097.8	1/19/1995	7/5/2006	69	0.085	0.050	1,315,839	863,318	361,602
ATH 02	S Fork Saline R.	5140204	IL	37.67973	-88.8037	212.7	4/11/1983	12/10/1998	140	0.101	0.060	16,671	8,782	2,435
CAS0479	CASSELMAN R. CROS. BY RIVER RD. AT USGS STA.	5020006	MD	39.70389	-79.14	165.2	3/26/1974	8/4/1998	149	0.053	0.034	5,135	3,175	749
GMW020-0001	Greens Fk	5080003	IN	39.77167	-85.1092	223.4	3/19/1997	9/23/2003	60	0.065	0.030			
GMW040-0005	Whitewater River	5080003	IN	39.49806	-85.1825	1,497.0	3/18/1997	3/1/2006	90	0.061	0.030	162,541	84,564	48,402
GMW080-0001	Whitewater River	5080003	IN	39.35333	-84.9428	3,418.3	2/13/1986	3/1/2006	136	0.067	0.040	166,396	221,803	30,412
K3250000	S FORK NEW RIV AT NC 16 AND 88 NR JEFFERSON	5050001	NC	36.39473	-81.4075	533.6	4/23/1981	8/22/2007	164	0.049	0.030	18,755	15,286	2,393
K4500000	S FORK NEW RIV AT NC 221 NR SCOTTVILLE	5050001	NC	36.4736	-81.3365	778.2	10/2/1974	9/26/2007	178	0.065	0.030	41,669	23,866	6,970
K7900000	NEW RIV AT SR 1345 AT AMELIA	5050001	NC	36.5519	-81.1817	2,141.8	10/18/1976	9/26/2007	236	0.073	0.045	87,303	57,291	8,740
LEJ050-0006	Fish Cr	4100003	IN	41.55861	-84.8356	105.7	4/6/1999	2/6/2006	70	0.069	0.060	3,046	7,551	321
LEJ050-0007	Fish Cr	4100003	IN	41.465	-84.8142	174.9	4/6/1999	2/6/2006	84	0.077	0.060	5,628	8,691	625
LEJ060-0006	ST Joseph River	4100003	IN	41.3475	-84.8439	1,571.2	4/6/1999	2/6/2006	76	0.127	0.110	87,017	68,773	7,350

LEJ090-0008	Cedar Cr	4100003	IN	41.21889	-85.0767	785.3	4/6/1999	7/9/2001	28	0.103	0.090	45,207	44,872	9,996
LEJ090-0026	Cedar Cr	4100003	IN	41.215	-85.0514	787.7	8/27/2001	1/23/2006	49	0.105	0.070	59,876	45,095	8,614
LEJ100-0003	ST Joseph River	4100003	IN	41.08917	-85.1292	2,808.5	11/15/1983	1/23/2006	239	0.158	0.130	177,582	138,301	11,790
LEM010-0012	Maumee River	4100005	IN	41.08194	-85.1147	4,855.0	2/18/1971	1/23/2006	178	0.236	0.200	405,136	417,022	32,410
LEM010-0013	Maumee River	4100005	IN	41.16906	-84.8493	5,223.8	3/24/1971	1/23/2006	373	0.282	0.230	440,489	486,335	25,569
LEM010-0014	Maumee River	4100005	IN	41.08444	-85.0206	4,962.3	1/13/1971	1/23/2006	407	0.347	0.230	376,046	473,992	26,914
LES040-0007	St. Mary's River	4100004	IN	40.77917	-84.8422	1,305.6	6/5/1979	2/7/2006	301	0.304	0.265	136,714	190,676	7,834
LES060-0004	ST Mary's River	4100004	IN	41.08389	-85.1358	2,042.1	1/7/1986	1/23/2006	219	0.283	0.250	242,452	278,456	15,064
LES060-0005	ST Mary's River	4100004	IN	40.99111	-85.1169	1,847.3	1/8/1991	1/23/2006	162	0.280	0.270	248,968	262,509	15,113
LMG050-0006	Salt Cr	4040001	IN	41.59972	-87.1464	192.5	1/14/1986	1/30/2006	229	0.138	0.130	11,707	12,351	611
LMG050-0007	Salt Cr	4040001	IN	41.49861	-87.1417	92.1	1/18/1973	9/4/2003	346	0.173	0.140	4,100	8,169	249
LMG060-0008	E Br Little Calumet River	4040001	IN	41.61694	-87.1261	179.7	2/23/1971	1/30/2006	395	0.142	0.100	6,035	9,383	396
LMJ120-0009	Pigeon River	4050001	IN	41.74	-85.5569	875.4	4/15/1999	2/8/2006	81	0.043	0.030	11,616	32,768	1,389
LMJ150-0004	ST Joseph River	4050001	IN	41.72278	-85.8147	6,387.9	2/10/1971	2/8/2006	375	0.088	0.050	125,376	239,671	10,022
LMJ190-0006	Elkhart River	4050001	IN	41.50778	-85.7594	867.9	4/14/1999	2/8/2006	78	0.066	0.055	23,779	38,060	2,284
LMJ210-0008	Elkhart River	4050001	IN	41.68778	-85.9722	1,792.2	1/19/1973	2/8/2006	376	0.130	0.080	53,170	97,225	3,234
LMJ240-0008	ST Joseph River	4050001	IN	41.74444	-86.2728	9,395.8	3/11/1971	12/19/2002	350	0.147	0.090	332,469	427,177	28,913
LMJ240-0009	ST Joseph River	4050001	IN	41.67111	-86.1536	9,151.7	2/18/1986	5/9/2002	179	0.080	0.050	147,541	399,606	13,862
LMJ240-0026	ST Joseph River	4050001	IN	41.66639	-86.1678	9,155.3	6/19/2002	2/8/2006	43	0.046	0.040	171,707	399,663	34,804
LRBF-.5-BB14A71	BIG FORK RIVER BRIDGE ON MN-11, 4 MI E OF LOMAN	9030006	MN	48.5125	-93.71	3,884.3	7/27/1971	9/9/1992	88	0.074	0.050	64,555	56,139	7,515
LRLF7.5-BB14A71	LITTLE FORK R MN-11 BRIDGE, 0.5 MI W OF PELLAND	9030005	MN	48.52132	-93.5866	4,718.2	7/27/1971	11/3/1994	82	0.090	0.064	124,277	57,231	13,321
LSSL-52-BC15E53	ST. LOUIS R BRIDGE ON US-2, 2 MI SE OF BROOKSTON	4010201	MN	46.84944	-92.5758	8,425.6	10/19/1970	11/8/1994	233	0.071	0.053	153,553	103,980	7,975
LYO0004	LIT. YOUGH. R. OLD FOOT BR. 0.4M. AB. MOUTH	5020006	MD	39.41831	-79.4192	105.7	3/27/1974	12/5/1995	144	0.147	0.102	7,864	2,878	885
M03P19	E FK TODD FORK AT CLARKSVILLE - SR 133/132	5090202	OH	39.39861	-83.9831	98.8	12/10/1981	9/10/1998	56	0.284	0.080			
MNCO-.5-B-14A67	COTTONWOOD R AT MN-15, 0.5 MI SE OF NEW ULM	7020008	MN	44.28237	-94.4356	3,372.6	10/6/1970	11/16/1998	219	0.223	0.160	168,911	263,491	23,937
MNMN-91-BB15E71	MINNESOTA RIVER AT BRIDGE ON MN-22 AT ST. PETER	7020007	MN	44.30742	-93.9596	36,896.9	7/20/1971	8/6/1998	246	0.319	0.284	1,355,647	2,811,275	98,268
MR01	Monongahela River near South Pittsburgh, PA	5020005	PA	40.41026	-79.953	19,070.8	7/6/1976	9/7/2004	324	0.118	0.080	770,239	630,122	95,758
MS216	PRAIRIE CR AT 310TH ST .2 MI UPSTM OF L BYLLESBY	7040002	MN	44.50056	-92.9925	196.5	9/7/1989	9/24/1998	53	0.092	0.061	6,152	10,003	1,426
MSCW-.2-B-14A53	CROW RIVER AT BRIDGE ON CSAH-36 AT DAYTON	7010203	MN	45.24471	-93.522	6,685.9	11/3/1970	11/11/1998	157	0.302	0.268	285,099	531,799	24,781
MSRM-.6-BB14A53	RUM RIVER AT BRIDGE ON PLEASANT STREET IN ANOKA	7010207	MN	45.2066	-93.3867	4,055.4	10/5/1970	8/6/1998	110	0.139	0.119	53,458	97,207	7,161
MSRM-34-BB14A55	RUM RIVER BRIDGE ON CSAH-5, 0.5 MI W OF ISANTI	7010207	MN	45.49333	-93.2669	3,314.8	6/7/1974	9/7/1994	200	0.136	0.115	75,574	82,746	3,436
MSU--68CBB15E53	MISSISSIPPI R UPSTREM OF MN-15 BR AT SAUK RAPIDS	7010201	MN	45.59813	-94.1879	30,255.0	9/21/1988	9/30/1996	57	0.087	0.071	379,315	467,189	47,572
MSU-859---01E53	MISSISSIPPI R MPLS WATERWORKS INTAKE AT FRIDLEY	7010206	MN	45.04917	-93.2792	47,566.3	10/5/1970	8/6/1998	220	0.118	0.111	718,770	1,391,815	55,780
MSU-982CBB15E67	MISSISSIPPI RIVER AT BR ON MN-115 AT CAMP RIPLEY	7010104	MN	46.07472	-94.3344	26,472.7	11/4/1970	7/21/1998	234	0.073	0.055	181,993	298,717	18,034
MSU1186-BB15E67	MISSISSIPPI R AT MN-6 BRIDGE 8 MI SW OF COHASSET	7010101	MN	47.22955	-93.7572	7,549.5	10/19/1970	9/16/1996	216	0.052	0.040	55,952	22,167	3,532
MSU1292-BB15E67	MISSISSIPPI R AT BR ON CSAH-8 7 MI E OF BEMIDJI	7010101	MN	47.45333	-94.7122	1,639.3	9/22/1987	9/16/1996	56	0.061	0.044	5,437	12,819	1,048
MU01	Muskingum River near Devola, OH	5040004	OH	39.47028	-81.4911	20,773.8	7/8/1976	7/11/2006	221	0.217	0.120	1,722,147	1,508,996	231,085
NF01	Ohio River near Stratton, OH	5030101	WV	40.52754	-80.6259	61,752.2	7/9/1992	7/13/2006	84	0.069	0.060	1,988,212	2,513,184	170,581
NF03	Great Miami River near Elizabethtown, OH	5080002	OH	39.15326	-84.7952	13,864.7	7/3/1973	7/13/2006	314	0.365	0.340	1,138,276	1,353,663	97,714
NF04	Green River near Quinn Landing, KY	5110005	KY	37.6473	-87.4956	21,392.3	10/11/1976	7/13/2006	302	0.129	0.090	1,109,262	1,914,023	118,347
OBS140-0004	Blue River	5140104	IN	38.43389	-86.1917	724.9	1/17/1973	3/2/2006	256	0.090	0.063	25,674	61,355	2,732
OBS150-0008	Blue River	5140104	IN	38.22056	-86.2983	1,286.6	4/7/1999	2/8/2006	83	0.092	0.070	56,707	91,933	8,617
OR15	Ohio River near Louisville, KY	5140101	KY	38.28244	-85.7039	235,672.3	10/12/1976	7/26/2006	302	0.206	0.110	23,379,918	12,731,093	2,432,471
OR21	Ohio River near Smithland, KY	5140203	KY	37.14613	-88.4116	371,652.1	1/19/1982	7/10/2006	156	0.197	0.100	29,835,507	24,208,157	4,326,031
P06S08	MAUMEE R. AT EATER RD. (RM 91.48)	4100005	OH	41.22194	-84.6697	5,469.3	7/24/1984	9/30/1997	55	0.203	0.160	507,049	495,935	51,552
PRI002	TUG FORK AT KERMIT, W. VA.	5070201	WV	37.838	-82.4096	3,309.5	2/19/1985	11/16/2006	202	0.045	0.026	70,641	52,196	15,938
PRI003	TUG FORK AT STATE LINE	5070201	WV	37.56611	-82.1439	2,127.2	3/27/1979	7/20/2006	104	0.045	0.035	27,987	34,078	5,490
PRI008	SOUTH FORK CUMBERLAND RIVER AT BLUE HERON	5130104	KY	36.67028	-84.5489	2,463.2	10/12/1983	6/21/2006	81	0.020	0.011	53,092	52,326	19,561
PRI010	ROCKCASTLE RIVER AT BILLOWS	5130102	KY	37.17139	-84.2967	1,558.2	3/20/1979	6/19/2006	268	0.021	0.013	18,452	55,638	3,580
PRI014	ROUGH RIVER NEAR DUNDEE	5110004	KY	37.56278	-86.7708	1,991.8	6/20/1979	12/12/2006	177	0.057	0.046	73,212	130,016	8,544
PRI021	NOLIN RIVER AT WHITE MILLS	5110001	KY	37.55502	-86.0319	919.4	3/25/1980	12/14/2006	273	0.151	0.110	65,853	117,504	6,515
PRI024	KENTUCKY RIVER AT FRANKFORT	5100205	KY	38.21286	-84.8726	14,045.3	3/13/1979	3/23/2006	276	0.102	0.075	658,525	789,762	59,084
PRI025	KENTUCKY RIVER AT CAMP NELSON	5100205	KY	37.76917	-84.6172	11,732.2	1/23/1980	2/20/1997	197	0.097	0.061	563,344	552,649	60,876
PRI030	POND CREEK NEAR LOUISVILLE	5140102	KY	38.11998	-85.796	166.9	1/18/1979	4/9/1998	221	0.935	0.638	50,465	28,866	5,999
PRI031	NORTH FORK KENTUCKY RIVER AT JACKSON	5100201	KY	37.55111	-83.3844	2,853.8	4/19/1984	9/19/2006	215	0.035	0.019	67,186	119,339	14,955
PRI034	SOUTH ELKHORN CREEK NEAR MIDWAY	5100205	KY	38.14139	-84.6453	272.0	4/12/1984	5/19/1999	175	1.225	0.933	145,666	121,826	11,891
PRI043	LITTLE RIVER NEAR CADIZ	5130205	KY	36.84056	-87.7775	697.5	10/15/1985	12/11/2006	207	0.140	0.110	38,445	78,240	4,724
PRI046	RED RIVER AT CLAY CITY	5100204	KY	37.86528	-83.9333	938.4	10/17/1985	9/18/2006	191	0.032	0.020	15,902	25,954	3,309
PRI048	TYGARTS CREEK NEAR LYNN	5090103	KY	38.59997	-82.9524	714.8	11/14/1985	9/12/2006	197	0.025	0.015	9,985	24,279	2,295
PRI049	LITTLE SANDY RIVER NEAR ARGILLITE	5090104	KY	38.49056	-82.8342	1,397.8	11/14/1985	11/14/2006	203	0.042	0.023	34,501	56,718	6,271

PRI051	HORSE LICK CREEK NEAR LAMERO	5130102	KY	37.32012	-84.1385	159.7	10/20/1992	6/19/2006	104	0.012	0.008	1,170	4,575	387
PRI052	SALT RIVER NEAR GLENSBORO	5140102	KY	38.00222	-85.0597	445.5	5/16/1989	12/19/2006	186	0.326	0.262	84,397	51,091	7,624
PRI055	GREEN RIVER NEAR LIVERMORE	5110003	KY	37.4782	-87.1269	15,743.0	7/10/1991	12/12/2006	136	0.067	0.044	608,460	1,412,708	65,339
PRI058	KENTUCKY RIVER NEAR TRAPP	5100204	KY	37.84667	-84.0811	8,383.3	1/16/1991	11/20/2006	139	0.024	0.016	197,339	263,223	41,392
PRI060	NORTH FORK LICKING RIVER NEAR MILFORD	5100101	KY	38.5812	-84.1656	738.5	6/11/1991	11/30/2006	136	0.139	0.095	75,171	94,056	11,385
PRI061	LICKING RIVER AT CLAYSVILLE	5100101	KY	38.52056	-84.1833	5,097.3	1/14/1991	11/30/2006	83	0.072	0.044	328,062	312,846	51,313
PRI064	LEVISA FORK AT LOUISA	5070203	KY	38.08056	-82.6003	6,001.1	6/26/1991	11/16/2006	128	0.029	0.024	130,985	88,958	26,455
PRI067	KENTUCKY RIVER AT HIGH BRIDGE	5100205	KY	37.81914	-84.7065	11,888.7	10/30/1997	11/20/2006	62	0.072	0.055	382,771	569,389	44,767
PRI069	RED RIVER NEAR KEYSBURG	5130206	TN	36.64081	-86.9791	1,226.3	6/10/1998	11/20/2006	60	0.060	0.046	46,004	147,452	6,831
PRI077	RUSSELL CREEK NEAR GRESHAM	5110001	KY	37.16781	-85.4702	685.4	6/8/1998	11/15/2006	54	0.055	0.035	28,961	71,763	8,467
PRI086	CUMBERLAND RIVER AT CALVIN	5130101	KY	36.72214	-83.6255	1,345.6	7/23/1998	12/13/2006	43	0.041	0.016			
PRI087	CLEAR FORK NEAR WILLIAMSBURG	5130101	KY	36.72589	-84.1424	918.6	7/8/1998	8/18/2004	37	0.018	0.015			
PRI092	GOOSE CREEK NEAR ONEIDA	5100203	KY	37.23703	-83.671	663.0	6/10/1998	9/19/2006	41	0.029	0.023	15,691	15,185	4,367
PRI100	FLOYDS FORK NEAR SHEPHERDSVILLE	5140102	KY	38.035	-85.6594	673.7	6/16/1998	12/19/2006	60	0.181	0.130	84,561	65,437	16,411
PRI102	HINKSTON CREEK AT RUDDLES MILLS	5100102	KY	38.30461	-84.2379	671.3	7/28/1998	11/30/2006	60	0.173	0.148	55,704	82,876	8,347
PRI105	BRASHEARS CREEK AT TAYLORSVILLE	5140102	KY	38.03722	-85.3406	672.8	8/11/1998	12/19/2006	54	0.281	0.213	196,088	102,249	33,180
PRI111	LICKING RIVER AT BUTLER	5100101	KY	38.78969	-84.3675	8,767.4	4/20/1999	11/30/2006	54	0.122	0.072	629,195	763,447	117,663
R12P02	KOKOSING R AT TILDEN AVE GAGE - MT. VERNON	5040003	OH	40.40556	-82.4997	522.3	7/16/1987	1/17/1997	81	0.060	0.050	10,015	30,929	1,317
RROT--1---10A53	OTTER TAIL R BRIDGE ON 4TH ST N AT BRECKENRIDGE	9020103	MN	46.26722	-96.5897	4,417.0	10/20/1970	11/2/1994	239	0.146	0.130	78,051	117,802	3,826
RRRR296---10E53	RED RIVER AT ALMONTE AVE S IN GRAND FORKS, ND	9020301	ND	47.90778	-97.0256	47,603.4	10/20/1970	11/10/1997	242	0.378	0.311	1,317,327	1,425,551	102,685
RRRR403---10E67	RED RIVER AT BRIDGE ON CSAH-39, 1 MI W OF PERLEY	9020107	MN	47.17979	-96.8247	35,883.6	10/20/1970	11/2/1994	237	0.515	0.450	865,139	1,070,188	75,544
RRRR452---10E71	RED RIVER BR ON MAIN AVE AT 3RD ST., IN MOORHEAD	9020104	ND	46.87397	-96.7763	14,792.9	7/26/1971	9/14/1998	245	0.211	0.180	313,006	539,306	28,724
S000-040	MINNESOTA RIVER AT MN-19 BRIDGE AT HENDERSON	7020012	MN	44.52942	-93.9008	38,613.0	10/6/1970	9/21/2006	278	0.323	0.270	1,669,509	2,906,750	100,542
S000-047	STRAIGHT R NEAR CSAH-1 1 MI SE OF CLINTON FALLS	7040002	MN	44.12414	-93.229	654.5	8/11/1977	9/7/2006	190	0.341	0.289	41,365	57,064	2,367
S000-054	MINNESOTA R CSAH-24 BRIDGE, 1 MI S OF COURTLAND	7020007	MN	44.25444	-94.3414	26,385.1	10/6/1970	9/21/2006	271	0.285	0.272	954,609	1,619,512	49,627
S000-055	MINNESOTA R BRIDGE ON CSAH-21, 3 MI NE OF DELHI	7020004	MN	44.6195	-95.1779	18,904.8	10/24/2000	9/14/2006	34	0.222	0.215	285,841	927,289	24,381
S000-056	ST. CROIX R AT MN-48 BR, 3.5 MI W OF DANBURY, WI	7030001	MN	46.01253	-92.444	5,917.6	10/23/1970	9/20/2007	250	0.058	0.040	61,124	57,472	6,192
S000-095	MISSISSIPPI R LOCK & DAM #6 AT TREMPEALEAU, WIS	7040006	WI	43.99828	-91.4391	149,360.1	10/7/1970	9/6/2006	189	0.192	0.178	4,710,711	7,324,315	196,936
S000-111	OTTER TAIL R BR ON CSAH-15 WEST OF FERGUS FALLS	9020103	MN	46.27542	-96.1344	4,032.5	10/18/1982	10/11/2006	85	0.075	0.064	35,223	61,601	1,874
S000-121	KETTLE R BRIDGE ON MN-48, 4.5 MI E OF HINCKLEY	7030003	MN	46.01086	-92.8398	2,556.8	10/23/1970	9/20/2007	268	0.068	0.058	43,517	50,748	3,080
S000-136	CEDAR RIVER AT CSAH-4, 3 MILES SOUTH OF AUSTIN	7080201	MN	43.60522	-92.9845	1,230.6	10/27/1970	9/7/2006	262	0.636	0.517	106,254	102,061	8,325
S000-156	W FK DES MOINES R CSAH-23 BRIDGE S OF PETERSBURG	7100002	MN	43.52617	-94.9192	3,291.0	10/14/1970	9/13/2006	200	0.320	0.280	187,464	245,396	10,696
S000-159	YELLOW MEDICINE R MN-67 BR 7 MI SE GRANITE FALLS	7020004	MN	44.72881	-95.4438	1,616.1	10/15/1970	9/14/2006	204	0.160	0.116	50,505	130,919	5,724
S000-186	TWO RIVERS ON US-75, 1 MI N OF HALLOCK	9020312	MN	48.78925	-96.9555	1,544.0	7/27/1971	10/12/2006	127	0.295	0.122	39,351	34,524	7,737
S000-268	ZUMBRO R S FORK AT CSAH-14, 3 MI N OF ROCHESTER	7040004	MN	44.108	-92.4475	871.4	6/4/1981	9/6/2006	166	0.690	0.374	34,668	72,670	2,698
S000-287	MISSISSIPPI R LOCK & DAM #5 3 MI SE OF MINNEISKA	7040003	MN	44.16011	-91.8116	146,351.0	5/21/1974	9/6/2006	220	0.179	0.170	4,320,441	7,226,460	191,431
S000-299	REDWOOD R AT BRIDGE ON CSAH-101 AT N REDWOOD	7020006	MN	44.56572	-95.0998	1,760.2	6/4/1974	9/14/2006	217	0.371	0.282	96,345	196,374	7,591
S001-131	LOST R AT CSAH-5 AT OKLEE	9020305	MN	47.84397	-95.8585	570.8	5/30/1984	7/9/2007	96	0.110	0.058	5,236	9,709	908
S002-088	THIEF R ON CSAH-7 BRG IN AGASSIZ NWR, 6 MI E OF HO	9020304	MN	48.30045	-96.0708	1,438.5	5/30/1984	9/11/2007	77	0.155	0.072	36,577	8,381	9,172
S002-118	CLEARWATER R AT KLONDIKE BRIDGE/BOTTINEAU AVE RED	9020305	MN	47.88607	-96.2769	3,155.6	2/6/1987	8/14/2007	179	0.118	0.075	56,172	51,183	6,184
S002-124	CLEARWATER R AT RD IN T151R42S45W 1 MI N PLUMMER	9020305	MN	47.9233	-96.0459	1,325.4	3/27/1991	9/4/2007	151	0.129	0.101	21,731	24,812	1,795
S002-283	MISSISSIPPI R AT FOREST ROUTE 2171, 9 MI NE OF CAS	7010101	MN	47.44445	-94.4252	2,762.7	6/29/1993	11/2/2004	29	0.032	0.018	11,604	17,659	1,728
S002-365	TWO RIVERS, S BR AT US-59 AT LAKE BRONSON	9020312	MN	48.7326	-96.6675	1,210.3	6/24/1991	10/22/2002	38	0.130	0.085	22,677	27,674	8,315
S002-963	RED LK R AT 2ND AVE NE (MURRAY BRIDGE) IN EAST GRA	9020303	MN	47.92372	-97.016	13,535.3	10/20/1970	8/14/2007	334	0.142	0.108	185,102	159,310	13,592
SL 191	ST. LOUIS RIVER AT SH-23 BRIDGE	4010201	MN	46.65882	-92.2842	9,027.3	10/23/1970	11/20/1996	487	0.059	0.050	132,439	116,466	4,422
SL 261	ST. LOUIS R.-N.THOMSON RESERVIOR	4010201	MN	46.66417	-92.4049	8,862.7	6/4/1974	11/20/1996	223	0.042	0.040	98,376	114,795	3,634
SL 303	ST. LOUIS R. AT SCANLON HYDO DAM	4010201	MN	46.70969	-92.4201	8,661.6	1/29/1974	11/20/1996	246	0.045	0.039	102,673	110,661	4,693
SL 500	ST. LOUIS RIVER AT COUNTY RD. 31	4010201	MN	46.86972	-92.6028	6,014.7	6/4/1974	11/20/1996	215	0.056	0.050	86,858	86,740	3,659
SR01	Scioto River at Lucasville, OH	5060002	OH	38.88111	-83.0175	15,984.7	9/7/1976	7/11/2006	251	0.335	0.260	1,051,628	1,260,216	114,954
UMI050-0006	Iroquois River	7120002	IN	40.82028	-87.4642	1,411.1	3/16/1999	2/15/2006	75	0.125	0.119	52,635	53,450	3,970
UMK030-0002	Kankakee River	7120001	IN	41.47722	-86.6044	966.5	2/21/1978	11/30/2000	248	0.098	0.050	18,306	74,898	2,065
UMK030-0020	Kankakee River	7120001	IN	41.46139	-86.6139	980.3	1/25/2001	2/15/2006	62	0.039	0.031	13,585	77,024	852
UMK060-0001	Yellow River	7120001	IN	41.3025	-86.6014	1,063.8	3/16/1999	2/15/2006	82	0.106	0.090	51,190	45,182	3,479
UMK080-0001	Kankakee River	7120001	IN	41.21974	-86.9687	3,441.1	3/16/1999	2/15/2006	81	0.076	0.060	111,361	156,302	5,819
UMK090-0001	Crooked Cr	7120001	IN	41.28222	-87.0258	224.8	3/16/1999	2/15/2006	78	0.050	0.040	4,560	4,894	586
V02P16	SCIOTO R. AT WARRENSBURG - OSTRANDER RD.	5060001	OH	40.30583	-83.1681	1,968.4	9/4/1979	10/2/1995	29	0.168	0.160	131,951	165,981	17,370
WAE050-0001	Eel River	5120104	IN	40.94806	-85.8911	1,284.0	1/16/1985	2/22/2006	239	0.181	0.120	124,995	75,967	10,219
WAW010-0063	Wildcat Cr	5120107	IN	40.48611	-86.1075	509.4	2/21/1974	2/14/2006	406	0.111	0.090	23,692	21,244	1,649
WAW020-0004	Wildcat Cr	5120107	IN	40.47361	-86.1842	646.7	1/25/1973	2/14/2006	443	0.312	0.170	54,170	44,307	4,810
WAW020-0039	Wildcat Cr	5120107	IN	40.48167	-86.53	981.2	9/23/1998	2/22/2006	89	0.170	0.150	58,218	70,261	5,304

WAW050-0005	Wildcat Cr	5120107	IN	40.45361	-86.8514	2,077.1	3/13/1980	2/22/2006	151	0.136	0.120	139,113	206,562	14,378
WBU100-0001	Wabash River	5120111	IN	39.22722	-87.5742	33,226.7	1/4/1978	2/1/2006	319	0.232	0.200	2,823,620	2,355,982	131,684
WBU160-0002	Busseron Cr	5120111	IN	38.97389	-87.4258	591.6	3/10/1999	11/12/2003	65	0.171	0.150	34,221	37,760	4,266
WBU200-0003	Wabash River	5120111	IN	38.68125	-87.5347	35,672.3	1/10/1990	2/13/2006	185	0.205	0.190	3,116,916	2,522,723	164,015
WDE010-0007	Wabash River	5120105	IN	40.73667	-86.505	9,981.3	2/1/1971	2/22/2006	349	0.290	0.180	1,067,974	840,646	86,195
WDE060-0001	Wabash River	5120105	IN	40.52861	-86.7603	11,496.6	2/21/2001	2/22/2006	61	0.216	0.180	1,153,958	954,380	88,943
WDE070-0006	Wabash River	5120105	IN	40.49556	-86.8233	16,696.8	1/22/1991	12/5/2000	112	0.168	0.145	1,383,464	1,154,712	95,377
WED010-0005	Big Blue River	5120204	IN	39.87361	-85.4389	149.4	1/24/1991	9/23/2003	95	0.147	0.130	11,208	17,557	1,194
WED050-0001	Big Blue River	5120204	IN	39.355	-85.9839	1,511.6	1/17/1973	2/9/2006	242	0.169	0.140	102,066	94,956	7,608
WEF050-0002	Flatrock River	5120205	IN	39.36361	-85.8553	1,056.8	4/15/1999	3/2/2006	84	0.063	0.050	61,476	77,272	12,825
WEF060-0003	E Fk White River	5120205	IN	39.20028	-85.9264	4,070.1	1/21/1986	2/8/2006	247	0.113	0.080	254,824	281,172	22,681
WEL040-0003	E Fk White River	5120208	IN	38.82583	-86.5133	10,459.6	2/13/1974	2/21/2006	356	0.138	0.100	876,777	758,900	56,984
WEL100-0002	E Fk White River	5120208	IN	38.80194	-86.645	12,221.4	2/16/1971	2/21/2006	398	0.153	0.100	709,317	820,994	46,979
WEL170-0001	E Fk White River	5120208	IN	38.53917	-87.2231	14,877.0	1/15/1980	2/28/2006	309	0.176	0.110	1,222,911	1,021,884	100,077
WEM070-0001	Vernon Fk Muscatatuck Riv	5120207	IN	38.97639	-85.62	516.4	7/9/1998	3/1/2006	106	0.165	0.125	30,811	57,221	3,385
WEU040-0001	E Fk White River	5120206	IN	38.98722	-85.8989	6,043.9	3/12/1971	2/9/2006	418	0.175	0.110	501,085	447,200	39,429
WLV030-0003	Wabash River	5120108	IN	40.41194	-87.0364	19,508.1	1/24/1973	2/2/2006	382	0.285	0.200	1,741,532	1,494,861	98,738
WLV080-0003	Wabash River	5120108	IN	40.255	-87.2997	21,085.4	3/15/1999	2/2/2006	81	0.221	0.220	1,743,644	1,557,835	78,158
WLV140-0001	Wabash River	5120108	IN	39.95167	-87.4192	25,311.8	2/26/1974	2/1/2006	361	0.220	0.200	2,122,153	1,874,089	89,038
WLV150-0001	Wabash River	5120108	IN	39.79222	-87.3744	28,903.1	2/9/1971	2/1/2006	373	0.227	0.190	2,333,000	2,100,950	105,811
WLV160-0001	Big Raccoon Cr	5120108	IN	39.79028	-86.9586	456.5	3/29/1999	2/1/2006	82	0.069	0.040	16,597	35,710	3,063
WLV190-0012	Big Raccoon Cr	5120108	IN	39.72917	-87.325	1,229.2	4/28/1999	2/1/2006	82	0.077	0.060	60,900	67,072	5,132
WLV200-0001	Wabash River	5120108	IN	39.65639	-87.3956	30,428.1	4/22/1976	2/1/2006	337	0.228	0.190	2,439,284	2,179,248	115,779
WMI060-0004	Mississinewa River	5120103	IN	40.62806	-85.7358	1,838.7	3/9/1971	2/14/2006	415	0.284	0.190	148,398	144,589	12,700
WMI060-0005	Mississinewa River	5120103	IN	40.57611	-85.6597	1,751.4	2/17/1971	2/14/2006	379	0.193	0.160	147,450	130,398	10,523
WMI060-0006	Mississinewa River	5120103	IN	40.74917	-86.0119	2,103.8	1/22/1991	2/22/2006	175	0.132	0.100	241,243	159,001	33,434
WPA040-0003	Patoka River	5120209	IN	38.32972	-86.9664	1,126.3	2/16/1971	2/27/2006	256	0.254	0.200	122,157	119,124	9,769
WPA060-0002	Patoka River	5120209	IN	38.3825	-87.3333	1,688.0	1/17/1973	2/27/2006	161	0.179	0.150	244,608	183,445	15,145
WQN0001	GENESEE RIVER	4130002	PA	41.9942	-77.8703	222.1	3/21/1990	12/1/1998	36	0.034	0.030	3,980	9,884	655
WQN0643	CONNEAUT CREEK	4120101	PA	41.918	-80.4692	398.2	1/19/1988	12/2/1998	129	0.053	0.040	16,670	17,881	1,665
WQN0702	MONONGAHELA RIVER	5020005	PA	40.1519	-79.9042	13,502.5	10/8/1970	12/7/1998	271	0.073	0.040	348,682	396,458	50,275
WQN0726	CASSELMAN RIVER	5020006	PA	39.7325	-79.1009	193.7	1/26/1988	12/2/1998	129	0.045	0.032	5,653	3,686	578
WQN0801	ALLEGHENY RIVER	5010009	PA	40.52745	-79.846	29,846.8	10/5/1971	12/16/1998	138	0.064	0.040	768,221	849,126	128,917
WQN0802	ALLEGHENY RIVER	5010006	PA	40.7611	-79.5458	23,274.4	1/4/1971	12/16/1998	275	0.065	0.040	639,046	618,257	82,788
WQN0807	ALLEGHENY RIVER	5010001	PA	41.965	-78.3861	1,422.6	12/21/1970	12/2/1998	280	0.071	0.040	24,442	24,852	2,862
WQN0843	CLARION RIVER	5010005	PA	41.1296	-79.5548	3,003.1	3/1/1973	12/7/1998	291	0.030	0.020	47,638	69,720	8,047
WQN0845	FRENCH CREEK	5010004	PA	41.402	-79.8316	3,187.4	2/20/1973	12/3/1998	204	0.057	0.050	96,525	108,248	7,851
WQN0846	FRENCH CREEK	5010004	PA	41.5893	-80.1502	2,094.4	10/11/1988	12/1/1998	124	0.078	0.068	99,496	73,045	6,464
WQN0861	MAHONING CREEK	5010006	PA	40.9221	-79.0064	411.4	1/21/1988	12/7/1998	130	0.064	0.040	11,515	12,103	1,048
WQN0867	ALLEGHENY RIVER	5010003	PA	41.2638	-79.8415	16,226.1	1/20/1988	12/3/1998	130	0.042	0.040	496,009	441,054	36,544
WQN0869	FRENCH CREEK	5010004	PA	41.7717	-80.1076	1,542.2	12/13/1994	12/2/1998	49	0.041	0.034	46,383	48,615	5,993
WQN0901	OHIO RIVER	5030101	OH	40.62058	-80.5589	60,905.2	10/5/1971	12/15/1998	263	0.099	0.080	2,336,723	2,487,939	235,451
WQN0902	OHIO RIVER	5030101	PA	40.533	-80.1881	50,462.4	2/10/1976	12/14/1998	256	0.111	0.080	3,048,111	1,773,835	170,574
WSA020-0002	Salamonie River	5120102	IN	40.55917	-85.2786	657.7	7/28/1998	2/13/2006	84	0.196	0.140	85,007	85,269	7,737
WSA040-0001	Salamonie River	5120102	IN	40.82972	-85.7189	1,452.7	2/17/1971	2/22/2006	372	0.147	0.100	141,669	125,100	14,391
WSA040-0005	Salamonie River	5120102	IN	40.74167	-85.5089	1,154.5	10/4/1971	2/13/2006	257	0.199	0.150	198,871	117,890	28,037
WSU020-0003	Sugar Cr	5120110	IN	40.14333	-86.6958	749.7	3/15/1999	2/2/2006	81	0.140	0.110	34,332	59,827	4,987
WSU050-0002	Sugar Cr	5120110	IN	40.05	-86.9225	1,338.7	3/15/1999	2/2/2006	83	0.218	0.160	68,307	107,280	7,746
WSU050-0005	Sugar Cr	5120110	IN	39.94611	-87.0586	1,721.4	1/24/1973	2/1/2006	378	0.146	0.100	81,958	129,242	8,188
WTI050-0009	Tippecanoe River	5120106	IN	41.09417	-86.2403	1,629.7	3/6/1991	2/8/2006	169	0.135	0.120	87,319	73,028	5,332
WTI080-0001	Tippecanoe River	5120106	IN	41.00667	-86.6028	2,695.6	3/25/1998	2/15/2006	94	0.087	0.080	101,846	115,770	5,608
WTI150-0011	Tippecanoe River	5120106	IN	40.59389	-86.7708	4,868.3	10/1/1986	2/22/2006	227	0.113	0.080	137,802	175,930	9,645
WUW060-0002	Wabash River	5120101	IN	40.61667	-84.965	1,233.8	1/8/1991	12/2/2002	133	0.434	0.370	248,245	204,590	25,904
WUW070-0002	Wabash River	5120101	IN	40.81944	-85.3425	1,661.0	1/10/1991	1/21/2003	135	0.310	0.300	191,353	238,169	15,405
WUW090-0001	Wabash River	5120101	IN	40.85833	-85.5075	2,000.7	1/10/1991	1/21/2003	140	0.235	0.200	219,679	251,551	18,052
WUW120-0002	Little River	5120101	IN	40.89861	-85.4133	702.6	2/25/1998	2/6/2006	91	0.239	0.205	63,165	43,048	6,560
WUW140-0001	Wabash River	5120101	IN	40.86889	-85.6019	2,987.6	2/23/1977	1/21/2003	285	0.308	0.260	286,367	317,000	18,951
WUW160-0006	Wabash River	5120101	IN	40.74222	-86.0967	7,083.3	1/12/1971	2/22/2006	405	0.241	0.180	682,602	648,096	45,315
WUW170-0002	Pipe Cr	5120101	IN	40.72167	-86.1983	452.3	3/25/1998	9/9/2003	63	0.134	0.100	23,485	39,052	4,274
WVE100-0001	Vermillion River	5120109	IN	39.96139	-87.4519	3,705.7	2/26/1974	2/1/2006	364	0.400	0.290	225,133	297,127	12,561

WWE060-0004	Mill Cr	5120203	IN	39.63667	-86.6406	144.4	10/1/1974	9/24/2003	231	0.072	0.040	3,249	10,261	464
WWE080-0001	Eel River	5120203	IN	39.35056	-87.0725	2,256.0	2/29/1996	2/1/2006	89	0.097	0.075	152,001	133,293	12,823
WWE090-0001	Eel River	5120203	IN	39.12423	-86.9702	3,124.8	1/22/1986	2/14/2006	136	0.152	0.110	252,990	190,426	24,133
WWL020-0003	W Fk White River	5120202	IN	39.28028	-86.7619	7,765.8	1/28/1991	2/14/2006	171	0.358	0.290	851,305	828,632	53,542
WWL030-0003	W Fk White River	5120202	IN	39.11194	-86.9625	11,405.4	2/29/1996	2/14/2006	89	0.292	0.270	1,162,133	1,035,477	67,233
WWL070-0003	W Fk White River	5120202	IN	38.795	-87.2417	12,919.1	1/28/1991	2/13/2006	181	0.238	0.220	1,322,943	1,144,024	72,074
WWL100-0005	White River	5120202	IN	38.51167	-87.2886	28,823.8	2/16/1971	2/28/2006	401	0.257	0.190	2,338,980	2,285,520	141,593
WWU010-0001	W Fk White River	5120201	IN	40.17833	-85.3422	581.5	1/17/1973	2/15/2006	377	0.133	0.100	39,899	28,936	3,327
WWU010-0006	W Fk White River	5120201	IN	40.18222	-84.9689	88.4	4/13/1978	9/23/2003	284	0.119	0.090	5,095	4,111	598
WWU020-0005	W Fk White River	5120201	IN	40.17889	-85.495	655.3	3/11/1971	2/15/2006	397	0.556	0.240	69,999	59,989	7,387
WWU030-0003	W Fk White River	5120201	IN	40.10667	-85.6728	1,059.2	3/11/1971	2/15/2006	397	0.323	0.190	82,608	77,459	8,237
WWU040-0004	W Fk White River	5120201	IN	40.14222	-85.8628	1,480.1	1/7/1991	2/13/2006	183	0.291	0.260	151,696	116,993	9,626
WWU090-0002	W Fk White River	5120201	IN	39.91	-86.105	3,182.6	7/21/1971	2/15/2006	390	0.318	0.240	284,298	216,588	15,473
WWU110-0001	Fall Cr	5120201	IN	39.78194	-86.1767	861.3	1/23/1986	2/15/2006	237	0.173	0.110	34,285	47,464	3,255
WWU110-0002	Fall Cr	5120201	IN	39.83444	-86.1219	803.5	1/21/1971	2/15/2006	387	0.122	0.080	21,653	36,735	1,577
WWU120-0001	Eagle Cr	5120201	IN	39.735	-86.1967	539.3	2/8/1971	2/15/2006	398	0.278	0.167	14,097	38,774	1,258
WWU120-0002	Eagle Cr	5120201	IN	39.77833	-86.2506	449.2	1/23/1986	2/15/2006	240	0.074	0.040	16,083	27,073	2,053
WWU140-0003	W Fk White River	5120201	IN	39.56694	-86.2558	5,259.9	10/6/1992	2/21/2006	164	0.496	0.402	614,140	536,031	41,868
WWU150-0007	White Lick Cr	5120201	IN	39.51361	-86.3803	746.8	2/22/1996	2/21/2006	90	0.184	0.160	41,699	211,773	2,675
WWU160-0004	W Fk White River	5120201	IN	39.43389	-86.4494	6,462.6	1/17/1973	2/21/2006	307	0.599	0.380	671,899	775,396	36,986
YOU0925	YOUGH. R. GA. 0.7M. UPST.FR.BR.ON RT 42 AT FD	5020006	MD	39.65335	-79.4079	763.6	10/9/1973	12/5/1995	159	0.078	0.030	35,522	13,262	4,856
YOU1139	YOUGH. R. N OF RT 20 BR.DOWNST.FR. LT. YOUGH.	5020006	MD	39.42472	-79.4208	349.7	3/27/1974	12/5/1995	178	0.094	0.053	21,707	7,819	2,655

Table S2_TN. Summary of measured (with Fluxmaster) and estimated (with SPARROW) loads for each monitoring site used in the calibration of the SPARROW models, with a summary of the measured loads. [Not all water-water quality used in Fluxmaster calculations if outside of year range specified in Table S1.]

Supplemental Material to Robertson and Saad, 2011, Journal of the American Water Resources Association, Nutrient Inputs to the Laurentian Great Lakes By Source and River Basin Estimated Using SPARROW Watershed Models.

[kg, kilogram; km^2, square kilometer; mg/L, milligrams per liter; kg/yr; kilograms per year]
HUC8's are based on Seaber, P.R., F.P. Kapinos, and G.L. Knapp, 1987. Hydrologic Unit maps: U.S. Geol. Surv. Water-Supply Paper 2294, 63 p. Available online at: http://water.usgs.gov/GIS/huc.html

Identification and Location Information							Total Nitrogen Concentration and Load Data							
Station Identification Number	Name	HUC8	State	Latitude (decimal)	Longitude (decimal)	Area (km^2)	Water Quality Start Date	Water Quality End Date	Number of Observations	Midmonthly Mean (mg/L)	Midmonthly Median (mg/L)	Fluxmaster Load (kg/yr)	SPARROW Estimated Load (kg/yr)	Standard Deviation in Fluxmaster Loads (kg/yr)
000755	CUMBERLAND R 262.9 @ HWY 231 QUAD 313SE	5130201	TN	36.29917	-86.2631	28,282.3	11/7/1977	12/7/1998	114	0.765	0.660	11,771,812	21,014,782	669,115
000770	CUMBERLAND R 174.5 U/S FROM CLEESE'S FERRY	5130202	TN	36.14842	-86.8902	33,040.0	9/10/1973	10/22/1998	154	1.143	0.885	10,696,136	26,534,388	1,689,770
002205	RED RIVER 8.4 QUAD 301SE	5130206	TN	36.52833	-87.2783	2,352.1	7/29/1982	7/8/1998	34	3.518	3.465	4,553,358	4,610,581	418,109
010	MISSISSIPPI R BR ON CR-441 1 MI SW OF BLACKBERRY	7010103	MN	47.17447	-93.4206	9,310.1	5/16/1974	9/27/1994	147	0.865	0.823	1,180,237	579,178	33,131
018	KNIFE RIVER UPSTREAM OF US-61 AT KNIFE RIVER	4010102	MN	46.94698	-91.7951	225.8	7/10/1974	9/22/1993	59	0.668	0.650	68,867	51,963	7,817
020051	DRUMMOND BOG DISCHARGE 1MI NE DRUMMOND,WI	4010302	WI	46.34888	-91.2584	88.9	10/4/1989	9/18/1997	89	1.146	0.995	34,776	13,062	3,499
03011800	Kinzua Creek near Guffey, PA	5010001	PA	41.76673	-78.7187	100.5	8/16/1995	8/16/1995	1	0.330	0.330			
03012600	Allegheny River at Warren, PA	5010001	PA	41.8245	-79.1189	5,757.6	4/15/2002	9/7/2005	20	0.706	0.590			
03015000	Conewango Creek at Russell, PA	5010002	PA	41.93811	-79.1331	2,113.4	4/15/2002	9/7/2005	21	1.011	1.000			
03015500	Brokenstraw Creek at Youngsville, PA	5010001	PA	41.85447	-79.3164	831.4	4/16/2002	9/7/2005	20	0.905	0.920			
03016000	Allegheny River at West Hickory, PA	5010003	PA	41.56971	-79.4056	9,479.4	10/25/1978	9/6/2005	115	1.065	0.905	4,124,793	5,825,335	381,097
03020500	Oil Creek at Rouseville, PA	5010003	PA	41.48096	-79.6953	777.0	4/24/2002	9/21/2005	20	0.527	0.520			
03024000	French Creek at Utica, PA	5010004	PA	41.43792	-79.9554	2,662.5	4/18/1996	11/20/2002	33	0.948	0.927	2,309,465	2,470,188	301,840
03029500	Clarion River at Cooksburg, PA	5010005	PA	41.3314	-79.209	2,090.1	4/16/2002	9/6/2005	19	0.408	0.400			
03031500	Allegheny River at Parker, PA	5010006	PA	41.10086	-79.6794	19,867.9	3/1/1982	1/26/2005	30	0.619	0.590	8,925,531	12,800,054	1,036,063
03032500	Redbank Creek at St. Charles, PA	5010006	PA	40.99482	-79.3952	1,367.5	6/19/2002	9/22/2005	19	0.711	0.660			
03040000	Stonycreek River at Ferndale, PA	5010007	PA	40.28592	-78.9203	1,168.1	4/23/1996	9/28/1998	36	1.064	1.045	728,852	936,880	32,554
03042000	Blacklick Creek at Josephine, PA	5010007	PA	40.47345	-79.1834	497.3								
03044000	Conemaugh River at Tunnelton, PA	5010007	PA	40.45451	-79.3909	3,517.2	4/10/2002	9/28/2005	21	1.364	1.240			
03049625	Allegheny River at New Kensington, PA	5010009	PA	40.56451	-79.7726	29,904.1	9/24/1973	8/10/2004	202	1.026	0.952	16,847,320	19,780,306	633,101
03050000	TYGART VALLEY RIVER NEAR DAILEY, WV	5020001	WV	38.80901	-79.8822	479.2	6/18/1974	4/19/1995	84	0.785	0.740	238,491	296,031	16,123
03061000	WEST FORK RIVER AT ENTERPRISE, WV	5020002	WV	39.42209	-80.2759	1,965.8	7/16/1974	9/16/2004	278	1.001	0.925	952,167	1,056,294	69,220
03063000	Monongahela R at Lock & Dam 8, at Pnt Marion, PA	5020003	PA	39.72702	-79.9115	7,044.8	6/17/1971	9/26/2005	24	0.896	0.900			
03072000	Dunkard Creek at Shannopin, PA	5020005	PA	39.75916	-79.9703	593.1	4/30/1996	9/26/2005	54	0.645	0.600	199,197	257,449	21,684
03077500	Youghiogheny River at Youghiogheny River Dam, PA	5020006	PA	39.80567	-79.3647	1,129.2	4/29/2002	9/27/2005	21	0.919	0.900			
03079000	Casselman River at Markleton, PA	5020006	PA	39.85987	-79.2283	989.4	8/17/1995	8/17/1995	1	0.810	0.810			
03083250	Sewickley Creek at Hunker, PA	5020006	PA	40.20642	-79.623	230.0								
03083500	Youghiogheny River at Sutersville, PA	5020006	PA	40.24003	-79.8063	4,441.9	11/1/1979	9/14/2005	72	1.196	1.080	3,499,933	3,250,147	204,514
03085000	Monongahela River at Braddock, PA	5020005	PA	40.39043	-79.8586	19,002.8	2/13/1973	8/10/2004	221	1.515	1.300	13,422,539	11,631,783	684,072
03086000	Ohio River at Sewickley, PA	5030101	PA	40.55023	-80.204	50,505.0	9/26/1973	9/1/2005	80	1.285	1.158	38,401,696	36,125,444	1,146,897
03099500	Mahoning River at Lowellville OH	5030103	OH	41.03598	-80.5368	2,779.1	10/26/1987	10/7/2000	143	3.289	2.980	2,325,354	2,905,490	123,856
03099600	Mahoning River at North Edinburg, PA	5030103	PA	41.01839	-80.4406	2,846.4	6/29/1994	9/13/2005	27	2.271	2.280	2,758,768	2,964,118	125,291
03103500	Shenango River at Sharpsville, PA	5030102	PA	41.26617	-80.4726	1,512.6	10/18/1978	9/19/2005	115	1.865	1.218	1,182,326	823,755	177,555
03105500	Beaver River at Wampum, PA	5030104	PA	40.88894	-80.3366	5,788.7	4/22/1977	9/15/2003	45	2.308	2.120	4,467,841	5,213,738	554,441
03106000	Connoquenessing Creek near Zelienople, PA	5030105	PA	40.81673	-80.2426	922.0	4/17/2002	9/1/2005	20	1.861	1.820			
03106500	Slippery Rock Creek at Wurtemburg, PA	5030105	PA	40.88412	-80.2338	1,030.8	4/17/2002	9/1/2005	21	0.931	0.880			
03107500	Beaver River at Beaver Falls, PA	5030104	PA	40.76298	-80.3159	8,044.5	7/1/1976	9/13/2007	380	2.443	2.280	6,732,541	9,867,563	208,198
03108000	Raccoon Creek at Moffatts Mill, PA	5030101	PA	40.62784	-80.3376	461.0	4/9/2002	9/1/2005	21	1.222	1.080			
03109500	Little Beaver Creek near East Liverpool OH	5030101	OH	40.67576	-80.5404	1,284.6	12/17/1975	12/22/1998	257	1.729	1.625	1,199,182	1,056,887	61,224
03146500	Licking River near Newark OH	5040006	OH	40.05923	-82.3383	1,390.8	10/11/1974	1/17/1997	272	3.605	3.260	2,163,203	2,094,262	98,759
03150000	Muskingum River at McConnelsville OH	5040004	OH	39.64409	-81.8506	19,223.0	1/23/1973	10/17/2004	1287	2.257	2.042	19,796,219	18,203,154	229,325
03155000	LITTLE KANAWHA RIVER AT PALESTINE, WV	5030203	WV	39.05893	-81.3893	3,926.4	1/29/1974	4/3/1995	238	0.604	0.520	1,390,454	1,729,759	83,613
03157000	Clear Creek near Rockbridge OH	5030204	OH	39.58835	-82.5785	230.5	5/4/1977	9/20/1990	31	1.680	1.200	877,074	282,778	400,956
03164000	NEW RIVER NEAR GALAX, VA	5050001	VA	36.64646	-80.9777	2,929.3	11/3/1970	12/17/1998	287	0.732	0.705	1,713,851	1,662,362	94,963
03167000	REED CREEK AT GRAHAMS FORGE, VA	5050001	VA	36.93946	-80.887	639.7	11/3/1970	11/17/1998	250	1.040	1.000	287,541	371,574	16,554
03170000	LITTLE RIVER AT GRAYSONTOWN, VA	5050001	VA	37.03733	-80.5567	777.0	7/25/1979	9/4/1998	130	0.560	0.535	240,018	450,878	16,525

03171500	NEW RIVER AT EGGLESTON, VA	5050002	VA	37.29008	-80.6161	7,617.2	11/18/1974	12/8/1998	160	1.078	1.000	3,641,783	4,212,080	179,974
03176500	NEW RIVER AT GLEN LYN, VA	5050002	VA	37.37221	-80.8613	9,759.1	11/2/1970	12/8/1998	403	1.145	1.025	3,959,439	7,704,718	176,576
03184000	GREENBRIER RIVER AT HILLDALE, WV	5050003	WV	37.64012	-80.8047	4,193.2	12/4/1973	4/6/1995	238	0.712	0.710	1,921,209	1,775,228	89,834
03189100	GAULEY RIVER NEAR CRAIGSVILLE, WV	5050005	WV	38.29094	-80.6409	1,370.1	6/25/1974	5/28/1998	72	0.774	0.600	929,573	948,442	103,689
03189600	GAULEY RIVER BELOW SUMMERSVILLE, WV	5050005	WV	38.21511	-80.8882	2,087.5	6/25/1974	5/28/1998	62	0.696	0.700	918,481	1,281,866	76,792
03193000	KANAWHA RIVER AT KANAWHA FALLS, WV	5050006	WV	38.13774	-81.2135	21,680.9	12/17/1973	9/3/1998	38	0.853	0.698	9,421,631	12,897,528	958,920
03194700	ELK RIVER BELOW WEBSTER SPRINGS, WV	5050007	WV	38.59741	-80.4905	688.9	1/23/1974	5/27/1998	62	0.574	0.515	366,545	483,540	36,648
03198350	CLEAR FORK AT WHITESVILLE, WV	5050009	WV	37.96624	-81.5241	162.7	10/22/1996	3/8/2000	49	0.678	0.692	81,963	67,547	6,225
03201300	KANAWHA RIVER AT WINFIELD, WV	5050008	WV	38.52657	-81.9116	30,585.3	2/15/1974	9/19/2007	586	1.274	1.100	14,160,528	17,319,495	566,046
03202000	Raccoon Creek at Adamsville OH	5090101	OH	38.85896	-82.3618	1,515.2	8/11/1993	9/1/1998	47	0.880	0.630	441,364	758,474	62,532
03209300	RUSSELL FORK AT ELKHORN CITY, KY	5070202	KY	37.30401	-82.3428	1,434.9	6/2/1993	12/12/1995	29	0.840	0.540	621,459	400,027	126,098
03209500	LEVISA FORK AT PIKEVILLE, KY	5070203	KY	37.46425	-82.5263	3,190.9	3/27/1979	11/16/2006	257	0.649	0.619	1,093,373	894,111	67,313
03215000	BIG SANDY R AT LOUISA, KY	5070204	WV	38.1708	-82.6347	10,093.2	10/23/1974	9/18/2007	385	0.980	0.850	4,697,521	3,108,672	363,709
03216600	OHIO RIVER AT GREENUP DAM NEAR GREENUP, KY	5090103	KY	38.64684	-82.8588	160,580.0	10/22/1974	9/13/2007	180	1.484	1.400	132,126,594	107,140,635	4,258,795
03219500	Scioto River near Prospect OH	5060001	OH	40.4195	-83.1971	1,468.5	2/12/1976	10/2/1995	125	5.543	4.870	3,606,528	6,890,304	282,520
03225500	Olentangy River near Delaware OH	5060001	OH	40.35499	-83.0678	1,017.9	6/18/1974	3/28/1995	66	3.617	3.100	2,158,667	2,962,198	376,577
03229000	Alum Creek at Columbus OH	5060001	OH	39.94484	-82.9413	489.5	4/13/1990	9/19/1996	53	1.624	1.560	278,048	530,240	22,458
03229500	Big Walnut Creek at Rees OH	5060001	OH	39.85673	-82.9571	1,409.0	1/29/1976	9/25/1991	27	1.694	1.480	763,305	1,530,484	66,105
03230450	Hellbranch Run near Harrisburg OH	5060001	OH	39.83063	-83.1598	95.8	1/12/1996	9/27/2005	144	3.333	3.285	290,948	115,300	23,167
03230500	Big Darby Creek at Darbyville OH	5060001	OH	39.70097	-83.1096	1,383.1	1/29/1976	12/12/1996	258	4.089	3.790	4,075,287	4,265,302	293,245
03230800	Deer Creek at Mount Sterling OH	5060002	OH	39.71514	-83.2574	590.5	6/19/1974	12/16/1996	58	4.172	3.200	1,762,453	2,162,758	350,022
03231500	Scioto River at Chillicothe OH	5060002	OH	39.34217	-82.9713	9,968.9	7/25/1974	10/18/2004	2767	4.622	4.310	20,088,578	28,576,209	176,929
03232000	Paint Creek near Greenfield OH	5060003	OH	39.37929	-83.3754	644.9	6/1/1993	1/10/1997	43	5.428	5.320	1,999,955	2,449,097	335,707
03234500	Scioto River at Higby OH	5060002	OH	39.21192	-82.8644	13,289.3	10/3/1974	12/14/1998	209	4.209	4.000	20,747,207	35,695,119	1,227,434
03237280	Upper Twin Creek at McGaw OH	5090201	OH	38.64375	-83.2155	31.6	10/21/1980	8/29/2001	154	0.541	0.480	3,153	12,340	569
03240000	Little Miami River near Oldtown OH	5090202	OH	39.74792	-83.9314	334.1	9/16/1975	12/17/1998	253	4.589	4.345	798,867	731,415	25,724
03242200	Anderson Fork near New Burlington OH	5090202	OH	39.56638	-83.9028	201.5	7/27/1983	9/1/1998	25	3.601	2.655			
03245500	Little Miami River at Milford OH	5090202	OH	39.17138	-84.2985	3,115.8	12/19/1974	9/13/2000	233	3.944	3.500	7,250,904	6,137,585	541,126
03248100	Little Miami River at Cincinnati OH	5090202	OH	39.10921	-84.4016	4,517.0	7/22/1975	9/14/2007	205	3.144	3.000	6,060,496	8,000,825	405,598
03265000	Stillwater River at Pleasant Hill OH	5080001	OH	40.05779	-84.356	1,302.8	5/20/1982	12/17/1998	54	4.811	4.010	5,411,659	6,752,496	719,119
03267900	Mad River at St Paris Pike at Eagle City OH	5080001	OH	39.96415	-83.8314	802.9	10/6/1976	10/13/2004	314	4.408	4.310	1,608,059	1,821,883	29,442
03270000	Mad River near Dayton OH	5080001	OH	39.79702	-84.0916	1,644.7	7/2/1974	12/27/1990	88	3.711	3.600	2,736,865	2,479,857	78,874
03271601	Great Miami River below Miamisburg OH	5080002	OH	39.60687	-84.2865	7,031.9	4/22/1996	10/18/2004	2713	4.939	4.441	16,861,463	16,403,568	177,338
03274000	Great Miami River at Hamilton OH	5080002	OH	39.39116	-84.5718	9,401.7	1/30/1975	9/18/2001	150	4.794	4.605	19,885,029	21,958,086	1,197,723
03274600	Great Miami River at New Baltimore OH	5080002	OH	39.26318	-84.6677	9,878.3	10/1/1974	9/1/1993	196	5.333	4.900	23,296,697	23,060,330	974,989
03275000	WHITEWATER RIVER NEAR ALPINE, IN	5080003	IN	39.57944	-85.1574	1,352.0	2/18/1987	9/13/2000	70	3.985	3.600	3,293,462	3,772,740	212,922
03275600	EAST FORK WHITEWATER RIVER AT ABINGTON, IND.	5080003	IN	39.73251	-84.9596	518.0	2/28/1977	2/14/2006	124	4.023	3.800	1,039,300	1,397,440	59,553
03277200	OHIO RIVER AT MARKLAND DAM NEAR WARSAW, KY	5090203	KY	38.77632	-84.9642	215,410.3	10/30/1974	9/18/2007	312	2.057	1.910	182,062,089	203,048,696	8,371,426
03281000	MIDDLE FORK KENTUCKY RIVER AT TALLEGA, KY	5100202	KY	37.55533	-83.5938	1,390.8	6/2/1982	9/19/2006	266	0.468	0.405	323,503	467,950	22,925
03281500	SOUTH FORK KENTUCKY RIVER AT BOONEVILLE, KY	5100203	KY	37.47928	-83.6751	1,870.0	6/2/1982	9/19/2006	257	0.502	0.443	487,198	731,725	40,011
03285000	DIX RIVER NEAR DANVILLE, KY	5100205	KY	37.64208	-84.6609	823.6	10/15/1985	10/3/2006	190	1.513	1.414	1,030,859	1,049,401	74,854
03289500	ELKHORN CREEK NEAR FRANKFORT, KY	5100205	KY	38.26859	-84.8146	1,225.1	10/14/1987	9/19/2006	97	4.315	3.900	2,187,725	1,632,593	188,022
03290500	KENTUCKY RIVER AT LOCK 2 AT LOCKPORT, KY	5100205	KY	38.43867	-84.9625	16,006.2	2/7/1973	6/6/1995	211	1.406	1.300	11,334,351	11,104,585	475,537
03291500	EAGLE CREEK AT GLENCOE, KY	5100205	KY	38.70589	-84.8252	1,131.8	12/13/1988	11/17/2006	164	1.073	0.920	730,523	862,376	78,447
03294600	OHIO RIVER AT KOSMOSDALE, KY	5140101	KY	38.03519	-85.9175	236,208.0	10/12/1976	9/18/2007	270	2.102	2.020	214,652,195	220,372,452	7,425,131
03298500	SALT RIVER AT SHEPHERDSVILLE, KY	5140102	KY	37.98519	-85.7174	3,100.2	10/31/1978	12/19/2006	364	1.926	1.700	3,537,572	3,689,896	167,455
03301630	ROLLING FORK NEAR LEBANON JUNCTION, KY	5140103	KY	37.82258	-85.7477	3,561.3	10/8/1974	12/19/2006	275	1.469	1.300	3,081,751	4,363,123	172,808
03303280	OHIO RIVER AT CANNELTON DAM AT CANNELTON, IN	5140201	KY	37.89848	-86.7042	251,230.0	10/1/1975	9/19/2007	442	1.843	1.812	234,617,401	233,546,731	5,768,358
03308500	GREEN RIVER AT MUNFORDVILLE, KY	5110001	KY	37.26779	-85.8857	4,333.1	3/25/1980	11/14/2006	304	1.195	1.141	3,295,823	5,678,041	128,763
03314500	BARREN RIVER AT BOWLING GREEN, KY	5110002	KY	37.0014	-86.4305	4,788.9	6/13/1979	9/12/1994	159	1.514	1.476	3,910,436	6,111,572	173,321
03322500	WABASH RIVER NEAR NEW CORYDON, IND	5120101	IN	40.56323	-84.8028	678.6	12/22/1970	2/13/2006	41	7.617	6.200	3,741,130	4,439,383	696,108
03325500	MISSISSINEWA RIVER NEAR RIDGEVILLE, IND.	5120103	IN	40.28015	-84.9958	344.5	2/5/1980	2/13/2006	152	5.469	5.250	1,596,372	2,104,568	154,919
03328500	EEL RIVER NEAR LOGANSPORT, IN	5120104	IN	40.78212	-86.2646	2,043.5	1/22/1976	2/22/2006	164	4.247	3.700	4,708,003	5,511,492	284,709
03329700	DEER CREEK NEAR DELPHI, IND.	5120105	IN	40.59032	-86.6217	709.7	3/25/1998	2/22/2006	92	5.189	4.250	2,563,364	3,201,954	234,048
03335500	WABASH RIVER AT LAFAYETTE IND	5120108	IN	40.42193	-86.8963	18,821.5	1/16/1980	12/6/1995	175	4.826	4.800	43,574,901	63,970,872	1,969,051
03336645	MIDDLE FORK VERMILION RIVER ABOVE OAKWOOD, IL	5120109	IL	40.13627	-87.7462	1,118.9	10/19/1978	12/8/1998	189	6.611	6.900	6,908,986	2,542,016	723,618
03338780	NORTH FORK VERMILION RIVER NEAR BISMARCK, IL	5120109	IL	40.26517	-87.6438	678.6	11/10/1988	12/30/1998	88	7.160	7.600	6,317,944	1,836,245	853,652
03339000	VERMILION RIVER NEAR DANVILLE, IL	5120109	IL	40.0874	-87.597	3,341.1	4/11/1978	12/10/1998	170	7.805	7.900	10,451,022	8,397,587	460,278
03341910	WABASH RIVER AT HUTSONVILLE, IL	5120111	IN	39.11028	-87.655	33,715.1	7/20/1972	6/20/1973	11	4.644	4.270			
03341920	TURMAN CREEK NR FARMERSBURG, IN	5120111	IN	39.24448	-87.4078	33.7	7/20/1972	6/20/1973	12	4.688	4.571			

03343395	EMBARRAS RIVER AT CAMARGO, IL	5120112	IL	39.79916	-88.1701	466.2	9/27/1978	1/31/1979	4	8.475	9.050			
03345500	EMBARRAS RIVER AT STE. MARIE, IL	5120112	IL	38.93536	-88.0146	3,926.4	11/17/1986	9/10/1996	42	5.530	5.350	11,040,984	8,572,962	1,822,120
03346000	NORTH FORK EMBARRAS RIVER NEAR OBLONG, IL	5120112	IL	39.00893	-87.9461	823.6								
03351500	FALL CREEK NEAR FORTVILLE, IND.	5120201	IN	39.95456	-85.8676	437.7	10/26/1972	2/15/2006	89	3.164	2.800	753,417	1,267,739	39,502
03354000	WHITE RIVER NEAR CENTERTON, IN	5120201	IN	39.49722	-86.4009	6,330.0	10/26/1972	8/17/1995	187	5.380	5.200	11,850,680	19,191,459	441,062
03357500	BIG WALNUT CREEK NEAR REELSVILLE, IN	5120203	IN	39.53572	-86.9763	844.3	3/17/1999	11/13/2003	55	3.181	2.825	1,365,613	1,725,285	100,161
03358000	MILL CREEK NEAR CATARACT, IND.	5120203	IN	39.43349	-86.7634	634.6	7/26/1971	2/14/2006	253	3.411	3.100	2,635,976	1,116,099	241,066
03362500	SUGAR CREEK NEAR EDINBURGH, IN	5120204	IN	39.36097	-85.9982	1,227.7	5/29/1996	2/9/2006	98	3.186	3.150	3,031,068	3,520,223	201,987
03366500	MUSCATATUCK RIVER NEAR DEPUTY, IN	5120207	IN	38.80426	-85.6738	758.9	4/5/1993	4/18/1995	28	1.504	1.450	774,203	841,202	133,212
03374100	WHITE RIVER AT HAZLETON, IN	5120202	IN	38.48977	-87.55	29,280.0	2/21/1973	2/13/2006	502	2.726	2.600	42,854,016	59,715,887	1,302,622
03378000	BONPAS CREEK AT BROWNS, IL	5120113	IL	38.38246	-87.9769	590.5	11/2/1989	11/2/1989	1	1.250	1.250			
03378500	WABASH RIVER AT NEW HARMONY, IND.	5120113	IN	38.1314	-87.9429	75,716.1	11/7/1974	7/13/2006	288	3.453	3.400	124,814,331	180,130,745	4,410,585
03378635	LITTLE WABASH RIVER NEAR EFFINGHAM, IL	5120114	IL	39.10344	-88.5926	621.6	6/11/1986	2/14/1990	4	1.800	1.325			
03378900	LITTLE WABASH RIVER AT LOUISVILLE, IL	5120114	IL	38.77291	-88.497	1,929.6	8/8/1989	2/22/1990	3	1.873	1.870			
03379500	LITTLE WABASH RIVER BELOW CLAY CITY, IL	5120114	IL	38.63345	-88.2953	2,929.3	8/7/1989	2/22/1990	3	1.533	1.150			
03379600	LITTLE WABASH RIVER AT BLOOD, IL	5120114	IL	38.51894	-88.1319	3,577.7	8/7/1989	2/21/1990	3	1.567	1.250			
03380500	SKILLET FORK AT WAYNE CITY, IL	5120115	IL	38.35867	-88.5848	1,201.8	7/31/1989	7/13/1998	4	2.127	1.985			
03381495	LITTLE WABASH RIVER AT MAIN ST AT CARMI, IL	5120114	IL	38.09232	-88.1561	7,997.9	10/29/1979	12/16/1998	178	2.229	2.100	7,547,067	11,780,193	359,332
03382090	SUGAR CREEK NEAR STONEFORT, IL	5140204	IL	37.65533	-88.7633	90.7	6/8/1993	12/14/1993	5	1.209	0.900			
03382100	SOUTH FORK SALINE RIVER NR CARRIER MILLS, IL	5140204	IL	37.63643	-88.6772	380.7	12/14/1977	12/10/1998	187	0.992	0.893	222,697	195,148	25,612
03382185	BANKSTON FORK NEAR DORRIS HEIGHTS, IL	5140204	IL	37.76754	-88.5404	201.2	6/8/1993	12/14/1993	4	1.278	1.270			
03384450	LUSK CREEK NEAR EDDYVILLE, IL	5140203	IL	37.47253	-88.5476	111.1	9/19/1979	12/23/1998	171	0.413	0.350	31,716	51,471	3,780
03404500	CUMBERLAND RIVER AT CUMBERLAND FALLS, KY	5130101	KY	36.83694	-84.3437	5,120.4	1/22/1979	8/21/2006	258	0.612	0.527	1,793,472	2,405,380	111,806
03428500	WEST FORK STONES RIVER NEAR SMYRNA, TN	5130203	TN	35.94027	-86.4649	613.8	9/29/1982	12/7/1998	31	2.452	1.770	492,953	701,115	59,186
03434500	HARPETH RIVER NEAR KINGSTON SPRINGS, TN	5130204	TN	36.122	-87.0989	1,763.8	6/25/1975	10/22/1998	50	1.334	1.005	1,694,607	1,402,023	259,178
03612000	CACHE RIVER AT FORMAN, IL	5140206	IL	37.3352	-88.9258	632.0	6/9/1992	12/8/1992	3	0.912	0.860			
04024000	ST. LOUIS RIVER AT SCANLON, MN	4010201	MN	46.70322	-92.4178	8,883.7	6/4/1974	8/1/1994	276	1.033	0.990	2,388,795	1,334,465	52,747
04024430	NEMADJI RIVER NEAR SOUTH SUPERIOR, WI	4010301	WI	46.63326	-92.0943	1,087.8	1/28/1974	3/19/1998	256	0.803	0.700	376,633	339,828	15,848
04027595	BAD RIVER AT ODANAH, WI	4010302	WI	46.61028	-90.6869	2,512.3	11/30/1970	2/7/2005	183	0.642	0.560	598,986	685,391	61,668
04040000	ONTONAGON RIVER NEAR ROCKLAND, MI	4020102	MI	46.7207	-89.2075	3,470.6	10/3/1974	8/29/1995	136	0.515	0.460	711,812	647,422	32,530
04057005	MANISTIQUE RIVER AT MANISTIQUE, MI	4060106	MI	45.95164	-86.2485	3,755.5	1/25/1971	10/26/1995	236	0.614	0.595	946,083	575,097	18,477
04063700	POPPLE RIVER NEAR FENCE, WI	4030108	WI	45.76347	-88.4632	360.0	10/21/1980	9/20/2007	229	0.644	0.561	60,721	60,654	2,798
04067500	MENOMINEE RIVER NEAR MC ALLISTER, WI	4030108	MI	45.32572	-87.6621	10,178.7	10/24/1979	8/8/2007	113	0.649	0.570	1,430,394	1,947,240	83,905
04071000	OCONTO RIVER NEAR GILLETT, WI	4030104	WI	44.86465	-88.3001	1,826.0	1/29/1973	10/13/2003	208	0.689	0.623	325,417	552,016	23,048
04072050	DUCK CREEK AT SEMINARY ROAD NEAR ONEIDA, WI	4030103	WI	44.46521	-88.2187	247.3	4/7/1993	10/20/2004	108	3.497	2.700	308,304	318,826	29,713
04072150	DUCK CREEK NEAR HOWARD, WI	4030103	WI	44.53371	-88.1291	279.7	9/21/1988	10/15/2002	217	2.875	2.255	250,337	368,806	33,256
040734644	SILVER CREEK AT SOUTH KORO ROAD NEAR RIPON, WI	4030201	WI	43.8581	-88.87	93.8	7/10/1995	7/10/1995	1	5.800	5.800			
04073468	GREEN LAKE INLET AT CT HIGHWAY A NR GREEN L, WI	4030201	WI	43.82369	-88.9269	138.6								
04073470	PUCHYAN RIVER AT GREEN LAKE, WI	4030201	WI	43.84665	-88.9601	266.8								
04075050	WOLF RIVER AT HIGHWAY M NEAR LANGLADE, WI	4030202	WI	45.12632	-88.6635	1,266.5	4/26/1994	8/28/2001	29	0.557	0.525	208,324	256,876	10,472
04077100	WOLF RIVER AT KESHENA, WI	4030202	WI	44.88332	-88.6348	2,297.8	5/31/1995	8/27/2001	27	0.584	0.535	404,848	484,196	28,836
04077630	RED RIVER AT MORGAN ROAD NEAR MORGAN, WI	4030202	WI	44.89814	-88.8441	295.3	11/4/1992	7/2/1998	171	1.213	1.074	138,169	125,558	4,798
04080798	TOMORROW RIVER NEAR NELSONVILLE, WI	4030202	WI	44.52432	-89.3381	114.0	4/9/1993	10/17/2002	38	2.559	2.500	83,212	66,428	6,181
04085139	FOX RIVER AT MOUTH AT GREEN BAY, WI	4030103	WI	44.53949	-88.0049	16,394.7	10/5/1988	9/15/2005	153	1.640	1.575	6,283,332	6,658,683	525,614
04085427	MANITOWOC RIVER AT MANITOWOC, WI	4030101	WI	44.10708	-87.7151	1,362.3	3/29/1979	9/20/2007	143	2.670	2.400	851,841	1,786,111	44,990
04086500	CEDAR CREEK NEAR CEDARBURG, WI	4040003	WI	43.32308	-87.9786	310.8	1/25/1977	11/8/2004	57	2.269	2.135	320,036	343,372	29,785
04086600	MILWAUKEE RIVER NEAR CEDARBURG, WI	4040003	WI	43.27999	-87.9421	1,572.1	9/28/1994	8/29/2005	19	2.220	2.138			
04087000	MILWAUKEE RIVER AT MILWAUKEE, WI	4040003	WI	43.10001	-87.909	1,802.6	1/25/1973	9/26/2007	436	2.141	2.000	1,007,138	1,895,869	25,666
04087170	MILWAUKEE RIVER AT MOUTH AT MILWAUKEE, WI	4060200	WI	43.02505	-87.8985	2,258.5	3/29/1994	8/29/2005	47	1.440	1.343	920,905	2,295,325	53,850
04101500	ST. JOSEPH RIVER AT NILES, MI	4050001	MI	41.82928	-86.2596	9,494.9	2/12/1979	9/22/1995	103	2.560	2.400	9,114,864	12,372,920	211,118
04102533	ST. JOSEPH RIVER AT ST. JOSEPH, MI	4050001	MI	42.11318	-86.4853	12,095.3	1/14/1971	10/27/1995	247	2.288	2.150	10,613,640	15,051,773	125,572
04119300	GRAND RIVER AT EASTMANVILLE, MI	4050006	MI	43.01511	-85.9559	13,545.7	2/14/1979	10/7/1994	98	2.627	2.415	11,591,397	15,148,541	464,881
04122500	PERE MARQUETTE RIVER AT SCOTTVILLE, MI	4060101	MI	43.94469	-86.2792	1,763.8	2/16/1971	11/17/2005	108	0.485	0.460	360,382	603,413	17,352
04137500	AU SABLE RIVER NEAR AU SABLE, MI	4070007	MI	44.43622	-83.4344	4,504.0	6/6/1978	11/9/2005	183	0.314	0.279	262,124	739,766	21,274
04156100	TITTABAWASSEE RIVER NR MIDLAND, MI	4080201	MI	43.56864	-84.1936	6,345.5	7/24/1980	9/23/1996	216	2.008	1.635	3,792,584	4,200,703	127,302
04157000	SAGINAW RIVER AT SAGINAW, MI	4080206	MI	43.41322	-83.9642	15,695.4	3/12/1976	7/21/2005	67	2.547	2.300	10,295,686	14,125,265	928,313
04161820	CLINTON R AT STERLING HEIGHTS, MI	4090003	MI	42.61452	-83.0262	800.3	4/4/1996	8/30/2005	81	2.311	1.977	431,451	350,969	19,395
04165500	CLINTON R AT MORAVIAN DRIVE AT MT. CLEMENS, MI	4090003	MI	42.59572	-82.9093	1,901.1	10/8/1974	8/24/1995	136	3.818	3.600	1,884,566	1,560,491	62,111
04170000	HURON RIVER AT MILFORD, MI	4090005	MI	42.57892	-83.6276	341.9	4/6/1984	9/8/2003	47	1.011	0.950	85,418	143,623	6,631
04170500	HURON RIVER NEAR NEW HUDSON, MI	4090005	MI	42.51253	-83.6763	383.3	4/6/1984	9/8/2003	44	0.924	0.900	68,285	123,541	5,060

04175600	RIVER RAISIN NEAR MANCHESTER, MI	4100002	MI	42.16796	-84.0765	341.9	4/2/1996	8/26/2004	61	0.927	0.919	83,109	222,392	2,182
04176500	RIVER RAISIN NEAR MONROE, MI	4100002	MI	41.96033	-83.5311	2,698.8	1/19/1978	9/30/2004	6679	3.772	3.191	5,209,595	5,017,828	78,222
04178000	ST. JOSEPH RIVER NEAR NEWVILLE, IN	4100003	IN	41.38571	-84.8019	1,579.9	3/7/1996	10/25/2004	89	2.492	2.023	1,782,752	2,905,728	175,155
04186500	Auglaize River near Fort Jennings OH	4100007	OH	40.94899	-84.2665	859.9	3/8/1996	8/9/2005	85	5.111	4.445	2,815,766	4,316,435	364,864
04189000	Blanchard River near Findlay OH	4100008	OH	41.05609	-83.6879	896.1	4/23/1973	9/19/1996	129	8.273	8.240	1,795,960	3,296,371	159,518
04193500	Maumee River at Waterville OH	4100009	OH	41.50016	-83.7139	16,394.7	4/24/1973	8/10/2005	8330	5.610	5.320	52,272,291	67,826,179	874,357
04197100	Honey Creek at Melmore OH	4100011	OH	41.02214	-83.1096	385.9	2/16/1976	9/30/2004	8429	5.802	4.810	1,104,792	1,685,775	18,836
04198000	Sandusky River near Fremont OH	4100011	OH	41.30779	-83.1584	3,240.1	4/23/1973	9/30/2004	7411	4.925	4.306	14,118,936	13,977,196	335,388
04199000	Huron River at Milan OH	4100012	OH	41.30077	-82.6088	960.9	10/2/1973	8/20/1998	1761	4.097	3.390	2,239,787	1,969,598	71,325
04199500	Vermilion River near Vermilion OH	4100012	OH	41.38199	-82.3169	678.6	4/23/1973	9/30/2004	1462	2.815	2.195	2,308,865	1,153,826	150,142
04200500	Black River at Elyria OH	4110001	OH	41.37967	-82.1047	1,025.6	4/23/1973	12/9/1998	325	5.498	4.300	1,338,022	1,615,378	149,278
04206000	Cuyahoga River at Old Portage OH	4110002	OH	41.13559	-81.5472	1,046.4	3/27/1975	9/19/2000	18	1.690	1.676			
04208000	Cuyahoga River at Independence OH	4110002	OH	41.39532	-81.6294	1,831.1	9/27/1973	9/30/2004	7602	3.531	3.300	2,372,409	2,365,637	16,659
04209000	Chagrin River at Willoughby OH	4110003	OH	41.63136	-81.4027	637.1	10/23/1973	12/1/1998	251	0.904	0.800	368,619	484,566	42,331
04212100	Grand River near Painesville OH	4110004	OH	41.7188	-81.2278	1,774.2	11/26/1975	9/30/2004	5152	1.014	0.910	1,220,025	1,860,623	17,681
04213500	CATTARAUGUS CREEK AT GOWANDA NY	4120102	NY	42.46389	-78.9352	1,129.2	7/16/1975	2/26/1998	164	1.543	1.485	1,116,475	1,121,544	43,979
04214500	BUFFALO CREEK AT GARDENVILLE NY	4120103	NY	42.85478	-78.755	367.8	10/22/1970	11/8/1994	81	0.975	0.890	342,030	476,489	33,518
04227000	CANASERAGA CREEK AT SHAKERS CROSSING NY	4130002	NY	42.7371	-77.8404	867.7	10/8/1974	11/4/1996	40	1.447	1.384	502,160	577,042	61,409
04229500	HONEOYE CREEK AT HONEOYE FALLS NY	4130003	NY	42.95681	-77.589	507.6	4/6/1998	4/6/2005	546	0.775	0.630	112,269	260,498	4,601
04230500	OATKA CREEK AT GARBUTT NY	4130003	NY	43.00998	-77.7914	518.0	3/22/1989	9/16/2005	690	2.186	2.177	600,060	846,727	11,241
04232034	IRONDEQUOIT CR AT RAILROAD MILLS, NR FISHERS NY	4140101	NY	43.02746	-77.4784	101.5	11/27/1991	9/16/2005	378	1.702	1.625	57,676	49,851	1,178
0423205010	IRONDEQUOIT CR ABV BLOSSOM RD NR ROCHESTER NY	4140101	NY	43.14506	-77.5119	367.8	12/1/1980	8/31/2005	2944	1.859	1.800	213,124	138,625	2,349
0423205025	IRONDEQUOIT CREEK AT EMPIRE BLVD, ROCHESTER NY	4140101	NY	43.17567	-77.5266	391.1	6/28/1990	12/30/2002	1650	1.726	1.675	171,184	145,129	2,004
04260500	BLACK RIVER AT WATERTOWN NY	4150101	NY	43.98543	-75.9251	4,827.8	10/8/1970	8/9/1994	274	0.792	0.715	3,387,768	3,747,991	93,196
05046000	OTTER TAIL RIVER BL ORWELL D NR FERGUS FALLS, MN	9020103	MN	46.20961	-96.1849	4,506.6	4/18/1985	6/16/2003	61	0.888	0.825	505,737	906,276	33,762
05046450	OTTER TAIL RIVER AB BRECKENRIDGE, MN	9020103	MN	46.26163	-96.5462	4,382.1	5/20/1997	6/17/2003	48	0.794	0.795	503,079	1,518,588	49,793
05046502	OTTER TAIL RIVER AT 11TH ST IN BRECKENRIDGE, MN	9020103	MN	46.2744	-96.5801	4,416.0	9/11/2001	7/16/2007	47	1.157	0.898	667,984	1,556,326	109,104
05049000	MUSTINKA RIVER AB WHEATON, MN	9020102	MN	45.82079	-96.4906	1,731.8	6/1/1994	9/4/2003	31	2.000	1.785	1,139,547	1,565,081	120,993
05051300	BOIS DE SIOUX RIVER NEAR DORAN, MN	9020101	ND	46.1523	-96.5794	4,869.2	3/27/1993	5/24/2005	46	1.949	1.530	824,341	1,127,928	164,186
05051510	RED RIVER OF THE NORTH BELOW WAHPETON, ND	9020104	MN	46.37444	-96.658	10,411.8	5/13/1974	11/6/2006	235	1.475	1.230	1,225,503	2,877,840	87,016
05051522	RED RIVER OF THE NORTH AT HICKSON, ND	9020104	ND	46.65953	-96.7974	11,137.0	11/3/1975	5/12/2004	104	1.368	1.300	1,026,235	3,230,260	90,728
05053000	WILD RICE RIVER NR ABERCROMBIE, ND	9020105	ND	46.46758	-96.7837	5,387.2	6/1/1994	11/6/2006	15	1.357	1.370			
05053800	RED RIVER OF THE NORTH ABOVE FARGO, ND	9020104	ND	46.80385	-96.7968	14,660.2	4/4/1994	5/12/2004	43	1.419	0.928	1,274,734	7,124,520	295,859
05054000	RED RIVER OF THE NORTH AT FARGO, ND	9020104	ND	46.86106	-96.7837	17,612.0	5/8/2003	8/23/2005	30	0.893	0.896	1,793,033	7,157,404	282,236
05056000	SHEYENNE RIVER NR WARWICK, ND	9020203	ND	47.80515	-98.7165	5,361.3	7/14/1993	10/3/2006	31	1.367	1.275	212,082	481,773	18,329
05057000	SHEYENNE RIVER NR COOPERSTOWN, ND	9020203	ND	47.43274	-98.0277	16,757.3	3/6/1979	8/22/2005	82	1.597	1.500	537,820	1,388,285	66,091
05057200	BALDHILL CREEK NR DAZEY, ND	9020203	ND	47.22916	-98.1249	1,789.7	3/21/1979	8/8/1995	48	1.542	1.425	131,850	199,414	13,216
05058000	SHEYENNE RIVER BELOW BALDHILL DAM, ND	9020204	ND	47.03367	-98.0832	19,347.3	3/21/1979	8/22/2005	44	1.836	1.770	499,482	1,253,574	50,991
05058700	SHEYENNE RIVER AT LISBON, ND	9020204	ND	46.44673	-97.6795	21,212.1	2/9/1993	5/10/2005	29	1.398	1.340	754,453	1,971,290	86,852
05059000	SHEYENNE RIVER NEAR KINDRED, ND	9020204	ND	46.62888	-97.0042	22,792.0	7/27/1976	11/6/2006	231	1.351	1.223	673,895	2,843,764	51,400
05062500	WILD RICE RIVER AT TWIN VALLEY, MN	9020108	MN	47.26558	-96.2473	2,419.1	9/24/1974	4/30/1997	177	1.228	0.960	656,722	771,068	37,181
05064000	WILD RICE RIVER AT HENDRUM, MN	9020108	MN	47.26754	-96.7979	4,040.4	4/6/1976	5/12/2004	115	1.127	0.885	993,378	2,171,633	278,152
05064500	RED RIVER OF THE NORTH AT HALSTAD, MN	9020107	ND	47.35182	-96.8439	56,462.0	1/27/1978	8/23/2005	122	2.204	1.800	7,049,961	18,610,865	837,426
05064900	BEAVER CREEK NR FINLEY, ND	9020109	ND	47.59452	-97.7094	414.4	2/24/1981	8/5/1996	50	2.020	1.625	51,721	248,184	7,110
05076000	THIEF RIVER NEAR THIEF RIVER FALLS, MN	9020304	MN	48.18555	-96.1701	2,551.2	11/2/1992	8/17/2006	27	2.123	1.550	1,289,869	233,032	266,549
05079000	RED LAKE RIVER AT CROOKSTON, MN	9020303	MN	47.77596	-96.6099	13,649.3	11/15/1974	5/11/2005	158	1.304	1.060	2,077,298	2,427,927	168,981
05082500	RED RIVER OF THE NORTH AT GRAND FORKS, ND	9020301	ND	47.92705	-97.0287	77,959.0	2/8/1993	8/15/2005	60	1.326	1.150	7,122,290	24,682,490	614,907
05082625	TURTLE R AT TURTLE R STATE PARK NR ARVILLA, ND	9020307	ND	47.93195	-97.5148	805.5	2/8/1993	8/10/2000	83	1.343	1.139	82,163	161,984	7,866
05083500	RED RIVER OF THE NORTH AT OSLO, MN	9020306	ND	48.19419	-97.1411	80,808.0	3/15/1973	5/12/2004	53	1.831	1.600	7,816,398	25,678,431	2,578,141
05085000	FOREST RIVER AT MINTO, ND	9020308	ND	48.28611	-97.3679	1,916.6	4/20/1998	8/15/2005	21	1.587	1.435			
05090000	PARK RIVER AT GRAFTON, ND	9020310	ND	48.4247	-97.412	1,800.1	4/24/1998	8/15/2005	17	1.528	1.325			
05099400	LITTLE SOUTH PEMBINA RIVER NR WALHALLA, ND	9020313	ND	48.86517	-98.0068	471.4	4/1/1976	9/26/1979	51	1.539	0.830	343,723	242,717	52,078
05116500	DES LACS RIVER AT FOXHOLM, ND	9010002	ND	48.3705	-101.57	2,432.0	11/5/1981	9/18/2006	29	1.849	1.570	29,840	108,423	3,719
05124480	KAWISHIWI RIVER NEAR ELY, MN	9030001	MN	47.92327	-91.5342	657.9	3/16/1976	5/15/2001	68	0.543	0.470	74,974	94,077	7,992
052	MISSISSIPPI R SHIELY CO. DOCK, GREY CLOUD ISLAND	7010206	MN	44.80278	-93.0139	90,034.4	3/7/1975	9/15/1994	263	3.995	3.535	66,297,372	84,033,154	2,513,626
05267000	MISSISSIPPI RIVER NEAR ROYALTON, MN	7010201	MN	45.82612	-94.3566	30,044.0	1/22/1975	8/5/1998	169	0.944	0.850	5,290,842	5,175,290	197,270
05280000	CROW RIVER AT ROCKFORD, MN	7010204	MN	45.08659	-93.7341	6,837.6	10/17/1972	8/3/2006	122	3.235	2.500	6,897,255	8,558,005	434,255
05283500	MISSISSIPPI RIVER AT ANOKA, MN	7010206	MN	45.19163	-93.3947	44,755.2	1/3/1973	8/6/1998	50	1.662	1.430	15,478,917	19,392,501	1,479,255
05286000	RUM RIVER NEAR ST. FRANCIS, MN	7010207	MN	45.32757	-93.3727	3,522.4	10/19/1972	4/2/1997	50	1.241	1.130	929,167	1,423,834	59,921
05287890	ELM CREEK NR CHAMPLIN, MN	7010206	MN	45.1633	-93.4365	222.7	2/26/1988	9/8/2005	192	1.415	1.220	90,169	99,492	5,735

05288500	MISSISSIPPI RIVER NEAR ANOKA, MN	7010206	MN	45.12671	-93.2964	49,469.0	10/4/1972	8/7/1998	27	1.423	1.210	12,568,412	21,212,641	943,095
05291000	WHETSTONE RIVER NEAR BIG STONE CITY, SD	7020001	SD	45.29189	-96.4872	1,030.8	3/25/1974	1/13/2004	25	1.426	1.060			
05294000	POMME DE TERRE RIVER AT APPLETON, MN	7020002	MN	45.20355	-96.0195	2,344.0	9/28/1972	9/24/2007	239	1.941	1.718	629,696	1,600,587	34,307
05316500	REDWOOD RIVER NEAR REDWOOD FALLS, MN	7020006	MN	44.52366	-95.1717	1,629.1	6/20/1990	3/19/2007	38	7.199	6.020	3,518,193	1,749,996	423,223
05317000	COTTONWOOD RIVER NEAR NEW ULM, MN	7020008	MN	44.29119	-94.4405	3,367.0	8/23/1989	3/19/2007	60	13.204	13.185	19,514,672	5,275,736	2,924,614
05319500	WATONWAN RIVER NEAR GARDEN CITY, MN	7020010	MN	44.0465	-94.1948	2,204.1	9/14/1976	9/27/2007	248	6.313	5.100	8,469,064	4,033,280	467,862
05320270	LITTLE COBB RIVER NEAR BEAUFORD, MN	7020011	MN	43.99641	-93.9087	336.7	4/24/1996	9/7/2005	98	8.553	8.855	1,289,429	799,732	124,842
05322000	BLUE EARTH RIVER AT MOUTH AT MANKATO, MN	7020007	MN	44.1633	-94.0369	9,056.9	10/6/1970	9/4/2001	385	7.777	7.170	43,554,488	21,062,357	3,306,825
05325000	MINNESOTA RIVER AT MANKATO, MN	7020007	MN	44.16966	-94.0031	38,591.0	3/4/1994	7/20/1994	8	5.333	5.800			
05330000	MINNESOTA RIVER NEAR JORDAN, MN	7020012	MN	44.69314	-93.6419	41,958.0	11/21/1972	9/3/1998	233	5.627	4.900	57,973,318	57,504,892	4,797,526
05331000	MISSISSIPPI RIVER AT ST. PAUL, MN	7010206	MN	44.93309	-93.1046	95,312.0	10/4/1972	9/15/1994	338	3.510	2.765	77,294,691	81,738,431	3,486,034
05331570	MISSISSIPPI RIVER AT NININGER, MN	7010206	MN	44.77583	-92.8984	95,830.0	1/31/1978	9/8/1995	90	3.893	3.300	71,119,779	84,079,816	5,112,865
05331580	MISSISSIPPI RIVER BELOW L&D #2 AT HASTINGS, MN	7010206	MN	44.74663	-92.8524	96,089.0	10/2/1995	8/13/2004	96	3.947	3.110	55,556,420	84,216,511	3,793,319
05333500	ST. CROIX RIVER NEAR DANBURY, WI	7030001	WI	46.07524	-92.2477	4,092.2	10/23/1975	11/16/2004	120	0.467	0.450	597,852	773,041	22,837
05338500	SNAKE RIVER NEAR PINE CITY, MN	7030004	MN	45.84057	-92.9319	2,522.7	9/29/1971	11/21/2005	90	1.260	1.190	622,214	874,811	32,841
05340500	ST. CROIX RIVER AT ST. CROIX FALLS, WI	7030005	MN	45.40688	-92.6482	16,161.6	10/2/1974	5/8/2007	191	0.798	0.730	3,756,772	4,715,978	156,631
05355200	CANNON RIVER AT WELCH, MN	7040002	MN	44.5645	-92.7316	3,470.6	10/30/1991	8/21/2007	30	5.037	4.900	4,598,767	5,808,287	205,338
05367500	RED CEDAR RIVER NEAR COLFAX, WI	7050007	WI	45.05308	-91.7119	2,823.1	6/7/1990	10/15/2003	7	1.737	1.920			
05368000	HAY RIVER AT WHEELER, WI	7050007	WI	45.04785	-91.911	1,082.6	6/7/1990	10/17/2002	7	2.213	2.230			
05369500	CHIPPEWA RIVER AT DURAND, WI	7050005	WI	44.62891	-91.9702	23,335.9	10/2/1974	9/5/2007	324	1.353	1.300	9,448,735	9,731,797	206,501
05378183	JOOS VALLEY CREEK NEAR FOUNTAIN CITY, WI	7040003	WI	44.21479	-91.6651	15.3	10/8/2002	9/30/2007	175	1.466	1.460	5,051	18,661	0
05378185	EAGLE CK AT CT HIGHWAY G NEAR FOUNTAIN CITY, WI	7040003	WI	44.2094	-91.6784	37.0								
05378500	MISSISSIPPI RIVER AT WINONA, MN	7040003	MN	44.05672	-91.6367	153,328.0	11/7/1974	8/20/1986	83	2.187	2.050	89,632,662	112,067,420	6,234,739
05379500	TREMPEALEAU RIVER AT DODGE, WI	7040005	WI	44.1316	-91.5531	1,665.4	5/24/1978	9/18/2007	104	2.620	2.520	1,262,910	1,571,028	36,476
05382000	BLACK RIVER NEAR GALESVILLE, WI	7040007	WI	44.06063	-91.2872	5,387.2	10/6/1970	9/18/2007	263	1.375	1.262	2,400,693	3,591,524	87,739
05388250	Upper Iowa River near Dorchester, IA	7060002	IA	43.42111	-91.5088	1,994.3	10/15/1998	9/12/2007	105	5.680	5.400	4,840,980	3,471,979	262,995
054	MISSISSIPPI RIVER AT BR ON MN-25 AT MONTICELLO	7010203	MN	45.30886	-93.7919	34,018.7	9/30/1988	9/7/1994	55	1.268	1.200	7,618,599	9,649,688	335,791
05401050	TENMILE CREEK NEAR NEKOOSA, WI	7070003	WI	44.26262	-89.8106	189.8	11/15/1973	10/17/2002	43	3.220	3.500	252,784	218,198	17,723
05405000	BARABOO RIVER NEAR BARABOO, WI	7070004	WI	43.48174	-89.6365	1,577.3	8/6/2001	9/17/2007	67	2.179	2.085	964,230	1,581,511	44,072
05406491	GARFOOT CREEK NEAR CROSS PLAINS, WI	7070005	WI	43.10998	-89.6794	14.0	10/16/1984	5/22/1991	73	4.181	3.445	14,985	9,449	945
05406500	BLACK EARTH CREEK AT BLACK EARTH, WI	7070005	WI	43.13388	-89.7325	118.1	4/28/1976	10/13/2002	62	3.470	3.180	159,548	104,122	18,065
05407000	WISCONSIN RIVER AT MUSCODA, WI	7070005	WI	43.1995	-90.4433	26,936.0	10/24/1974	9/17/2007	221	1.445	1.400	12,571,988	23,116,104	343,386
05407500	KICKAPOO RIVER AT ONTARIO, WI	7070006	WI	43.71476	-90.5869	388.5	1/17/1974	9/7/1977	44	1.224	1.080	137,095	417,523	15,785
05412500	Turkey River at Garber, IA	7060004	IA	42.73972	-91.2616	4,001.6	10/30/1979	9/12/2007	150	6.891	6.250	10,185,345	7,662,893	405,493
05413500	GRANT RIVER AT BURTON, WI	7060003	WI	42.72037	-90.8195	696.7	11/19/1986	10/16/2003	53	4.359	4.200	1,143,483	924,022	96,476
05418500	Maquoketa River near Maquoketa, IA	7060006	IA	42.08363	-90.6325	4,022.3	12/9/1970	6/5/2002	66	5.361	4.795	13,490,087	8,087,238	2,741,451
05418950	APPLE RIVER NEAR ELIZABETH, IL	7060005	IL	42.31862	-90.2543	536.1								
05420500	Mississippi River at Clinton, IA	7080101	IL	41.77928	-90.2511	221,704.0	2/11/1974	9/7/2005	279	2.359	2.327	152,228,231	172,794,943	5,076,698
05420680	Wapsipinicon River near Tripoli, IA	7080102	IA	42.83627	-92.2571	896.1	4/18/1996	8/3/2004	73	5.894	5.224	2,969,160	2,317,584	431,381
05422000	Wapsipinicon River near De Witt, IA	7080103	IA	41.76649	-90.5347	6,050.2	11/25/1974	9/5/2007	156	6.544	6.546	17,425,917	14,978,090	903,339
05423000	WEST BRANCH ROCK R NEAR WAUPUN, WI	7090001	WI	43.66803	-88.6524	105.4	8/4/1998	6/28/2000	2	5.099	5.099			
05423510	WEST BRANCH ROCK R @ STATE HWY 49 NR WAUPUN,WI	7090001	WI	43.63383	-88.6846	292.7	8/4/1998	10/14/2002	7	7.684	7.320			
05424057	ROCK RIVER AT HORICON, WI	7090001	WI	43.4501	-88.6323	1,181.0	8/31/1998	8/17/1999	33	5.019	5.020	3,074,343	1,285,613	213,123
05424082	ROCK RIVER AT HUSTISFORD, WI	7090001	WI	43.34537	-88.598	1,323.5	4/6/1978	3/29/1979	28	2.223	1.780	457,550	1,287,647	30,328
05425500	ROCK RIVER AT WATERTOWN, WI	7090001	WI	43.18806	-88.7265	2,509.7	9/1/1998	11/30/2004	68	3.001	3.031	1,793,669	2,474,472	69,851
05426000	CRAWFISH RIVER AT MILFORD, WI	7090002	WI	43.09992	-88.8497	1,973.6	1/26/1977	10/14/2003	144	3.432	3.280	1,790,460	2,330,316	86,617
05427085	ROCK RIVER AT ROBERT STREET AT FORT ATKINSON, WI	7090001	WI	42.92751	-88.8429	5,801.6	10/6/1998	10/14/2003	37	3.462	3.230	5,381,688	6,358,860	615,216
05427570	ROCK RIVER AT INDIANFORD, WI	7090001	WI	42.80392	-89.0892	6,811.7	1/27/1977	8/17/1999	250	2.808	2.610	5,599,121	6,458,294	321,889
05430175	YAHARA RIVER NEAR FULTON, WI	7090001	WI	42.82649	-89.1725	1,341.6	7/26/1977	10/14/2003	67	5.058	4.550	1,798,276	938,853	155,268
05430500	ROCK RIVER AT AFTON, WI	7090001	WI	42.60937	-89.0704	8,650.6	10/28/1970	9/19/2007	268	3.507	3.400	7,896,048	7,823,759	208,437
05431486	TURTLE CK AT CARVERS ROCK ROAD NEAR CLINTON, WI	7090001	WI	42.59752	-88.829	515.4	9/2/1998	10/17/2002	39	8.818	8.065	1,015,170	747,406	86,191
05434500	PECATONICA RIVER AT MARTINTOWN, WI	7090003	WI	42.50961	-89.7993	2,678.1	12/8/1970	9/18/2007	93	5.164	5.235	4,848,288	3,741,782	185,229
05435500	PECATONICA RIVER AT FREEPORT, IL	7090003	IL	42.30304	-89.619	3,434.3	1/2/1980	1/2/1980	1	4.900	4.900			
05435800	PECATONICA RIVER AT HARRISON, IL	7090003	IL	42.42751	-89.1957	4,630.9	5/23/1984	8/8/1984	2	6.345	6.345			
05437500	ROCK RIVER AT ROCKTON, IL	7090005	IL	42.44956	-89.0697	16,480.2	6/6/1979	8/18/1999	208	5.471	5.375	28,129,748	19,539,017	935,792
05438250	COON CREEK AT RILEY, IL	7090006	IL	42.1828	-88.6413	220.4	8/11/1983	1/25/1984	5	5.187	4.715			
05438600	KISHWAUKEE R AB SOUTH BRANCH NR PERRYVILLE, IL	7090006	IL	42.2018	-88.9792	1,696.5	8/24/1983	2/22/1984	5	4.365	3.230			
05439500	SOUTH BRANCH KISHWAUKEE RIVER NR FAIRDALE IL	7090006	IL	42.11032	-88.9008	1,002.3	8/11/1983	10/24/1983	4	5.567	5.500			
05440000	KISHWAUKEE RIVER NEAR PERRYVILLE, IL	7090006	IL	42.19379	-89.0002	2,846.4	8/24/1983	8/16/1984	8	5.404	5.580			
05440700	ROCK RIVER AT BYRON, IL	7090005	IL	42.12273	-89.2557	19,456.8	5/24/1979	12/16/1998	175	5.522	5.300	30,692,599	27,537,115	649,446

05442200	ROCK RIVER AT GRAND DETOUR, IL	7090005	IL	41.88998	-89.4206	22,020.2	6/5/1979	12/16/1998	158	5.654	5.400	36,295,039	29,580,945	745,430
05443500	ROCK RIVER AT COMO, IL	7090005	IL	41.78184	-89.7504	22,670.3	4/10/1979	12/16/1998	161	5.515	5.300	37,333,510	29,594,642	851,452
05444000	ELKHORN CREEK NEAR PENROSE, IL	7090005	IL	41.90255	-89.6967	378.1	6/5/1985	9/2/1998	4	9.848	9.400			
05446500	ROCK RIVER NEAR JOSLIN, IL	7090005	IL	41.55601	-90.1853	24,731.9	11/13/1974	12/16/1998	232	4.981	4.800	43,213,306	33,638,066	1,642,484
05447500	GREEN RIVER NEAR GENESEO, IL	7090007	IL	41.48886	-90.1578	2,597.8	3/5/1980	12/16/1998	167	5.136	4.850	4,625,512	5,593,101	194,532
05449500	Iowa River near Rowan, IA	7080207	IA	42.75993	-93.6223	1,082.6	3/28/1983	11/7/2006	173	7.569	6.900	4,306,140	2,814,492	229,745
05451210	South Fork Iowa River NE of New Providence, IA	7080207	IA	42.31483	-93.1524	580.2	3/19/1996	6/2/2005	104	10.054	10.564	4,504,271	2,662,902	614,521
05453100	Iowa River at Marengo, IA	7080208	IA	41.81218	-92.0643	7,236.5	11/26/1974	9/4/1998	44	7.214	7.585	17,814,157	18,379,049	1,662,235
05455100	Old Mans Creek near Iowa City, IA	7080209	IA	41.60642	-91.6157	520.6	6/14/1995	9/4/2007	127	7.095	6.680	1,931,390	953,686	216,936
05458000	Little Cedar River near Ionia, IA	7080201	IA	43.03352	-92.5037	792.5	4/19/2001	12/8/2004	52	7.878	4.715	2,479,055	2,339,878	316,399
05458900	West Fork Cedar River at Finchford, IA	7080204	IA	42.62936	-92.5432	2,191.1	1/12/1978	9/4/2007	274	6.686	6.350	9,530,426	5,390,540	496,718
05463050	Cedar River at Cedar Falls, IA	7080205	IA	42.53935	-92.4497	12,261.1	7/22/1975	9/4/2007	198	6.221	5.750	35,003,486	31,343,258	1,716,289
05464020	Cedar River at Gilbertville, IA	7080205	IA	42.41582	-92.2188	13,571.6	10/6/1970	9/4/2007	206	6.687	6.310	40,681,940	34,184,896	1,417,382
05464220	Wolf Creek near Dysart, IA	7080205	IA	42.2517	-92.2988	774.4	3/21/1996	5/24/2004	56	9.625	10.000	1,400,470	1,536,475	218,333
05464760	Cedar River near Bertram, IA	7080206	IA	41.92572	-91.5502	18,013.5	7/22/1975	9/5/2007	193	7.532	7.300	46,220,984	41,228,792	2,051,776
05465500	Iowa River at Wapello, IA	7080209	IA	41.17835	-91.181	32,375.0	11/1/1983	9/1/2005	196	6.759	6.500	71,554,442	70,612,572	3,078,699
05466500	EDWARDS RIVER NEAR NEW BOSTON, IL	7080104	IL	41.18696	-90.9676	1,152.6								
05469000	HENDERSON CREEK NEAR OQUAWKA, IL	7080104	IL	41.00188	-90.8543	1,118.9	8/30/1978	4/4/1979	3	6.767	5.300			
05471500	South Skunk River near Oskaloosa, IA	7080105	IA	41.35524	-92.6571	4,234.7	10/11/1999	9/11/2007	94	7.768	6.890	15,567,627	8,801,156	2,054,478
05474000	Skunk River at Augusta, IA	7080107	IA	40.75317	-91.2761	11,168.1	1/31/1978	9/7/2005	193	6.362	6.370	22,983,187	19,943,203	2,221,234
05474500	Mississippi River at Keokuk, IA	7080104	IL	40.39247	-91.3718	308,210.0	3/17/1975	7/22/1999	138	3.551	3.560	309,898,256	326,532,187	24,311,408
05476000	DES MOINES RIVER AT JACKSON, MN	7100002	MN	43.61937	-94.9843	3,237.5	10/12/1972	6/29/1976	20	3.874	3.100			
05481650	Des Moines River near Saylorville, IA	7100004	IA	41.68033	-93.6688	15,128.2	7/14/1975	9/6/2007	126	6.915	6.970	40,997,273	28,308,009	3,486,005
05484500	Raccoon River at Van Meter, IA	7100006	IA	41.53414	-93.9499	8,912.2	7/14/1975	9/6/2007	278	7.887	7.700	30,097,735	22,699,689	2,021,191
05486000	North River near Norwalk, IA	7100008	IA	41.45784	-93.655	903.9	5/4/1975	9/6/2007	253	5.298	4.900	1,630,697	1,172,338	133,040
05490600	Des Moines River at St. Francisville, MO	7100009	IA	40.46211	-91.5671	37,037.0	10/19/1970	11/2/2006	274	5.416	5.100	68,149,107	62,953,615	4,507,982
05495000	Fox River at Wayland, MO	7110001	MO	40.39215	-91.5981	1,036.0	10/19/1970	9/7/2005	57	1.663	1.090	892,330	1,153,492	210,996
05495500	BEAR CREEK NEAR MARCELLINE, IL	7110001	IL	40.14358	-91.3415	903.9	5/17/1978	9/11/1998	9	2.645	2.400			
05500000	South Fabius River near Taylor, MO	7110003	MO	39.89678	-91.5799	1,605.8	7/18/1972	9/7/2005	152	1.554	1.310	1,024,384	1,647,636	89,355
05514500	Cuivre River near Troy, MO	7110008	MO	39.00937	-90.9764	2,338.8	7/19/1972	9/7/2005	113	1.730	1.400	2,070,048	2,993,562	196,697
05518000	KANKAKEE RIVER AT SHELBY, IN	7120001	IN	41.18248	-87.3435	4,607.6	1/6/1976	2/15/2006	292	2.507	2.050	4,852,586	9,163,068	202,785
05520500	KANKAKEE RIVER AT MOMENCE, IL	7120001	IL	41.15944	-87.6682	5,941.5	9/18/1979	2/21/2001	223	2.562	2.100	5,828,965	12,219,545	351,374
05525000	IROQUOIS RIVER AT IROQUOIS, IL	7120002	IL	40.82321	-87.5818	1,776.7	8/2/1978	7/10/1987	7	2.786	2.100			
05525500	SUGAR CREEK AT MILFORD, IL	7120002	IL	40.63164	-87.7243	1,155.1	8/2/1978	12/15/2004	80	7.106	6.941	5,891,345	2,565,899	816,991
05526000	IROQUOIS RIVER NEAR CHEBANSE, IL	7120002	IL	41.00908	-87.8253	5,415.7	12/5/1979	2/21/2001	93	7.517	7.700	25,660,142	15,009,744	3,551,256
05527500	KANKAKEE RIVER NEAR WILMINGTON, IL	7120001	IL	41.34607	-88.1881	13,338.5	12/14/1977	12/2/1998	204	4.409	4.100	30,904,139	32,021,261	1,288,347
05527800	DES PLAINES RIVER AT RUSSELL, IL	7120004	IL	42.48755	-87.9255	318.6	8/12/1981	8/9/2001	42	3.568	3.321	324,724	594,333	31,560
05528000	DES PLAINES RIVER NEAR GURNEE, IL	7120004	IL	42.34398	-87.9406	600.9	12/20/1977	2/8/1984	39	6.968	6.430	1,169,143	1,111,526	55,406
05529000	DES PLAINES RIVER NEAR DES PLAINES, IL	7120004	IL	42.08194	-87.8903	932.4	8/12/1981	7/27/1988	4	5.730	5.250			
05530590	DES PLAINES RIVER NEAR SCHILLER PARK, IL	7120004	IL	41.95333	-87.8546	1,150.0	10/15/1979	11/30/1998	176	6.164	5.800	2,198,048	2,240,312	59,760
05531500	SALT CREEK AT WESTERN SPRINGS, IL	7120004	IL	41.82604	-87.9	297.9	11/15/1979	8/17/2005	272	8.858	8.326	1,020,935	856,599	25,360
05532500	DES PLAINES RIVER AT RIVERSIDE, IL	7120004	IL	41.82278	-87.8201	1,631.7	4/9/1987	8/18/2004	189	6.365	6.004	3,391,861	3,327,751	96,191
05534050	DES PLAINES RIVER AT LOCKPORT, IL	7120004	IL	41.59642	-88.0686	1,813.0	8/13/1981	10/12/1995	32	6.171	5.400	3,839,993	15,170,819	317,797
05536195	LITTLE CALUMET RIVER AT MUNSTER, IND.	7120003	IN	41.57715	-87.5223	233.1	1/13/1971	2/6/2006	390	5.567	5.100	290,552	222,916	11,788
05536995	CHICAGO SANITARY AND SHIP CANAL AT ROMEOVILLE, IL	7120004	IL	41.64088	-88.06	1,914.0	4/9/1987	2/21/2001	91	7.172	7.121	16,766,922	10,181,310	830,581
05539000	HICKORY CREEK AT JOLIET, IL	7120004	IL	41.51445	-88.0741	278.4	8/13/1981	11/26/1984	8	4.055	4.150			
05539900	WEST BRANCH DU PAGE RIVER NEAR WEST CHICAGO, IL	7120004	IL	41.91086	-88.1792	73.8	8/12/1981	7/29/1988	6	8.742	8.050			
05540095	WEST BRANCH DU PAGE RIVER NEAR WARRENVILLE, IL	7120004	IL	41.82189	-88.1724	234.1	12/19/1977	2/7/1984	39	6.501	6.250	554,148	603,291	19,261
05540210	EAST BRANCH DU PAGE RIVER AT ROUTE 34 AT LISLE, IL	7120004	IL	41.80059	-88.0815	133.1	8/13/1981	9/17/1997	8	8.440	8.300			
05540290	DU PAGE RIVER NEAR NAPERVILLE, IL	7120004	IL	41.69003	-88.1662	569.8				6.453	6.450			
05540500	DU PAGE RIVER AT SHOREWOOD, IL	7120004	IL	41.5223	-88.1932	839.2	8/13/1981	9/12/1990	51	8.342	8.100	2,882,749	2,351,167	102,421
05542000	MAZON RIVER NEAR COAL CITY, IL	7120005	IL	41.28711	-88.3606	1,178.5	8/3/1988	9/12/1990	10	11.195	9.300			
05543500	ILLINOIS RIVER AT MARSEILLES, IL	7120005	IL	41.3259	-88.7181	21,390.8	11/13/1974	12/15/1998	287	6.001	6.000	50,939,066	67,140,287	1,771,681
055451345	WHITE RIVER AT CENTER STREET AT LAKE GENEVA, WI	7120006	WI	42.59057	-88.4337	75.1								
05546700	FOX RIVER NEAR CHANNEL LAKE, IL	7120006	IL	42.47919	-88.1783	2,255.9	3/14/1979	12/1/1998	189	3.466	3.160	2,051,983	2,013,632	56,182
05548280	NIPPERSINK CREEK NEAR SPRING GROVE, IL	7120006	IL	42.44352	-88.2472	497.3	3/14/1979	12/1/1998	185	4.228	4.000	552,749	609,010	12,449
05549600	FOX RIVER AT BURTONS BRIDGE, IL	7120006	IL	42.27948	-88.2271	3,232.1	7/26/1979	12/17/1998	167	3.230	2.900	2,644,254	2,513,766	88,367
05550000	FOX RIVER AT ALGONQUIN, IL	7120006	IL	42.16596	-88.2904	3,633.8	3/13/1979	12/7/1998	197	3.076	2.900	2,863,522	2,810,948	84,226
05551000	FOX RIVER AT SOUTH ELGIN, IL	7120007	IL	41.9962	-88.2941	4,030.0	3/13/1979	12/7/1998	182	3.559	3.400	3,531,301	3,393,210	100,629
05551700	BLACKBERRY CREEK NEAR YORKVILLE, IL	7120007	IL	41.67123	-88.4462	181.8	2/6/1979	12/10/1998	182	4.066	3.800	223,053	417,496	9,288

05551995	SOMONAUK CREEK AT SHERIDAN, IL	7120007	IL	41.54361	-88.6867	215.7	4/1/1981	12/10/1998	158	5.113	4.650	351,246	544,038	24,222
05552500	FOX RIVER AT DAYTON, IL	7120007	IL	41.38325	-88.7909	6,843.4	4/25/1979	1/13/1998	207	4.933	4.600	9,682,444	11,005,089	354,950
05553500	ILLINOIS RIVER AT OTTAWA, IL	7130001	IL	41.34199	-88.8467	28,357.9	4/11/1989	8/4/2004	101	5.834	5.956	72,746,226	78,468,104	3,110,969
05554490	VERMILION RIVER AT MC DOWELL, IL	7130002	IL	40.83059	-88.5748	1,427.1	8/14/1978	8/11/1997	6	2.991	3.200			
05555300	VERMILION RIVER NEAR LEONORE, IL	7130002	IL	41.20959	-88.9373	3,240.1	4/16/1986	4/16/1986	1	9.400	9.400			
05556200	ILLINOIS RIVER AT HENNEPIN	7130001	IL	41.25788	-89.3488	33,143.2	4/12/1989	9/13/1990	11	6.245	6.200			
05556500	BIG BUREAU CREEK AT PRINCETON, IL	7130001	IL	41.36494	-89.4976	507.6	6/24/1986	8/11/1997	2	7.359	7.359			
05558995	ILLINOIS RIVER AT LACON, IL	7130001	IL	41.02504	-89.4173	35,394.9	4/12/1989	9/13/1990	11	6.064	5.200			
05559900	ILLINOIS RIVER AT WATER COMPANY AT PEORIA, IL	7130001	IL	40.72493	-89.5473	36,001.0	11/17/1970	12/15/1998	209	5.984	6.000	75,951,218	92,922,023	3,464,177
05563800	ILLINOIS RIVER AT PEKIN, IL	7130003	IL	40.57309	-89.6548	36,555.1	10/14/1977	12/14/1998	180	5.833	5.800	78,205,238	66,214,555	2,111,223
05567510	MACKINAW RIVER BELOW CONGERVILLE, IL	7130004	IL	40.58672	-89.2785	2,009.8								
05568005	MACKINAW RIVER BELOW GREEN VALLEY, IL	7130004	IL	40.44754	-89.6912	2,828.3								
05568800	INDIAN CREEK NEAR WYOMING, IL	7130005	IL	41.01846	-89.8359	162.4	10/18/1996	9/24/1998	29	8.973	8.621	607,719	403,446	57,829
05568915	SPOON RIVER NEAR DAHINDA, IL	7130005	IL	40.90791	-90.0868	1,976.4								
05569500	SPOON RIVER AT LONDON MILLS, IL	7130005	IL	40.70676	-90.2829	2,776.5	8/31/1978	8/31/1978	1	1.400	1.400			
05570000	SPOON RIVER AT SEVILLE, IL	7130005	IL	40.48497	-90.3422	4,236.7	8/30/1978	8/13/1993	44	5.587	5.900	10,008,047	7,738,502	1,317,538
05570520	ILLINOIS RIVER AT POWER COMPANY AT HAVANA, IL	7130003	IL	40.28032	-90.0815	47,397.0	4/24/1978	11/21/1978	9	5.429	5.900			
05570910	SANGAMON RIVER AT FISHER, IL	7130006	IL	40.31119	-88.3227	621.6	1/17/1979	12/30/1998	186	8.224	9.110	3,320,301	1,278,570	297,136
05572000	SANGAMON RIVER AT MONTICELLO, IL	7130006	IL	40.02573	-88.5891	1,424.5	5/14/1981	8/16/2005	113	7.382	7.663	4,909,010	3,037,949	446,662
05572125	SANGAMON R AT ALLERTON PARK NEAR MONTICELLO, IL	7130006	IL	40.00226	-88.6353	1,489.4	10/19/1978	12/21/1998	183	7.662	8.140	4,791,080	3,088,855	336,452
05573504	SANGAMON R. AT L DECATUR WATER INTAKE AT DECATUR	7130006	IL	39.82893	-88.9597	2,403.6	10/10/1984	12/1/1998	119	5.803	6.500	5,843,956	4,151,311	596,320
05573540	SANGAMON RIVER AT ROUTE 48 AT DECATUR, IL	7130006	IL	39.83094	-88.9763	2,429.4				4.820	2.400			
05573650	SANGAMON RIVER NEAR NIANTIC, IL	7130006	IL	39.79723	-89.1054	2,737.8	7/22/1996	7/22/1996	1	8.156	8.200			
05573800	SANGAMON RIVER AT ROBY, IL	7130006	IL	39.74227	-89.3992	3,273.8				8.585	9.200			
05575500	SOUTH FORK SANGAMON RIVER AT KINCAID, IL	7130007	IL	39.57884	-89.3925	1,455.6	10/13/1977	11/24/1998	191	5.504	5.400	1,822,643	2,781,984	81,663
05576022	SOUTH FORK SANGAMON RIVER BELOW ROCHESTER, IL	7130007	IL	39.75411	-89.5662	2,253.3	8/14/1978	9/27/1982	14	4.785	4.600			
05576500	SANGAMON RIVER AT RIVERTON, IL	7130008	IL	39.84754	-89.55	6,780.6	1/9/1986	12/2/1998	114	6.663	6.800	14,500,711	13,418,582	800,170
05577505	SPRING CR AT BURNS LANE BRIDGE AT SPRINGFIELD,IL	7130008	IL	39.82055	-89.6877	282.3	7/23/1981	8/21/1996	2	1.525	1.525			
05578000	SANGAMON RIVER AT PETERSBURG, IL	7130008	IL	40.01102	-89.844	7,933.2	5/1/1978	8/27/1996	12	5.549	5.350			
05578500	SALT CREEK NEAR ROWELL, IL	7130009	IL	40.11457	-89.0505	867.7	8/15/1978	10/4/1982	15	4.953	4.600			
05579500	LAKE FORK NEAR CORNLAND, IL	7130009	IL	39.95123	-89.3841	554.3	6/7/1978	10/4/1982	15	6.879	7.350			
05580000	KICKAPOO CREEK AT WAYNESVILLE, IL	7130009	IL	40.25517	-89.1283	587.9	8/15/1978	8/14/1997	13	6.263	3.100			
05580500	KICKAPOO CREEK NEAR LINCOLN, IL	7130009	IL	40.1864	-89.3668	792.5	9/18/1978	10/5/1982	14	6.893	7.400			
05582000	SALT CREEK NEAR GREENVIEW, IL	7130009	IL	40.13304	-89.7367	4,672.4	5/1/1978	10/7/1982	19	5.712	6.400			
05583000	SANGAMON RIVER NEAR OAKFORD, IL	7130008	IL	40.12302	-89.9852	13,190.9	12/20/1977	11/30/1998	223	5.609	5.700	27,449,997	26,120,488	908,985
05584500	LA MOINE RIVER AT COLMAR, IL	7130010	IL	40.33088	-90.896	1,696.5	2/17/1975	12/16/1998	357	4.965	5.300	3,971,357	2,712,370	319,641
05585000	LA MOINE RIVER AT RIPLEY, IL	7130010	IL	40.02384	-90.6308	3,348.9	7/16/1975	12/14/1998	284	4.268	4.200	5,682,330	4,924,044	302,341
05586100	ILLINOIS RIVER AT VALLEY CITY, IL	7130011	IL	39.70341	-90.6462	69,267.0	12/12/1974	8/18/2005	371	5.452	5.500	121,688,706	113,278,137	3,126,464
05587000	MACOUPIN CREEK NEAR KANE, IL	7130012	IL	39.23422	-90.3946	2,248.1	6/6/1978	8/7/1997	7	1.697	1.700			
05587455	MISSISSIPPI RIVER BELOW GRAFTON, IL	7110009	IL	38.95033	-90.3715	443,667.0	5/4/1989	9/12/2005	183	3.897	3.875	479,811,492	532,032,940	16,028,600
05587900	CAHOKIA CREEK AT EDWARDSVILLE, IL	7140101	IL	38.82447	-89.975	549.1	6/12/1984	9/15/1998	6	1.644	1.250			
05591200	KASKASKIA RIVER AT COOKS MILLS, IL	7140201	IL	39.58315	-88.4132	1,225.1	12/1/1977	12/3/1998	194	7.841	8.590	5,318,485	3,243,366	330,282
05591300	KASKASKIA RIVER AT ALLENVILLE, IL	7140201	IL	39.57281	-88.5322	1,310.5	1/16/1980	12/3/1998	174	7.793	9.300	5,719,742	3,380,958	366,809
05591400	JONATHAN CREEK NEAR SULLIVAN, IL	7140201	IL	39.60111	-88.5461	141.7	3/3/1980	12/7/1998	154	8.902	10.000	612,134	356,300	59,225
05591700	WEST OKAW RIVER NEAR LOVINGTON, IL	7140201	IL	39.73133	-88.6623	290.1	4/7/1980	12/7/1998	153	9.576	10.300	1,390,984	693,300	82,398
05592000	KASKASKIA RIVER AT SHELBYVILLE, IL	7140201	IL	39.39884	-88.7881	2,729.9	10/31/1977	12/15/1998	197	4.898	4.480	4,246,542	4,500,772	187,891
05592100	KASKASKIA RIVER NEAR COWDEN, IL	7140201	IL	39.22962	-88.8449	3,444.7	10/17/1977	12/21/1998	196	4.542	4.050	5,424,519	5,680,754	223,110
05592195	BECK CREEK AT HERRICK, IL	7140201	IL	39.21639	-89.0206	251.2	7/25/1983	1/12/1998	4	2.223	1.835			
05592500	KASKASKIA RIVER AT VANDALIA, IL	7140202	IL	38.95644	-89.0877	5,024.6	12/12/1979	12/22/1998	178	3.915	2.950	7,330,351	7,662,662	363,124
05592600	HICKORY CREEK NEAR BLUFF CITY, IL	7140202	IL	38.92525	-89.0393	201.5	1/19/1989	12/22/1998	74	1.294	0.975	117,270	221,942	28,475
05592800	HURRICANE CREEK NEAR MULBERRY GROVE, IL	7140202	IL	38.91851	-89.2428	393.7	12/12/1979	12/22/1998	174	1.731	1.390	464,889	534,017	44,904
05592900	EAST FORK KASKASKIA RIVER NEAR SANDOVAL, IL	7140202	IL	38.69236	-89.0999	292.7	12/12/1979	12/17/1998	164	1.676	1.170	270,681	366,742	26,328
05592930	NORTH FORK KASKASKIA RIVER NEAR PATOKA, IL	7140202	IL	38.77361	-89.0861	101.3	1/19/1989	12/17/1998	74	2.039	1.590	75,800	107,353	14,291
05593010	KASKASKIA RIVER BELOW CARLYLE, IL	7140202	IL	38.57449	-89.3693	7,081.1	8/13/1979	12/15/1998	177	1.938	1.555	4,274,571	7,435,624	239,312
05593505	CROOKED CREEK NEAR ODIN, IL	7140202	IL	38.56389	-89.0503	231.0	4/20/1982	11/14/1996	5	2.825	2.150			
05594000	SHOAL CREEK NEAR BREESE, IL	7140203	IL	38.60959	-89.4945	1,903.7	4/28/1982	11/21/1996	6	1.878	2.000			
05594100	KASKASKIA RIVER NEAR VENEDY STATION, IL	7140204	IL	38.44995	-89.6298	11,377.9	11/12/1974	11/18/1998	232	2.191	1.850	8,983,397	13,617,597	427,609
05594450	SILVER CREEK NEAR TROY, IL	7140204	IL	38.7152	-89.8304	398.9	4/21/1982	11/19/1996	5	2.870	3.100			
05594800	SILVER CREEK NEAR FREEBURG, IL	7140204	IL	38.39653	-89.873	1,201.8	4/27/1982	11/20/1996	6	3.500	3.300			
05595200	RICHLAND CREEK NEAR HECKER, IL	7140204	IL	38.32375	-89.9711	334.1	4/27/1982	11/20/1996	6	9.080	9.100			

05595700	BIG MUDDY RIVER NEAR MT. VERNON, IL	7140106	IL	38.30935	-88.9887	186.2	10/2/1985	12/2/1998	119	1.578	1.220	196,967	158,334	30,069
05595730	RAYSE CREEK NEAR WALTONVILLE, IL	7140106	IL	38.25381	-89.0399	227.9	12/20/1979	12/2/1998	161	2.222	1.540	324,890	249,618	36,718
05595830	CASEY FORK AT ROUTE 37 NEAR MT. VERNON, IL	7140106	IL	38.26947	-88.8989	227.1	12/18/1979	12/2/1998	161	4.719	3.100	174,353	189,280	17,782
05597000	BIG MUDDY RIVER AT PLUMFIELD, IL	7140106	IL	37.90148	-89.0143	2,056.5	11/17/1981	3/5/1996	6	1.294	1.200			
05597500	CRAB ORCHARD CREEK NEAR MARION, IL	7140106	IL	37.73092	-88.8895	82.1	6/4/1979	3/6/1996	25	1.004	1.000			
05599500	BIG MUDDY RIVER AT MURPHYSBORO, IL	7140106	IL	37.74924	-89.3471	5,617.7	11/11/1974	12/1/1998	229	1.491	1.400	2,797,891	4,995,011	155,676
07014500	Meramec River near Sullivan, MO	7140102	MO	38.15847	-91.1086	3,820.3	10/22/1970	9/1/2005	200	0.427	0.389	777,228	1,957,528	79,384
07016400	Bourbeuse River above Union, MO	7140103	MO	38.432	-91.0199	2,092.7	10/22/1970	9/7/2005	153	0.604	0.480	928,806	1,418,085	128,860
07018100	Big River near Richwoods, MO	7140104	MO	38.15988	-90.7055	1,903.7	11/9/1992	9/1/2005	75	0.432	0.340	633,909	929,960	97,841
07019280	Meramec River at Paulina Hills, MO	7140102	MO	38.46283	-90.4148	10,152.8	10/22/1970	9/8/2005	198	0.776	0.755	3,354,755	5,952,507	201,955
090162	SAGINAW R AT MIDLAND ST BR; BANGOR TWP, SEC 21	4080206	MI	43.60139	-83.8919	15,845.6	1/29/1974	6/18/1996	113	2.942	2.700	11,228,510	16,254,027	549,546
093051	CHIPPEWA R @ NSP HOLCOMBE DM TAILRACE	7050005	WI	45.22441	-91.1281	11,923.6	2/10/1977	6/6/2001	148	0.805	0.770	2,876,410	2,808,313	115,700
10070001	Beaver Creek near Cedar Falls	7080205	IA	42.5732	-92.5065	1,009.2	10/26/1998	9/4/2007	109	8.922	7.900	4,105,467	2,748,383	241,002
10070002	Wolf Creek at La Porte City	7080205	IA	42.3158	-92.1936	847.6	6/5/2001	9/4/2007	86	9.438	8.700	1,938,002	1,690,519	150,421
10070004	Black Hawk Creek at Waterloo	7080205	IA	42.4695	-92.4134	848.1	10/2/2001	9/4/2007	78	9.625	8.800	2,709,175	1,998,550	244,190
10090001	Cedar River near Janesville	7080201	IA	42.6496	-92.4664	4,287.4	10/26/1998	9/10/2007	99	6.881	6.000	11,847,419	11,882,846	632,162
10120001	Shell Rock River at Shell Rock	7080202	IA	42.7116	-92.5812	4,427.4	10/25/1999	9/10/2007	114	6.426	5.515	10,306,608	10,852,463	447,180
10160001	Cedar River at Cedar Bluff	7080206	IA	41.78801	-91.3124	18,117.6	10/19/1999	7/5/2006	82	7.086	6.495	46,503,997	41,729,486	2,512,299
10170002	Winnebago River Upstream of Mason City	7080203	IA	43.1929	-93.2103	1,162.4	11/11/1999	9/10/2007	97	6.338	5.520	3,109,493	3,108,711	225,829
10170003	Winnebago River Downstream of Mason City	7080203	IA	43.1257	-93.1231	1,595.9	11/11/1999	9/10/2007	86	6.625	6.320	3,981,120	4,078,735	274,302
10220002	Volga River near Elkport	7060004	IA	42.7497	-91.2766	1,042.7	10/7/1999	9/12/2007	100	6.062	5.210	1,836,345	1,662,577	100,396
10250001	South Raccoon River near Redfield	7100007	IA	41.5798	-94.1827	2,512.2	10/20/1999	9/6/2007	100	6.144	4.760	5,695,934	5,179,142	661,608
103094	BLACK RIVER	7040007	WI	44.57076	-90.5952	1,717.2	3/30/1978	9/3/2003	148	1.457	1.245	995,962	1,817,754	87,277
10340001	Cedar River near Charles City	7080201	IA	43.00726	-92.6029	2,833.8	1/12/1971	9/10/2007	317	6.940	6.470	8,424,711	7,854,614	386,326
10370001	North Raccoon River near Jefferson	7100006	IA	42.0119	-94.4286	4,091.6	10/20/1999	11/13/2006	90	10.234	9.760	19,416,412	11,407,053	1,917,760
10400001	Boone River near Stratford	7100005	IA	42.3411	-93.892	2,286.1	10/18/1999	9/10/2007	90	9.661	9.550	13,029,519	5,776,260	2,072,635
10420001	Iowa River near Gifford	7080207	IA	42.3102	-93.0755	2,039.0	10/7/1999	9/12/2007	92	7.838	7.015	6,042,843	5,785,759	591,338
10440001	Cedar Creek near Oakland Mills	7080107	IA	40.9254	-91.6735	1,385.9	10/12/1998	9/4/2007	89	5.559	5.100	3,296,643	2,458,010	467,372
10460001	West Fork Des Moines River near Humboldt	7100002	IA	42.6744	-94.2069	5,926.7	6/5/1973	9/11/2007	119	6.123	6.100	15,309,076	9,189,744	1,015,373
10490001	North Fork Maquoketa River near Hurstville	7060006	IA	42.09025	-90.6723	1,527.3	10/13/1998	9/5/2007	107	7.950	7.500	3,674,899	2,696,566	151,866
10490002	Maquoketa River near Maquoketa	7060006	IA	42.0757	-90.6823	2,470.8	10/19/1999	9/5/2007	95	7.505	7.090	6,186,821	5,117,451	309,609
10500001	Indian Creek near Colfax	7080105	IA	41.74621	-93.2264	1,038.0	10/18/1999	9/12/2007	94	7.343	7.900	4,059,564	2,324,131	675,670
10520002	Iowa River Upstream of Iowa City	7080209	IA	41.6937	-91.5466	8,122.5	11/30/1999	9/5/2007	86	6.508	6.470	13,762,050	17,899,139	1,362,674
10520003	Iowa River Downstream of Iowa City	7080209	IA	41.5547	-91.5186	8,565.0	11/30/1999	9/5/2007	86	6.699	6.495	16,065,670	18,574,352	1,470,360
10540001	North Skunk River	7080106	IA	41.28387	-92.2574	1,649.6	10/12/1998	9/11/2007	97	6.413	6.400	4,064,430	2,749,653	488,590
10550001	East Fork of The Des Moines River near St. Joseph	7100003	IA	42.95138	-94.2335	2,432.9	10/12/1998	9/11/2007	107	8.274	8.700	12,351,056	4,995,018	1,047,974
10570002	Cedar River Upstream of Cedar Rapids	7080205	IA	42.0692	-91.7847	16,245.2	11/3/1999	9/5/2007	85	7.070	6.670	39,262,615	38,983,624	3,296,041
10630001	Whitebreast Creek near Knoxville	7100008	IA	41.3026	-93.1713	917.6	10/26/1999	8/1/2007	77	2.260	1.800	720,382	804,404	125,820
10630002	Cedar Creek near Bussey	7100009	IA	41.2191	-92.9077	965.0	10/25/1999	9/5/2007	97	2.224	1.115	871,687	931,172	129,987
10640002	Iowa River Downstream of Marshalltown	7080208	IA	42.0508	-92.8464	4,254.3	1/27/1976	9/12/2007	117	9.128	8.690	13,443,297	13,743,744	770,970
10640003	Iowa River Upstream of Marshalltown	7080208	IA	42.0917	-93.0002	3,817.6	10/11/1999	9/12/2007	84	9.040	8.050	12,173,955	12,962,473	1,103,467
10700001	Cedar River near Conesville	7080206	IA	41.4093	-91.29	19,949.4	10/14/1999	9/4/2007	95	7.061	6.800	51,307,236	45,164,546	2,684,740
10770001	Beaver Creek near Grimes	7100004	IA	41.6883	-93.7353	969.3	10/11/1999	9/6/2007	95	8.535	8.600	3,778,604	2,125,875	535,276
10770003	Des Moines River Downstream of Des Moines	7100008	IA	41.4877	-93.3384	29,882.3	11/15/1999	9/6/2007	86	7.926	7.850	55,980,259	59,988,418	4,092,720
10850003	South Skunk River Upstream of Ames	7080105	IA	42.06631	-93.6207	822.3	10/7/1999	9/12/2007	82	12.298	13.350	5,512,559	1,707,551	928,307
10900002	Des Moines River Downstream of Ottumwa	7100009	IA	40.95677	-92.34	34,441.8	11/2/1999	9/11/2007	86	6.555	6.450	54,153,986	60,820,395	3,767,212
10900003	Des Moines River Upstream of Ottumwa	7100009	IA	41.0874	-92.5258	33,982.4	11/2/1999	9/11/2007	84	6.639	6.600	53,926,040	60,400,495	3,677,836
10910001	Middle River near Indianola	7100008	IA	41.4247	-93.5871	1,269.3	10/15/1998	9/6/2007	85	3.775	2.950	2,056,745	1,656,525	363,880
10940002	Des Moines River Upstream of Fort Dodge	7100004	IA	42.5436	-94.2163	9,531.0	10/19/1999	9/10/2007	85	7.451	7.400	33,487,955	17,211,299	3,012,123
123016	Mississippi R. at LD 9	7060001	WI	43.21167	-91.1025	166,212.4	2/3/1977	9/4/2007	313	2.301	2.250	104,399,453	124,577,616	3,847,586
123017	Kickapoo R. at Stueben	7070006	WI	43.18214	-90.8506	1,773.6	2/3/1977	9/20/2007	282	1.442	1.330	735,083	1,563,151	18,070
130001	Maquoketa River	7060006	IA	42.18667	-90.3106	4,818.0	5/5/1993	11/8/2005	279	7.006	6.551	11,086,312	9,057,292	464,063
130003	APPLE RIVER NEAR HANOVER, IL	7060005	IL	42.18718	-90.236	657.4	5/3/1993	11/8/2005	237	4.247	4.182	1,123,465	819,109	75,618
130202	BATTLE CREEK R@ 9 MILE RD; PENNFIELD TWP SEC 21	4050003	MI	42.36417	-85.1222	536.5	10/8/1979	9/21/1993	149	1.886	1.640	368,217	527,743	12,120
133416	SUGAR RIVER AT STH 69 DWNSTM BRIDGE	7090004	WI	42.94904	-89.5445	212.4	5/27/1997	12/5/2000	48	6.478	6.440	635,266	272,682	52,815
133417	SUGAR RIVER AT VALLEY RD.	7090004	WI	42.9725	-89.5667	120.7	5/27/1997	12/5/2000	49	6.103	5.980	217,023	149,973	10,515
140001	Wapsipinicon River	7080103	IA	41.7273	-90.3646	6,524.4	8/8/1978	11/8/2005	281	5.867	5.801	17,370,872	16,343,047	840,109
160376	E BR DUPAGE R .5 MI S NAPERVILLE T37N R10E NE7	7120004	IL	41.71111	-88.1278	194.2	3/24/1983	9/17/1997	8	8.093	7.020			
160383	E BR DUPAGE R MAPLE AV LISLE T38N R10E SE10	7120004	IL	41.78571	-88.0792	136.9	3/25/1983	9/17/1997	8	8.172	7.215			
163001	ST. LOUIS RIVER AT OLIVER	4010201	WI	46.65669	-92.2004	9,114.0	7/11/1972	7/17/1990	183	1.078	1.050	2,537,362	1,439,049	72,982

163002	BOIS BRULE RIVER	4010301	WI	46.67936	-91.5949	466.6	7/31/1973	7/24/2007	53	0.402	0.361	82,641	118,720	10,298
171408	MISSISSINEWA RIVER AT PERU MILE POINT 0.8	5120103	IN	40.75389	-86.0231	2,107.3	10/14/1981	5/3/1995	10	4.485	4.725			
171420	ST. MARY'S RIVER NEAR FT WAYNE AT MI PT 10.83	4100004	IN	40.988	-85.099	1,800.1	2/22/1977	12/10/1985	59	5.059	4.150	8,290,047	11,406,774	1,286,852
171433	WABASH R AT US 27 BRIDGE N OF GENEVA, MI PT451.9	5120101	IN	40.61667	-84.9542	1,211.3	3/4/1980	11/5/1987	70	5.136	3.900	4,047,143	6,749,526	649,484
173051	RED CEDAR R @ CTH Y BRIDGE	7050007	WI	44.71927	-91.9034	4,852.4	8/5/1999	8/5/1999	1	1.290	1.290			
173208	Red Cedar R. at Menomonie	7050007	WI	44.88336	-91.9319	4,627.8	8/4/1987	9/5/2007	174	1.935	1.850	2,709,144	2,527,308	66,001
173993	WABASH RIVER AT VINCENNES US 50 AT MI PT 129.8	5120111	IN	38.70722	-87.5192	35,578.4	1/15/1980	12/27/1985	65	4.328	4.100	67,804,929	99,203,700	4,007,323
174000	WABASH R NR HUNTINGTON IND AT MILE POINT 409.05	5120101	IN	40.85479	-85.4983	1,993.2	1/10/1980	12/11/1985	59	5.526	5.250	4,292,240	10,252,786	666,878
174001	WHITE RIVER NEAR HAZELTON.	5120202	IN	38.49083	-87.5653	29,382.0	7/23/1986	7/23/1986	1	3.100	3.100			
174035	WHITE R AT PERKINSVILLE MILE POINT 278.610	5120201	IN	40.14167	-85.88	1,889.5	2/24/1977	8/19/1992	62	4.438	3.800	4,581,978	6,977,565	540,983
174303	BLUE R ON INTERSTATE 70 AT MILE POINT 63.76	5120204	IN	39.85401	-85.4773	242.3	2/8/1977	10/22/1985	25	5.088	4.500			
174317	WHITE RIVER AT EDWARDSPORT, MILE POINT 80.98	5120202	IN	38.80696	-87.2459	12,931.6	1/15/1980	12/27/1985	63	3.492	3.550	20,140,360	28,088,496	1,390,722
174319	WHITE RIVER AT SPENCER MILE POINT 162.22	5120202	IN	39.28778	-86.7458	7,751.8	2/10/1977	12/26/1985	68	4.889	4.600	14,625,722	20,294,749	768,536
174350	TIPPECANOE R-ROCHESTER US 31 N OF TOWN MI PT 107	5120106	IN	41.10603	-86.2204	1,485.7	7/22/1991	7/22/1991	1	3.700	3.700			
174554	WHITE R-W FORK-WAVERLY S.R. 144 BR MI PT 210	5120201	IN	39.55972	-86.2744	5,275.6	1/21/1986	11/30/1995	113	5.384	4.900	9,830,469	16,991,373	509,558
1BBFW0001	BEECH FORK LAKE	5090102	WV	38.29936	-82.3967	146.3	8/17/1973	8/2/1995	49	0.772	0.550	50,390	65,587	10,557
1DEWW0001	JOHNS CR OF LEVISA FORK KY	5070203	KY	37.73908	-82.7313	4,733.8	5/28/1974	7/30/1996	51	0.825	0.700	2,285,937	133,245	519,503
1DILW0003	LICKING CK AT TOBOSO GAGING STA	5040006	OH	40.05694	-82.22	1,743.9	8/29/1973	8/27/1996	42	3.937	3.600	3,083,053	2,387,022	373,327
1ELTW0031	East Fork Twelvepole Ck	5090102	WV	38.05222	-82.3081	127.9	6/15/1976	11/21/1997	68	0.373	0.250	17,731	43,729	5,017
1ELTW0032	Kiah Creek	5090102	WV	38.06098	-82.266	75.2	5/16/1977	11/21/1997	63	0.449	0.250	10,715	22,643	2,795
1FRLW0001	LEVISA FORK KY	5070202	KY	37.42494	-82.4121	1,019.3	6/25/1974	3/29/1994	72	0.655	0.600	299,957	287,132	28,872
1JWFW0001	POUND RIVER VA	5070202	VA	37.23734	-82.3433	574.7	6/26/1974	12/12/1995	74	0.878	0.700	174,947	153,495	25,954
1JWFW0002	POUND RIVER VA	5070202	VA	37.16594	-82.5233	215.4	6/26/1974	12/13/1995	72	1.237	0.625	176,273	63,074	45,952
1JWFW0003	CRANES NEST R OF POUND RIVER VA	5070202	VA	37.12397	-82.4385	179.7	6/25/1974	12/12/1995	72	1.028	0.800	141,564	47,373	38,244
1PCSW0002	Paint Creek, Main Inflow to Lake	5060003	OH	39.32166	-83.3866	706.7	7/26/1973	12/16/1996	60	5.399	4.500	1,939,414	2,563,202	204,138
1PESW0003	STILLWATER CREEK ABOVE POOL	5040001	OH	40.10192	-81.1461	130.0	6/14/1983	10/14/1996	30	0.844	0.800	35,674	73,738	5,251
1PESW0008	STILLWATER CREEK	5040001	OH	40.10259	-81.1311	130.0	5/14/1987	12/19/1997	46	0.380	0.238	15,314	71,401	4,880
1RDBW0012	Guyandot River	5070101	WV	37.61083	-81.7342	1,291.5	1/29/1980	12/29/1997	52	0.517	0.290	283,353	464,651	52,430
1RDBW0015	Clear Fork of Guyandot River	5070101	WV	37.60917	-81.7242	333.5	1/29/1980	12/29/1997	76	0.579	0.450	110,104	139,955	18,786
1SUMW0004	MUDDLETY CREEK OF GAULEY RIVER	5050005	WV	38.32675	-80.8326	128.0	10/11/1979	5/27/1998	33	0.609	0.600	50,309	66,663	7,804
1SUTW0008	RIGHT FK HOLLY RIVER WV	5050007	WV	38.63576	-80.4663	142.2	6/2/1976	5/27/1998	54	0.508	0.450	73,468	75,337	10,164
230028	GRAND RIVER AT WEBSTER RD BR; DELTA TWP SEC 3	4050004	MI	42.76111	-84.6496	3,251.8	4/3/1973	9/25/1993	319	3.425	3.200	3,071,756	2,807,405	59,119
233001	Sugar R. at Broadhead	7090004	WI	42.55023	-89.3663	1,551.8	12/8/1970	9/18/2007	148	4.990	4.900	2,562,636	2,092,869	86,206
243020	Fox R. at Berlin	4030201	WI	43.96667	-88.9502	3,426.7	1/20/1977	7/10/2007	201	1.807	1.780	1,897,705	1,923,075	72,752
250098	FLINT R AT CARPENTER RD BR; CITY OF FLINT	4080204	MI	43.07639	-83.6542	1,591.4	9/18/1973	9/23/1996	212	1.580	1.480	775,492	948,378	24,001
260001	Cuivre River	7110008	MO	38.92417	-90.7489	3,177.5	5/3/1993	11/8/2005	201	1.850	1.691	2,646,675	3,801,231	245,513
263001	MONTREAL RIVER NEAR SAXON, WI	4010302	WI	46.55658	-90.4141	689.3	4/16/1991	3/19/1998	10	0.926	0.850			
273038	BLACK RIVER AT BYPASS CHANNEL @ K	7040007	WI	44.38942	-90.7601	3,299.2	1/13/1994	6/5/2001	77	1.304	1.185	1,624,713	2,589,766	182,025
303066	Fox (IL) R. near New Munster	7120006	WI	42.61085	-88.2254	2,040.9	3/13/1997	7/30/2007	42	3.179	2.855	1,665,557	1,780,533	130,630
313038	KEWAUNEE RIVER NEAR KEWAUNEE, WI	4030102	WI	44.45745	-87.5557	343.4	6/28/1983	9/18/2007	120	3.926	3.650	380,809	536,957	27,031
320540	Iowa River HWY 92 BR AT COLUMBUS JUCTION	7080209	IA	41.27885	-91.3447	31,516.5	9/22/1976	8/2/2006	221	6.402	6.300	64,358,174	69,520,224	2,325,102
324015	CEDAR RIVER AT LEWIS ACCESS, 6.5 MI N OF PALO	7080205	IA	42.16135	-91.8107	15,903.7								
343033	Wolf R. at Langlade	4030202	WI	45.18998	-88.7337	1,204.9	7/10/2001	9/20/2007	75	0.578	0.594	232,897	232,852	15,362
353068	Wisconsin R. at Merril	7070002	WI	45.17822	-89.6862	7,122.0	5/25/1978	9/20/2007	78	0.783	0.729	1,516,534	1,083,728	55,940
373001	WISCONSIN RIVER AT WAUSAU	7070002	WI	44.95773	-89.6358	7,829.1	11/19/1970	6/19/2001	262	0.944	0.890	2,070,641	1,542,910	82,175
373325	BIG EAU PLEINE RIVER AT STRATFORD, WI	7070002	WI	44.82076	-90.0788	632.0	2/2/1976	9/12/2007	85	1.571	1.330	490,061	738,950	58,312
380031x	GRAND R AT MAPLE GROVE RD; RIVES TWP, SEC 35	4050004	MI	42.34167	-84.4016	932.4	8/30/1971	8/8/2001	126	2.860	2.620	734,481	547,050	140,369
380153	SHEYENNE RIVER - BALHILL DAM	9020204	ND	47.02735	-98.0862	9,847.5	8/22/1995	11/7/2006	29	1.341	1.180	247,285	942,781	20,873
3822350602001	HOCKING R. DST LANCASTER - 1ST U.S. RT 33 BRIDGE	5030204	OH	39.68595	-82.5738	179.7	1/23/1976	1/8/1997	252	4.007	3.810	235,188	314,126	7,945
383001	PESHTIGO RIVER AT PESHTIGO, WI	4030105	WI	45.05381	-87.7471	2,794.6	12/9/1970	8/8/2007	293	0.827	0.750	614,286	849,885	23,572
390057	KALAMAZOO R AT "G" AVE BRIDGE; CITY OF AUGUSTA	4050003	MI	42.33528	-85.3453	2,356.1	8/30/1971	9/21/1993	191	1.832	1.830	1,366,131	2,327,871	22,509
390553	South Skunk River CO RD BR E57	7080105	IA	41.97322	-93.5806	1,480.0	11/8/1979	11/9/1998	79	10.880	10.365	5,052,821	3,425,640	710,419
390566	SOUTH SKUNK RIVER APPROX 3.5 MI NNW OF CAMBRIDGE	7080105	IA	41.95035	-93.5511	1,523.8	10/8/1991	9/12/2007	184	11.327	11.250	5,272,574	3,482,294	295,997
391732085414401	CLIFTY CREEK AT CO RD 1150 E NEAR HARTSVILLE, IN	5120206	IN	39.29227	-85.6955	227.7	3/26/1993	8/10/2004	33	6.025	6.300	876,056	934,705	381,065
394340085524601	SUGAR CREEK AT CO RD 400 S AT NEW PALESTINE, IN	5120204	IN	39.72782	-85.8794	239.8	5/7/1992	8/30/2005	271	2.859	2.404	510,462	1,180,824	36,961
40007	Cannon River	7040002	MN	44.58477	-92.5628	3,835.2	10/1/1991	11/8/2005	249	4.886	4.748	5,301,861	6,202,996	206,843
410050	GRAND RIVER AT KNAPP STREET; ADA TWP, SEC 7	4050006	MI	43.00611	-85.5425	11,577.7	8/30/1971	9/22/1993	204	2.236	2.030	9,021,655	12,462,977	220,432
410052	GRAND R AT M-11 BRIDGE; CITY OF GRANDVILLE	4050006	MI	42.91524	-85.7667	12,790.2	8/30/1971	9/22/1993	240	2.465	2.260	9,964,717	14,403,151	187,894
413002	MILWAUKEE RIVER @ BROWN DEER ROAD	4040003	WI	43.17769	-87.9561	1,632.4	12/16/1970	11/17/1976	41	2.053	1.910	910,911	1,869,721	64,684
423014	North Raccoon River CO RD BR D46 SEC 13/24 AT SAC	7100006	IA	42.33971	-94.9859	1,834.5	10/9/1979	9/10/2007	350	11.751	11.200	6,882,868	5,098,036	263,435

426046	South River BRIDGE HIWAY 92	7100008	IA	41.37214	-93.4309	1,230.8	11/6/1986	9/6/2007	93	1.776	1.300	1,121,505	1,148,392	186,981
430380	Des Moines River CO RD E26 NW OF BOONE	7100004	IA	42.08029	-93.9382	14,065.6	1/5/1971	10/27/1981	195	6.473	6.260	49,718,547	28,707,560	4,862,869
433002	OCONTO RIVER NEAR OCONTO, WI	4030104	WI	44.88271	-87.8843	2,504.5	10/26/1988	7/11/2007	47	1.197	0.933	566,377	1,130,840	36,260
443001	WISCONSIN RIVER AT RHINELANDER	7070001	WI	45.64094	-89.4205	2,221.8	4/17/1974	12/11/2001	115	0.603	0.610	341,760	193,818	13,731
443002	WISCONSIN R AT HAT RAPIDS DAM	7070001	WI	45.5742	-89.4789	2,929.2	4/17/1974	6/30/1999	212	0.896	0.855	464,508	348,080	20,678
443003	WISCONSIN RIVER BRIDGE AT MCNAUGHTON	7070001	WI	45.73376	-89.5133	2,015.8	3/27/1979	11/29/2001	173	0.588	0.550	290,558	182,313	12,809
443015	East Fk Des Moines River HWY 169 BR 1 MI N ST JOSE	7100003	IA	42.93537	-94.2271	2,439.4	1/19/1971	12/7/1998	151	8.038	7.900	8,414,842	5,028,205	687,647
443704	Wapsipinicon River CO RD BR 0.5 MI W. OF D16 N. OF	7080102	IA	42.51197	-91.9746	2,386.1	10/30/1979	9/10/2007	142	6.441	5.900	7,534,303	6,708,912	429,053
444066	Des Moines River CO RD BR P59 AT KALO S. OF FT DOD	7100004	IA	42.43159	-94.1325	10,842.0	11/16/1979	9/10/2007	113	7.988	7.990	33,278,353	20,714,904	2,092,063
46021	DUPAGE R OLD RT 6 S CHANNAHON T34N R9E NW20	7120004	IL	41.41944	-88.2278	954.2	3/23/1983	2/10/1984	4	7.605	7.960			
47018	W BR DUPAGE R 2 MI S NAPERVILLE T37N R10E NE6	7120004	IL	41.72	-88.1322	319.9	3/25/1983	2/6/1984	4	5.465	4.430			
47041	HICKORY CR 1 MI NE NEW LENOX T35N R11E NE14	7120004	IL	41.51611	-87.973	198.8	9/8/1983	4/5/1984	3	5.033	4.500			
47087	Kaskaskia River RT 133 BRIDGE IN CHESTERVILLE.	7140201	IL	39.70266	-88.3871	931.1	4/27/1983	12/16/1997	5	7.516	8.370			
48046	FOX R AT WEDRON T34N R4E NE9	7120007	IL	41.4319	-88.7726	6,602.5	5/17/1982	8/30/1996	5	3.808	3.220			
48246	SALT CR YORK RD HINSDALE T39N R11E SE36	7120004	IL	41.82083	-87.9271	306.0	4/13/1995	10/2/1995	8	6.205	4.300			
48247	SALT CR RT 51 ELMHURST T39N R11E SW14	7120004	IL	41.86592	-87.9517	253.7	4/12/1995	10/2/1995	8	6.128	4.055			
48278	S BR KISHWAUKEE R 1.5 MI W SYCAMORE T41NR4ESE25	7090006	IL	41.99662	-88.7222	240.5	8/12/1983	7/16/1984	4	9.648	9.065			
483026	MISS R.LOCK+DAM # 2 HASTINGS,MN.	7010206	MN	44.76058	-92.8674	90,233.2	9/16/1971	9/15/1994	288	3.890	3.400	68,389,635	84,207,592	1,976,074
483027	Mississippi R. Above LD 3	7040001	WI	44.61125	-92.611	109,835.0	10/6/1970	12/1/1998	342	3.092	2.725	74,371,335	90,585,780	3,399,701
48343	KICKAPOO CR 1 MI SSW CHARLESTON T12N R9E SW22	5120112	IL	39.46311	-88.1934	253.8								
48414	KICKAPOO CR RT 136 BR 1 M W HEYWORTH T22NR2ESW32	7130009	IL	40.31209	-89.0144	306.9	9/16/1981	9/24/1997	4	5.610	6.245			
48494	HURRICANE CR 4 MI WNW HERRIN T8SR1ESE16	7140106	IL	37.82277	-89.095	62.0	7/25/1995	2/29/1996	2	1.445	1.445			
48525	PECATONICA R E EDGE WINSLOW T29N R6E SE22	7090003	IL	42.49312	-89.7897	2,676.1	5/1/1984	11/18/1998	6	6.250	6.250			
4CRR11005	CHEAT RIVER RM 75.6	5020004	WV	39.12125	-79.6756	1,850.2	4/26/1973	3/12/1996	109	0.571	0.530	1,192,563	1,155,065	72,682
500233	CLINTON R@ NO. BOUND GRATIOT AVE IN MT. CLEMENS	4090003	MI	42.58417	-82.8828	1,951.2	1/17/1974	11/22/2005	362	3.250	3.130	1,057,180	1,576,475	30,875
501790	ROCKY R. AT LAKEWOOD - PARK BLVD. (RM 3.0)	4110001	OH	41.46944	-81.8317	746.3	11/19/1980	12/9/1998	228	4.691	4.260	946,884	1,006,616	36,347
501810	ROCKY R. AT FAIRVIEW PARK - PURITAS RD (RM 8.18)	4110001	OH	41.43778	-81.8456	728.6	5/22/1974	9/3/1997	28	4.323	3.880	700,959	1,000,712	84,169
502070	CUYAHOGA R AT SHALERSVILLE - DIAGONAL RD-SR 303	4110002	OH	41.23806	-81.3031	488.7	8/8/1973	12/10/1998	70	1.000	0.950	207,335	347,905	17,298
502080	L. CUYAHOGA R. AT AKRON - OTTO ST GAGE (RM 1.85)	4110002	OH	41.09444	-81.5217	160.5	8/8/1973	9/4/1996	65	2.022	1.620	107,296	43,040	13,012
502130	CUYAHOGA R. AT CLEVELAND - LOWER HARVARD AVE.	4110002	OH	41.44778	-81.685	2,032.8	10/23/1991	12/10/1998	81	5.560	4.970	5,390,208	4,449,083	209,591
503003	WISCONSIN RIVER ABOVE DUBAY DAM	7070002	WI	44.6649	-89.6505	12,483.3	8/17/1976	4/5/2001	193	1.329	1.265	4,667,486	5,090,275	244,080
503059	WISCONSIN RIVER ABOVE STEVENS POINT DAM	7070003	WI	44.51641	-89.5864	12,686.8	8/18/1976	6/19/2001	94	1.274	1.217	4,606,342	5,061,014	288,542
510017	MANISTEE R. AT HIGH BR RD; DICKSON TWP, SEC 33	4060103	MI	44.2675	-86.0139	3,621.4	4/8/1975	10/12/1982	104	0.505	0.480	758,157	1,043,704	22,868
523061	Root River	4040002	WI	42.78528	-87.8299	478.9	1/26/1977	9/18/2007	259	4.297	3.930	605,794	633,615	38,630
53210	Fox R. at DePere	4030204	WI	44.46111	-88.0591	16,171.5	10/17/1988	1/11/2005	143	1.670	1.596	7,452,501	5,765,558	298,009
543056	YAHARA R AT CTH M AT FULTON	7090001	WI	42.80806	-89.1255	1,231.4	7/18/1977	10/26/1997	72	6.214	5.465	1,503,207	999,746	54,071
550353	Tug Fork at Fort Gay, W.Va.	5070201	WV	38.11694	-82.5983	4,030.6	3/5/1985	8/19/2004	126	0.873	0.760	3,159,218	1,272,446	433,835
550446	Monongahela River below Morgantown, W. Va.	5020003	WV	39.65806	-79.9931	6,920.1	7/16/1974	9/15/2004	254	1.031	0.900	5,078,786	4,041,592	334,488
550476	Coal River at Tornado, W. Va.	5050009	WV	38.33889	-81.8408	2,223.3	11/13/1973	8/16/2004	246	0.856	0.760	1,375,009	835,200	118,262
550500	New River at C&O Railroad Bridge	5050004	WV	38.15092	-81.1799	17,993.3	11/19/1973	8/17/2004	247	0.979	0.900	8,208,787	10,849,961	582,723
550544	Elk River at Coonskin Park, above Charleston, WV	5050007	WV	38.38531	-81.586	3,917.8	11/13/1973	7/8/2004	205	0.689	0.630	1,844,456	1,919,168	190,935
573052	Wisconsin R. at Wisconsin Del	7070003	WI	43.62734	-89.7808	20,668.3	2/28/1977	9/17/2007	243	1.345	1.296	8,085,332	18,381,401	209,965
573081	BARABOO RIVER 1994 MILWAUKEE BRIDGE	7070004	WI	43.47032	-89.7689	1,433.7	9/23/1997	12/3/2001	53	1.906	1.905	809,568	1,464,340	38,393
573082	BARABOO RIVER AT HWY 113	7070004	WI	43.45833	-89.7165	1,455.5	9/23/1997	12/3/2001	53	1.904	1.911	825,258	1,514,486	35,993
580046	RAISIN R. AT ERA DOCK NEAR MOUTH; CITY OF MONROE	4100002	MI	41.90056	-83.3544	2,743.0	10/12/1970	10/3/2005	283	3.299	2.630	2,387,707	5,436,475	173,174
600150	GREAT MIAMI R. AT MIAMISBURG - S.R. 725	5080002	OH	39.64444	-84.2897	7,015.9	11/21/1994	12/17/1998	28	4.894	4.730	18,733,215	16,352,764	1,327,129
600810	SCIOTO R. AT SHADEVILLE - S.R. 665	5060001	OH	39.8325	-83.0083	4,387.8	1/22/1976	10/7/1996	477	7.242	7.220	9,706,478	15,237,444	270,245
600850	SCIOTO R. AT COLUMBUS - MOUND ST.	5060001	OH	39.95222	-83.0125	4,183.1	6/18/1980	10/7/1996	460	3.988	3.580	11,049,586	13,539,642	501,342
600870	SCIOTO R. AT COLUMBUS - FRANK RD.	5060001	OH	39.91667	-83.0097	4,205.8	9/3/1980	10/7/1996	501	4.321	3.755	10,327,433	13,513,285	402,213
600880	SCIOTO R. AT COLUMBUS - I-270 S.	5060001	OH	39.88062	-83.0179	4,232.5	6/17/1980	10/7/1996	484	7.319	7.325	8,707,759	15,175,126	217,635
600900	SCIOTO R. NR COMMERCIAL POINT - S.R. 762	5060001	OH	39.77417	-83.0075	5,879.7	10/18/1979	10/7/1996	509	6.457	6.365	11,440,366	18,595,067	233,406
600910	SCIOTO R. NR SOUTH BLOOMFIELD - S.R. 316	5060001	OH	39.71944	-83.0125	5,987.4	6/17/1980	10/7/1996	439	6.306	6.120	12,060,063	18,711,077	270,903
600920	SCIOTO R. DST CHILLICOTHE - U.S. RT. 35	5060002	OH	39.30556	-82.9203	10,010.7	8/15/1979	9/23/1997	17	3.219	3.210			
600960	SCIOTO R. AT CIRCLEVILLE - U.S. RT. 22	5060002	OH	39.60139	-82.9553	8,331.7	1/22/1976	9/16/1996	73	4.716	4.850	14,353,106	24,681,683	1,182,014
601000	SCIOTO R. AT PIKETON - U.S. RT. 23 (RM 34.50)	5060002	OH	39.07222	-83.0056	15,114.5	8/1/1979	9/23/1997	6	2.863	2.860			
601010	SCIOTO R. AT JASPER - S.R. 124 (RM 30.08)	5060002	OH	39.04716	-83.05	15,135.1	8/1/1979	9/23/1997	6	2.980	2.920			
601290	OLENTANGY R. NR WORTHINGTON - I-270 N.	5060001	OH	40.11091	-83.0329	1,284.0	2/4/1976	1/23/1997	114	3.493	3.185	2,332,780	3,292,658	174,689
601340	SCIOTO R. UPST CIRCLEVILLE - FLORENCE CHAPEL RD.	5060001	OH	39.63278	-82.9625	6,830.3	10/25/1977	1/8/1997	246	5.189	5.120	11,509,977	20,148,935	346,187
601350	MILL CREEK OFF WALDO ROAD - MARYSVILLE (RM 16.8)	5060001	OH	40.25583	-83.3453	235.4	5/17/1977	1/23/1997	228	6.534	5.270	378,559	872,088	23,128
601400	RACCOON CREEK AT ADAMSVILLE - U.S. RT. 35	5090101	OH	38.87361	-82.3561	1,518.7	10/25/1977	12/14/1998	121	0.865	0.720	474,519	738,678	34,815

601930	TUSCARAWAS R. AT MASSILLON - WARMINGTON ST.	5040001	OH	40.75222	-81.5292	1,343.7	1/29/1976	12/22/1998	266	5.559	4.800	2,014,277	1,512,635	65,904
601940	NIMISHILLEN CRK DST N. INDUSTRY - HOWENSTINE RD.	5040001	OH	40.71718	-81.3473	463.2	2/25/1976	12/22/1998	263	6.735	6.330	1,272,103	829,000	28,092
602280	MAHONING R. AT LEAVITTSBURG - LEAVITT RD.	5030103	OH	41.23944	-80.8811	1,487.5	10/16/1973	12/22/1998	297	1.294	1.200	826,047	1,173,554	50,313
603095	Sheboygan R. at Esslingen Par	4030101	WI	43.74053	-87.7512	1,103.7	2/23/1977	9/10/2007	321	2.785	2.510	941,136	1,797,659	39,630
610060	GREAT MIAMI R. AT DAYTON - MONUMENT AVE.	5080002	OH	39.76334	-84.2021	6,491.1	10/12/1978	12/17/1998	220	4.456	4.070	15,481,231	14,605,178	560,082
610520	L. MIAMI R. NR MORROW - STUBBS MILL RD.	5090202	OH	39.3634	-84.1735	2,498.5	4/7/1976	9/1/1998	71	3.723	3.525	4,928,478	5,192,944	339,765
610530	E FK L MIAMI R NR MILFORD - NR TERRACE PARK C.C.	5090202	OH	39.15526	-84.2898	1,292.6	2/28/1978	9/8/1998	62	2.450	2.390	1,228,901	1,797,086	68,886
611740	MUSKINGUM R. DST COSHOCTON - S.R. 83	5040004	OH	40.23547	-81.8713	12,562.8	1/14/1976	12/16/1998	186	2.452	2.420	12,749,932	13,096,522	360,159
611790	TUSCARAWAS R. AT NEWCOMERSTOWN - RIVER ST.	5040001	OH	40.26139	-81.6092	6,322.3	10/28/1977	12/16/1998	208	2.530	2.400	6,161,408	6,091,545	162,840
63029	Mississippi R. Above LD 4	7040003	WI	44.32495	-91.9227	141,590.8	1/24/1977	12/1/1998	264	1.956	1.880	58,590,582	106,389,496	1,190,091
643509	Wis R Tailwater - Otter RPS Dam	7070001	WI	45.90361	-89.3214	1,427.1	8/10/1999	11/29/2001	26	0.645	0.564			
683096	Fox (IL) R. below Waukesha	7120006	WI	42.93375	-88.2928	475.2	1/26/1977	1/30/2007	220	4.419	4.330	606,878	539,428	16,037
693035	Wolf R. at New London	4030202	WI	44.3917	-88.7496	6,123.2	1/19/1977	9/5/2007	286	1.547	1.370	2,847,773	3,428,775	104,356
6ACNR009.17	Cranes Nest River N.E. FROM ROUTE 83	5070202	VA	37.15083	-82.4108	198.6	4/13/1982	11/2/1998	124	0.825	0.785	77,297	50,042	3,916
6ADIS001.24	Dismal Creek, RT. 666 BRIDGE	5070202	VA	37.24278	-82.0275	223.0	7/27/1976	12/14/1998	177	0.423	0.385	55,244	69,393	4,222
6AKOX008.11	Knox Creek RT. 697 AT STATE LINE	5070201	KY	37.47064	-82.0624	228.9	7/18/1974	12/14/1998	193	0.590	0.565	82,733	62,168	11,939
6ALEV131.52	Levisa Fork WELLMORE COAL CO.DOCK #14 BR OFF 460 .	5070202	VA	37.34833	-82.2056	770.1	6/17/1992	12/14/1998	74	0.478	0.465	271,336	226,853	31,531
6ALEV143.86	Levisa Fork TEEL BR.ON RAILROAD AVE OFF RT 83, .1	5070202	VA	37.27694	-82.1008	505.9	6/17/1992	12/14/1998	73	0.496	0.490	157,035	145,071	18,639
6AMCR007.46	McClure River RT. 63 BR N OF CLINCH	5070202	VA	37.16831	-82.361	251.0	5/4/1973	12/7/1998	213	0.555	0.455	54,096	70,302	6,294
6ARSS026.98	Russell Fork UPSTREAM OF LAZARUS BR & DOWNSTR RT 7	5070202	VA	37.1925	-82.2878	389.9	5/14/1992	12/7/1998	42	0.456	0.383	142,054	111,785	23,557
6ASAT000.03	Slate Creek RT. 460 BRIDGE IN GRUNDY	5070202	VA	37.27889	-82.0994	106.2	7/27/1976	9/11/1986	102	0.637	0.585	58,678	30,242	9,096
70005	BLACK RIVER NEAR GALESVILLE, WI	7040007	WI	43.96192	-91.3371	5,868.2	5/6/1993	11/9/2005	240	1.463	1.340	2,673,797	4,009,662	102,886
713001	FOX RIVER AT OMRO	4030201	WI	44.04014	-88.7446	3,829.4	12/15/1970	1/9/1996	65	1.746	1.660	2,038,287	2,422,316	92,338
713002	Fox R. at Neenah and Menasha	4030203	WI	44.18647	-88.4565	15,567.1	7/17/1986	9/5/2007	210	1.310	1.306	5,328,966	3,911,163	134,440
723002	Wisconsin R. at Biron	7070003	WI	44.43405	-89.7764	13,944.2	8/17/1976	9/19/2007	272	1.460	1.420	5,508,019	5,917,414	119,845
723020	WISCONSIN RIVER AT STH 73 IN NEKOOSA	7070003	WI	44.31438	-89.8884	14,230.6	2/8/1978	4/5/2001	98	1.655	1.580	5,755,872	6,686,464	314,279
730023	SHIAWASSEE R@ FERGUS RD; ST. CHARLES TWP SEC 22	4080203	MI	43.25472	-84.1056	1,576.2	8/31/1971	11/29/2005	93	1.720	1.411	1,008,888	1,273,111	75,891
730150	SAGINAW R OFF CENTER ST BR; SAGINAW TWP, SEC 35	4080206	MI	43.40222	-83.9661	15,322.4	1/29/1974	6/18/1996	96	2.789	2.580	10,444,976	14,123,032	578,891
740144	BLACK R AT 10TH AVE. BRIDGE; CITY OF PORT HURON	4090001	MI	42.98083	-82.4336	1,836.3	5/21/1973	6/18/1978	56	1.768	1.255	2,214,303	2,101,154	259,781
740153	BLACK R AT WADHAMS RD BRIDGE; KIMBALL TWP, SEC 2	4090001	MI	42.98981	-82.5378	1,759.8	5/22/1973	8/24/1993	214	2.892	2.210	2,061,062	2,085,291	147,046
784016	ENGLISH RIVER AT BRIDGE CO RD W61 AT RIVERSIDE	7080209	IA	41.47548	-91.5802	1,626.8	10/20/1986	9/11/2007	255	6.010	5.710	4,188,503	3,204,683	283,850
80001	LaCrosse River	7040006	WI	43.81882	-91.2535	1,234.4	10/4/1999	9/18/2007	125	1.938	1.852	584,872	881,207	11,262
810006	SALINE R AT MAPLE RD BRIDGE; SALINE TWP, SEC 13	4100002	MI	42.13	-83.7756	247.6	9/1/1971	10/8/1980	40	3.120	2.920	252,681	304,912	17,628
810042	HURON R AT SUPERIOR RD BR; SUPERIOR TWP, SEC 32	4090005	MI	42.25954	-83.6394	2,060.6	9/1/1971	8/25/1993	242	2.119	2.000	940,626	1,323,641	17,228
810242	HURON R AT HURON BR PARK; ANN ARBOR TWP., SEC 17	4090005	MI	42.30333	-83.7467	1,892.3	4/16/1975	9/14/2005	214	1.085	0.990	622,542	903,466	31,273
821004	CEDAR CREEK AT BRIDGE 3 MI WNW OF OAKLAND MILLS	7080107	IA	40.94456	-91.6783	1,383.1	10/20/1986	12/1/1998	147	5.489	5.000	3,522,281	2,469,064	396,916
9-CRK003.00	Crooked Creek, RT. 635 BRIDGE AT INTERSECTION WITH	5050001	VA	36.76861	-80.9078	185.7	6/18/1992	11/30/1998	38	0.616	0.578	74,415	112,633	5,049
9-EKC003.78	AT MT CARMEL CHURCH ON RT 650, 1.8 MI OFF RT 660	5050001	VA	36.69806	-81.0575	164.4	5/21/1992	12/17/1998	40	1.017	0.975	108,825	97,970	8,159
9-FXC000.84	LOW WATER BRIDGE OFF RT 711,APPROX .75 MI W. FOX	5050001	VA	36.61444	-81.3056	197.9	5/21/1992	11/30/1998	38	0.466	0.465	66,670	95,084	9,742
9-LRV000.34	Little River RT. 605 BRIDGE, S OF RADFORD	5050001	VA	37.07861	-80.5753	910.7	11/2/1970	11/4/1998	95	0.700	0.647	426,779	525,965	105,024
9-NEW081.72	New RIver RT. 11 BRIDGE AT RADFORD	5050001	VA	37.13861	-80.575	7,151.9	11/2/1970	12/8/1998	304	0.937	0.880	4,758,762	3,835,299	239,353
9-NEW107.51	New River STA #25 ALLSONIA AT GAGE (PULASKI COUNTY	5050001	VA	36.93639	-80.7461	5,726.1	11/29/1993	12/7/1998	57	0.786	0.785	2,463,362	3,218,602	139,605
9-NEW187.46	New River OFF RT. 58 AT MOUTH OF WILSON OR RT. 93	5050001	VA	36.58531	-81.3142	1,744.4	11/3/1970	12/17/1998	291	0.776	0.755	813,207	945,001	40,996
9-WFC016.45	Wolf Creek at PRIVATELY OWNED LOW WATER BR OFF RT	5050002	VA	37.25641	-81.0143	505.6	6/15/1992	11/3/1998	32	0.661	0.655	166,781	192,474	13,017
9-WLK004.34	Walker Creek RT. 622 BRIDGE, BELOW PEARISBURG	5050002	VA	37.29651	-80.7063	791.9	11/2/1970	11/17/1998	90	0.625	0.567	170,354	277,774	35,695
9-WLS002.57	Wilscon Creek, RT 721 BRIDGE OFF RT 56/16, APPROX	5050001	VA	36.59972	-81.3547	75.6	6/10/1992	11/30/1998	38	0.564	0.555	26,448	46,709	1,695
90003	Upper Iowa River	7060002	IA	43.47444	-91.2944	2,568.5	10/26/1979	9/20/2004	397	4.767	4.532	5,301,980	4,116,261	207,534
93001	Chippewa R. at Chippewa Falls	7050005	WI	44.93001	-91.397	14,346.7	10/6/1970	1/11/2005	137	0.905	0.868	3,794,112	4,254,691	114,346
AR01	Alllegheny River at Aspinwall, PA (rmi -7.4)	5010009	PA	40.48684	-79.9049	30,097.8	7/18/2002	7/5/2006	24	1.325	1.239			
ATH 02	S Fork Saline R.	5140204	IL	37.67973	-88.8037	212.7	6/9/1993	12/16/1993	4	1.475	0.850			
CAS0479	CASSELMAN R. CROS. BY RIVER RD. AT USGS STA.	5020006	MD	39.70389	-79.14	165.2	1/7/1985	8/4/1998	92	0.900	0.870	104,664	113,417	4,828
GMW020-0001	Greens Fk	5080003	IN	39.77167	-85.1092	223.4	3/19/1997	9/23/2003	60	3.737	3.400	492,070	809,110	41,468
GMW040-0005	Whitewater River	5080003	IN	39.49806	-85.1825	1,497.0	3/18/1997	3/1/2006	88	3.558	3.400	3,243,563	3,915,590	298,831
GMW080-0001	Whitewater River	5080003	IN	39.35333	-84.9428	3,418.3	7/25/1996	3/1/2006	99	2.713	2.616	5,186,027	6,976,412	314,333
K3250000	S FORK NEW RIV AT NC 16 AND 88 NR JEFFERSON	5050001	NC	36.39473	-81.4075	533.6	4/23/1981	8/22/2007	149	0.812	0.800	365,716	323,839	13,836
K4500000	S FORK NEW RIV AT NC 221 NR SCOTTVILLE	5050001	NC	36.4738	-81.3365	778.2	10/2/1974	8/22/2007	171	0.793	0.770	560,656	487,202	24,317
K7900000	NEW RIV AT SR 1345 AT AMELIA	5050001	NC	36.5519	-81.1817	2,141.8	3/15/1976	9/26/2007	236	0.816	0.780	1,196,112	1,160,056	49,273
LEJ050-0006	Fish Cr	4100003	IN	41.55861	-84.8356	105.7	4/6/1999	2/6/2006	70	2.017	1.800	155,359	163,799	19,136
LEJ050-0007	Fish Cr	4100003	IN	41.465	-84.8142	174.9	4/6/1999	2/6/2006	81	1.984	1.607	204,208	267,134	20,189
LEJ060-0006	ST Joseph River	4100003	IN	41.3475	-84.8439	1,571.2	4/6/1999	2/6/2006	76	2.526	1.900	2,278,720	3,168,928	201,541

LEJ090-0008	Cedar Cr	4100003	IN	41.21889	-85.0767	785.3	4/6/1999	7/9/2001	28	3.504	2.500	2,502,687	1,797,858	461,735
LEJ090-0026	Cedar Cr	4100003	IN	41.215	-85.0514	787.7	8/27/2001	1/23/2006	49	3.142	2.500	1,524,502	1,805,711	177,454
LEJ100-0003	ST Joseph River	4100003	IN	41.08917	-85.1292	2,808.5	11/15/1983	1/23/2006	237	3.121	2.650	3,652,656	5,962,107	237,081
LEM010-0012	Maumee River	4100005	IN	41.08194	-85.1147	4,855.0	2/22/1977	1/23/2006	107	3.911	3.400	11,068,160	17,847,825	1,084,898
LEM010-0013	Maumee River	4100005	IN	41.16906	-84.8493	5,223.8	4/7/1971	1/23/2006	299	4.771	4.100	10,968,103	19,938,835	520,726
LEM010-0014	Maumee River	4100005	IN	41.08444	-85.0206	4,962.3	2/22/1977	1/23/2006	304	4.956	4.500	10,297,824	19,273,125	488,628
LES040-0007	St. Mary's River	4100004	IN	40.77917	-84.8422	1,305.6	1/9/1980	2/7/2006	161	5.644	5.000	6,100,027	8,709,466	512,783
LES060-0004	ST Mary's River	4100004	IN	41.08389	-85.1358	2,042.1	1/7/1986	1/23/2006	219	5.535	4.900	7,189,170	11,883,735	556,088
LES060-0005	ST Mary's River	4100004	IN	40.99111	-85.1169	1,847.3	4/6/1999	1/23/2006	70	5.412	4.800	7,518,948	11,589,412	832,341
LMG050-0006	Salt Cr	4040001	IN	41.59972	-87.1464	192.5	6/29/1999	1/30/2006	72	3.161	3.000	228,521	269,890	12,326
LMG050-0007	Salt Cr	4040001	IN	41.49861	-87.1417	92.1	9/9/1976	9/4/2003	107	3.686	3.500	130,334	164,361	8,148
LMG060-0008	E Br Little Calumet River	4040001	IN	41.61694	-87.1261	179.7	10/6/1976	1/30/2006	137	1.896	1.700	169,105	194,201	9,417
LMJ120-0009	Pigeon River	4050001	IN	41.74	-85.5569	875.4	4/15/1999	2/8/2006	82	2.241	2.100	904,596	1,284,123	36,575
LMJ150-0004	ST Joseph River	4050001	IN	41.72278	-85.8147	6,387.9	1/5/1977	2/8/2006	291	2.626	2.400	6,258,606	7,300,182	159,624
LMJ190-0006	Elkhart River	4050001	IN	41.50778	-85.7594	867.9	4/14/1999	2/8/2006	79	2.545	2.300	960,007	1,558,999	41,813
LMJ210-0008	Elkhart River	4050001	IN	41.68778	-85.9722	1,792.2	1/5/1977	2/8/2006	259	3.053	2.500	2,270,760	3,084,407	75,752
LMJ240-0008	ST Joseph River	4050001	IN	41.74444	-86.2728	9,395.8	1/5/1977	12/19/2002	255	3.032	2.700	10,926,583	12,277,355	436,153
LMJ240-0009	ST Joseph River	4050001	IN	41.67111	-86.1536	9,151.7	9/21/1989	5/9/2002	36	2.399	2.300	8,184,896	11,879,627	270,058
LMJ240-0026	ST Joseph River	4050001	IN	41.66639	-86.1678	9,155.3	6/19/2002	2/8/2006	44	2.457	2.250	9,754,804	11,880,025	482,316
LRBF-.5-BB14A71	BIG FORK RIVER BRIDGE ON MN-11, 4 MI E OF LOMAN	9030006	MN	48.5125	-93.71	3,884.3	9/22/1971	9/9/1992	71	1.230	1.120	997,109	737,181	50,052
LRLF7.5-BB14A71	LITTLE FORK R MN-11 BRIDGE, 0.5 MI W OF PELLAND	9030005	MN	48.52132	-93.5866	4,718.2	9/22/1971	9/9/1992	65	1.270	1.180	1,585,562	796,609	97,805
LSSL-52-BC15E53	ST. LOUIS R BRIDGE ON US-2, 2 MI SE OF BROOKSTON	4010201	MN	46.84944	-92.5758	8,425.6	10/19/1970	9/13/1994	217	1.184	0.995	2,483,704	1,270,233	106,646
LYO0004	LIT. YOUGH. R. OLD FOOT BR. 0.4M. AB. MOUTH	5020006	MD	39.41831	-79.4192	105.7	5/7/1981	12/5/1995	87	1.676	1.695	114,086	97,841	7,397
M03P19	E FK TODD FORK AT CLARKSVILLE - SR 133/132	5090202	OH	39.39861	-83.9831	98.8	12/10/1981	9/10/1998	55	3.938	3.660	268,896	299,888	49,500
MNCO-.5-B-14A67	COTTONWOOD R AT MN-15, 0.5 MI SE OF NEW ULM	7020008	MN	44.28237	-94.4356	3,372.6	10/6/1970	9/26/1994	183	4.816	3.680	9,989,724	5,276,080	876,432
MNMN-91-BB15E71	MINNESOTA RIVER AT BRIDGE ON MN-22 AT ST. PETER	7020007	MN	44.30742	-93.9596	36,896.9	9/13/1971	9/26/1994	214	6.208	5.620	65,168,696	50,489,494	3,748,247
MR01	Monongahela River near South Pittsburgh, PA	5020005	PA	40.41026	-79.953	19,070.8	7/6/1976	9/7/2004	264	1.540	1.420	11,876,182	11,920,373	769,775
MS216	PRAIRIE CR AT 310TH ST .2 MI UPSTM OF L BYLLESBY	7040002	MN	44.50056	-92.9925	196.5	9/7/1989	9/15/1994	47	6.032	5.730	355,208	381,431	27,022
MSCW-.2-B-14A53	CROW RIVER AT BRIDGE ON CSAH-36 AT DAYTON	7010203	MN	45.24471	-93.522	6,685.9	11/3/1970	6/25/1998	121	3.244	2.945	5,869,744	8,779,526	559,050
MSRM-.6-BB14A53	RUM RIVER AT BRIDGE ON PLEASANT STREET IN ANOKA	7010207	MN	45.2066	-93.3867	4,055.4	10/5/1970	9/7/1994	90	1.374	1.300	1,049,025	1,630,066	52,603
MSRM-34-BB14A55	RUM RIVER BRIDGE ON CSAH-5, 0.5 MI W OF ISANTI	7010207	MN	45.49333	-93.2669	3,314.8	6/7/1974	9/7/1994	198	1.367	1.300	846,490	1,275,048	26,529
MSU--68CBB15E53	MISSISSIPPI R UPSTREM OF MN-15 BR AT SAUK RAPIDS	7010201	MN	45.59813	-94.1879	30,255.0	9/21/1988	9/7/1994	54	1.072	1.020	5,400,424	6,804,474	223,592
MSU-859---01E53	MISSISSIPPI R MPLS WATERWORKS INTAKE AT FRIDLEY	7010206	MN	45.04917	-93.2792	47,566.3	10/5/1970	9/7/1994	218	1.481	1.410	13,563,552	21,347,034	294,006
MSU-982CBB15E67	MISSISSIPPI RIVER AT BR ON MN-115 AT CAMP RIPLEY	7010104	MN	46.07472	-94.3344	26,472.7	11/4/1970	9/29/1994	206	0.995	0.940	5,110,469	4,326,499	140,796
MSU1186-BB15E67	MISSISSIPPI R AT MN-6 BRIDGE 8 MI SW OF COHASSET	7010101	MN	47.22955	-93.7572	7,549.5	10/19/1970	9/27/1994	195	0.855	0.815	1,003,338	246,279	32,665
MSU1292-BB15E67	MISSISSIPPI R AT BR ON CSAH-8 7 MI E OF BEMIDJI	7010101	MN	47.45333	-94.7122	1,639.3	9/22/1987	9/27/1994	53	0.731	0.670	122,856	164,332	9,939
MU01	Muskingum River near Devola, OH	5040004	OH	39.47028	-81.4911	20,773.8	7/8/1976	9/18/2007	221	2.290	2.210	22,296,615	19,092,812	1,314,496
NF01	Ohio River near Stratton, OH	5030101	WV	40.52754	-80.6259	61,752.2	7/16/2002	9/17/2007	31	1.507	1.425	57,083,730	47,995,385	4,620,017
NF03	Great Miami River near Elizabethtown, OH	5080002	OH	39.15326	-84.7952	13,864.7	6/19/1975	9/14/2007	267	4.404	4.280	19,695,327	30,889,325	1,251,190
NF04	Green River near Quinn Landing, KY	5110005	KY	37.6473	-87.4956	21,392.3	10/11/1976	9/13/2007	255	1.585	1.480	19,471,305	27,027,433	999,984
OBS140-0004	Blue River	5140104	IN	38.43389	-86.1917	724.9	10/16/1985	3/2/2006	103	2.616	2.530	1,380,881	1,014,142	107,347
OBS150-0008	Blue River	5140104	IN	38.22056	-86.2983	1,286.6	4/7/1999	2/8/2006	81	2.372	2.400	2,062,040	1,530,947	118,786
OR15	Ohio River near Louisville, KY	5140101	KY	38.28244	-85.7039	235,672.3	10/12/1976	9/18/2007	268	1.804	1.720	204,007,764	218,099,355	7,693,802
OR21	Ohio River near Smithland, KY	5140203	KY	37.14613	-88.4116	371,652.1	1/19/1982	9/20/2007	110	1.871	1.790	479,188,761	473,974,896	34,738,541
P06S08	MAUMEE R. AT EATER RD. (RM 91.48)	4100005	OH	41.22194	-84.6697	5,469.3	7/24/1984	9/30/1997	55	3.952	3.700	9,152,250	20,697,423	748,099
PRI002	TUG FORK AT KERMIT, W. VA.	5070201	WV	37.838	-82.4096	3,309.5	2/19/1985	9/21/2006	193	0.820	0.762	1,620,251	1,013,985	132,872
PRI003	TUG FORK AT STATE LINE	5070201	WV	37.56611	-82.1439	2,127.2	3/27/1979	9/21/2006	102	0.592	0.552	852,954	580,808	125,934
PRI008	SOUTH FORK CUMBERLAND RIVER AT BLUE HERON	5130104	KY	36.67028	-84.5489	2,463.2	10/12/1983	8/22/2006	74	0.353	0.319	785,125	1,637,637	200,719
PRI010	ROCKCASTLE RIVER AT BILLOWS	5130102	KY	37.17139	-84.2967	1,558.2	3/20/1979	6/19/2006	254	0.603	0.573	574,875	946,572	39,949
PRI014	ROUGH RIVER NEAR DUNDEE	5110004	KY	37.56278	-86.7708	1,991.8	6/20/1979	12/12/2006	176	1.211	1.124	1,148,940	1,710,301	63,099
PRI021	NOLIN RIVER AT WHITE MILLS	5110001	KY	37.55502	-86.0319	919.4	3/25/1980	10/18/2006	257	3.100	3.102	1,507,351	1,753,355	57,232
PRI024	KENTUCKY RIVER AT FRANKFORT	5100205	KY	38.21286	-84.8726	14,045.3	3/13/1979	1/19/2006	269	1.117	1.041	7,457,412	8,870,432	406,157
PRI025	KENTUCKY RIVER AT CAMP NELSON	5100205	KY	37.76917	-84.6172	11,732.2	1/23/1980	2/20/1997	194	1.001	0.895	5,513,831	6,372,141	263,628
PRI030	POND CREEK NEAR LOUISVILLE	5140102	KY	38.11998	-85.796	166.9	1/18/1979	4/9/1998	218	3.853	3.340	253,631	168,649	15,365
PRI031	NORTH FORK KENTUCKY RIVER AT JACKSON	5100201	KY	37.55111	-83.3844	2,853.8	4/19/1984	9/19/2006	204	0.698	0.661	1,104,778	918,228	76,317
PRI034	SOUTH ELKHORN CREEK NEAR MIDWAY	5100205	KY	38.14139	-84.6453	272.0	4/12/1984	5/19/1999	162	7.349	6.612	980,162	532,547	62,835
PRI043	LITTLE RIVER NEAR CADIZ	5130205	KY	36.84056	-87.7775	697.5	10/15/1985	8/9/2006	185	3.404	3.396	1,505,147	1,238,485	77,634
PRI046	RED RIVER AT CLAY CITY	5100204	KY	37.86528	-83.9333	938.4	10/17/1985	5/12/2006	175	0.590	0.499	318,035	541,738	31,554
PRI048	TYGARTS CREEK NEAR LYNN	5090103	KY	38.59997	-82.9524	714.8	11/14/1985	7/18/2006	190	0.719	0.638	259,566	397,194	18,814
PRI049	LITTLE SANDY RIVER NEAR ARGILLITE	5090104	KY	38.49056	-82.8342	1,397.8	11/14/1985	7/18/2006	192	0.846	0.784	509,374	719,741	27,813

PRI051	HORSE LICK CREEK NEAR LAMERO	5130102	KY	37.32012	-84.1385	159.7	10/20/1992	6/19/2006	98	0.357	0.305	35,733	84,843	5,459
PRI052	SALT RIVER NEAR GLENSBORO	5140102	KY	38.00222	-85.0597	445.5	5/16/1989	12/19/2006	177	2.147	2.116	833,689	627,795	74,080
PRI055	GREEN RIVER NEAR LIVERMORE	5110003	KY	37.4782	-87.1269	15,743.0	7/10/1991	12/12/2006	137	1.540	1.453	13,685,309	20,198,834	661,863
PRI058	KENTUCKY RIVER NEAR TRAPP	5100204	KY	37.84667	-84.0811	8,383.3	2/13/1991	11/20/2006	128	0.544	0.518	2,665,876	3,367,537	196,939
PRI060	NORTH FORK LICKING RIVER NEAR MILFORD	5100101	KY	38.5812	-84.1656	738.5	7/15/1991	11/30/2006	125	1.550	1.374	904,457	856,263	98,300
PRI061	LICKING RIVER AT CLAYSVILLE	5100101	KY	38.52056	-84.1833	5,097.3	2/21/1991	9/27/2006	78	0.828	0.727	2,628,117	3,620,546	207,469
PRI064	LEVISA FORK AT LOUISA	5070203	KY	38.08056	-82.6003	6,001.1	6/26/1991	11/16/2006	122	0.681	0.660	1,902,744	1,810,692	120,419
PRI067	KENTUCKY RIVER AT HIGH BRIDGE	5100205	KY	37.81914	-84.7065	11,888.7	11/18/1997	9/18/2006	53	0.943	0.929	4,708,948	6,576,879	270,111
PRI069	RED RIVER NEAR KEYSBURG	5130206	TN	36.64081	-86.9791	1,226.3	6/10/1998	9/19/2006	44	4.395	4.558	2,834,143	2,790,711	203,672
PRI077	RUSSELL CREEK NEAR GRESHAM	5110001	KY	37.16781	-85.4702	685.4	6/8/1998	9/19/2006	55	1.334	1.153	1,512,938	1,174,890	471,347
PRI086	CUMBERLAND RIVER AT CALVIN	5130101	KY	36.72214	-83.6255	1,345.6	7/23/1998	9/19/2005	37	0.506	0.426	683,689	587,347	114,235
PRI087	CLEAR FORK NEAR WILLIAMSBURG	5130101	KY	36.72589	-84.1424	918.6	7/8/1998	8/18/2004	34	0.346	0.309	172,017	419,522	49,319
PRI092	GOOSE CREEK NEAR ONEIDA	5100203	KY	37.23703	-83.671	663.0	6/10/1998	9/19/2006	38	0.580	0.581	265,767	280,762	80,442
PRI100	FLOYDS FORK NEAR SHEPHERDSVILLE	5140102	KY	38.035	-85.6594	673.7	6/16/1998	12/19/2006	57	1.820	1.668	738,023	720,420	87,605
PRI102	HINKSTON CREEK AT RUDDLES MILLS	5100102	KY	38.30461	-84.2379	671.3	7/28/1998	9/27/2006	52	1.819	1.545	1,020,327	746,858	167,796
PRI105	BRASHEARS CREEK AT TAYLORSVILLE	5140102	KY	38.03722	-85.3406	672.8	8/11/1998	12/19/2006	48	1.765	1.455	2,141,090	1,037,048	343,141
PRI111	LICKING RIVER AT BUTLER	5100101	KY	38.78969	-84.3675	8,767.4	4/20/1999	9/27/2006	47	1.132	0.992	6,425,148	7,518,928	1,148,093
R12P02	KOKOSING R AT TILDEN AVE GAGE - MT. VERNON	5040003	OH	40.40556	-82.4997	522.3	8/15/1974	1/17/1997	84	1.853	1.630	563,133	862,486	35,462
RROT--1---10A53	OTTER TAIL R BRIDGE ON 4TH ST N AT BRECKENRIDGE	9020103	MN	46.26722	-96.5897	4,417.0	10/20/1970	9/29/1994	221	1.369	1.205	747,107	1,557,144	35,410
RRRR296---10E53	RED RIVER AT ALMONTE AVE S IN GRAND FORKS, ND	9020301	ND	47.90778	-97.0256	47,603.4	10/20/1970	9/29/1994	219	1.879	1.700	7,619,824	21,680,023	383,295
RRRR403---10E67	RED RIVER AT BRIDGE ON CSAH-39, 1 MI W OF PERLEY	9020107	MN	47.17979	-96.8247	35,883.6	10/20/1970	9/29/1994	218	2.200	1.990	4,957,298	15,138,777	272,861
RRRR452---10E71	RED RIVER BR ON MAIN AVE AT 3RD ST., IN MOORHEAD	9020104	ND	46.87397	-96.7763	14,792.9	9/20/1971	9/29/1994	211	1.498	1.330	2,466,560	7,120,476	113,056
S000-040	MINNESOTA RIVER AT MN-19 BRIDGE AT HENDERSON	7020012	MN	44.52942	-93.9008	38,613.0	10/6/1970	9/26/1994	222	6.187	5.405	71,172,140	55,649,337	4,280,542
S000-047	STRAIGHT R NEAR CSAH-1 1 MI SE OF CLINTON FALLS	7040002	MN	44.12414	-93.229	654.5	8/11/1977	8/23/2005	155	6.590	6.425	1,531,794	1,773,172	151,475
S000-054	MINNESOTA R CSAH-24 BRIDGE, 1 MI S OF COURTLAND	7020007	MN	44.25444	-94.3414	26,385.1	10/6/1970	9/26/1994	220	3.939	3.100	27,191,539	25,678,705	1,349,338
S000-055	MINNESOTA R BRIDGE ON CSAH-21, 3 MI NE OF DELHI	7020004	MN	44.6195	-95.1779	18,904.8	7/17/2001	8/8/2001	2	3.196	2.980			
S000-056	ST. CROIX R AT MN-48 BR, 3.5 MI W OF DANBURY, WI	7030001	MN	46.01253	-92.444	5,917.6	10/23/1970	9/20/2007	201	0.712	0.650	1,076,354	1,206,737	67,084
S000-095	MISSISSIPPI R LOCK & DAM #6 AT TREMPEALEAU, WIS	7040006	WI	43.99828	-91.4391	149,360.1	10/7/1970	9/21/1994	136	2.466	2.290	86,771,170	113,217,513	2,785,693
S000-111	OTTER TAIL R BR ON CSAH-15 WEST OF FERGUS FALLS	9020103	MN	46.27542	-96.1344	4,032.5	10/18/1982	12/9/2002	54	0.997	0.930	508,464	834,133	30,542
S000-121	KETTLE R BRIDGE ON MN-48, 4.5 MI E OF HINCKLEY	7030003	MN	46.01086	-92.8398	2,556.8	10/23/1970	9/7/2007	220	0.987	0.950	714,582	703,492	38,105
S000-136	CEDAR RIVER AT CSAH-4, 3 MILES SOUTH OF AUSTIN	7080201	MN	43.60522	-92.9845	1,230.6	10/27/1970	9/8/1994	218	6.803	6.400	3,538,609	3,410,124	161,770
S000-156	W FK DES MOINES R CSAH-23 BRIDGE S OF PETERSBURG	7100002	MN	43.52617	-94.9192	3,291.0	10/14/1970	10/9/2002	130	4.821	4.650	6,457,899	3,931,525	459,634
S000-159	YELLOW MEDICINE R MN-67 BR 7 MI SE GRANITE FALLS	7020004	MN	44.72881	-95.4438	1,616.1	10/15/1970	7/17/2001	129	2.541	1.728	1,742,734	1,708,914	202,619
S000-186	TWO RIVERS ON US-75, 1 MI N OF HALLOCK	9020312	MN	48.78925	-96.9555	1,544.0	9/21/1971	6/12/2002	47	2.098	1.630	469,507	777,946	135,113
S000-268	ZUMBRO R S FORK AT CSAH-14, 3 MI N OF ROCHESTER	7040004	MN	44.108	-92.4475	871.4	4/6/1981	9/8/1994	127	6.579	6.420	1,459,374	1,181,187	38,439
S000-287	MISSISSIPPI R LOCK & DAM #5 3 MI SE OF MINNEISKA	7040003	MN	44.16011	-91.8116	146,351.0	5/21/1974	9/21/1994	187	2.656	2.350	100,684,212	111,970,052	3,504,905
S000-299	REDWOOD R AT BRIDGE ON CSAH-101 AT N REDWOOD	7020006	MN	44.56572	-95.0998	1,760.2	6/4/1974	9/4/2001	167	5.296	5.080	2,956,121	1,963,174	408,669
S001-131	LOST R AT CSAH-5 AT OKLEE	9020305	MN	47.84397	-95.8585	570.8	8/27/1987	7/9/2007	41	1.630	1.290	172,922	162,118	33,460
S002-088	THIEF R ON CSAH-7 BRG IN AGASSIZ NWR, 6 MI E OF HO	9020304	MN	48.30045	-96.0708	1,438.5	2/23/1993	10/11/2006	25	2.103	1.833			
S002-118	CLEARWATER R AT KLONDIKE BRIDGE/BOTTINEAU AVE RED	9020305	MN	47.88607	-96.2769	3,155.6	1/19/1992	5/2/2006	82	1.643	1.340	841,373	963,862	127,020
S002-124	CLEARWATER R AT RD IN T151R42S45W 1 MI N PLUMMER	9020305	MN	47.9233	-96.0459	1,325.4	4/27/1992	9/4/2007	94	1.601	1.360	293,312	396,943	28,171
S002-283	MISSISSIPPI R AT FOREST ROUTE 2171, 9 MI NE OF CAS	7010101	MN	47.44445	-94.4252	2,762.7	3/31/2004	11/2/2004	16	1.281	1.110			
S002-365	TWO RIVERS, S BR AT US-59 AT LAKE BRONSON	9020312	MN	48.7326	-96.6675	1,210.3	4/8/1993	6/12/2002	4	1.576	1.436			
S002-963	RED LK R AT 2ND AVE NE (MURRAY BRIDGE) IN EAST GRA	9020303	MN	47.92372	-97.016	13,535.3	10/20/1970	11/3/2004	247	1.398	1.260	2,115,828	3,001,687	120,168
SL 191	ST. LOUIS RIVER AT SH-23 BRIDGE	4010201	MN	46.65882	-92.2842	9,027.3	10/23/1970	9/12/1994	380	1.061	1.040	2,473,429	1,408,906	53,263
SL 261	ST. LOUIS R.-N.THOMSON RESERVIOR	4010201	MN	46.66417	-92.4049	8,862.7	6/4/1974	7/17/1990	158	1.053	1.040	2,350,435	1,376,283	54,769
SL 303	ST. LOUIS R. AT SCANLON HYDO DAM	4010201	MN	46.70969	-92.4201	8,661.6	1/29/1974	7/17/1990	179	1.075	1.035	2,395,905	1,333,860	55,050
SL 500	ST. LOUIS RIVER AT COUNTY RD. 31	4010201	MN	46.86972	-92.6028	6,014.7	6/4/1974	7/17/1990	159	1.181	1.190	1,823,974	1,045,553	54,020
SR01	Scioto River at Lucasville, OH	5060002	OH	38.88111	-83.0175	15,984.7	9/7/1976	9/18/2007	198	3.811	3.330	19,798,858	37,118,724	1,606,364
UMI050-0006	Iroquois River	7120002	IN	40.82028	-87.4642	1,411.1	3/16/1999	2/15/2006	75	6.622	7.100	5,239,842	5,495,377	482,718
UMK030-0002	Kankakee River	7120001	IN	41.47722	-86.6044	966.5	1/30/1980	11/30/2000	226	2.392	1.900	756,565	1,957,857	42,901
UMK030-0020	Kankakee River	7120001	IN	41.46139	-86.6139	980.3	1/25/2001	2/15/2006	62	2.097	1.800	775,817	2,042,725	56,939
UMK060-0001	Yellow River	7120001	IN	41.3025	-86.6014	1,063.8	3/16/1999	2/15/2006	82	4.772	4.150	2,810,797	2,665,997	319,715
UMK080-0001	Kankakee River	7120001	IN	41.21974	-86.9687	3,441.1	3/16/1999	2/15/2006	80	2.779	2.200	4,515,538	6,741,157	360,818
UMK090-0001	Crooked Cr	7120001	IN	41.28222	-87.0258	224.8	3/16/1999	2/15/2006	76	1.008	0.800	112,689	452,310	11,878
V02P16	SCIOTO R. AT WARRENSBURG - OSTRANDER RD.	5060001	OH	40.30583	-83.1681	1,968.4	9/4/1979	10/2/1995	30	4.679	3.970	4,909,162	8,445,667	992,934
WAE050-0001	Eel River	5120104	IN	40.94806	-85.8911	1,284.0	1/16/1985	2/22/2006	109	3.813	3.000	3,219,541	3,039,958	287,899
WAW010-0063	Wildcat Cr	5120107	IN	40.48611	-86.1075	509.4	4/24/1974	2/14/2006	201	5.521	5.700	1,917,315	1,896,466	164,681
WAW020-0004	Wildcat Cr	5120107	IN	40.47361	-86.1842	646.7	4/24/1974	2/14/2006	211	6.726	6.600	1,646,307	2,463,890	87,962
WAW020-0039	Wildcat Cr	5120107	IN	40.48167	-86.53	981.2	9/23/1998	2/22/2006	89	4.811	4.200	2,624,308	3,960,416	193,589

WAW050-0005	Wildcat Cr	5120107	IN	40.45361	-86.8514	2,077.1	3/13/1980	2/22/2006	118	4.648	4.100	6,120,870	8,459,749	507,535
WBU100-0001	Wabash River	5120111	IN	39.22722	-87.5742	33,226.7	1/16/1980	2/1/2006	297	4.718	4.600	70,235,610	95,416,759	2,468,853
WBU160-0002	Busseron Cr	5120111	IN	38.97389	-87.4258	591.6	3/10/1999	11/12/2003	63	1.823	1.700	488,987	776,281	41,439
WBU200-0003	Wabash River	5120111	IN	38.68125	-87.5347	35,672.3	4/2/1996	2/13/2006	96	4.526	4.600	76,985,736	99,216,087	3,843,579
WDE010-0007	Wabash River	5120105	IN	40.73667	-86.505	9,981.3	1/17/1980	2/22/2006	165	4.560	4.000	21,540,956	37,667,803	940,423
WDE060-0001	Wabash River	5120105	IN	40.52861	-86.7603	11,496.6	2/21/2001	2/22/2006	61	5.203	4.600	26,984,748	43,309,191	1,904,487
WDE070-0006	Wabash River	5120105	IN	40.49556	-86.8233	16,696.8	1/22/1991	12/5/2000	112	4.863	4.800	37,973,592	55,202,087	2,236,938
WED010-0005	Big Blue River	5120204	IN	39.87361	-85.4389	149.4	5/29/1996	9/23/2003	76	8.105	7.400	390,427	300,043	22,218
WED050-0001	Big Blue River	5120204	IN	39.355	-85.9839	1,511.6	3/26/1980	2/9/2006	121	4.506	4.300	3,713,662	5,036,236	169,529
WEF050-0002	Flatrock River	5120205	IN	39.36361	-85.8553	1,056.8	4/15/1999	3/2/2006	83	4.708	5.200	4,521,454	4,142,345	647,816
WEF060-0003	E Fk White River	5120205	IN	39.20028	-85.9264	4,070.1	4/18/1996	2/8/2006	122	3.924	3.800	9,338,737	13,208,072	363,833
WEL040-0003	E Fk White River	5120208	IN	38.82583	-86.5133	10,459.6	5/12/1980	2/21/2006	114	2.863	2.800	20,601,967	23,494,902	1,105,173
WEL100-0002	E Fk White River	5120208	IN	38.80194	-86.645	12,221.4	2/10/1977	2/21/2006	304	2.725	2.500	18,617,367	24,525,655	737,391
WEL170-0001	E Fk White River	5120208	IN	38.53917	-87.2231	14,877.0	1/15/1980	2/28/2006	193	2.595	2.500	22,205,470	28,056,803	892,823
WEM070-0001	Vernon Fk Muscatatuck Riv	5120207	IN	38.97639	-85.62	516.4	7/9/1998	3/1/2006	103	2.192	2.100	564,897	904,941	57,775
WEU040-0001	E Fk White River	5120206	IN	38.98722	-85.8989	6,043.9	2/9/1977	2/9/2006	198	4.075	4.100	15,551,643	17,732,545	595,663
WLV030-0003	Wabash River	5120108	IN	40.41194	-87.0364	19,508.1	2/2/1977	2/2/2006	296	4.858	4.800	46,089,461	65,689,218	1,589,961
WLV080-0003	Wabash River	5120108	IN	40.255	-87.2997	21,085.4	3/15/1999	2/2/2006	81	5.008	4.900	51,238,125	68,698,002	2,632,564
WLV140-0001	Wabash River	5120108	IN	39.95167	-87.4192	25,311.8	1/16/1980	2/1/2006	148	4.959	4.900	60,391,776	78,471,763	2,378,972
WLV150-0001	Wabash River	5120108	IN	39.79222	-87.3744	28,903.1	6/29/1982	2/1/2006	90	5.135	4.950	68,626,594	87,258,697	3,275,212
WLV160-0001	Big Raccoon Cr	5120108	IN	39.79028	-86.9586	456.5	3/29/1999	2/1/2006	82	4.122	3.750	1,960,552	1,374,930	226,888
WLV190-0012	Big Raccoon Cr	5120108	IN	39.72917	-87.325	1,229.2	4/28/1999	2/1/2006	82	2.581	2.350	1,594,946	2,263,060	99,038
WLV200-0001	Wabash River	5120108	IN	39.65639	-87.3956	30,428.1	2/3/1977	2/1/2006	154	4.828	4.800	70,637,862	90,009,391	2,989,675
WMI060-0004	Mississinewa River	5120103	IN	40.62806	-85.7358	1,838.7	2/22/1977	2/14/2006	157	4.407	3.900	4,166,465	7,738,103	279,980
WMI060-0005	Mississinewa River	5120103	IN	40.57611	-85.6597	1,751.4	2/22/1977	2/14/2006	258	4.115	3.746	4,585,766	7,324,321	322,001
WMI060-0006	Mississinewa River	5120103	IN	40.74917	-86.0119	2,103.8	5/3/1995	2/22/2006	97	4.159	4.072	4,549,023	8,544,423	384,416
WPA040-0003	Patoka River	5120209	IN	38.32972	-86.9664	1,126.3	3/2/1977	2/27/2006	146	3.030	2.700	1,784,928	1,597,360	152,161
WPA060-0002	Patoka River	5120209	IN	38.3825	-87.3333	1,688.0	6/26/1996	2/27/2006	99	2.782	2.400	3,117,123	2,595,523	282,401
WQN0001	GENESEE RIVER	4130002	PA	41.9942	-77.8703	222.1								
WQN0643	CONNEAUT CREEK	4120101	PA	41.918	-80.4692	398.2								
WQN0702	MONONGAHELA RIVER	5020005	PA	40.1519	-79.9042	13,502.5	10/26/1978	10/5/1987	42	1.173	1.066	10,074,225	7,674,910	738,509
WQN0726	CASSELMAN RIVER	5020006	PA	39.7325	-79.1009	193.7								
WQN0801	ALLEGHENY RIVER	5010009	PA	40.52745	-79.846	29,846.8								
WQN0802	ALLEGHENY RIVER	5010006	PA	40.7611	-79.5458	23,274.4	10/31/1978	5/6/1993	102	0.986	0.906	15,118,525	15,088,033	750,193
WQN0807	ALLEGHENY RIVER	5010001	PA	41.965	-78.3861	1,422.6								
WQN0843	CLARION RIVER	5010005	PA	41.1296	-79.5548	3,003.1								
WQN0845	FRENCH CREEK	5010004	PA	41.402	-79.8316	3,187.4								
WQN0846	FRENCH CREEK	5010004	PA	41.5893	-80.1502	2,094.4								
WQN0861	MAHONING CREEK	5010006	PA	40.9221	-79.0064	411.4								
WQN0867	ALLEGHENY RIVER	5010003	PA	41.2638	-79.8415	16,226.1								
WQN0869	FRENCH CREEK	5010004	PA	41.7717	-80.1076	1,542.2								
WQN0901	OHIO RIVER	5030101	OH	40.62058	-80.5589	60,905.2	8/20/1975	9/9/1986	2	1.828	1.828			
WQN0902	OHIO RIVER	5030101	PA	40.533	-80.1881	50,462.4								
WSA020-0002	Salamonie River	5120102	IN	40.55917	-85.2786	657.7	7/28/1998	2/13/2006	83	4.403	3.900	1,925,210	2,622,052	252,130
WSA040-0001	Salamonie River	5120102	IN	40.82972	-85.7189	1,452.7	6/5/1980	2/22/2006	114	4.483	4.200	2,895,907	5,010,103	245,307
WSA040-0005	Salamonie River	5120102	IN	40.74167	-85.5089	1,154.5	10/4/1971	2/13/2006	178	4.141	3.500	3,678,800	4,779,341	482,994
WSU020-0003	Sugar Cr	5120110	IN	40.14333	-86.6958	749.7	3/15/1999	2/2/2006	81	5.919	5.625	3,527,420	2,103,134	394,200
WSU050-0002	Sugar Cr	5120110	IN	40.05	-86.9225	1,338.7	3/15/1999	2/2/2006	83	5.757	5.300	4,710,264	4,208,369	445,826
WSU050-0005	Sugar Cr	5120110	IN	39.94611	-87.0586	1,721.4	6/29/1982	2/1/2006	83	4.811	4.400	6,306,744	5,075,866	634,509
WTI050-0009	Tippecanoe River	5120106	IN	41.09417	-86.2403	1,629.7	7/22/1991	2/8/2006	97	2.978	2.800	2,687,596	2,898,706	141,341
WTI080-0001	Tippecanoe River	5120106	IN	41.00667	-86.6028	2,695.6	3/25/1998	2/15/2006	94	3.149	2.735	5,020,604	5,460,680	414,391
WTI150-0011	Tippecanoe River	5120106	IN	40.59389	-86.7708	4,868.3	1/20/1998	2/22/2006	99	3.610	3.100	9,314,723	10,795,175	636,575
WUW060-0002	Wabash River	5120101	IN	40.61667	-84.965	1,233.8	1/27/1998	12/2/2002	60	7.333	6.500	6,689,760	6,814,731	981,376
WUW070-0002	Wabash River	5120101	IN	40.81944	-85.3425	1,661.0	8/19/1992	1/21/2003	62	7.906	7.100	6,923,129	8,931,989	1,319,584
WUW090-0001	Wabash River	5120101	IN	40.85833	-85.5075	2,000.7	1/28/1998	1/21/2003	60	7.080	6.350	8,118,956	10,257,774	1,657,804
WUW120-0002	Little River	5120101	IN	40.89861	-85.4133	702.6	2/25/1998	2/6/2006	91	4.262	3.550	2,022,914	2,395,413	217,519
WUW140-0001	Wabash River	5120101	IN	40.86889	-85.6019	2,987.6	1/10/1980	1/21/2003	120	6.020	5.150	7,655,810	13,551,179	818,623
WUW160-0006	Wabash River	5120101	IN	40.74222	-86.0967	7,083.3	1/16/1973	2/22/2006	110	5.135	4.850	15,394,352	28,905,588	877,603
WUW170-0002	Pipe Cr	5120101	IN	40.72167	-86.1983	452.3	3/25/1998	9/9/2003	63	4.895	4.150	1,295,615	2,229,012	133,640
WVE100-0001	Vermillion River	5120109	IN	39.96139	-87.4519	3,705.7	1/16/1980	2/1/2006	149	6.761	6.900	10,191,536	8,727,539	543,579

WWE060-0004	Mill Cr	5120203	IN	39.63667	-86.6406	144.4	10/1/1974	9/24/2003	131	2.857	2.600	363,105	241,069	37,709
WWE080-0001	Eel River	5120203	IN	39.35056	-87.0725	2,256.0	3/17/1999	2/1/2006	83	2.741	2.800	4,194,455	3,739,200	247,126
WWE090-0001	Eel River	5120203	IN	39.12423	-86.9702	3,124.8	6/26/1996	2/14/2006	98	2.550	2.600	5,705,147	5,070,776	352,706
WWL020-0003	W Fk White River	5120202	IN	39.28028	-86.7619	7,765.8	4/2/1996	2/14/2006	119	4.094	3.800	12,367,978	20,319,289	524,782
WWL030-0003	W Fk White River	5120202	IN	39.11194	-86.9625	11,405.4	4/20/1999	2/14/2006	82	3.716	3.550	17,665,952	25,731,293	875,082
WWL070-0003	W Fk White River	5120202	IN	38.795	-87.2417	12,919.1	4/2/1996	2/13/2006	119	3.122	3.100	18,911,163	28,091,212	724,006
WWL100-0005	White River	5120202	IN	38.51167	-87.2886	28,823.8	2/9/1977	2/28/2006	301	3.079	2.900	43,997,556	59,096,741	1,497,644
WWU010-0001	W Fk White River	5120201	IN	40.17833	-85.3422	581.5	2/24/1977	2/15/2006	175	2.859	2.300	1,349,240	2,246,696	118,727
WWU010-0006	W Fk White River	5120201	IN	40.18222	-84.9689	88.4	4/9/1980	9/23/2003	260	3.788	3.300	264,873	342,066	33,107
WWU020-0005	W Fk White River	5120201	IN	40.17889	-85.495	655.3	1/9/1980	2/15/2006	260	4.278	4.000	1,228,181	2,788,056	61,131
WWU030-0003	W Fk White River	5120201	IN	40.10667	-85.6728	1,059.2	2/24/1977	2/15/2006	264	3.372	3.000	1,885,798	3,738,397	84,799
WWU040-0004	W Fk White River	5120201	IN	40.14222	-85.8628	1,480.1	8/19/1992	2/13/2006	119	3.438	3.200	2,218,866	5,423,902	85,792
WWU090-0002	W Fk White River	5120201	IN	39.91	-86.105	3,182.6	1/22/1980	2/15/2006	185	4.200	3.900	6,761,876	11,249,214	275,997
WWU110-0001	Fall Cr	5120201	IN	39.78194	-86.1767	861.3	12/8/1988	2/15/2006	195	2.532	2.400	945,300	1,634,962	47,131
WWU110-0002	Fall Cr	5120201	IN	39.83444	-86.1219	803.5	1/22/1980	2/15/2006	180	2.185	1.900	974,227	1,595,261	57,115
WWU120-0001	Eagle Cr	5120201	IN	39.735	-86.1967	539.3	1/22/1980	2/15/2006	306	4.461	4.100	492,796	725,967	25,542
WWU120-0002	Eagle Cr	5120201	IN	39.77833	-86.2506	449.2	3/16/1989	2/15/2006	197	2.253	2.000	473,811	621,501	28,755
WWU140-0003	W Fk White River	5120201	IN	39.56694	-86.2558	5,259.9	10/6/1992	2/21/2006	153	5.552	5.117	9,394,952	16,971,854	441,202
WWU150-0007	White Lick Cr	5120201	IN	39.51361	-86.3803	746.8	3/18/1999	2/21/2006	79	2.761	2.500	1,589,920	1,774,337	158,533
WWU160-0004	W Fk White River	5120201	IN	39.43389	-86.4494	6,462.6	2/10/1977	2/21/2006	117	4.464	4.300	11,083,301	19,265,713	410,612
YOU0925	YOUGH. R. GA. 0.7M. UPST.FR.BR.ON RT 42 AT FD	5020006	MD	39.65335	-79.4079	763.6	1/7/1985	12/5/1995	82	0.918	0.883	587,470	458,531	34,499
YOU1139	YOUGH. R. N OF RT 20 BR.DOWNST.FR. LT. YOUGH.	5020006	MD	39.42472	-79.4208	349.7	9/16/1980	12/5/1995	89	1.303	1.205	355,562	276,964	22,092

Table S5_TP. Total annual nitrogen and phosphorus loads and yields, with confidence intervals, for HUC8 watersheds of Major River Basin 3 (MRB3).
Accumulated nondelivered loads and yields from each individual HUC8 (based on sum of incremental loads), and does not include loads from upstream HUC8s.
Direct results from the SPARROW models: not corrected for any spatial biases.

Supplemental Material to Robertson and Saad, 2011, Journal of the American Water Resources Association, Nutrient Inputs to the Laurentian Great Lakes By Source and River Basin Estimated Using SPARROW Watershed Models.

The total load for each HUC8 watershed was calculated by summing the simulated incremental loads from each SPARROW reach within a HUC8 watershed.
Yield is load per unit area of the HUC8 watershed.
[%, percent; CI, Confidence interval; kg, kilogram; ha, hectare]

HUC8's are based on Seaber, P.R., F.P. Kapinos, and G.L. Knapp, 1987. Hydrologic Unit maps: U.S. Geol. Surv. Water-Supply Paper 2294, 63 p. Available online at: http://water.usgs.gov/GIS/huc.html

									Percent Contribution by Source					
HUC8	HUC8 NAME	Great Lake/Basin	Total Phosphorus Load (kg)	Lower 95% CI Total Phosphorus Load (kg)	Upper 95% CI Total Phosphorus Load (kg)	Total Phosphorus Yield (kg/km^2)	Lower 95% CI Total Phosphorus Yield (kg/km^2)	Upper 95% CI Total Phosphorus Yield (kg/km^2)	Forest, wetland, shrub	Point sources	Urban and open areas	Fertilizers (farm)	Manure (confined)	Manure (unconfined)
04010101	Baptism-Brule	Lake Superior	49,426	20,259	92,171	8.46	3.47	15.78	96.6	0.5	2.8	0.0	0.0	0.1
04010102	Beaver-Lester	Lake Superior	67,504	26,878	116,634	39.50	15.73	68.25	37.1	53.6	8.1	0.2	0.5	0.5
04010201	St. Louis	Lake Superior	116,759	42,725	208,191	15.73	5.76	28.04	66.2	14.7	12.5	1.0	2.7	2.8
04010202	Cloquet	Lake Superior	12,540	5,158	26,323	6.32	2.60	13.26	91.6	0.0	5.9	0.4	0.9	1.3
04010301	Beartrap-Nemadji	Lake Superior	101,507	43,228	186,681	20.98	8.94	38.59	60.4	12.2	10.6	1.6	10.3	4.9
04010302	Bad-Montreal	Lake Superior	72,580	30,212	156,511	21.74	9.05	46.88	72.8	5.9	8.7	1.6	7.7	3.3
04020101	Black-Presque Isle	Lake Superior	49,723	20,107	110,848	18.87	7.63	42.07	85.0	6.6	8.0	0.0	0.2	0.2
04020102	Ontonagon	Lake Superior	44,586	17,576	86,723	12.32	4.86	23.96	84.7	1.4	9.4	0.4	2.3	1.8
04020103	Keweenaw Peninsula	Lake Superior	57,880	22,548	96,710	20.20	7.87	33.75	51.4	36.4	10.6	0.3	0.8	0.5
04020104	Sturgeon	Lake Superior	23,496	8,185	56,745	12.71	4.43	30.70	85.0	0.0	9.7	0.8	3.0	1.4
04020105	Dead-Kelsey	Lake Superior	29,758	10,188	63,409	12.55	4.30	26.74	58.6	21.0	19.1	0.1	0.3	0.9
04020201	Betsy-Chocolay	Lake Superior	37,752	13,981	65,526	12.62	4.67	21.90	49.4	36.1	13.0	0.2	0.5	0.7
04020202	Tahquamenon	Lake Superior	19,148	7,772	34,174	8.96	3.64	15.99	77.6	5.8	15.3	0.3	0.4	0.6
04020203	Waiska	Lake Superior	13,452	5,168	26,335	17.11	6.57	33.49	34.7	44.7	13.2	0.5	3.8	3.0
04030101	Manitowoc-Sheboygan	Lake Michigan	347,506	127,046	682,374	81.46	29.78	159.95	3.7	21.1	5.6	12.4	50.2	6.9
04030102	Door-Kewaunee	Lake Michigan	123,774	45,933	236,297	62.56	23.22	119.43	7.6	18.7	6.5	13.7	46.9	6.4
04030103	Duck-Pensaukee	Lake Michigan	63,133	23,333	113,640	48.91	18.08	88.03	7.0	3.7	9.9	16.2	54.7	8.5
04030104	Oconto	Lake Michigan	59,836	22,082	114,506	22.16	8.18	42.40	28.0	4.8	14.0	12.1	34.6	6.4
04030105	Peshtigo	Lake Michigan	41,394	16,270	68,539	13.85	5.44	22.93	41.3	6.2	17.5	7.9	21.8	5.1
04030106	Brule	Lake Michigan	38,983	14,245	69,146	14.33	5.24	25.42	78.3	3.5	13.9	1.5	1.6	1.2
04030107	Michigamme	Lake Michigan	15,393	6,640	27,885	8.40	3.63	15.22	84.1	2.6	12.3	0.2	0.2	0.6
04030108	Menominee	Lake Michigan	108,862	45,561	196,117	18.22	7.63	32.83	41.8	32.7	14.5	2.4	6.6	2.0
04030109	Cedar-Ford	Lake Michigan	49,031	16,593	96,520	18.44	6.24	36.30	55.0	17.7	13.4	3.0	8.2	2.8
04030110	Escanaba	Lake Michigan	32,045	13,008	59,152	13.41	5.45	24.76	58.5	18.0	17.3	1.6	3.1	1.5
04030111	Tacoosh-Whitefish	Lake Michigan	21,640	8,406	38,609	13.02	5.06	23.22	64.2	8.9	21.6	1.3	2.4	1.6
04030112	Fishdam-Sturgeon	Lake Michigan	14,383	5,758	26,302	9.56	3.83	17.49	68.2	0.0	21.3	2.9	4.8	2.7
04030201	Upper Fox	Lake Michigan	122,967	42,516	215,393	28.39	9.82	49.74	10.0	15.5	11.6	23.8	33.2	5.8
04030202	Wolf	Lake Michigan	239,207	112,646	476,824	24.88	11.72	49.59	18.4	7.1	10.2	16.7	41.3	6.4
04030203	Lake Winnebago	Lake Michigan	57,378	17,766	107,721	43.06	13.33	80.83	4.7	9.6	5.5	19.2	53.4	7.7
04030204	Lower Fox	Lake Michigan	221,418	79,835	440,280	187.53	67.62	372.90	1.2	44.5	8.2	8.3	32.9	5.0
04040001	Little Calumet-Galien	Lake Michigan	130,350	51,635	295,437	71.14	28.18	161.23	5.1	55.5	22.5	12.5	3.4	1.0
04040002	Pike-Root	Lake Michigan	260,895	95,797	528,655	247.56	90.90	501.63	1.3	79.4	10.7	4.7	3.4	0.6
04040003	Milwaukee	Lake Michigan	187,023	70,288	292,976	82.76	31.10	129.65	3.7	48.4	19.2	7.2	18.8	2.7
04050001	St. Joseph	Lake Michigan	535,271	209,721	1,118,905	43.54	17.06	91.00	4.5	23.8	18.5	22.8	26.1	4.2
04050002	Black-Macatawa	Lake Michigan	79,306	31,079	141,461	53.87	21.11	96.09	4.6	21.7	19.4	11.0	41.3	1.9
04050003	Kalamazoo	Lake Michigan	276,671	130,342	620,411	52.71	24.83	118.20	5.0	37.8	14.8	12.4	27.5	2.4

04050004	Upper Grand	Lake Michigan	199,930	76,842	373,628	44.03	16.92	82.29	7.9	30.1	19.3	21.3	17.4	3.9
04050005	Maple	Lake Michigan	92,858	37,737	188,464	37.68	15.31	76.48	5.2	7.4	9.8	37.3	35.5	4.9
04050006	Lower Grand	Lake Michigan	290,578	113,175	596,004	58.44	22.76	119.87	4.9	32.1	18.8	14.3	27.6	2.3
04050007	Thornapple	Lake Michigan	84,944	26,929	178,079	38.12	12.08	79.91	10.3	5.1	14.4	32.9	31.6	5.8
04060101	Pere Marquette-White	Lake Michigan	115,201	44,975	245,709	20.60	8.04	43.94	19.1	38.5	21.5	9.6	9.4	1.8
04060102	Muskegon	Lake Michigan	105,162	37,402	198,052	14.92	5.31	28.10	23.1	13.1	29.9	9.2	20.0	4.7
04060103	Manistee	Lake Michigan	84,277	29,590	166,036	16.70	5.86	32.91	25.4	47.4	20.4	2.2	2.8	1.8
04060104	Betsie-Platte	Lake Michigan	27,005	10,545	66,615	13.08	5.11	32.27	26.9	31.1	30.0	6.5	3.7	1.8
04060105	Boardman-Charlevoix	Lake Michigan	54,541	21,254	85,547	12.44	4.85	19.51	28.4	21.5	35.5	6.1	5.6	2.8
04060106	Manistique	Lake Michigan	31,346	14,407	57,725	8.30	3.81	15.28	62.0	19.9	16.3	0.5	0.6	0.7
04060107	Brevoort-Millecoquins	Lake Michigan	12,585	4,837	22,004	8.20	3.15	14.34	68.6	1.2	25.2	0.5	3.1	1.4
04070001	St. Marys	Lake Huron	22,579	8,143	44,351	17.96	6.48	35.28	41.1	27.1	18.2	1.0	7.9	4.7
04070002	Carp-Pine	Lake Huron	25,230	9,650	52,082	14.91	5.70	30.78	48.2	33.5	14.9	0.2	1.6	1.6
04070003	Lone Lake-Ocqueoc	Lake Huron	67,125	26,658	134,252	32.86	13.05	65.73	19.7	62.2	11.6	2.5	2.6	1.5
04070004	Cheboygan	Lake Huron	11,788	4,048	20,630	5.04	1.73	8.83	43.2	8.6	36.5	2.6	5.7	3.4
04070005	Black	Lake Huron	7,616	3,073	15,409	4.88	1.97	9.86	56.9	0.0	32.0	3.2	4.4	3.5
04070006	Thunder Bay	Lake Huron	45,048	15,831	76,992	13.63	4.79	23.29	36.7	21.6	22.5	5.4	9.4	4.4
04070007	Au Sable	Lake Huron	48,942	16,149	81,816	9.30	3.07	15.54	45.0	1.9	47.5	1.0	2.6	2.1
04080101	Au Gres-Rifle	Lake Huron	48,790	17,242	93,393	18.26	6.45	34.96	26.7	15.0	25.8	10.2	17.5	4.9
04080102	Kawkawlin-Pine	Lake Huron	18,906	6,779	35,940	14.90	5.34	28.32	11.5	4.1	41.1	35.7	6.0	1.7
04080103	Pigeon-Wiscoggin	Lake Huron	109,787	43,399	195,415	50.58	19.99	90.03	1.8	27.9	8.1	31.3	28.5	2.4
04080104	Birch-Willow	Lake Huron	75,269	28,497	165,545	51.63	19.55	113.55	4.0	14.8	8.1	34.9	34.7	3.5
04080201	Tittabawassee	Lake Huron	75,020	29,882	151,020	20.34	8.10	40.95	15.9	35.8	25.8	9.6	9.3	3.6
04080202	Pine	Lake Huron	56,428	21,485	124,539	20.43	7.78	45.10	11.7	19.5	22.0	21.9	20.6	4.4
04080203	Shiawassee	Lake Huron	68,392	28,105	125,100	23.78	9.77	43.49	9.9	15.0	24.6	33.4	13.8	3.3
04080204	Flint	Lake Huron	163,686	62,623	293,494	42.36	16.21	75.95	8.0	41.4	25.5	15.7	7.2	2.1
04080205	Cass	Lake Huron	59,887	22,472	110,373	24.95	9.36	45.97	8.8	10.6	17.8	37.3	21.6	3.9
04080206	Saginaw	Lake Huron	135,956	47,928	294,628	203.99	71.91	442.07	0.2	83.7	8.5	6.2	1.2	0.2
04090001	St. Clair	Lake Erie	139,583	51,498	231,744	41.78	15.42	69.37	8.9	30.3	12.6	27.6	16.5	4.1
04090002	Lake St. Clair	Lake Erie	14,502	5,942	25,212	29.14	11.94	50.67	2.9	41.9	54.0	0.7	0.3	0.2
04090003	Clinton	Lake Erie	177,188	74,267	283,622	68.95	28.90	110.37	3.6	49.5	39.2	5.3	1.8	0.6
04090004	Detroit	Lake Erie	947,079	370,718	1,879,417	377.96	147.95	750.04	0.2	91.6	8.0	0.2	0.1	0.0
04090005	Huron	Lake Erie	97,108	34,313	190,794	40.63	14.36	79.83	6.8	37.6	42.5	7.9	4.2	1.0
04100001	Ottawa-Stony	Lake Erie	60,276	21,157	98,002	35.98	12.63	58.49	3.0	22.9	40.9	25.7	6.6	0.9
04100002	Raisin	Lake Erie	116,104	47,718	248,181	40.84	16.78	87.29	4.4	33.5	15.7	29.2	14.8	2.5
04100003	St. Joseph	Lake Erie	138,424	53,932	288,736	49.26	19.19	102.74	5.4	14.9	11.1	41.5	22.5	4.7
04100004	St. Marys	Lake Erie	278,488	96,661	520,651	135.25	46.95	252.86	0.7	8.1	5.9	24.8	57.8	2.7
04100005	Upper Maumee	Lake Erie	97,959	30,702	238,873	109.90	34.44	267.98	0.9	57.7	7.9	23.1	8.8	1.5
04100006	Tiffin	Lake Erie	83,910	30,815	178,536	40.67	14.94	86.54	4.2	9.9	10.7	47.4	24.8	3.1
04100007	Auglaize	Lake Erie	309,622	126,052	499,052	69.17	28.16	111.48	1.1	19.6	8.1	47.2	22.6	1.5
04100008	Blanchard	Lake Erie	114,672	48,075	210,781	59.76	25.05	109.85	1.3	16.5	10.2	49.9	20.8	1.2
04100009	Lower Maumee	Lake Erie	269,222	99,686	579,134	98.66	36.53	212.24	0.6	62.2	9.3	22.4	5.2	0.4
04100010	Cedar-Portage	Lake Erie	139,994	52,185	264,804	56.06	20.90	106.04	2.1	21.5	12.7	56.4	6.7	0.6
04100011	Sandusky	Lake Erie	317,763	125,188	655,727	66.29	26.12	136.80	2.2	15.8	9.0	52.6	18.4	2.0
04100012	Huron-Vermilion	Lake Erie	184,967	59,161	392,747	93.74	29.98	199.05	5.9	10.9	5.6	56.7	17.7	3.1
04110001	Black-Rocky	Lake Erie	232,917	80,190	402,141	99.30	34.19	171.45	6.7	46.7	17.8	17.4	9.3	2.0
04110002	Cuyahoga	Lake Erie	324,274	123,867	742,931	155.10	59.24	355.33	4.3	73.4	16.6	3.1	1.8	0.8
04110003	Ashtabula-Chagrin	Lake Erie	210,631	79,878	374,752	130.70	49.56	232.53	6.3	64.8	18.7	5.6	3.4	1.1
04110004	Grand	Lake Erie	104,162	36,701	167,823	56.64	19.96	91.26	24.9	4.5	11.1	31.9	21.0	6.6
04120101	Chautauqua-Conneaut	Lake Erie	167,609	63,963	321,052	74.76	28.53	143.20	11.4	52.9	11.5	7.9	13.0	3.3
04120102	Cattaraugus	Lake Erie	52,305	17,748	95,344	36.09	12.25	65.78	26.1	6.7	7.5	14.9	37.4	7.3
04120103	Buffalo-Eighteenmile	Lake Erie	146,941	55,394	285,859	78.91	29.75	153.50	12.3	40.5	13.3	9.5	21.2	3.2
04120104	Niagara	Lake Ontario	518,390	190,483	1,107,598	205.27	75.43	438.59	3.1	78.4	5.5	4.5	7.2	1.3

04130001	Oak Orchard-Twelvemile	Lake Ontario	158,734	75,045	318,243	47.61	22.51	95.46	8.5	45.0	7.2	22.4	13.4	3.5
04130002	Upper Genesee	Lake Ontario	152,743	52,958	281,132	41.58	14.41	76.52	26.2	9.1	5.4	14.6	36.6	8.2
04130003	Lower Genesee	Lake Ontario	201,650	68,694	341,134	73.13	24.91	123.71	8.3	21.2	8.6	22.5	33.0	6.4
04140101	Irondequoit-Ninemile	Lake Ontario	206,172	85,160	437,939	112.96	46.66	239.95	6.4	70.3	7.5	7.1	7.5	1.2
04140102	Salmon-Sandy	Lake Ontario	75,141	23,119	147,503	30.28	9.32	59.43	39.0	24.3	6.0	6.2	20.2	4.4
04140201	Seneca	Lake Ontario	436,812	148,071	802,922	48.98	16.60	90.04	9.9	32.0	8.1	17.8	27.3	4.9
04140202	Oneida	Lake Ontario	95,050	34,525	150,921	24.45	8.88	38.82	27.3	16.0	11.5	11.7	28.4	5.0
04140203	Oswego	Lake Ontario	13,081	5,172	24,765	34.70	13.72	65.69	26.6	27.3	15.1	10.2	16.9	3.8
04150101	Black	Lake Ontario	144,610	50,020	273,349	29.16	10.09	55.13	31.2	31.2	3.6	5.8	24.3	4.0
04150102	Chaumont-Perch	Lake Ontario	56,686	17,954	92,314	61.84	19.58	100.70	18.6	5.0	4.8	11.0	50.8	9.7
04150301	Upper St. Lawrence	Lake Ontario	55,458	20,789	86,452	23.47	8.80	36.59	22.2	12.0	6.6	9.5	41.1	8.6
04150302	Oswegatchie	St.Lawrence	54,482	19,691	97,588	20.84	7.53	37.33	42.8	8.1	5.2	7.2	30.5	6.2
04150303	Indian	St.Lawrence	28,223	10,813	54,279	19.56	7.50	37.63	39.0	3.0	9.8	7.6	33.4	7.2
04150304	Grass	St.Lawrence	42,991	15,460	80,921	23.75	8.54	44.71	42.2	12.4	6.4	6.5	27.1	5.4
04150305	Raquette	St.Lawrence	44,089	17,933	86,221	13.65	5.55	26.70	67.2	10.6	7.2	2.5	10.3	2.3
04150306	St. Regis	St.Lawrence	31,078	12,333	60,588	13.94	5.53	27.17	60.8	12.5	5.4	3.6	14.7	3.0
04150307	English-Salmon	St.Lawrence	35,042	11,419	77,310	20.81	6.78	45.91	41.1	5.6	7.6	8.3	31.8	5.7
05010001	Upper Allegheny	Ohio River	164,803	51,360	279,885	24.73	7.71	42.01	51.9	18.3	7.8	4.8	13.3	3.9
05010002	Conewango	Ohio River	84,627	32,875	159,920	35.74	13.88	67.54	28.8	12.1	8.8	12.0	31.0	7.3
05010003	Middle Allegheny-Tionesta	Ohio River	90,476	41,841	164,105	20.74	9.59	37.61	53.9	10.1	12.1	6.0	12.9	5.1
05010004	French	Ohio River	108,316	37,847	197,640	34.03	11.89	62.09	28.9	7.4	12.7	15.5	27.7	7.8
05010005	Clarion	Ohio River	74,447	38,088	139,626	23.06	11.80	43.24	43.2	29.7	12.0	4.1	7.0	4.0
05010006	Middle Allegheny-Redbank	Ohio River	121,427	50,700	249,909	27.88	11.64	57.38	37.4	11.1	17.7	11.8	14.2	7.7
05010007	Conemaugh	Ohio River	116,111	43,266	204,941	32.68	12.18	57.68	28.1	16.3	17.0	13.0	18.6	7.0
05010008	Kiskiminetas	Ohio River	48,600	23,153	84,762	36.41	17.35	63.51	32.5	17.9	18.9	8.3	14.8	7.5
05010009	Lower Allegheny	Ohio River	75,970	28,928	131,835	58.99	22.46	102.37	17.1	38.3	30.5	4.6	5.6	3.8
05020001	Tygart Valley	Ohio River	93,152	37,277	171,767	26.22	10.49	48.34	50.1	10.3	14.7	4.2	2.1	18.6
05020002	West Fork	Ohio River	84,050	35,098	162,494	36.65	15.30	70.85	45.3	16.8	14.4	2.9	1.0	19.6
05020003	Upper Monongahela	Ohio River	57,102	18,391	105,524	47.85	15.41	88.42	30.3	43.2	14.7	1.2	1.4	9.1
05020004	Cheat	Ohio River	68,298	26,752	130,147	18.37	7.20	35.00	60.7	5.5	15.9	2.9	3.3	11.7
05020005	Lower Monongahela	Ohio River	185,751	84,075	323,192	48.70	22.04	84.74	23.6	30.2	22.7	3.9	7.7	11.9
05020006	Youghiogheny	Ohio River	149,306	68,250	283,853	32.94	15.06	62.63	26.9	13.3	17.4	10.6	21.5	10.4
05030101	Upper Ohio	Ohio River	486,333	179,422	903,346	93.96	34.66	174.52	11.2	60.6	12.4	4.5	7.0	4.2
05030102	Shenango	Ohio River	118,416	42,912	216,784	42.89	15.54	78.52	16.3	17.8	16.0	16.1	24.2	9.6
05030103	Mahoning	Ohio River	242,963	77,032	457,656	83.56	26.49	157.40	8.5	50.6	15.5	12.5	10.1	2.9
05030104	Beaver	Ohio River	19,276	7,050	34,199	61.47	22.48	109.06	18.3	33.5	25.4	5.4	12.0	5.5
05030105	Connoquenessing	Ohio River	216,438	82,094	439,629	99.80	37.85	202.71	8.5	68.9	9.0	4.2	6.2	3.3
05030106	Upper Ohio-Wheeling	Ohio River	166,454	63,214	319,622	42.41	16.11	81.44	33.2	22.3	12.5	4.9	10.0	17.1
05030201	Little Muskingum-Middle Island	Ohio River	214,533	88,514	388,710	45.93	18.95	83.23	46.6	24.2	8.5	4.1	5.2	11.4
05030202	Upper Ohio-Shade	Ohio River	183,273	74,682	316,079	50.74	20.67	87.50	39.3	17.4	9.7	9.3	6.5	17.8
05030203	Little Kanawha	Ohio River	216,832	79,155	456,483	36.30	13.25	76.42	68.7	1.7	8.8	3.0	0.6	17.2
05030204	Hocking	Ohio River	120,410	47,007	222,553	38.67	15.10	71.48	32.7	19.1	12.5	17.6	8.6	9.6
05040001	Tuscarawas	Ohio River	611,862	216,179	1,097,263	91.00	32.15	163.20	7.6	38.4	9.9	12.2	25.8	6.1
05040002	Mohican	Ohio River	208,312	78,361	372,511	80.82	30.40	144.53	8.5	17.6	7.8	26.8	32.3	7.0
05040003	Walhonding	Ohio River	287,930	111,205	488,861	89.60	34.61	152.13	8.1	12.1	5.9	21.2	43.8	8.9
05040004	Muskingum	Ohio River	220,568	82,123	396,626	54.03	20.12	97.16	27.3	8.9	8.6	20.2	18.6	16.4
05040005	Wills	Ohio River	87,714	41,970	146,027	39.74	19.01	66.15	34.0	6.5	10.4	11.7	15.4	22.0
05040006	Licking	Ohio River	128,868	43,943	224,878	63.81	21.76	111.36	10.6	13.5	9.4	31.0	31.0	4.5
05050001	Upper New	Ohio River	287,506	105,661	536,811	37.61	13.82	70.22	21.0	7.3	10.5	18.2	13.2	29.8
05050002	Middle New	Ohio River	109,822	41,972	205,902	26.11	9.98	48.96	39.6	6.7	14.9	8.9	6.1	23.8
05050003	Greenbrier	Ohio River	100,896	36,350	159,826	23.44	8.44	37.12	45.2	4.0	11.7	6.5	11.0	21.6
05050004	Lower New	Ohio River	42,439	13,035	89,582	23.58	7.24	49.77	38.9	30.4	24.0	2.2	0.2	4.3
05050005	Gauley	Ohio River	60,182	23,873	115,309	16.18	6.42	30.99	69.1	5.2	15.3	1.9	2.2	6.3

05050006 Upper Kanawha	Ohio River	44,992	18,957	88,988	33.43	14.09	66.13	26.7	58.5	13.2	0.2	0.0	1.3
05050007 Elk	Ohio River	80,341	34,736	137,423	20.32	8.79	34.77	74.4	4.1	14.7	0.9	0.1	5.9
05050008 Lower Kanawha	Ohio River	125,011	44,393	238,628	56.10	19.92	107.08	41.8	28.8	11.1	4.2	2.2	11.9
05050009 Coal	Ohio River	28,933	10,634	58,012	12.58	4.62	25.22	72.0	4.7	20.3	0.7	0.0	2.2
05060001 Upper Scioto	Ohio River	868,584	351,019	1,704,808	103.00	41.63	202.17	2.2	42.4	9.9	31.2	12.1	2.2
05060002 Lower Scioto	Ohio River	241,220	109,576	527,589	43.01	19.54	94.07	20.1	18.0	7.5	38.3	9.6	6.5
05060003 Paint	Ohio River	176,663	65,550	327,173	59.76	22.17	110.67	6.2	32.0	5.3	44.0	7.0	5.4
05070101 Upper Guyandotte	Ohio River	33,995	15,186	65,174	14.01	6.26	26.85	67.4	8.7	22.9	0.3	0.0	0.8
05070102 Lower Guyandotte	Ohio River	59,726	21,977	117,494	29.42	10.83	57.88	60.6	13.5	17.6	2.2	0.2	5.9
05070201 Tug	Ohio River	62,656	24,154	111,680	15.54	5.99	27.70	68.0	7.9	21.7	0.4	0.3	1.6
05070202 Upper Levisa	Ohio River	45,856	16,889	90,773	14.60	5.38	28.90	66.6	5.5	23.0	1.3	0.4	3.1
05070203 Lower Levisa	Ohio River	43,534	16,288	80,188	15.11	5.65	27.83	53.0	17.7	25.0	1.9	0.2	2.2
05070204 Big Sandy	Ohio River	31,670	12,720	59,912	29.73	11.94	56.24	54.9	16.7	13.7	4.3	1.5	8.7
05080001 Upper Great Miami	Ohio River	526,515	193,600	970,639	82.55	30.35	152.19	1.1	27.8	7.9	25.3	35.9	2.0
05080002 Lower Great Miami	Ohio River	572,881	235,803	1,142,796	161.17	66.34	321.51	2.3	60.4	8.2	16.1	10.7	2.3
05080003 Whitewater	Ohio River	257,584	87,769	513,779	67.11	22.87	133.86	10.0	10.9	6.4	38.1	26.5	8.1
05090101 Raccoon-Symmes	Ohio River	159,914	70,764	294,769	40.99	18.14	75.56	38.5	16.9	11.4	10.4	7.4	15.5
05090102 Twelvepole	Ohio River	24,671	9,501	42,802	21.76	8.38	37.76	74.3	1.8	16.2	1.4	0.4	5.9
05090103 Little Scioto-Tygarts	Ohio River	123,355	50,414	261,934	47.39	19.37	100.62	27.1	29.8	13.4	12.9	6.9	9.9
05090104 Little Sandy	Ohio River	77,494	34,385	142,941	41.52	18.42	76.59	43.8	11.4	9.8	13.1	3.9	18.1
05090201 Ohio Brush-Whiteoak	Ohio River	390,292	137,780	706,743	71.91	25.39	130.22	21.3	10.7	4.8	35.9	10.7	16.6
05090202 Little Miami	Ohio River	478,419	180,256	1,066,353	104.38	39.33	232.65	5.8	49.2	9.5	26.9	4.9	3.8
05090203 Middle Ohio-Laughery	Ohio River	516,933	171,372	993,507	141.95	47.06	272.81	11.4	55.7	8.1	11.0	6.0	7.8
05100101 Licking	Ohio River	541,015	223,770	1,084,983	75.16	31.09	150.73	23.5	2.7	4.9	21.6	13.9	33.4
05100102 South Fork Licking	Ohio River	298,331	103,901	623,969	123.98	43.18	259.31	7.3	8.2	3.4	25.0	12.7	43.4
05100201 North Fork Kentucky	Ohio River	134,417	54,591	243,983	38.94	15.81	70.68	33.0	54.3	10.1	1.1	0.2	1.2
05100202 Middle Fork Kentucky	Ohio River	25,499	10,628	52,497	17.55	7.31	36.13	78.5	1.9	16.6	1.7	0.3	1.0
05100203 South Fork Kentucky	Ohio River	40,955	16,550	67,261	21.14	8.54	34.72	65.7	4.5	16.4	6.6	1.1	5.7
05100204 Upper Kentucky	Ohio River	115,431	42,158	225,544	41.11	15.01	80.33	38.4	3.0	9.3	16.8	5.7	26.8
05100205 Lower Kentucky	Ohio River	985,263	357,124	2,335,273	117.70	42.66	278.98	11.8	21.5	4.6	20.6	9.9	31.7
05110001 Upper Green	Ohio River	646,809	295,571	1,194,380	79.59	36.37	146.96	13.2	4.4	3.7	30.0	17.9	30.8
05110002 Barren	Ohio River	601,082	194,467	1,058,845	102.75	33.24	181.00	8.1	5.5	4.1	29.6	19.1	33.6
05110003 Middle Green	Ohio River	171,909	62,899	321,788	64.72	23.68	121.15	18.5	8.2	4.4	27.8	27.3	13.8
05110004 Rough	Ohio River	196,282	71,323	341,945	69.79	25.36	121.59	17.3	1.8	3.3	27.5	26.7	23.4
05110005 Lower Green	Ohio River	284,153	101,167	558,770	121.36	43.21	238.64	7.0	2.4	3.2	42.4	31.3	13.7
05110006 Pond	Ohio River	180,314	74,715	451,698	87.91	36.43	220.22	16.4	2.3	3.1	31.9	36.3	10.0
05120101 Upper Wabash	Ohio River	417,608	143,698	737,729	99.20	34.14	175.25	1.3	7.0	5.7	31.4	51.9	2.8
05120102 Salamonie	Ohio River	125,106	50,307	270,245	87.80	35.30	189.65	1.9	2.2	4.2	36.0	52.9	2.9
05120103 Mississinewa	Ohio River	159,158	62,833	330,499	76.87	30.35	159.62	1.9	12.3	7.0	37.6	38.6	2.6
05120104 Eel	Ohio River	134,907	51,972	206,239	64.08	24.68	97.96	2.1	17.9	7.2	35.3	33.5	4.0
05120105 Middle Wabash-Deer	Ohio River	128,269	51,446	274,494	74.69	29.95	159.83	1.2	2.7	5.2	35.3	52.1	3.5
05120106 Tippecanoe	Ohio River	190,403	71,915	413,057	36.94	13.95	80.14	2.1	15.3	10.8	39.9	29.4	2.5
05120107 Wildcat	Ohio River	207,617	68,785	381,628	95.67	31.70	175.85	1.0	11.4	6.5	34.8	44.7	1.7
05120108 Middle Wabash-Little Vermilion	Ohio River	372,225	139,954	655,187	63.59	23.91	111.93	3.9	29.9	6.7	43.5	13.0	2.9
05120109 Vermilion	Ohio River	297,145	117,257	657,843	80.48	31.76	178.17	1.8	12.4	7.2	69.9	6.8	1.9
05120110 Sugar	Ohio River	148,382	56,771	312,029	72.70	27.82	152.88	2.9	4.6	6.2	46.1	36.8	3.4
05120111 Middle Wabash-Busseron	Ohio River	348,563	111,188	599,981	66.57	21.24	114.59	7.0	15.5	7.6	55.1	12.7	2.1
05120112 Embarras	Ohio River	676,081	212,096	1,405,133	105.94	33.24	220.19	4.0	2.5	4.4	59.7	26.7	2.7
05120113 Lower Wabash	Ohio River	295,531	102,123	588,331	83.71	28.93	166.64	4.5	2.5	5.2	65.4	19.5	3.0
05120114 Little Wabash	Ohio River	818,755	295,718	1,560,285	148.44	53.61	282.88	4.5	2.0	3.3	53.0	30.8	6.6
05120115 Skillet	Ohio River	269,104	114,284	544,266	97.25	41.30	196.70	6.8	0.5	3.9	63.1	17.9	7.8
05120201 Upper White	Ohio River	801,844	336,639	1,748,726	113.09	47.48	246.64	2.2	57.3	12.0	19.8	6.8	1.9
05120202 Lower White	Ohio River	297,078	108,736	619,593	70.93	25.96	147.94	11.0	9.3	4.6	33.2	37.1	4.9

05120203	Eel	Ohio River	190,468	76,353	335,184	60.92	24.42	107.20	10.3	5.4	5.5	50.6	21.2	6.9
05120204	Driftwood	Ohio River	197,930	79,257	400,375	65.07	26.06	131.62	3.3	19.1	10.2	40.3	21.7	5.3
05120205	Flatrock-Haw	Ohio River	83,242	29,370	167,568	60.96	21.51	122.71	1.7	4.0	7.3	48.1	33.9	5.0
05120206	Upper East Fork White	Ohio River	207,178	83,793	328,062	91.46	36.99	144.82	4.8	10.9	5.1	37.6	37.1	4.6
05120207	Muscatatuck	Ohio River	227,511	96,937	411,865	76.90	32.76	139.21	17.2	4.5	4.1	43.8	22.7	7.7
05120208	Lower East Fork White	Ohio River	306,634	117,218	545,420	58.11	22.21	103.36	18.9	8.3	5.2	22.2	35.8	9.6
05120209	Patoka	Ohio River	218,768	87,252	403,161	97.79	39.00	180.21	8.1	7.9	3.3	22.9	55.1	2.8
05130101	Upper Cumberland	Cumberland	163,702	65,929	283,418	27.12	10.92	46.96	39.7	25.3	16.0	6.6	2.2	10.2
05130102	Rockcastle	Cumberland	68,115	24,124	110,762	34.45	12.20	56.02	37.5	1.7	12.0	15.8	7.6	25.4
05130103	Upper Cumberland-Lake Cumberland	Cumberland	271,347	89,943	552,657	55.72	18.47	113.48	19.7	2.3	5.5	25.7	16.4	30.4
05130104	South Fork Cumberland	Cumberland	81,548	30,410	179,990	22.74	8.48	50.19	47.1	2.7	14.5	6.9	15.7	13.1
05130105	Obey	Cumberland	99,087	35,849	172,433	40.37	14.61	70.26	20.9	15.6	6.1	11.8	16.1	29.5
05130106	Upper Cumberland-Cordell Hull	Cumberland	86,665	32,548	161,319	41.91	15.74	78.01	28.4	2.8	9.8	15.3	9.6	34.0
05130107	Collins	Cumberland	148,074	56,506	277,200	72.28	27.58	135.32	11.9	2.4	4.7	29.4	21.8	29.8
05130108	Caney	Cumberland	212,911	74,834	439,634	45.96	16.15	94.90	20.8	7.6	8.3	19.7	12.1	31.5
05130201	Lower Cumberland-Old Hickory Lake	Cumberland	184,414	75,363	346,550	71.98	29.41	135.26	17.6	6.3	7.1	17.1	11.9	40.0
05130202	Lower Cumberland-Sycamore	Cumberland	340,172	135,295	627,960	202.29	80.46	373.43	5.0	81.4	7.9	2.0	0.8	2.9
05130203	Stones	Cumberland	335,841	132,419	591,354	140.15	55.26	246.78	7.7	64.2	5.4	5.3	4.9	12.6
05130204	Harpeth	Cumberland	125,145	45,315	214,386	55.78	20.20	95.55	20.5	22.3	10.2	12.1	8.7	26.0
05130205	Lower Cumberland	Cumberland	342,936	131,316	755,232	56.55	21.65	124.53	20.6	7.2	4.5	36.2	10.9	20.5
05130206	Red	Cumberland	404,980	159,165	744,544	107.61	42.29	197.84	6.2	8.4	4.8	45.7	15.9	19.0
05140101	Silver-Little Kentucky	Ohio River	420,991	171,372	845,181	128.27	52.21	257.51	11.5	54.9	7.7	12.2	4.3	9.4
05140102	Salt	Ohio River	429,487	170,027	750,343	111.40	44.10	194.62	12.7	19.2	6.1	23.1	16.1	22.8
05140103	Rolling Fork	Ohio River	411,974	170,696	849,846	109.81	45.50	226.53	17.0	2.8	2.3	27.1	19.0	31.8
05140104	Blue-Sinking	Ohio River	310,745	119,967	541,275	63.65	24.57	110.86	19.3	5.5	4.5	29.6	20.1	21.0
05140201	Lower Ohio-Little Pigeon	Ohio River	299,903	105,650	616,061	81.99	28.88	168.43	12.3	24.5	4.5	29.3	21.1	8.4
05140202	Highland-Pigeon	Ohio River	262,802	106,918	518,489	101.40	41.25	200.05	4.5	36.8	6.9	39.2	7.8	4.8
05140203	Lower Ohio-Bay	Ohio River	131,801	49,818	242,353	46.99	17.76	86.40	27.9	2.5	4.8	35.6	10.2	18.8
05140204	Saline	Ohio River	224,652	80,098	357,938	74.15	26.44	118.14	12.2	3.4	5.6	58.5	13.3	7.0
05140205	Tradewater	Ohio River	180,927	75,488	348,008	74.24	30.98	142.80	16.0	13.7	2.8	31.1	20.0	16.4
05140206	Lower Ohio	Lower Ohio	186,928	78,601	408,302	70.41	29.61	153.80	11.5	11.6	6.8	38.0	18.3	13.9
07010101	Mississippi Headwaters	Upper Miss. R	15,646	5,988	29,678	3.30	1.26	6.27	75.4	1.7	11.2	3.0	4.1	4.5
07010102	Leech Lake	Upper Miss. R	7,222	2,303	11,930	2.21	0.71	3.66	81.9	0.4	6.6	3.3	4.5	3.3
07010103	Prairie-Willow	Upper Miss. R	78,276	28,459	143,476	13.20	4.80	24.20	59.1	22.4	9.5	2.2	3.9	2.9
07010104	Elk-Nokasippi	Upper Miss. R	105,815	40,033	205,482	22.71	8.59	44.10	23.1	16.8	10.4	10.5	33.8	5.4
07010105	Pine	Upper Miss. R	6,044	2,410	9,477	3.04	1.21	4.77	75.8	1.9	10.3	2.4	5.6	4.0
07010106	Crow Wing	Upper Miss. R	53,850	19,245	117,859	10.72	3.83	23.45	32.2	9.9	13.8	15.3	22.7	6.2
07010107	Redeye	Upper Miss. R	39,843	14,272	74,786	19.11	6.84	35.87	16.7	6.7	9.6	25.3	37.5	4.1
07010108	Long Prairie	Upper Miss. R	51,144	19,707	93,827	20.35	7.84	37.33	10.7	14.4	9.0	17.0	40.1	8.8
07010201	Platte-Spunk	Upper Miss. R	119,016	42,980	227,894	46.44	16.77	88.93	4.1	19.4	5.1	15.3	51.6	4.5
07010202	Sauk	Upper Miss. R	145,427	49,519	261,840	56.77	19.33	102.22	1.7	9.5	5.4	20.3	58.0	5.1
07010203	Clearwater-Elk	Upper Miss. R	113,601	43,580	184,069	39.72	15.24	64.37	3.7	44.9	9.6	15.3	23.8	2.6
07010204	Crow	Upper Miss. R	196,098	67,305	387,926	51.18	17.57	101.25	3.0	23.7	5.6	26.6	38.5	2.6
07010205	South Fork Crow	Upper Miss. R	335,714	136,739	561,096	102.15	41.60	170.72	1.3	27.1	3.2	32.0	34.4	2.0
07010206	Twin Cities	Upper Miss. R	1,199,174	413,455	2,298,260	513.69	177.11	984.51	0.4	92.9	5.6	0.7	0.5	0.1
07010207	Rum	Upper Miss. R	99,998	37,645	208,606	24.01	9.04	50.08	20.5	15.5	12.2	17.3	28.4	6.2
07020001	Upper Minnesota	Upper Miss. R	82,157	29,500	151,908	15.27	5.48	28.24	4.3	1.0	4.9	48.8	29.3	11.7
07020002	Pomme De Terre	Upper Miss. R	136,929	53,913	258,472	57.60	22.68	108.73	2.3	3.4	4.3	41.1	45.1	3.8
07020003	Lac Qui Parle	Upper Miss. R	199,488	74,203	368,656	68.55	25.50	126.68	1.9	27.3	2.9	32.2	27.5	8.2
07020004	Hawk-Yellow Medicine	Upper Miss. R	402,079	158,017	753,977	77.84	30.59	145.97	1.3	7.2	3.9	37.4	47.0	3.2
07020005	Chippewa	Upper Miss. R	226,300	84,769	437,344	42.05	15.75	81.27	2.8	2.9	5.6	45.6	39.5	3.7
07020006	Redwood	Upper Miss. R	196,418	84,781	322,906	104.05	44.91	171.06	0.7	25.3	3.1	26.0	40.6	4.4
07020007	Middle Minnesota	Upper Miss. R	307,052	105,224	592,801	84.62	29.00	163.37	1.3	18.7	4.2	26.4	47.8	1.7

07020008	Cottonwood	Upper Miss. R	264,597	114,319	558,104	81.68	35.29	172.29	0.7	4.5	3.5	34.4	53.1	3.7
07020009	Blue Earth	Upper Miss. R	501,353	206,834	959,148	127.15	52.46	243.26	0.4	12.3	2.8	27.1	55.2	2.0
07020010	Watonwan	Upper Miss. R	221,336	87,036	464,275	96.55	37.97	202.53	0.4	28.0	2.9	22.6	41.6	4.5
07020011	Le Sueur	Upper Miss. R	352,508	119,203	698,500	121.79	41.18	241.32	0.7	3.0	2.8	32.4	55.9	5.2
07020012	Lower Minnesota	Upper Miss. R	382,254	149,346	757,109	75.42	29.47	149.37	2.2	40.9	10.4	19.9	24.6	2.0
07030001	Upper St. Croix	Upper Miss. R	62,747	22,064	118,555	11.74	4.13	22.18	60.6	1.9	13.9	5.7	12.9	5.0
07030002	Namekagon	Upper Miss. R	22,601	9,306	43,647	8.53	3.51	16.48	65.8	0.0	21.1	2.9	7.0	3.3
07030003	Kettle	Upper Miss. R	52,428	18,531	109,139	19.02	6.72	39.59	47.6	13.2	9.9	6.8	15.9	6.4
07030004	Snake	Upper Miss. R	64,144	20,902	118,802	25.12	8.19	46.53	31.7	6.8	8.2	18.2	23.6	11.6
07030005	Lower St. Croix	Upper Miss. R	179,671	65,985	334,749	26.89	9.87	50.09	12.4	18.7	13.9	17.6	30.5	6.9
07040001	Rush-Vermillion	Upper Miss. R	190,644	75,523	332,851	65.89	26.10	115.05	4.6	37.4	7.8	16.7	28.2	5.4
07040002	Cannon	Upper Miss. R	262,804	100,150	522,782	68.73	26.19	136.71	2.0	22.7	5.8	26.6	39.0	3.9
07040003	Buffalo-Whitewater	Upper Miss. R	223,802	81,345	354,510	64.66	23.50	102.42	7.7	20.8	4.6	17.0	43.2	6.6
07040004	Zumbro	Upper Miss. R	317,261	107,354	579,551	85.08	28.79	155.41	2.1	11.1	5.2	29.2	47.0	5.4
07040005	Trempealeau	Upper Miss. R	85,158	27,994	138,002	44.87	14.75	72.72	10.4	12.6	6.2	12.4	51.3	7.1
07040006	La Crosse-Pine	Upper Miss. R	78,317	30,149	145,733	46.01	17.71	85.62	12.6	19.9	11.6	11.8	36.9	7.3
07040007	Black	Upper Miss. R	227,231	93,520	427,647	38.81	15.97	73.03	15.3	4.6	7.0	12.3	52.5	8.3
07040008	Root	Upper Miss. R	383,669	143,604	696,435	88.46	33.11	160.58	4.1	21.0	3.2	24.6	39.3	7.8
07050001	Upper Chippewa	Upper Miss. R	73,587	31,451	143,400	14.71	6.29	28.66	64.8	0.6	10.7	3.5	16.4	4.0
07050002	Flambeau	Upper Miss. R	42,915	15,079	88,134	14.20	4.99	29.15	45.0	34.7	11.0	1.2	6.3	1.8
07050003	South Fork Flambeau	Upper Miss. R	19,290	9,075	38,467	10.04	4.72	20.02	66.3	8.0	13.9	1.6	7.7	2.5
07050004	Jump	Upper Miss. R	49,216	17,555	92,376	22.60	8.06	42.42	51.0	2.6	8.3	5.3	26.2	6.6
07050005	Lower Chippewa	Upper Miss. R	163,620	59,458	243,195	30.68	11.15	45.61	15.3	12.0	10.2	13.3	42.5	6.7
07050006	Eau Claire	Upper Miss. R	70,099	26,551	132,220	29.79	11.28	56.19	16.4	10.3	9.1	11.9	45.0	7.2
07050007	Red Cedar	Upper Miss. R	140,392	50,469	244,594	28.92	10.40	50.38	11.5	13.3	8.7	13.6	48.2	4.6
07060001	Coon-Yellow	Upper Miss. R	253,992	101,104	482,259	69.03	27.48	131.06	10.2	4.1	4.2	19.2	47.8	14.5
07060002	Upper Iowa	Upper Miss. R	240,256	91,201	447,013	92.68	35.18	172.44	3.6	1.4	3.2	29.5	49.2	13.0
07060003	Grant-Little Maquoketa	Upper Miss. R	326,646	125,620	501,020	112.82	43.39	173.05	4.7	2.1	2.7	17.8	58.5	14.2
07060004	Turkey	Upper Miss. R	445,574	171,915	768,766	101.54	39.18	175.20	2.6	2.2	3.1	26.6	55.8	9.7
07060005	Apple-Plum	Upper Miss. R	345,332	146,263	675,912	90.00	38.12	176.16	5.0	7.4	4.3	30.8	38.6	13.9
07060006	Maquoketa	Upper Miss. R	465,408	162,106	937,593	95.78	33.36	192.95	1.9	1.5	3.3	24.2	56.6	12.6
07070001	Upper Wisconsin	Upper Miss. R	85,295	33,716	190,120	14.99	5.93	33.41	33.2	52.1	11.7	1.1	1.4	0.5
07070002	Lake Dubay	Upper Miss. R	329,311	135,806	576,966	46.84	19.32	82.07	14.5	28.5	6.6	9.4	35.3	5.7
07070003	Castle Rock	Upper Miss. R	716,198	284,426	1,406,013	83.72	33.25	164.36	4.0	77.6	4.3	4.8	7.7	1.5
07070004	Baraboo	Upper Miss. R	91,599	36,110	139,678	54.30	21.40	82.80	8.7	7.5	6.3	15.6	51.4	10.4
07070005	Lower Wisconsin	Upper Miss. R	255,953	96,051	473,773	42.00	15.76	77.74	15.4	4.6	7.0	15.9	46.1	11.0
07070006	Kickapoo	Upper Miss. R	107,562	41,150	206,702	53.71	20.55	103.21	16.2	3.2	5.3	13.4	48.7	13.1
07080101	Copperas-Duck	Upper Miss. R	279,258	105,273	477,630	108.32	40.83	185.27	2.6	47.2	8.1	23.0	15.2	3.8
07080102	Upper Wapsipinicon	Upper Miss. R	228,129	87,596	419,295	56.31	21.62	103.49	1.1	6.0	7.5	28.8	49.2	7.5
07080103	Lower Wapsipinicon	Upper Miss. R	169,459	65,443	321,798	66.63	25.73	126.53	1.5	2.2	6.0	39.4	41.2	9.7
07080104	Flint-Henderson	Upper Miss. R	555,712	181,509	966,224	89.89	29.36	156.29	3.8	6.5	4.8	48.5	27.5	8.9
07080105	South Skunk	Upper Miss. R	494,697	167,244	976,257	103.11	34.86	203.49	1.1	2.5	4.5	30.8	54.6	6.6
07080106	North Skunk	Upper Miss. R	233,706	81,567	420,824	103.52	36.13	186.41	1.3	1.6	3.2	32.5	49.3	12.1
07080107	Skunk	Upper Miss. R	585,199	251,686	1,328,517	136.76	58.82	310.46	3.2	1.4	2.8	29.0	52.8	10.8
07080201	Upper Cedar	Upper Miss. R	287,961	132,616	571,010	66.06	30.42	130.99	0.6	12.0	7.0	27.1	49.0	4.3
07080202	Shell Rock	Upper Miss. R	170,917	61,984	308,602	60.96	22.11	110.07	0.6	27.1	7.0	26.1	35.1	4.1
07080203	Winnebago	Upper Miss. R	104,951	40,488	204,515	59.09	22.79	115.14	0.8	7.9	8.9	38.1	40.1	4.2
07080204	West Fork Cedar	Upper Miss. R	158,392	60,852	317,232	71.54	27.49	143.29	0.4	1.7	5.4	28.0	60.6	3.8
07080205	Middle Cedar	Upper Miss. R	514,861	194,642	993,206	82.29	31.11	158.74	0.7	7.0	7.1	33.8	45.1	6.4
07080206	Lower Cedar	Upper Miss. R	211,686	83,999	412,395	75.29	29.88	146.68	2.0	10.3	6.2	36.1	36.8	8.7
07080207	Upper Iowa	Upper Miss. R	485,643	181,473	891,759	128.25	47.92	235.49	0.3	1.0	3.0	19.3	72.8	3.6
07080208	Middle Iowa	Upper Miss. R	317,145	107,610	655,179	74.12	25.15	153.12	2.2	4.9	5.2	40.5	36.0	11.1
07080209	Lower Iowa	Upper Miss. R	569,099	205,317	1,094,025	128.84	46.48	247.69	1.3	3.2	3.5	27.0	55.0	9.9

07090001	Upper Rock	Upper Miss. R	444,555	151,266	795,825	58.21	19.81	104.20	2.9	43.2	7.7	13.1	29.2	3.9
07090002	Crawfish	Upper Miss. R	107,431	38,993	196,709	52.51	19.06	96.14	3.5	7.7	6.1	22.2	52.9	7.7
07090003	Pecatonica	Upper Miss. R	511,878	195,793	875,066	105.22	40.25	179.87	2.3	6.4	3.1	33.1	45.3	9.9
07090004	Sugar	Upper Miss. R	120,489	46,793	176,116	61.87	24.03	90.43	3.5	10.3	5.7	27.5	45.1	8.0
07090005	Lower Rock	Upper Miss. R	466,726	184,065	781,186	83.48	32.92	139.72	1.9	16.9	8.7	45.8	21.8	4.9
07090006	Kishwaukee	Upper Miss. R	271,535	92,089	477,157	82.42	27.95	144.83	1.0	9.0	8.3	45.4	32.8	3.4
07090007	Green	Upper Miss. R	170,816	69,318	360,567	59.48	24.14	125.56	1.2	3.4	6.2	56.8	28.2	4.2
07100001	Des Moines Headwaters	Upper Miss. R	224,313	76,252	454,229	70.25	23.88	142.25	0.7	11.4	3.2	30.7	49.5	4.4
07100002	Upper Des Moines	Upper Miss. R	249,044	91,825	467,501	87.94	32.42	165.07	0.4	2.5	3.7	23.7	65.3	4.3
07100003	East Fork Des Moines	Upper Miss. R	291,690	117,629	526,775	85.44	34.46	154.30	0.3	1.3	3.6	30.7	59.8	4.2
07100004	Middle Des Moines	Upper Miss. R	219,142	74,752	408,063	49.71	16.96	92.57	2.0	5.3	9.7	41.0	38.1	4.0
07100005	Boone	Upper Miss. R	252,511	99,744	480,082	109.41	43.22	208.02	0.4	3.6	3.1	24.4	66.9	1.6
07100006	North Raccoon	Upper Miss. R	531,265	194,640	979,380	81.06	29.70	149.44	0.6	2.1	5.7	29.1	57.6	4.9
07100007	South Raccoon	Upper Miss. R	252,398	99,295	524,759	86.03	33.84	178.86	2.1	1.4	3.6	25.6	56.5	10.7
07100008	Lake Red Rock	Upper Miss. R	542,153	211,976	987,865	85.75	33.53	156.24	5.6	11.1	4.3	30.6	29.4	19.0
07100009	Lower Des Moines	Upper Miss. R	591,820	257,895	1,140,157	106.14	46.25	204.47	9.4	2.1	2.8	28.8	35.3	21.6
07110001	Bear-Wyaconda	Upper Miss. R	559,429	245,137	1,054,237	126.97	55.64	239.27	5.4	15.7	2.7	38.3	22.3	15.6
07110002	North Fabius	Upper Miss. R	212,906	77,139	469,093	89.54	32.44	197.29	9.0	0.0	2.6	41.7	21.5	25.2
07110003	South Fabius	Upper Miss. R	166,756	62,244	286,140	102.52	38.27	175.92	7.3	0.0	2.0	50.2	18.9	21.7
07110004	The Sny	Upper Miss. R	466,060	174,767	1,023,476	90.30	33.86	198.31	9.5	2.6	3.6	43.5	27.3	13.5
07110005	North Fork Salt	Upper Miss. R	284,696	112,738	568,540	123.15	48.77	245.94	5.7	0.0	2.3	40.6	28.1	23.3
07110006	South Fork Salt	Upper Miss. R	419,627	169,185	838,619	134.33	54.16	268.46	5.8	1.6	2.2	45.7	24.3	20.5
07110007	Salt	Upper Miss. R	224,574	94,873	422,405	109.40	46.22	205.78	8.8	0.0	2.2	50.7	20.8	17.5
07110008	Cuivre	Upper Miss. R	425,195	154,947	815,502	131.66	47.98	252.52	8.8	1.1	2.5	45.2	25.6	16.8
07110009	Peruque-Piasa	Upper Miss. R	163,444	62,337	307,878	94.01	35.85	177.08	10.2	25.9	13.5	33.2	11.3	5.8
07120001	Kankakee	Upper Miss. R	334,591	124,019	654,864	42.64	15.80	83.45	2.0	28.2	12.6	44.5	10.8	1.9
07120002	Iroquois	Upper Miss. R	325,678	134,193	657,508	58.82	24.24	118.75	0.6	3.6	6.6	69.5	18.2	1.5
07120003	Chicago	Upper Miss. R	536,548	192,074	1,024,839	340.83	122.01	651.01	0.3	89.3	9.8	0.5	0.1	0.0
07120004	Des Plaines	Upper Miss. R	1,332,596	496,454	2,309,855	341.19	127.11	591.41	0.9	83.7	10.6	3.6	0.9	0.2
07120005	Upper Illinois	Upper Miss. R	192,935	92,137	429,357	75.30	35.96	167.58	1.5	7.5	6.4	68.3	14.5	1.8
07120006	Upper Fox	Upper Miss. R	249,232	104,578	441,349	63.39	26.60	112.26	3.1	58.7	19.7	10.3	7.0	1.2
07120007	Lower Fox	Upper Miss. R	258,345	96,286	496,090	90.39	33.69	173.57	0.9	31.7	12.0	35.3	18.4	1.7
07130001	Lower Illinois-Senachwine Lake	Upper Miss. R	266,483	105,680	557,779	52.11	20.66	109.06	3.1	16.2	8.0	57.8	11.9	3.0
07130002	Vermilion	Upper Miss. R	297,021	116,420	704,713	86.23	33.80	204.59	0.5	4.6	4.8	66.2	22.4	1.4
07130003	Lower Illinois-Lake Chautauqua	Upper Miss. R	289,840	122,525	588,298	74.29	31.40	150.78	7.3	22.3	8.2	44.9	10.8	6.5
07130004	Mackinaw	Upper Miss. R	313,497	114,948	508,170	105.00	38.50	170.20	1.3	2.8	4.6	64.0	23.9	3.3
07130005	Spoon	Upper Miss. R	429,236	163,576	871,861	88.62	33.77	180.01	4.5	3.6	4.0	55.4	23.0	9.5
07130006	Upper Sangamon	Upper Miss. R	358,682	153,294	733,521	95.45	40.79	195.20	0.9	25.8	6.0	60.1	5.8	1.3
07130007	South Fork Sangamon	Upper Miss. R	243,235	84,420	372,376	81.37	28.24	124.57	1.4	10.2	5.5	68.2	11.5	3.2
07130008	Lower Sangamon	Upper Miss. R	171,866	66,537	330,344	70.05	27.12	134.64	3.7	14.2	7.4	53.6	17.8	3.4
07130009	Salt	Upper Miss. R	422,124	181,796	799,721	86.63	37.31	164.12	0.9	7.7	5.4	65.4	17.9	2.7
07130010	La Moine	Upper Miss. R	289,076	117,447	535,423	79.69	32.38	147.61	7.1	2.7	3.8	54.6	20.5	11.2
07130011	Lower Illinois	Upper Miss. R	545,077	221,199	991,279	91.71	37.22	166.78	7.6	7.3	4.0	46.7	26.6	7.7
07130012	Macoupin	Upper Miss. R	278,338	97,622	474,839	112.73	39.54	192.32	5.1	1.8	3.5	55.4	25.9	8.2
07140101	Cahokia-Joachim	Mississippi R	1,825,273	704,559	3,356,550	425.40	164.20	782.27	2.2	86.0	3.5	5.0	2.2	1.1
07140102	Meramec	Meramec	255,734	88,770	444,578	45.89	15.93	79.78	29.1	24.5	10.7	11.6	7.5	16.5
07140103	Bourbeuse	Meramec	116,953	44,046	265,969	53.72	20.23	122.16	20.1	2.0	7.0	21.1	21.0	28.8
07140104	Big	Meramec	100,894	33,626	200,016	40.13	13.37	79.55	42.3	2.1	9.1	9.3	14.6	22.6
07140105	Upper Mississippi-Cape Girardeau	Mississippi R	238,850	76,559	407,974	55.13	17.67	94.17	16.7	5.5	6.9	36.1	16.1	18.6
07140106	Big Muddy	Big Muddy	463,803	189,845	809,437	74.11	30.33	129.33	11.1	7.0	6.3	49.2	16.1	10.4
07140107	Whitewater	Whitewater	165,862	62,455	339,027	49.69	18.71	101.56	21.3	4.8	5.5	29.3	16.3	22.7
07140108	Cache	Cashe	43,389	16,160	100,547	48.78	18.17	113.03	19.6	2.3	8.0	49.5	7.6	13.0
07140201	Upper Kaskaskia	Kaskaskia	277,113	95,240	524,683	68.43	23.52	129.57	3.9	5.6	5.4	66.4	14.4	4.3

07140202	Middle Kaskaskia	Kaskaskia	569,103	234,337	1,102,134	128.57	52.94	248.98	5.8	2.4	3.5	50.5	30.7	7.1
07140203	Shoal	Kaskaskia	317,054	145,111	640,800	133.12	60.93	269.05	5.1	3.6	3.4	49.7	32.1	6.2
07140204	Lower Kaskaskia	Kaskaskia	517,581	219,611	1,107,224	125.06	53.06	267.54	4.3	9.2	5.0	49.7	25.6	6.1
09010001	Upper Souris	Souris River	173,579	60,232	319,070	30.50	10.58	56.07	4.0	3.8	8.8	68.6	1.1	13.7
09010002	Des Lacs	Souris River	19,692	7,717	34,644	7.53	2.95	13.25	2.3	0.0	7.8	71.8	2.3	15.7
09010003	Lower Souris	Souris River	47,377	15,238	91,401	8.03	2.58	15.49	5.9	0.0	10.0	67.3	0.6	16.2
09010004	Willow	Souris River	31,510	13,955	60,394	6.52	2.89	12.49	9.8	0.0	15.2	53.0	2.2	19.8
09010005	Deep	Souris River	19,842	5,128	43,783	4.33	1.12	9.56	3.0	0.6	12.3	63.5	1.1	19.6
09020101	Bois De Sioux	Red River	64,675	23,021	119,006	31.96	11.38	58.81	2.7	0.7	4.6	69.2	17.1	5.7
09020102	Mustinka	Red River	155,264	65,544	276,860	57.55	24.29	102.62	1.9	1.3	3.9	68.5	21.5	2.9
09020103	Otter Tail	Red River	117,885	46,926	262,956	21.72	8.64	48.44	6.7	10.8	8.8	37.4	31.5	4.8
09020104	Upper Red	Red River	144,332	56,154	305,564	135.89	52.87	287.70	1.6	35.6	6.1	46.2	8.7	1.8
09020105	Western Wild Rice	Red River	243,164	82,044	438,406	41.09	13.86	74.08	2.7	0.0	5.5	68.2	14.4	9.1
09020106	Buffalo	Red River	98,961	37,785	165,542	29.09	11.11	48.67	5.6	3.1	8.6	55.3	24.4	3.1
09020107	Elm-Marsh	Red River	128,149	45,088	241,707	58.50	20.58	110.34	1.2	1.3	4.2	88.3	2.7	2.3
09020108	Eastern Wild Rice	Red River	104,216	41,127	223,219	24.23	9.56	51.89	13.1	2.0	6.8	57.7	15.2	5.2
09020109	Goose	Red River	69,934	27,346	145,034	20.47	8.00	42.45	3.8	0.9	7.6	84.0	0.6	3.1
09020201	Devils Lake	Red River	9,706	3,554	20,080	1.05	0.38	2.17	13.0	0.0	10.9	65.9	1.9	8.3
09020202	Upper Sheyenne	Red River	19,171	5,961	43,093	3.71	1.15	8.33	4.5	0.0	10.2	63.2	1.7	20.5
09020203	Middle Sheyenne	Red River	50,599	19,507	91,933	9.58	3.69	17.41	6.9	0.0	10.2	64.8	1.7	16.3
09020204	Lower Sheyenne	Red River	207,980	79,606	403,315	42.89	16.42	83.17	3.2	1.7	6.7	73.5	5.3	9.6
09020205	Maple	Red River	67,666	28,192	142,284	17.40	7.25	36.58	1.7	0.0	6.0	84.5	3.3	4.5
09020301	Sandhill-Wilson	Red River	85,225	34,363	167,161	28.38	11.44	55.66	2.5	6.2	10.6	75.1	3.0	2.6
09020302	Red Lakes	Red River	2,373	750	5,346	0.50	0.16	1.12	81.1	0.1	6.0	2.3	4.8	5.7
09020303	Red Lake	Red River	91,450	32,380	179,641	28.21	9.99	55.42	11.6	19.4	10.1	48.4	6.2	4.5
09020304	Thief	Red River	14,285	5,819	27,480	5.05	2.06	9.72	23.4	0.2	9.9	54.7	7.0	4.7
09020305	Clearwater	Red River	51,313	17,661	103,002	14.85	5.11	29.80	23.0	5.9	11.6	35.8	13.6	10.0
09020306	Grand Marais-Red	Red River	53,001	20,825	108,776	43.67	17.16	89.63	1.8	5.1	7.3	76.8	4.9	4.1
09020307	Turtle	Red River	58,995	24,049	129,403	33.35	13.59	73.14	1.5	36.9	5.2	52.8	1.5	2.1
09020308	Forest	Red River	42,681	17,463	71,402	15.93	6.52	26.65	2.8	0.0	5.7	86.4	0.9	4.2
09020309	Snake	Red River	42,960	17,047	78,123	15.72	6.24	28.59	3.7	2.8	9.5	73.7	6.4	4.0
09020310	Park	Red River	83,619	30,653	152,201	27.41	10.05	49.90	2.1	1.8	5.9	86.3	0.0	3.9
09020311	Lower Red	Red River	63,646	21,925	131,609	31.94	11.00	66.05	4.3	1.1	8.7	78.2	2.9	4.8
09020312	Two Rivers	Red River	78,334	29,695	159,174	26.66	10.11	54.17	9.2	1.7	9.4	65.5	7.5	6.7
09020313	Pembina	Red River	83,515	33,281	142,936	24.06	9.59	41.19	5.1	0.0	6.0	86.1	0.0	2.8
09020314	Roseau	Rainy River	53,679	19,621	93,686	12.71	4.64	22.17	27.7	7.1	9.6	37.5	14.0	4.2
09030001	Rainy Headwaters	Rainy River	6,746	2,886	12,885	0.88	0.38	1.68	99.2	0.0	0.8	0.0	0.0	0.1
09030002	Vermilion	Rainy River	15,283	5,840	30,219	5.71	2.18	11.29	91.4	3.7	3.7	0.1	0.3	0.8
09030003	Rainy Lake	Rainy River	21,281	6,914	40,883	7.49	2.43	14.38	94.5	0.0	2.4	0.8	0.6	1.7
09030004	Upper Rainy	Rainy River	11,024	4,126	22,611	3.66	1.37	7.50	97.1	0.0	1.5	0.4	0.3	0.8
09030005	Little Fork	Rainy River	57,231	27,658	99,695	11.68	5.64	20.34	88.7	0.8	6.3	0.9	1.2	2.1
09030006	Big Fork	Rainy River	56,139	21,516	128,584	10.26	3.93	23.50	90.2	1.2	4.8	1.0	0.8	1.9
09030007	Rapid	Rainy River	30,902	11,078	60,592	11.27	4.04	22.10	92.1	0.0	2.6	2.9	0.9	1.6
09030008	Lower Rainy	Rainy River	5,452	2,146	11,007	4.36	1.71	8.79	67.0	0.0	11.3	13.3	3.4	5.0
09030009	Lake of the Woods	Rainy River	14,728	4,954	29,837	6.87	2.31	13.92	49.5	0.6	12.0	24.7	8.8	4.4

Table S5_TN. Total annual nitrogen and phosphorus loads and yields, with confidence intervals, for HUC8 watersheds of Major River Basin 3 (MRB3).
Accumulated nondelivered loads and yields from each individual HUC8 (based on sum of incremental loads), and does not include loads from upstream HUC8s.
Direct results from the SPARROW models: not corrected for any spatial biases.

Supplemental Material to Robertson and Saad, 2011, Journal of the American Water Resources Association, Nutrient Inputs to the Laurentian Great Lakes By Source and River Basin Estimated Using SPARROW Watershed Models.

The total load for each HUC8 watershed was calculated by summing the simulated incremental loads from each SPARROW reach within a HUC8 watershed.
Yield is load per unit area of the HUC8 watershed.
[%, percent; CI, Confidence interval; kg, kilogram; ha, hectare]

HUC8's are based on Seaber, P.R., F.P. Kapinos, and G.L. Knapp, 1987. Hydrologic Unit maps: U.S. Geol. Surv. Water-Supply Paper 2294, 63 p. Available online at: http://water.usgs.gov/GIS/huc.html

									Percent Contribution by Source				
HUC8	HUC8 NAME	Great Lake/Basin	Total Nitrogen Load (kg)	Lower 95% CI Total Nitrogen Load (kg)	Upper 95% CI Total Nitrogen Load (kg)	Total Nitrogen Yield (kg/km^2)	Lower 95% CI Total Nitrogen Yield (kg/km^2)	Upper 95% CI Total Nitrogen Yield (kg/km^2)	Atmosphere	Point sources	Additional agricultural sources	Farm fertilizers	Manure (confined)
04010101	Baptism-Brule	Lake Superior	700,720	309,197	1,248,652	119.94	52.92	213.73	98.3	1.4	0.3	0.0	0.0
04010102	Beaver-Lester	Lake Superior	988,551	487,648	1,806,756	578.45	285.35	1,057.23	33.1	66.0	0.1	0.3	0.4
04010201	St. Louis	Lake Superior	1,361,701	734,263	2,210,806	183.42	98.91	297.80	84.0	10.0	1.1	1.8	3.1
04010202	Cloquet	Lake Superior	158,148	78,606	320,659	79.67	39.60	161.54	97.8	0.0	0.4	0.7	1.1
04010301	Beartrap-Nemadji	Lake Superior	1,863,229	915,066	3,164,297	385.19	189.17	654.17	72.3	16.1	1.7	2.0	7.9
04010302	Bad-Montreal	Lake Superior	895,149	404,910	1,747,682	268.12	121.28	523.47	81.3	5.1	4.6	2.2	6.8
04020101	Black-Presque Isle	Lake Superior	552,804	270,361	890,174	209.80	102.61	337.84	91.8	6.1	1.8	0.1	0.2
04020102	Ontonagon	Lake Superior	677,576	280,177	1,071,461	187.17	77.39	295.98	86.7	0.9	8.9	0.7	2.8
04020103	Keweenaw Peninsula	Lake Superior	524,460	245,489	793,161	183.03	85.67	276.81	83.3	11.6	3.1	0.7	1.3
04020104	Sturgeon	Lake Superior	367,030	179,834	567,178	198.58	97.30	306.86	87.8	0.0	7.7	1.5	3.0
04020105	Dead-Kelsey	Lake Superior	437,294	245,208	703,593	184.38	103.39	296.66	81.4	16.8	1.2	0.2	0.4
04020201	Betsy-Chocolay	Lake Superior	549,281	279,169	1,015,276	183.55	93.29	339.27	92.4	5.6	0.7	0.5	0.8
04020202	Tahquamenon	Lake Superior	404,031	226,119	747,649	189.00	105.78	349.74	94.9	3.7	0.5	0.6	0.3
04020203	Waiska	Lake Superior	225,128	104,767	396,769	286.28	133.23	504.55	74.6	5.5	14.8	1.0	4.2
04030101	Manitowoc-Sheboygan	Lake Michigan	6,820,879	3,503,702	12,295,333	1,598.87	821.30	2,882.14	18.6	9.1	14.3	16.6	41.5
04030102	Door-Kewaunee	Lake Michigan	2,384,751	1,164,183	4,242,311	1,205.33	588.42	2,144.21	20.4	22.1	7.0	15.5	34.9
04030103	Duck-Pensaukee	Lake Michigan	1,156,920	529,154	1,944,539	896.24	409.92	1,506.39	21.4	1.7	18.0	18.5	40.4
04030104	Oconto	Lake Michigan	1,223,624	603,757	1,918,827	453.12	223.58	710.56	43.4	3.6	14.0	14.0	25.0
04030105	Peshtigo	Lake Michigan	900,597	485,684	1,719,912	301.24	162.46	575.29	52.2	4.4	13.8	10.6	19.0
04030106	Brule	Lake Michigan	506,234	254,559	803,068	186.14	93.60	295.28	85.5	3.5	6.2	2.9	1.8
04030107	Michigamme	Lake Michigan	188,878	89,043	307,510	103.12	48.61	167.89	97.0	1.4	0.9	0.4	0.2
04030108	Menominee	Lake Michigan	1,512,318	705,524	2,554,776	253.17	118.11	427.69	65.8	11.8	8.2	5.0	9.2
04030109	Cedar-Ford	Lake Michigan	673,234	272,064	971,717	253.17	102.31	365.41	72.6	3.5	7.9	5.7	10.3
04030110	Escanaba	Lake Michigan	463,985	258,981	789,513	194.23	108.41	330.50	83.1	5.4	4.7	3.0	3.8
04030111	Tacoosh-Whitefish	Lake Michigan	360,218	154,368	527,475	216.67	92.85	317.28	84.3	6.1	4.3	2.4	3.0
04030112	Fishdam-Sturgeon	Lake Michigan	335,007	149,231	591,677	222.78	99.24	393.47	82.0	0.0	8.5	4.7	4.8
04030201	Upper Fox	Lake Michigan	2,911,491	1,700,298	5,262,546	672.28	392.61	1,215.15	26.2	8.7	21.4	24.3	19.3
04030202	Wolf	Lake Michigan	5,052,305	2,732,733	7,481,839	525.48	284.23	778.17	29.2	3.5	17.7	20.0	29.7
04030203	Lake Winnebago	Lake Michigan	501,293	235,687	891,363	376.17	176.86	668.88	24.0	10.3	15.8	18.7	31.2
04030204	Lower Fox	Lake Michigan	2,775,680	1,590,514	5,132,524	2,350.88	1,347.09	4,347.02	10.6	47.6	8.2	9.4	24.1
04040001	Little Calumet-Galien	Lake Michigan	2,784,993	1,678,699	4,579,040	1,519.84	916.11	2,498.89	22.9	49.2	9.7	16.1	2.1
04040002	Pike-Root	Lake Michigan	4,508,049	2,244,893	7,820,173	4,277.61	2,130.14	7,420.43	9.4	77.6	5.2	5.4	2.3
04040003	Milwaukee	Lake Michigan	2,295,809	1,033,554	3,979,893	1,015.96	457.38	1,761.22	25.4	18.9	14.5	15.1	26.0

04050001	St. Joseph	Lake Michigan	15,088,761	7,038,707	24,137,831	1,227.22	572.48	1,963.20	24.3	9.3	19.0	31.2	16.2
04050002	Black-Macatawa	Lake Michigan	1,797,611	886,474	2,889,680	1,221.06	602.15	1,962.87	23.8	15.3	13.9	20.0	27.0
04050003	Kalamazoo	Lake Michigan	5,739,352	2,735,719	9,265,705	1,093.41	521.19	1,765.23	25.3	17.1	13.8	22.6	21.3
04050004	Upper Grand	Lake Michigan	4,211,819	2,315,294	6,909,532	927.62	509.92	1,521.77	28.0	21.3	15.6	23.9	11.2
04050005	Maple	Lake Michigan	3,804,576	1,886,363	6,430,752	1,543.92	765.50	2,609.64	22.6	1.9	21.0	35.5	18.9
04050006	Lower Grand	Lake Michigan	6,105,806	3,202,811	10,349,679	1,228.04	644.17	2,081.59	23.7	19.7	12.9	22.6	21.0
04050007	Thornapple	Lake Michigan	2,144,735	1,192,891	3,690,093	962.47	535.32	1,655.96	31.7	3.2	19.8	30.1	15.3
04060101	Pere Marquette-White	Lake Michigan	2,889,841	1,269,227	4,234,558	516.76	226.96	757.22	44.8	16.3	12.4	16.6	9.9
04060102	Muskegon	Lake Michigan	2,714,116	1,261,471	5,203,536	385.15	179.01	738.41	51.5	2.9	12.3	14.7	18.6
04060103	Manistee	Lake Michigan	1,545,539	721,556	2,995,434	306.30	143.00	593.64	67.3	15.3	8.7	4.9	3.8
04060104	Betsie-Platte	Lake Michigan	572,094	266,578	981,718	277.13	129.14	475.56	70.4	2.4	12.7	11.1	3.4
04060105	Boardman-Charlevoix	Lake Michigan	1,097,596	576,107	2,206,576	250.30	131.38	503.20	59.0	9.8	15.3	10.5	5.4
04060106	Manistique	Lake Michigan	575,183	255,548	981,440	152.25	67.64	259.78	91.6	5.3	1.5	0.9	0.7
04060107	Brevoort-Millecoquins	Lake Michigan	275,966	119,827	596,646	179.91	78.12	388.97	91.6	0.0	3.9	1.0	3.5
04070001	St. Marys	Lake Huron	510,910	278,947	856,347	406.43	221.90	681.22	60.8	12.0	19.1	1.4	6.6
04070002	Carp-Pine	Lake Huron	410,795	206,654	774,853	242.74	122.11	457.87	81.4	9.4	6.6	0.5	2.2
04070003	Lone Lake-Ocqueoc	Lake Huron	524,600	274,685	877,062	256.84	134.48	429.40	69.3	10.2	6.1	8.7	5.7
04070004	Cheboygan	Lake Huron	191,377	86,216	320,479	81.87	36.88	137.10	76.7	0.9	11.7	4.6	6.1
04070005	Black	Lake Huron	124,744	56,803	213,380	79.86	36.36	136.60	83.8	0.0	6.6	5.1	4.5
04070006	Thunder Bay	Lake Huron	673,503	368,302	1,145,223	203.74	111.42	346.44	65.6	6.9	7.9	9.3	10.3
04070007	Au Sable	Lake Huron	868,160	427,581	1,661,686	164.91	81.22	315.64	90.7	1.2	2.9	2.0	3.2
04080101	Au Gres-Rifle	Lake Huron	1,128,943	494,239	2,021,715	422.59	185.01	756.77	51.1	5.9	9.8	17.5	15.6
04080102	Kawkawlin-Pine	Lake Huron	1,624,239	772,693	3,113,373	1,280.11	608.98	2,453.74	29.1	0.6	23.0	42.8	4.5
04080103	Pigeon-Wiscoggin	Lake Huron	5,460,093	2,256,371	9,801,255	2,515.58	1,039.56	4,515.65	17.2	1.5	23.5	41.4	16.3
04080104	Birch-Willow	Lake Huron	2,612,370	1,430,649	4,723,372	1,791.87	981.31	3,239.85	19.0	2.3	20.7	38.1	19.9
04080201	Tittabawassee	Lake Huron	1,816,493	901,835	3,309,424	492.51	244.52	897.29	43.6	13.2	14.0	19.4	9.7
04080202	Pine	Lake Huron	2,681,004	1,297,738	3,985,737	970.88	469.95	1,443.37	31.4	4.8	20.0	29.5	14.3
04080203	Shiawassee	Lake Huron	3,361,079	1,683,145	5,287,558	1,168.45	585.13	1,838.17	28.0	4.4	23.1	36.5	8.1
04080204	Flint	Lake Huron	3,394,194	1,690,544	5,141,172	878.33	437.47	1,330.40	28.7	27.5	14.9	23.2	5.7
04080205	Cass	Lake Huron	2,864,282	1,339,000	4,466,686	1,193.07	557.74	1,860.52	26.9	2.6	20.2	38.7	11.6
04080206	Saginaw	Lake Huron	2,848,255	1,386,081	4,922,129	4,273.64	2,079.73	7,385.37	10.4	59.0	10.6	18.1	1.9
04090001	St. Clair	Lake Erie	3,407,801	1,358,946	5,572,562	1,020.10	406.79	1,668.10	29.0	11.0	21.2	28.5	10.3
04090002	Lake St. Clair	Lake Erie	253,689	123,551	487,096	509.79	248.28	978.83	56.5	42.0	0.7	0.6	0.1
04090003	Clinton	Lake Erie	1,990,381	844,137	3,110,462	774.56	328.50	1,210.45	29.0	53.8	6.3	8.8	2.0
04090004	Detroit	Lake Erie	13,919,875	6,419,893	27,913,197	5,555.17	2,562.06	11,139.66	4.5	95.0	0.1	0.3	0.1
04090005	Huron	Lake Erie	1,807,380	820,459	3,527,973	756.23	343.29	1,476.14	28.5	45.6	6.9	14.7	4.3
04100001	Ottawa-Stony	Lake Erie	2,773,681	1,470,831	4,124,611	1,655.44	877.85	2,461.73	26.6	6.8	21.1	40.2	5.3
04100002	Raisin	Lake Erie	5,437,430	2,919,945	8,067,315	1,912.51	1,027.04	2,837.53	23.9	8.5	22.1	35.6	9.8
04100003	St. Joseph	Lake Erie	5,963,017	2,661,307	10,638,990	2,121.86	946.99	3,785.74	24.9	2.2	23.2	39.4	10.3
04100004	St. Marys	Lake Erie	11,884,023	5,671,338	19,528,331	5,771.69	2,754.39	9,484.28	13.4	1.3	18.0	33.7	33.5
04100005	Upper Maumee	Lake Erie	4,334,829	2,094,288	7,129,389	4,863.00	2,349.46	7,998.06	16.4	29.8	20.8	28.0	5.1
04100006	Tiffin	Lake Erie	6,448,329	3,060,166	9,929,018	3,125.52	1,483.27	4,812.62	21.3	1.5	25.9	41.0	10.3
04100007	Auglaize	Lake Erie	23,202,188	10,253,721	44,190,524	5,183.06	2,290.55	9,871.58	18.1	3.0	25.1	44.8	9.0
04100008	Blanchard	Lake Erie	8,188,925	3,837,818	12,501,518	4,267.69	2,000.09	6,515.21	19.3	2.8	26.3	43.6	8.1
04100009	Lower Maumee	Lake Erie	11,824,611	6,733,553	19,633,256	4,333.44	2,467.69	7,195.12	16.8	14.7	21.9	41.9	4.8
04100010	Cedar-Portage	Lake Erie	10,447,629	5,276,705	17,441,996	4,183.89	2,113.12	6,984.87	20.4	2.5	27.4	46.8	2.8
04100011	Sandusky	Lake Erie	19,259,894	9,629,829	30,515,467	4,017.98	2,008.96	6,366.10	19.2	2.1	25.1	46.9	6.7
04100012	Huron-Vermilion	Lake Erie	3,833,563	1,839,649	8,207,346	1,942.87	932.35	4,159.54	19.9	5.5	24.0	42.9	7.7
04110001	Black-Rocky	Lake Erie	4,148,902	1,975,842	7,186,809	1,768.85	842.39	3,064.04	20.9	40.7	10.4	20.8	7.2

04110002	Cuyahoga	Lake Erie	4,468,976	2,268,143	6,563,351	2,137.44	1,084.82	3,139.14	13.4	78.6	2.0	4.1	1.7
04110003	Ashtabula-Chagrin	Lake Erie	3,621,720	1,565,383	5,800,159	2,247.27	971.32	3,598.98	17.3	69.3	3.4	7.5	2.6
04110004	Grand	Lake Erie	1,896,581	897,803	3,392,822	1,031.34	488.21	1,844.98	40.2	1.8	17.7	27.3	13.1
04120101	Chautauqua-Conneaut	Lake Erie	3,382,203	1,466,694	5,099,897	1,508.57	654.19	2,274.71	30.5	38.7	9.9	8.6	12.3
04120102	Cattaraugus	Lake Erie	1,415,283	652,251	2,537,783	976.47	450.02	1,750.93	42.5	3.7	14.3	10.6	28.9
04120103	Buffalo-Eighteenmile	Lake Erie	2,592,539	1,306,281	4,465,456	1,392.18	701.46	2,397.92	30.4	33.3	6.9	8.6	20.8
04120104	Niagara	Lake Ontario	6,787,416	3,212,136	11,932,202	2,687.69	1,271.95	4,724.93	12.6	71.2	3.2	4.4	8.6
04130001	Oak Orchard-Twelvemile	Lake Ontario	2,391,829	1,272,141	3,690,735	717.45	381.59	1,107.06	33.4	22.6	16.3	17.0	10.6
04130002	Upper Genesee	Lake Ontario	2,407,792	1,237,369	4,863,548	655.39	336.81	1,323.84	36.7	4.3	11.5	12.6	34.9
04130003	Lower Genesee	Lake Ontario	2,891,557	1,298,600	5,433,498	1,048.63	470.94	1,970.47	26.3	9.2	14.8	18.4	31.3
04140101	Irondequoit-Ninemile	Lake Ontario	2,813,941	1,134,424	5,266,751	1,541.78	621.56	2,885.69	18.1	65.5	3.7	6.1	6.5
04140102	Salmon-Sandy	Lake Ontario	2,335,437	1,116,846	3,836,089	941.04	450.02	1,545.71	70.7	3.2	6.0	4.4	15.8
04140201	Seneca	Lake Ontario	6,767,219	3,728,817	11,418,857	758.86	418.14	1,280.48	26.4	29.1	10.9	13.4	20.1
04140202	Oneida	Lake Ontario	2,389,674	1,374,465	4,684,693	614.68	353.54	1,205.01	55.7	9.4	7.1	7.5	20.3
04140203	Oswego	Lake Ontario	289,615	159,680	465,419	768.19	423.54	1,234.50	54.2	16.8	7.1	7.9	13.9
04150101	Black	Lake Ontario	4,014,087	1,753,892	7,231,202	809.51	353.70	1,458.30	63.0	4.4	4.0	5.1	23.5
04150102	Chaumont-Perch	Lake Ontario	1,386,664	611,705	2,253,270	1,512.64	667.28	2,457.97	50.0	2.1	6.8	6.8	34.4
04150301	Upper St. Lawrence	Lake Ontario	1,205,381	610,246	2,353,180	510.18	258.29	996.00	58.7	8.1	5.1	4.9	23.2
04150302	Oswegatchie	St.Lawrence	1,319,452	610,002	2,120,240	504.73	233.34	811.05	67.3	2.6	2.6	4.9	22.5
04150303	Indian	St.Lawrence	749,627	399,309	1,262,627	519.64	276.80	875.25	67.1	0.8	3.1	5.0	24.0
04150304	Grass	St.Lawrence	916,110	523,688	1,801,989	506.14	289.33	995.58	58.0	10.0	3.6	5.1	23.2
04150305	Raquette	St.Lawrence	864,457	480,324	1,615,614	267.66	148.72	500.23	79.4	5.7	1.6	2.4	11.0
04150306	St. Regis	St.Lawrence	771,595	423,138	1,358,314	346.01	189.75	609.11	75.4	3.8	2.8	3.3	14.7
04150307	English-Salmon	St.Lawrence	760,408	419,688	1,244,494	451.53	249.21	738.98	49.6	2.9	5.5	7.9	34.0
05010001	Upper Allegheny	Ohio River	3,528,988	1,541,738	5,732,988	529.64	231.39	860.42	72.4	7.9	4.3	4.2	11.2
05010002	Conewango	Ohio River	2,092,966	1,037,038	3,608,936	883.95	437.98	1,524.20	49.6	6.7	10.3	8.9	24.5
05010003	Middle Allegheny-Tionesta	Ohio River	2,500,964	1,178,251	4,474,868	573.17	270.03	1,025.55	76.7	4.4	5.5	5.0	8.4
05010004	French	Ohio River	2,821,004	1,454,494	5,816,571	886.30	456.97	1,827.44	51.1	3.1	13.0	13.5	19.2
05010005	Clarion	Ohio River	1,850,469	888,829	3,064,467	573.11	275.28	949.10	77.4	5.0	5.8	4.9	6.9
05010006	Middle Allegheny-Redbank	Ohio River	2,906,298	1,426,421	5,015,541	667.29	327.51	1,151.58	64.7	4.4	7.2	12.1	11.7
05010007	Conemaugh	Ohio River	2,766,902	1,338,535	4,244,206	778.79	376.75	1,194.60	56.3	9.6	4.8	14.1	15.2
05010008	Kiskiminetas	Ohio River	896,616	422,203	1,436,405	671.77	316.33	1,076.20	61.6	13.9	4.3	8.4	11.8
05010009	Lower Allegheny	Ohio River	1,019,691	574,775	1,565,568	791.81	446.32	1,215.69	42.9	38.9	6.3	6.0	5.9
05020001	Tygart Valley	Ohio River	2,119,982	1,042,194	3,761,187	596.66	293.32	1,058.56	82.5	4.8	5.2	5.3	2.2
05020002	West Fork	Ohio River	1,193,687	615,399	1,871,255	520.49	268.34	815.94	75.7	15.0	3.2	4.3	1.8
05020003	Upper Monongahela	Ohio River	802,383	376,657	1,543,430	672.34	315.61	1,293.29	57.2	34.7	3.4	2.3	2.3
05020004	Cheat	Ohio River	2,193,106	1,042,945	3,888,050	589.86	280.51	1,045.73	88.6	1.9	2.8	3.7	3.1
05020005	Lower Monongahela	Ohio River	2,474,748	1,094,204	3,934,784	648.85	286.89	1,031.66	52.6	30.8	3.6	5.0	8.0
05020006	Youghiogheny	Ohio River	3,312,912	1,711,150	5,712,489	730.92	377.53	1,260.33	55.3	7.8	4.9	12.5	19.5
05030101	Upper Ohio	Ohio River	6,568,610	3,362,411	10,208,614	1,269.01	649.60	1,972.24	23.0	61.4	3.8	5.6	6.2
05030102	Shenango	Ohio River	2,007,203	1,059,444	3,719,833	727.05	383.75	1,347.39	38.2	13.2	15.4	15.9	17.3
05030103	Mahoning	Ohio River	3,144,531	1,403,591	5,138,732	1,081.51	482.74	1,767.38	24.7	44.1	8.0	14.7	8.4
05030104	Beaver	Ohio River	225,203	114,840	432,853	718.15	366.21	1,380.32	41.6	36.3	5.1	6.3	10.6
05030105	Connoquenessing	Ohio River	4,612,417	2,117,796	8,783,811	2,126.79	976.52	4,050.23	16.7	70.7	4.2	3.9	4.5
05030106	Upper Ohio-Wheeling	Ohio River	2,332,305	1,242,264	4,304,878	594.25	316.52	1,096.85	61.2	18.3	5.1	6.1	9.2
05030201	Little Muskingum-Middle Island	Ohio River	2,703,089	1,408,023	5,615,481	578.77	301.48	1,202.36	68.6	19.1	3.0	5.2	4.1
05030202	Upper Ohio-Shade	Ohio River	4,022,889	1,831,311	6,867,983	1,113.65	506.96	1,901.25	32.2	57.0	1.8	6.0	3.0
05030203	Little Kanawha	Ohio River	2,617,430	1,375,060	3,965,352	438.19	230.20	663.85	92.1	1.4	2.3	3.6	0.6
05030204	Hocking	Ohio River	2,300,749	1,006,137	3,884,368	738.96	323.15	1,247.58	48.7	9.9	12.0	23.2	6.1

05040001	Tuscarawas	Ohio River	6,463,652	3,048,709	12,849,525	961.34	453.43	1,911.11	27.9	12.5	11.9	20.7	27.1
05040002	Mohican	Ohio River	3,275,973	1,650,561	5,317,052	1,271.07	640.42	2,063.01	22.9	13.5	15.7	27.7	20.1
05040003	Walhonding	Ohio River	3,787,188	2,043,438	5,665,537	1,178.56	635.91	1,763.10	24.9	2.8	14.4	27.0	30.9
05040004	Muskingum	Ohio River	2,710,571	1,178,574	4,323,993	664.00	288.71	1,059.23	51.0	2.9	8.5	24.5	13.0
05040005	Wills	Ohio River	1,101,397	584,042	2,036,581	498.96	264.58	922.62	59.0	5.9	10.2	13.9	11.0
05040006	Licking	Ohio River	2,429,921	1,237,823	4,682,567	1,203.26	612.95	2,318.73	29.8	8.2	18.3	28.7	14.9
05050001	Upper New	Ohio River	4,198,165	2,280,846	8,636,687	549.16	298.36	1,129.76	51.1	5.7	0.7	26.3	16.2
05050002	Middle New	Ohio River	4,088,147	1,918,692	6,874,688	972.08	456.23	1,634.66	22.6	69.2	0.4	5.0	2.8
05050003	Greenbrier	Ohio River	1,791,574	788,944	2,747,886	416.15	183.26	638.28	78.4	2.5	2.0	9.9	7.2
05050004	Lower New	Ohio River	827,786	489,337	1,177,984	459.92	271.88	654.50	75.3	20.2	0.6	3.5	0.3
05050005	Gauley	Ohio River	2,050,248	972,612	3,487,562	551.05	261.41	937.37	93.1	2.0	1.8	2.1	1.0
05050006	Upper Kanawha	Ohio River	721,538	418,643	1,275,572	536.20	311.11	947.92	61.6	37.7	0.2	0.5	0.0
05050007	Elk	Ohio River	1,935,838	824,538	3,406,674	489.73	208.59	861.82	96.0	1.8	1.1	1.0	0.1
05050008	Lower Kanawha	Ohio River	1,368,680	664,049	2,588,637	614.18	297.98	1,161.62	57.7	33.5	2.6	4.5	1.7
05050009	Coal	Ohio River	861,821	406,984	1,543,014	374.68	176.94	670.83	96.6	1.8	0.7	0.9	0.1
05060001	Upper Scioto	Ohio River	24,660,303	13,394,785	39,027,319	2,924.39	1,588.45	4,628.14	19.8	15.9	20.9	36.2	7.1
05060002	Lower Scioto	Ohio River	6,121,722	3,140,405	9,776,703	1,091.54	559.95	1,743.25	32.7	6.3	18.8	37.6	4.6
05060003	Paint	Ohio River	6,809,943	2,965,766	10,125,935	2,303.48	1,003.18	3,425.12	22.1	1.9	25.1	47.0	3.8
05070101	Upper Guyandotte	Ohio River	837,148	375,936	1,495,070	344.93	154.89	616.01	94.7	4.5	0.2	0.4	0.1
05070102	Lower Guyandotte	Ohio River	885,372	436,599	1,590,702	436.18	215.09	783.67	85.4	10.1	1.7	2.5	0.3
05070201	Tug	Ohio River	1,272,812	612,302	2,098,577	315.68	151.86	520.48	94.8	3.7	0.7	0.5	0.3
05070202	Upper Levisa	Ohio River	879,752	471,476	1,614,696	280.08	150.10	514.05	95.0	3.0	0.2	1.5	0.4
05070203	Lower Levisa	Ohio River	932,377	453,549	1,875,794	323.62	157.42	651.07	90.1	6.9	0.5	2.4	0.2
05070204	Big Sandy	Ohio River	465,834	218,323	884,281	437.26	204.93	830.04	84.1	10.4	0.4	4.0	1.1
05080001	Upper Great Miami	Ohio River	14,601,517	7,893,476	26,049,254	2,289.36	1,237.61	4,084.24	16.9	6.2	19.9	34.6	22.4
05080002	Lower Great Miami	Ohio River	8,793,246	3,718,257	13,236,699	2,473.84	1,046.07	3,723.94	18.5	27.6	15.1	28.9	10.0
05080003	Whitewater	Ohio River	7,567,155	3,976,509	11,169,785	1,971.56	1,036.05	2,910.19	23.9	3.8	21.1	40.2	11.1
05090101	Raccoon-Symmes	Ohio River	2,227,059	1,094,158	3,446,901	570.86	280.46	883.54	63.1	15.0	5.4	11.4	5.2
05090102	Twelvepole	Ohio River	420,593	218,957	688,467	371.04	193.16	607.35	96.5	0.8	0.8	1.4	0.4
05090103	Little Scioto-Tygarts	Ohio River	2,149,238	1,063,902	3,363,211	825.61	408.69	1,291.95	46.5	35.9	2.8	11.5	3.4
05090104	Little Sandy	Ohio River	967,419	432,523	1,476,548	518.36	231.76	791.17	80.8	2.2	0.8	13.4	2.8
05090201	Ohio Brush-Whiteoak	Ohio River	5,170,571	2,425,181	9,124,300	952.69	446.85	1,681.17	45.9	2.5	10.7	34.2	6.7
05090202	Little Miami	Ohio River	8,016,858	4,064,813	14,527,726	1,749.05	886.83	3,169.54	24.3	13.9	19.6	38.0	4.2
05090203	Middle Ohio-Laughery	Ohio River	7,145,697	3,634,847	11,286,198	1,962.19	998.12	3,099.16	22.4	60.6	4.5	9.8	2.8
05100101	Licking	Ohio River	5,496,385	2,724,320	9,405,312	763.56	378.46	1,306.59	62.3	2.6	2.1	21.6	11.3
05100102	South Fork Licking	Ohio River	2,617,492	1,367,332	4,460,123	1,087.78	568.24	1,853.54	47.5	4.1	2.9	32.9	12.6
05100201	North Fork Kentucky	Ohio River	1,212,726	541,515	2,058,841	351.29	156.86	596.39	92.8	4.2	0.1	2.5	0.3
05100202	Middle Fork Kentucky	Ohio River	493,218	252,899	843,469	339.42	174.04	580.46	97.3	0.4	0.3	1.7	0.3
05100203	South Fork Kentucky	Ohio River	781,869	377,936	1,322,689	403.58	195.08	682.74	89.7	2.2	0.2	7.1	0.9
05100204	Upper Kentucky	Ohio River	1,693,937	893,500	2,812,525	603.28	318.21	1,001.66	78.3	2.3	0.7	15.4	3.4
05100205	Lower Kentucky	Ohio River	8,692,647	4,116,210	17,228,151	1,038.46	491.74	2,058.14	44.6	13.1	2.3	29.4	10.6
05110001	Upper Green	Ohio River	10,008,072	5,104,055	18,721,247	1,231.46	628.04	2,303.59	36.9	2.0	5.3	37.3	18.4
05110002	Barren	Ohio River	7,655,307	4,039,470	16,317,748	1,308.58	690.50	2,789.31	30.8	3.0	7.7	39.8	18.8
05110003	Middle Green	Ohio River	2,672,245	1,310,105	3,897,604	1,006.10	493.26	1,467.45	35.9	2.6	10.5	32.7	18.3
05110004	Rough	Ohio River	2,691,662	1,252,031	4,321,749	957.11	445.20	1,536.75	38.2	1.0	9.5	31.3	20.1
05110005	Lower Green	Ohio River	4,097,362	2,247,067	7,192,296	1,749.90	959.68	3,071.69	21.0	1.9	20.9	38.8	17.4
05110006	Pond	Ohio River	2,626,283	1,447,168	6,000,405	1,280.42	705.55	2,925.44	26.2	1.6	17.3	32.4	22.5
05120101	Upper Wabash	Ohio River	18,202,145	10,230,276	31,235,474	4,323.93	2,430.21	7,420.00	16.4	2.2	21.7	36.4	23.3
05120102	Salamonie	Ohio River	5,010,804	2,596,089	9,753,547	3,516.43	1,821.85	6,844.74	18.2	0.6	25.0	37.2	18.9

05120103	Mississinewa	Ohio River	8,553,821	4,209,172	17,909,616	4,131.18	2,032.88	8,649.69	18.9	3.3	25.0	38.5	14.3
05120104	Eel	Ohio River	5,762,763	2,360,631	9,030,795	2,737.08	1,121.21	4,289.27	19.4	3.1	25.3	37.5	14.7
05120105	Middle Wabash-Deer	Ohio River	6,119,428	2,967,898	10,443,651	3,563.09	1,728.08	6,080.91	15.4	0.5	23.2	40.0	20.9
05120106	Tippecanoe	Ohio River	11,600,734	6,160,389	19,208,853	2,250.65	1,195.17	3,726.70	18.1	1.6	25.5	43.2	11.6
05120107	Wildcat	Ohio River	8,465,134	4,234,693	13,460,649	3,900.70	1,951.33	6,202.61	15.6	3.1	23.3	41.0	16.9
05120108	Middle Wabash-Little Vermilion	Ohio River	11,966,107	5,813,175	20,773,688	2,044.24	993.10	3,548.89	20.2	5.0	26.7	42.5	5.7
05120109	Vermilion	Ohio River	8,729,164	4,719,820	13,896,983	2,364.22	1,278.32	3,763.88	17.6	5.8	27.9	46.7	2.0
05120110	Sugar	Ohio River	5,640,723	2,814,300	10,448,876	2,763.76	1,378.91	5,119.59	17.7	1.2	25.9	42.7	12.5
05120111	Middle Wabash-Busseron	Ohio River	9,291,888	5,018,387	17,516,378	1,774.69	958.48	3,345.51	22.2	9.2	24.0	40.6	3.9
05120112	Embarras	Ohio River	12,820,933	6,867,402	21,788,133	2,009.06	1,076.13	3,414.23	19.6	1.3	27.0	45.0	7.0
05120113	Lower Wabash	Ohio River	6,768,940	3,142,324	12,651,648	1,917.22	890.03	3,583.43	21.2	0.9	24.9	47.6	5.4
05120114	Little Wabash	Ohio River	8,846,040	4,508,090	13,336,241	1,603.79	817.32	2,417.87	22.1	1.7	23.5	41.6	11.1
05120115	Skillet	Ohio River	3,330,344	1,710,588	6,343,973	1,203.59	618.21	2,292.73	27.3	0.3	24.3	42.0	6.2
05120201	Upper White	Ohio River	19,842,956	9,665,965	36,286,879	2,798.64	1,363.28	5,117.88	18.3	25.8	20.2	31.1	4.7
05120202	Lower White	Ohio River	6,959,915	4,250,779	14,987,105	1,661.83	1,014.96	3,578.49	25.4	7.8	18.1	33.2	15.5
05120203	Eel	Ohio River	5,071,831	2,529,204	9,627,193	1,622.13	808.92	3,079.09	26.0	2.5	25.2	39.3	7.0
05120204	Driftwood	Ohio River	8,791,500	4,606,803	17,060,026	2,890.21	1,514.49	5,608.49	20.6	3.9	26.9	40.1	8.4
05120205	Flatrock-Haw	Ohio River	4,416,572	2,284,759	7,069,712	3,234.23	1,673.12	5,177.11	18.9	0.7	27.7	41.5	11.1
05120206	Upper East Fork White	Ohio River	5,550,486	2,255,073	10,057,294	2,450.23	995.49	4,439.74	19.3	4.2	22.3	38.0	16.1
05120207	Muscatatuck	Ohio River	3,835,750	1,915,123	5,461,082	1,296.47	647.31	1,845.83	30.1	2.4	20.1	37.9	9.5
05120208	Lower East Fork White	Ohio River	5,479,437	2,871,799	9,105,099	1,038.43	544.24	1,725.54	39.1	5.6	13.4	25.2	16.6
05120209	Patoka	Ohio River	3,612,543	1,684,766	6,685,839	1,614.77	753.07	2,988.50	23.2	7.2	14.9	28.9	25.8
05130101	Upper Cumberland	Cumberland	2,891,409	1,656,264	5,784,358	479.04	274.40	958.33	81.8	8.6	0.1	7.6	1.8
05130102	Rockcastle	Cumberland	1,158,783	568,635	1,955,658	586.03	287.58	989.04	73.1	0.6	0.1	19.2	7.0
05130103	Upper Cumberland-Lake Cumberland	Cumberland	4,595,256	2,543,824	7,704,048	943.55	522.33	1,581.88	46.7	1.4	3.6	32.9	15.4
05130104	South Fork Cumberland	Cumberland	2,276,855	1,067,953	3,928,226	634.84	297.77	1,095.28	76.2	1.1	0.1	7.8	14.7
05130105	Obey	Cumberland	1,414,120	679,392	2,449,980	576.17	276.81	998.22	55.2	1.9	6.2	18.0	18.7
05130106	Upper Cumberland-Cordell Hull	Cumberland	1,751,786	834,333	2,764,627	847.08	403.44	1,336.84	59.8	1.7	12.0	18.0	8.5
05130107	Collins	Cumberland	3,188,557	1,367,359	5,315,834	1,556.55	667.50	2,595.01	46.3	1.1	7.5	27.1	18.1
05130108	Caney	Cumberland	4,410,150	2,369,863	9,185,919	951.98	511.56	1,982.88	56.0	3.4	6.5	22.7	11.4
05130201	Lower Cumberland-Old Hickory Lake	Cumberland	2,295,640	1,067,044	4,242,092	895.98	416.46	1,655.67	56.8	6.9	4.8	21.1	10.5
05130202	Lower Cumberland-Sycamore	Cumberland	3,650,635	1,853,460	6,730,649	2,170.92	1,102.19	4,002.50	18.7	75.5	0.9	3.8	1.2
05130203	Stones	Cumberland	1,822,036	887,599	3,617,458	760.37	370.41	1,509.63	63.5	4.2	5.5	15.8	11.0
05130204	Harpeth	Cumberland	1,725,057	819,686	2,431,601	768.88	365.34	1,083.79	62.3	5.9	3.7	18.2	9.9
05130205	Lower Cumberland	Cumberland	5,128,629	2,819,105	7,818,734	845.64	464.83	1,289.21	35.7	2.8	15.8	38.1	7.6
05130206	Red	Cumberland	6,878,592	3,391,812	13,431,457	1,827.78	901.27	3,569.01	19.9	4.4	21.6	43.4	10.6
05140101	Silver-Little Kentucky	Ohio River	5,313,050	2,678,767	8,616,441	1,618.80	816.17	2,625.28	25.0	54.2	5.3	12.5	2.9
05140102	Salt	Ohio River	4,792,570	2,159,649	8,639,544	1,243.05	560.15	2,240.84	35.7	20.4	5.2	25.1	13.6
05140103	Rolling Fork	Ohio River	4,455,453	2,074,106	7,949,349	1,187.61	552.86	2,118.91	41.1	3.4	5.9	32.6	16.9
05140104	Blue-Sinking	Ohio River	4,750,680	1,910,484	7,216,515	973.04	391.31	1,478.09	43.2	1.3	9.5	33.7	12.3
05140201	Lower Ohio-Little Pigeon	Ohio River	4,816,030	1,916,535	8,775,912	1,316.66	523.96	2,399.26	30.8	9.5	17.7	31.2	10.7
05140202	Highland-Pigeon	Ohio River	5,728,707	2,790,410	10,698,021	2,210.28	1,076.61	4,127.56	18.3	28.2	19.5	30.8	3.2
05140203	Lower Ohio-Bay	Ohio River	2,261,892	915,042	4,332,109	806.40	326.23	1,544.47	47.0	1.2	17.3	29.3	5.2
05140204	Saline	Ohio River	3,660,641	1,733,217	6,733,979	1,208.18	572.04	2,222.52	30.7	2.0	21.7	41.1	4.5
05140205	Tradewater	Ohio River	2,335,754	1,265,657	4,183,322	958.43	519.34	1,716.54	30.4	4.1	18.7	33.6	13.2
05140206	Lower Ohio	Lower Ohio	2,903,158	1,513,616	4,755,142	1,093.58	570.16	1,791.20	33.5	8.2	18.2	30.9	9.2
07010101	Mississippi Headwaters	Upper Miss. R	178,665	85,032	312,401	37.73	17.96	65.98	89.6	0.8	2.1	4.3	3.3
07010102	Leech Lake	Upper Miss. R	79,338	31,977	130,230	24.32	9.80	39.93	91.0	0.2	1.7	3.8	3.3
07010103	Prairie-Willow	Upper Miss. R	938,931	447,996	1,332,296	158.34	75.55	224.68	70.1	21.4	2.1	3.5	2.9

07010104	Elk-Nokasippi	Upper Miss. R	1,484,605	652,415	2,315,370	318.66	140.04	496.97	43.2	5.1	10.0	15.6	26.0
07010105	Pine	Upper Miss. R	88,269	43,733	160,484	44.43	22.01	80.79	90.9	0.4	2.1	3.0	3.6
07010106	Crow Wing	Upper Miss. R	908,438	419,466	1,570,647	180.78	83.47	312.56	50.2	2.0	13.3	19.3	15.1
07010107	Redeye	Upper Miss. R	804,105	407,742	1,336,834	385.64	195.55	641.13	29.2	1.9	17.2	27.6	24.0
07010108	Long Prairie	Upper Miss. R	740,254	362,033	1,226,579	294.49	144.03	487.96	29.9	4.5	18.7	20.7	26.2
07010201	Platte-Spunk	Upper Miss. R	1,755,340	860,733	2,954,622	684.99	335.89	1,152.99	19.2	2.4	12.3	23.9	42.3
07010202	Sauk	Upper Miss. R	2,063,406	936,801	3,377,488	805.50	365.70	1,318.49	15.4	3.9	17.1	24.3	39.3
07010203	Clearwater-Elk	Upper Miss. R	1,716,895	879,545	3,509,134	600.38	307.57	1,227.10	19.7	21.2	15.8	23.2	20.1
07010204	Crow	Upper Miss. R	2,989,831	1,276,260	5,217,322	780.39	333.12	1,361.80	20.4	3.3	22.0	30.1	24.2
07010205	South Fork Crow	Upper Miss. R	5,789,970	2,377,504	9,739,083	1,761.69	723.39	2,963.27	17.6	2.4	23.6	37.7	18.7
07010206	Twin Cities	Upper Miss. R	4,541,305	2,160,843	6,382,835	1,945.36	925.64	2,734.22	8.7	85.8	1.5	2.9	1.2
07010207	Rum	Upper Miss. R	1,657,220	840,212	2,396,364	397.86	201.71	575.31	37.9	5.9	17.7	22.0	16.5
07020001	Upper Minnesota	Upper Miss. R	894,153	400,894	1,517,902	166.24	74.53	282.20	25.7	0.7	27.0	35.7	10.9
07020002	Pomme De Terre	Upper Miss. R	1,649,269	774,850	2,645,903	693.80	325.95	1,113.05	18.7	1.3	27.4	37.4	15.2
07020003	Lac Qui Parle	Upper Miss. R	2,044,003	848,758	3,940,421	702.37	291.65	1,354.02	21.4	5.5	27.7	33.2	12.2
07020004	Hawk-Yellow Medicine	Upper Miss. R	6,878,621	3,320,612	13,812,556	1,331.73	642.89	2,674.18	18.4	1.8	25.5	36.6	17.7
07020005	Chippewa	Upper Miss. R	3,875,477	2,140,612	5,720,489	720.13	397.76	1,062.96	20.3	1.0	27.9	36.2	14.6
07020006	Redwood	Upper Miss. R	1,963,746	827,802	3,880,524	1,040.27	438.52	2,055.67	18.6	4.2	24.9	32.3	20.1
07020007	Middle Minnesota	Upper Miss. R	8,276,777	3,384,525	13,100,387	2,281.05	932.76	3,610.42	16.3	3.3	21.4	33.9	25.0
07020008	Cottonwood	Upper Miss. R	5,323,437	3,128,613	8,984,438	1,643.37	965.81	2,773.53	17.0	1.2	24.4	34.7	22.6
07020009	Blue Earth	Upper Miss. R	9,823,085	4,912,717	16,440,576	2,491.35	1,245.97	4,169.70	14.8	1.3	21.5	36.3	26.2
07020010	Watonwan	Upper Miss. R	4,180,988	2,370,847	7,533,213	1,823.90	1,034.25	3,286.25	15.8	1.1	23.4	35.7	24.0
07020011	Le Sueur	Upper Miss. R	7,058,298	3,096,430	12,885,627	2,438.53	1,069.77	4,451.78	16.7	0.7	24.3	36.5	21.7
07020012	Lower Minnesota	Upper Miss. R	9,049,993	4,016,152	15,357,911	1,785.53	792.37	3,030.06	17.0	14.2	18.8	31.0	19.0
07030001	Upper St. Croix	Upper Miss. R	1,271,528	653,594	2,336,371	237.90	122.29	437.14	78.1	0.8	4.1	7.3	9.7
07030002	Namekagon	Upper Miss. R	538,401	247,960	860,711	203.28	93.62	324.98	87.7	0.0	2.7	4.0	5.6
07030003	Kettle	Upper Miss. R	747,900	346,047	1,611,936	271.33	125.54	584.78	69.1	5.4	2.9	9.3	13.3
07030004	Snake	Upper Miss. R	921,110	495,598	1,565,604	360.79	194.12	613.23	49.5	2.4	9.6	21.3	17.2
07030005	Lower St. Croix	Upper Miss. R	3,654,605	1,859,685	5,208,245	546.88	278.28	779.36	35.8	5.2	15.5	21.0	22.4
07040001	Rush-Vermillion	Upper Miss. R	2,803,287	1,323,691	4,471,686	968.92	457.52	1,545.58	23.4	8.4	19.3	25.6	23.3
07040002	Cannon	Upper Miss. R	6,203,191	3,130,441	10,955,128	1,622.19	818.64	2,864.86	18.3	2.1	20.6	35.9	23.0
07040003	Buffalo-Whitewater	Upper Miss. R	3,239,390	1,529,545	5,288,097	935.89	441.90	1,527.79	24.8	3.9	13.6	22.0	35.7
07040004	Zumbro	Upper Miss. R	5,622,571	2,717,691	10,119,074	1,507.73	728.77	2,713.49	17.8	6.1	18.1	32.2	25.8
07040005	Trempealeau	Upper Miss. R	1,768,575	799,820	3,327,826	931.89	421.44	1,753.49	25.3	2.2	16.9	17.4	38.2
07040006	La Crosse-Pine	Upper Miss. R	1,370,616	702,253	2,388,193	805.27	412.59	1,403.12	30.3	16.4	11.5	14.4	27.5
07040007	Black	Upper Miss. R	4,083,755	2,217,549	7,847,187	697.42	378.71	1,340.13	29.5	1.9	20.5	13.7	34.5
07040008	Root	Upper Miss. R	5,777,620	2,861,047	10,689,371	1,332.18	659.69	2,464.71	21.7	1.0	16.2	34.2	26.8
07050001	Upper Chippewa	Upper Miss. R	1,268,233	688,463	1,937,259	253.45	137.59	387.15	73.0	0.4	8.3	4.7	13.6
07050002	Flambeau	Upper Miss. R	513,585	251,255	1,066,280	169.89	83.11	352.72	73.7	9.4	6.1	2.6	8.2
07050003	South Fork Flambeau	Upper Miss. R	351,099	186,387	523,598	182.77	97.03	272.57	80.6	1.4	7.0	2.8	8.3
07050004	Jump	Upper Miss. R	764,555	440,168	1,332,923	351.08	202.13	612.08	55.7	0.8	14.4	7.1	22.0
07050005	Lower Chippewa	Upper Miss. R	3,687,043	1,721,416	7,035,030	691.45	322.82	1,319.31	27.3	5.0	21.3	16.2	30.3
07050006	Eau Claire	Upper Miss. R	1,476,897	777,233	2,690,259	627.64	330.30	1,143.29	29.8	1.4	21.7	14.4	32.6
07050007	Red Cedar	Upper Miss. R	2,738,080	1,461,279	3,870,945	564.02	301.01	797.38	29.0	2.7	16.3	18.4	33.5
07060001	Coon-Yellow	Upper Miss. R	3,471,933	1,899,352	6,617,948	943.55	516.18	1,798.53	29.1	1.8	14.4	24.1	30.6
07060002	Upper Iowa	Upper Miss. R	4,117,194	2,039,610	6,663,427	1,588.25	786.80	2,570.48	19.9	0.8	15.7	37.8	25.8
07060003	Grant-Little Maquoketa	Upper Miss. R	3,841,915	1,839,892	6,267,409	1,327.01	635.51	2,164.79	21.7	3.1	15.6	22.5	37.0
07060004	Turkey	Upper Miss. R	8,119,859	4,574,837	13,945,016	1,850.45	1,042.57	3,177.96	17.9	0.5	17.2	37.3	27.1
07060005	Apple-Plum	Upper Miss. R	4,658,255	1,977,784	8,233,112	1,214.07	515.47	2,145.78	23.2	5.0	16.5	32.6	22.6

07060006	Maquoketa	Upper Miss. R	9,057,444	4,884,837	15,592,635	1,863.99	1,005.28	3,208.92	16.7	0.7	17.5	34.5	30.5
07070001	Upper Wisconsin	Upper Miss. R	681,330	348,963	1,050,432	119.74	61.33	184.61	76.0	13.8	3.9	3.6	2.7
07070002	Lake Dubay	Upper Miss. R	4,489,887	2,120,412	8,386,502	638.69	301.63	1,192.98	27.1	6.0	22.1	13.9	30.9
07070003	Castle Rock	Upper Miss. R	14,843,050	8,077,187	25,483,435	1,735.13	944.21	2,978.98	9.2	71.6	5.9	7.1	6.2
07070004	Baraboo	Upper Miss. R	1,693,683	925,593	3,122,424	1,003.96	548.66	1,850.86	24.8	3.1	19.9	18.4	33.8
07070005	Lower Wisconsin	Upper Miss. R	4,978,846	2,739,689	9,011,932	816.99	449.56	1,478.79	31.7	1.6	17.7	18.5	30.5
07070006	Kickapoo	Upper Miss. R	1,753,525	795,296	3,297,234	875.58	397.11	1,646.39	32.4	0.5	18.3	15.2	33.6
07080101	Copperas-Duck	Upper Miss. R	5,217,816	2,257,732	8,091,850	2,023.97	875.77	3,138.80	14.3	37.1	13.4	27.6	7.6
07080102	Upper Wapsipinicon	Upper Miss. R	10,465,919	4,308,063	15,308,742	2,583.28	1,063.35	3,778.62	17.2	0.8	22.2	38.3	21.4
07080103	Lower Wapsipinicon	Upper Miss. R	5,888,791	3,376,037	10,127,518	2,315.51	1,327.48	3,982.21	16.1	0.6	21.9	45.2	16.2
07080104	Flint-Henderson	Upper Miss. R	9,900,613	4,867,970	20,452,109	1,601.42	787.39	3,308.12	18.3	4.6	23.4	43.3	10.4
07080105	South Skunk	Upper Miss. R	9,618,580	5,109,884	16,521,082	2,004.89	1,065.10	3,443.64	18.1	1.0	22.0	39.4	19.5
07080106	North Skunk	Upper Miss. R	3,472,885	1,884,705	5,953,894	1,538.36	834.86	2,637.36	20.6	1.0	19.9	39.4	19.1
07080107	Skunk	Upper Miss. R	7,120,603	3,266,716	12,024,071	1,664.01	763.40	2,809.91	19.4	1.1	21.2	36.8	21.5
07080201	Upper Cedar	Upper Miss. R	11,948,697	6,069,647	18,325,000	2,741.05	1,392.39	4,203.79	16.4	1.5	21.9	37.5	22.8
07080202	Shell Rock	Upper Miss. R	6,781,951	2,789,468	10,954,675	2,418.83	994.88	3,907.07	17.7	1.2	24.0	40.5	16.6
07080203	Winnebago	Upper Miss. R	4,362,849	2,000,424	6,585,816	2,456.25	1,126.23	3,707.77	18.0	2.7	23.5	42.6	13.3
07080204	West Fork Cedar	Upper Miss. R	5,419,357	2,523,177	8,467,235	2,447.89	1,139.70	3,824.60	16.0	0.4	22.6	38.1	22.9
07080205	Middle Cedar	Upper Miss. R	12,242,433	6,608,055	18,803,944	1,956.67	1,056.14	3,005.38	17.4	3.7	23.2	38.2	17.4
07080206	Lower Cedar	Upper Miss. R	5,264,704	2,535,952	8,481,080	1,872.59	902.01	3,016.61	17.6	6.7	21.6	40.8	13.5
07080207	Upper Iowa	Upper Miss. R	12,932,968	6,819,695	20,737,994	3,415.29	1,800.92	5,476.42	13.5	0.5	19.2	30.1	36.7
07080208	Middle Iowa	Upper Miss. R	5,922,828	2,996,221	10,610,549	1,384.20	700.24	2,479.76	20.7	2.2	21.6	42.2	13.4
07080209	Lower Iowa	Upper Miss. R	8,059,824	4,117,389	13,324,046	1,824.75	932.18	3,016.58	18.1	3.1	19.7	37.1	22.1
07090001	Upper Rock	Upper Miss. R	7,485,987	4,110,520	14,768,761	980.17	538.21	1,933.73	20.2	14.6	18.9	21.6	24.7
07090002	Crawfish	Upper Miss. R	2,450,335	1,007,757	4,728,342	1,197.62	492.55	2,311.02	19.4	2.6	21.9	23.9	32.1
07090003	Pecatonica	Upper Miss. R	7,438,895	3,596,265	15,028,250	1,529.06	739.21	3,089.05	19.5	2.5	18.8	31.9	27.4
07090004	Sugar	Upper Miss. R	2,571,629	1,318,860	3,824,298	1,320.46	677.20	1,963.67	20.6	3.0	18.0	28.7	29.7
07090005	Lower Rock	Upper Miss. R	9,520,867	4,489,613	18,566,766	1,702.83	802.98	3,320.71	18.0	9.1	23.1	40.0	9.8
07090006	Kishwaukee	Upper Miss. R	6,508,092	3,084,800	9,771,912	1,975.33	936.30	2,965.97	17.5	4.6	23.2	41.0	13.7
07090007	Green	Upper Miss. R	6,450,222	3,294,025	10,325,917	2,246.14	1,147.07	3,595.76	16.5	0.9	25.1	46.8	10.7
07100001	Des Moines Headwaters	Upper Miss. R	3,680,352	1,829,257	6,418,076	1,152.58	572.87	2,009.95	18.6	1.5	25.5	33.8	20.6
07100002	Upper Des Moines	Upper Miss. R	5,528,322	2,421,781	7,654,277	1,952.04	855.13	2,702.71	15.7	1.0	22.7	33.5	27.1
07100003	East Fork Des Moines	Upper Miss. R	7,160,162	3,599,895	11,685,787	2,097.31	1,054.46	3,422.93	15.5	0.5	23.2	39.6	21.1
07100004	Middle Des Moines	Upper Miss. R	9,406,134	5,220,943	14,498,262	2,133.83	1,184.40	3,289.01	18.4	0.9	24.3	43.3	13.0
07100005	Boone	Upper Miss. R	5,900,104	2,838,197	9,103,860	2,556.48	1,229.77	3,944.65	14.9	1.3	22.3	36.2	25.4
07100006	North Raccoon	Upper Miss. R	17,486,568	8,748,447	31,299,034	2,668.15	1,334.86	4,775.69	16.0	0.6	23.4	39.3	20.7
07100007	South Raccoon	Upper Miss. R	5,746,240	2,477,459	9,909,712	1,958.53	844.41	3,377.60	18.1	0.6	21.5	32.8	27.0
07100008	Lake Red Rock	Upper Miss. R	7,411,914	3,138,119	13,848,773	1,172.25	496.32	2,190.29	26.8	11.7	18.5	32.1	10.9
07100009	Lower Des Moines	Upper Miss. R	6,355,521	3,244,562	9,572,308	1,139.79	581.87	1,716.68	28.8	7.4	15.0	33.8	15.1
07110001	Bear-Wyaconda	Upper Miss. R	5,504,334	3,055,031	11,270,430	1,249.27	693.37	2,557.96	24.5	5.9	20.4	38.3	10.8
07110002	North Fabius	Upper Miss. R	2,225,495	1,151,313	3,653,730	935.98	484.21	1,536.66	34.0	0.0	17.2	38.8	9.9
07110003	South Fabius	Upper Miss. R	1,758,953	899,607	3,125,048	1,081.39	553.07	1,921.26	29.4	0.0	18.7	43.8	8.2
07110004	The Sny	Upper Miss. R	5,042,250	2,444,437	9,719,675	976.97	473.63	1,883.25	26.3	1.7	19.1	41.2	11.7
07110005	North Fork Salt	Upper Miss. R	2,695,394	1,305,168	5,380,085	1,165.96	564.58	2,327.29	28.6	0.0	21.4	38.5	11.5
07110006	South Fork Salt	Upper Miss. R	3,907,996	1,796,388	7,033,304	1,251.04	575.06	2,251.52	25.4	2.0	20.2	41.8	10.6
07110007	Salt	Upper Miss. R	2,358,800	1,305,788	3,913,123	1,149.10	636.12	1,906.30	25.4	0.0	21.9	44.1	8.6
07110008	Cuivre	Upper Miss. R	3,858,857	1,802,109	6,167,830	1,194.87	558.01	1,909.83	25.8	1.5	21.0	41.0	10.8
07110009	Peruque-Piasa	Upper Miss. R	2,005,686	974,460	3,388,854	1,153.60	560.48	1,949.15	25.3	28.2	14.4	27.5	4.6
07120001	Kankakee	Upper Miss. R	16,726,834	6,960,580	24,820,337	2,131.54	887.00	3,162.91	21.3	6.7	24.8	40.9	6.3

07120002	Iroquois	Upper Miss. R	15,364,160	8,663,649	23,667,073	2,774.81	1,564.68	4,274.34	16.8	0.6	26.2	46.6	9.7
07120003	Chicago	Upper Miss. R	6,522,917	3,159,767	12,977,836	4,143.53	2,007.17	8,243.87	5.8	93.0	0.4	0.8	0.1
07120004	Des Plaines	Upper Miss. R	19,335,826	10,169,930	33,276,562	4,950.67	2,603.87	8,520.00	9.3	83.7	2.5	3.9	0.7
07120005	Upper Illinois	Upper Miss. R	5,920,960	3,217,017	11,704,672	2,311.01	1,255.63	4,568.44	20.6	7.8	25.4	42.5	3.8
07120006	Upper Fox	Upper Miss. R	3,389,797	1,658,831	6,809,636	862.20	421.93	1,732.04	26.3	28.1	15.5	20.8	9.3
07120007	Lower Fox	Upper Miss. R	7,710,903	3,592,817	14,009,369	2,697.90	1,257.06	4,901.62	18.5	13.5	21.1	38.8	8.1
07130001	Lower Illinois-Senachwine Lake	Upper Miss. R	8,313,487	4,541,624	14,434,747	1,625.56	888.04	2,822.47	19.2	2.9	24.6	49.0	4.2
07130002	Vermilion	Upper Miss. R	8,586,113	3,998,294	14,128,099	2,492.70	1,160.78	4,101.64	18.7	1.6	28.1	45.8	5.8
07130003	Lower Illinois-Lake Chautauqua	Upper Miss. R	5,971,231	3,196,000	12,016,998	1,530.41	819.13	3,079.93	20.3	15.5	19.9	39.3	5.0
07130004	Mackinaw	Upper Miss. R	5,247,582	2,735,797	9,520,327	1,757.61	916.32	3,188.71	17.3	1.6	26.1	47.3	7.7
07130005	Spoon	Upper Miss. R	8,833,883	3,975,579	15,021,504	1,823.91	820.83	3,101.46	19.5	1.6	25.1	45.8	8.0
07130006	Upper Sangamon	Upper Miss. R	7,615,421	3,959,168	12,684,422	2,026.59	1,053.60	3,375.54	15.6	9.7	25.2	47.1	2.4
07130007	South Fork Sangamon	Upper Miss. R	5,751,739	2,657,502	9,065,299	1,924.12	889.01	3,032.59	16.7	5.2	25.7	48.1	4.3
07130008	Lower Sangamon	Upper Miss. R	4,464,691	2,234,322	7,857,100	1,819.71	910.66	3,202.38	17.6	8.3	22.8	45.1	6.2
07130009	Salt	Upper Miss. R	9,477,724	4,513,878	17,046,604	1,945.09	926.37	3,498.43	16.1	4.7	26.2	47.2	5.8
07130010	La Moine	Upper Miss. R	5,209,083	2,059,812	9,207,302	1,436.07	567.86	2,538.32	21.9	1.5	25.1	43.8	7.6
07130011	Lower Illinois	Upper Miss. R	8,106,820	4,076,554	16,034,725	1,363.92	685.85	2,697.73	21.8	2.8	23.1	41.5	10.8
07130012	Macoupin	Upper Miss. R	3,613,690	1,856,060	5,898,749	1,463.65	751.76	2,389.16	20.5	1.5	25.1	43.0	9.8
07140101	Cahokia-Joachim	Mississippi R	7,195,506	2,736,017	13,715,133	1,676.97	637.65	3,196.43	17.2	59.6	6.7	13.4	3.1
07140102	Meramec	Meramec	3,374,910	1,808,594	5,459,530	605.61	324.54	979.68	57.5	19.3	0.8	17.1	5.3
07140103	Bourbeuse	Meramec	1,559,422	792,529	2,987,010	716.25	364.01	1,371.95	47.9	2.0	2.7	32.2	15.2
07140104	Big	Meramec	1,231,782	520,213	2,079,061	489.92	206.91	826.91	73.2	1.8	1.5	13.5	10.0
07140105	Upper Mississippi-Cape Girardeau	Mississippi R	4,117,023	2,056,523	7,033,724	950.30	474.69	1,623.55	38.0	4.1	15.0	34.6	8.3
07140106	Big Muddy	Big Muddy	5,473,645	2,720,451	9,778,070	874.57	434.67	1,562.33	32.3	6.7	18.2	36.3	6.4
07140107	Whitewater	Whitewater	2,735,979	1,308,600	5,416,429	819.59	392.01	1,622.55	45.7	0.0	10.3	34.1	9.9
07140108	Cache	Cashe	869,290	398,776	1,583,585	977.22	448.29	1,780.21	40.0	0.7	18.1	37.4	3.8
07140201	Upper Kaskaskia	Kaskaskia	6,367,594	3,129,135	11,094,218	1,572.42	772.71	2,739.61	18.8	2.5	27.8	46.2	4.7
07140202	Middle Kaskaskia	Kaskaskia	4,883,184	2,252,682	7,392,692	1,103.16	508.90	1,670.09	22.9	2.8	20.7	40.7	12.9
07140203	Shoal	Kaskaskia	3,053,296	1,648,439	5,358,078	1,281.98	692.13	2,249.69	21.4	3.2	21.4	40.3	13.6
07140204	Lower Kaskaskia	Kaskaskia	6,055,893	2,494,506	11,514,787	1,463.27	602.74	2,782.28	20.9	9.4	19.4	39.8	10.5
09010001	Upper Souris	Souris River	2,367,756	1,118,363	4,530,225	416.10	196.53	796.12	20.0	2.4	38.4	38.8	0.4
09010002	Des Lacs	Souris River	159,650	75,306	284,072	61.06	28.80	108.64	18.6	0.0	38.2	42.3	0.9
09010003	Lower Souris	Souris River	503,798	246,100	902,453	85.36	41.70	152.91	23.2	0.0	33.6	42.9	0.3
09010004	Willow	Souris River	407,258	197,576	845,659	84.22	40.86	174.89	26.3	0.0	35.5	37.1	1.1
09010005	Deep	Souris River	201,378	84,996	405,621	43.97	18.56	88.57	20.9	0.0	37.5	40.9	0.7
09020101	Bois De Sioux	Red River	912,675	480,114	1,822,644	450.99	237.24	900.64	18.6	0.2	32.6	43.6	5.0
09020102	Mustinka	Red River	1,658,739	747,113	2,878,453	614.81	276.92	1,066.89	18.2	1.0	32.5	42.9	5.4
09020103	Otter Tail	Red River	1,557,558	881,227	3,098,342	286.94	162.34	570.79	24.8	4.2	25.2	30.3	15.5
09020104	Upper Red	Red River	1,165,165	625,978	1,903,682	1,097.05	589.38	1,792.39	13.6	25.8	24.7	33.1	2.7
09020105	Western Wild Rice	Red River	3,650,955	1,746,936	5,049,596	616.88	295.17	853.21	16.9	0.0	27.3	51.5	4.3
09020106	Buffalo	Red River	1,689,662	894,642	3,428,563	496.77	263.03	1,008.01	20.5	0.9	34.3	35.9	8.5
09020107	Elm-Marsh	Red River	1,946,756	989,441	3,630,063	888.66	451.66	1,657.06	14.0	0.3	35.6	48.9	1.2
09020108	Eastern Wild Rice	Red River	2,212,028	1,108,857	3,827,960	514.25	257.79	889.92	22.6	1.2	35.6	34.4	6.2
09020109	Goose	Red River	1,019,383	526,606	1,743,117	298.40	154.15	510.25	13.7	0.0	33.3	52.7	0.3
09020201	Devils Lake	Red River	95,162	43,810	178,992	10.29	4.74	19.36	23.2	0.0	31.9	44.1	0.8
09020202	Upper Sheyenne	Red River	333,547	118,423	750,435	64.47	22.89	145.05	23.3	0.0	32.7	42.9	1.1
09020203	Middle Sheyenne	Red River	672,198	305,126	1,064,074	127.28	57.78	201.49	24.8	0.0	32.4	41.7	1.1
09020204	Lower Sheyenne	Red River	2,936,218	1,272,539	5,229,634	605.53	262.43	1,078.49	18.3	1.4	31.1	47.3	1.9
09020205	Maple	Red River	1,873,445	830,617	3,132,112	481.62	213.53	805.19	15.4	0.0	34.5	48.8	1.2

09020301	Sandhill-Wilson	Red River	1,707,449	863,774	2,982,831	568.58	287.64	993.28	15.4	0.1	36.9	46.1	1.5
09020302	Red Lakes	Red River	21,604	10,119	45,689	4.52	2.12	9.55	91.3	0.0	1.8	3.0	3.9
09020303	Red Lake	Red River	1,760,501	857,531	2,906,949	543.15	264.56	896.85	21.0	6.5	38.1	31.8	2.7
09020304	Thief	Red River	255,296	115,422	395,957	90.30	40.82	140.05	24.8	0.0	38.5	34.1	2.6
09020305	Clearwater	Red River	965,816	459,471	1,448,377	279.44	132.94	419.06	32.0	1.4	28.6	31.1	7.0
09020306	Grand Marais-Red	Red River	903,479	366,683	1,467,599	744.46	302.14	1,209.28	13.6	6.1	37.8	40.7	1.7
09020307	Turtle	Red River	829,093	353,572	1,456,134	468.64	199.86	823.07	9.6	25.7	26.1	38.1	0.5
09020308	Forest	Red River	545,386	261,543	990,825	203.59	97.63	369.88	12.1	0.0	32.6	54.9	0.4
09020309	Snake	Red River	1,081,603	451,389	1,999,140	395.81	165.18	731.58	16.0	1.6	43.0	37.3	2.1
09020310	Park	Red River	1,422,671	689,516	2,722,398	466.39	226.04	892.48	11.7	1.6	30.9	55.5	0.2
09020311	Lower Red	Red River	1,420,144	597,111	2,402,888	712.73	299.67	1,205.94	15.2	0.2	41.8	41.3	1.6
09020312	Two Rivers	Red River	1,531,192	748,649	2,778,037	521.09	254.78	945.41	18.3	0.6	41.7	35.9	3.5
09020313	Pembina	Red River	1,556,799	661,167	2,734,295	448.58	190.51	787.86	13.9	0.0	32.6	53.1	0.3
09020314	Roseau	Rainy River	1,036,267	541,603	1,945,456	245.28	128.19	460.47	35.5	1.3	30.2	27.4	5.6
09030001	Rainy Headwaters	Rainy River	60,677	32,529	109,544	7.90	4.23	14.26	100.0	0.0	0.0	0.0	0.0
09030002	Vermilion	Rainy River	143,055	66,712	257,415	53.46	24.93	96.20	95.8	3.2	0.4	0.2	0.4
09030003	Rainy Lake	Rainy River	230,103	85,356	375,465	80.96	30.03	132.10	96.6	0.0	1.5	1.3	0.6
09030004	Upper Rainy	Rainy River	145,047	73,289	276,218	48.11	24.31	91.61	98.3	0.0	0.8	0.6	0.3
09030005	Little Fork	Rainy River	796,609	359,280	1,592,027	162.54	73.31	324.84	94.7	0.5	2.0	1.5	1.4
09030006	Big Fork	Rainy River	737,181	347,566	1,265,074	134.73	63.52	231.20	96.4	0.3	0.9	1.7	0.7
09030007	Rapid	Rainy River	370,904	169,234	646,509	135.28	61.72	235.80	91.0	0.0	3.9	4.3	0.8
09030008	Lower Rainy	Rainy River	85,541	40,360	139,502	68.33	32.24	111.44	66.1	0.0	14.1	17.1	2.7
09030009	Lake of the Woods	Rainy River	224,601	119,978	352,367	104.82	55.99	164.44	55.0	0.1	19.0	21.6	4.3

Table S6_TP. Total annual phosphorus and nitrogen loads and yields, with confidence intervals, for all tributaries to each lake with a drainage area greater than 150 square kilometers.
Tributaries to each Great Lake are ranked based on their relative loads and yields. A value of 1 indicates it has the largest load or relatively largest yield.
All loads and yields are adjusted to remove known spatial biases by only predicting areas that are not monitored. This results in very small standard errors and confidence intervals for basins for which a large percentage is monitored.

Supplemental Material to Robertson and Saad, 2011, Journal of the American Water Resources Association, Nutrient Inputs to the Laurentian Great Lakes By Source and River Basin Estimated Using SPARROW Watershed Models.

Yield is load per unit area of the HUC8 watershed.
[%, percent; CI, Confidence interval; kg, kilogram; km^2, square kilometer]

											Percent Contribution by Source					
Great Lake/River Basin	River Name	MRB Identification Number	Area (km^2)	Total Phosphorus Load (kg)	Total Phosphorus Load Rank	Standard Error in Phosphorus Load Estimate (kg)	Lower 95% CI Total Phosphorus Load (kg)	Upper 95% CI Total Phosphorus Load (kg)	Total Phosphorus Yield (kg/km^2)	Total Phosphorus Yield Rank	Forest, wetland, shrub	Point sources	Urban and open	Fertilizers (farm)	Manure (confined)	Manure (unconfined)
Erie	MAUMEE R	11873	16,948	1,653,319	1	103,727	1,547,561	1,845,445	97.6	8	1.7	26.1	9.0	36.8	24.4	2.0
Erie	RIVER ROUGE	11782	1,208	711,507	2	437,646	234,141	1,300,085	589.1	1	0.1	92.4	7.2	0.2	0.1	0.0
Erie	SANDUSKY R	11898	3,462	366,155	3	3,992	362,242	372,639	105.8	6	2.1	9.3	7.3	56.4	22.5	2.3
Erie	CUYAHOGA R	11920	2,091	267,787	4	3,422	264,956	271,980	128.1	4	4.1	71.3	18.7	3.3	1.8	0.7
Erie	CATTARAUGUS CR	11946	1,449	146,757	5	5,777	140,338	155,017	101.3	7	26.7	5.9	7.1	14.6	38.0	7.6
Erie	CLINTON R	12280	2,570	140,416	6	13,432	127,145	152,497	54.6	15	3.3	51.2	37.2	5.6	1.9	0.6
Erie	HURON R	11910	1,078	115,679	7	4,607	110,543	121,226	107.3	5	4.2	11.8	6.0	59.4	16.2	2.4
Erie	BLACK R	11753	1,853	107,066	8	473	106,505	107,703	57.8	12	8.6	4.6	11.3	42.2	27.2	6.0
Erie	ROCKY R	11917	756	98,123	9	6,713	91,210	106,283	129.8	3	8.9	48.7	23.5	10.5	6.6	1.8
Erie	VERMILLION R	11913	678	97,247	10	551	96,619	98,369	143.5	2	8.0	8.9	4.0	53.3	21.5	4.2
Erie	GRAND R	11931	1,839	94,147	11	2,410	92,007	96,301	51.2	16	24.8	4.5	11.4	31.8	20.9	6.6
Erie	BUFFALO CR	11961	1,167	84,732	12	35,095	46,265	128,505	72.6	9	13.4	28.3	13.9	11.7	28.3	4.4
Erie	PORTAGE R	11892	1,477	81,813	13	47,501	33,766	171,518	55.4	13	1.7	14.7	10.2	64.7	7.9	0.9
Erie	HURON R	12520	2,390	65,567	14	16,970	46,555	94,030	27.4	26	4.5	49.4	35.1	6.9	3.3	0.7
Erie	BLACK R	11914	1,230	54,786	15	12,509	40,452	69,020	44.5	18	7.6	28.8	17.8	28.1	14.6	3.0
Erie	RIVER RAISIN	11795	2,843	39,435	16	47	39,397	39,502	13.9	29	4.1	32.5	15.9	29.9	15.1	2.5
Erie	CHAGRIN R	11927	673	36,809	17	6,273	30,220	45,400	54.7	14	13.8	40.8	32.3	8.2	3.1	1.9
Erie	BELLE R	11763	577	34,561	18	20,267	13,792	63,067	59.8	10	7.4	53.5	9.6	17.9	8.7	2.9
Erie	TOUSSAINT CR	11891	594	23,227	19	13,502	9,770	38,553	39.1	22	1.2	27.4	17.0	49.8	4.1	0.5
Erie	ASHTABULA R	11930	361	21,094	20	12,046	8,035	39,399	58.4	11	24.5	9.1	11.6	24.3	24.2	6.3
Erie	CONNEAUT CR	11936	487	19,755	21	1,826	17,705	23,298	40.5	21	27.7	7.4	12.1	16.8	28.6	7.5
Erie	OTTAWA CR	11794	440	19,264	22	12,492	6,637	36,674	43.8	19	1.4	7.5	51.1	27.5	11.4	1.2
Erie	EIGHTEENMILE CR	11959	305	13,825	23	7,938	5,377	28,103	45.4	17	27.4	0.0	10.7	19.8	36.9	5.2
Erie	MUDDY CR	11897	300	12,527	24	7,349	5,676	25,231	41.8	20	2.6	2.1	10.6	74.3	8.4	2.0
Erie	PINE R	11762	470	11,743	25	6,778	4,589	23,101	25.0	28	25.4	1.4	22.7	31.5	13.4	5.6
Erie	STONY CR	11793	359	9,906	26	5,896	3,851	20,172	27.6	25	3.8	36.8	30.5	21.6	6.3	1.1
Erie	GREEN CR	11909	284	9,692	27	5,691	3,507	18,133	34.1	23	2.4	0.1	13.5	70.5	11.3	2.2
Erie	ELK CR	11937	251	8,431	28	4,840	3,459	16,446	33.6	24	27.5	0.4	16.1	24.7	23.6	7.7
Erie	SWAN CR	11790	281	7,524	29	4,492	2,755	14,285	26.8	27	6.6	4.8	34.3	46.0	6.6	1.7
Huron	SAGINAW R	11747	16,258	487,521	1	25,944	459,427	533,504	30.0	4	7.2	46.1	19.1	16.5	8.9	2.2
Huron	THUNDER BAY R	11606	3,306	45,048	2	26,046	15,831	76,992	13.6	13	36.7	21.6	22.5	5.4	9.4	4.4
Huron	PINNEBOG R	11661	462	30,382	3	17,695	9,975	55,357	65.7	1	2.2	19.5	6.9	31.6	37.2	2.6
Huron	AU SABLE R	11619	5,265	22,540	4	4,800	17,086	27,098	4.3	20	44.1	3.1	45.0	1.4	3.4	3.1
Huron	RIFLE R	11652	972	19,352	5	11,259	7,232	33,941	19.9	7	24.6	22.7	27.4	7.5	14.5	3.3
Huron	CHEBOYGAN R	11593	3,900	17,740	6	11,405	5,797	31,750	4.5	19	47.8	5.7	35.0	2.8	5.3	3.5
Huron	AU GRES R	11647	745	16,751	7	9,676	7,587	37,208	22.5	5	24.9	7.1	18.2	16.4	26.6	6.9
Huron	PIGEON R	11660	399	16,517	8	9,746	6,890	31,368	41.4	3	2.4	3.5	6.6	39.2	45.1	3.2
Huron	WILLOW R	11664	263	14,372	9	8,410	4,810	21,951	54.6	2	4.0	0.0	5.4	40.1	47.2	3.3
Huron	PINE R	11578	739	13,639	10	8,007	4,985	26,174	18.5	9	38.6	44.4	11.9	0.3	2.5	2.3
Huron	MUNUSCONG R	12285	633	10,437	11	6,024	4,030	20,034	16.5	10	47.1	19.4	19.2	1.1	8.6	4.7
Huron	KAWKAWLIN R	11654	576	8,220	12	5,105	2,718	17,103	14.3	12	12.3	1.9	42.1	37.0	4.8	1.9

Huron	WHITNEY DRAIN	11643	374	5,021	13	2,922	1,916	10,392	13.4	15	38.6	2.9	25.9	9.8	16.0	6.9
Huron	SILVER CR	11640	385	4,564	14	2,688	1,802	9,795	11.9	16	27.6	25.9	31.8	3.5	7.6	3.6
Huron	PINE R, N BR	11658	255	3,827	15	2,361	1,630	7,310	15.0	11	12.9	3.8	44.5	26.9	9.9	1.9
Huron	OCQUEOC R	11584	361	3,633	16	2,117	1,462	6,836	10.1	17	55.8	0.0	28.7	6.8	5.1	3.6
Huron	CARP R	11583	396	3,420	17	2,007	1,284	6,836	8.6	18	80.9	0.0	17.1	0.1	1.0	0.9
Huron	WISCOGGIN DRAIN	11659	167	3,161	18	1,869	1,360	5,551	18.9	8	1.3	0.0	16.9	56.3	21.6	3.9
Huron	CHARLOTTE R	12284	152	3,050	19	1,767	1,123	5,922	20.1	6	50.0	0.0	17.6	2.6	18.6	11.2
Huron	LONG L CR	11588	176	2,388	20	1,385	781	5,590	13.6	14	47.6	0.0	24.0	12.6	10.5	5.3
Michigan	FOX R	11232	16,459	634,948	1	0	634,948	634,948	38.6	16	7.6	23.3	9.1	15.7	38.3	6.0
Michigan	GRAND R	11412	14,205	483,160	2	26,514	454,717	527,518	34.0	18	6.2	26.4	17.4	20.4	26.3	3.4
Michigan	ST JOSEPH R	11271	12,196	385,329	3	1	385,328	385,330	31.6	20	4.4	26.8	21.2	20.3	23.9	3.5
Michigan	KALAMAZOO R	11343	5,249	253,971	4	111,413	156,065	492,685	48.4	9	4.7	38.6	14.5	11.6	28.3	2.2
Michigan	MUSKEGON R	11488	7,047	105,162	5	61,776	37,402	198,052	14.9	32	23.1	13.1	29.9	9.2	20.0	4.7
Michigan	MENOMINEE R	11104	10,525	87,273	6	8,160	78,472	98,520	8.3	46	50.8	25.8	14.2	2.1	5.3	1.8
Michigan	MANISTEE R	11515	5,046	79,451	7	30,897	46,115	130,357	15.7	28	24.2	50.0	19.5	2.1	2.6	1.7
Michigan	MANITOWOC R	11019	1,395	64,767	8	8,435	55,710	75,828	46.4	13	3.8	20.7	4.4	11.9	52.0	7.1
Michigan	SHEBOYGAN R	11027	1,112	48,911	9	1,704	47,074	51,650	44.0	15	3.6	41.3	6.4	10.7	33.2	4.8
Michigan	MILWAUKEE R	11260	2,260	47,313	10	43	47,283	47,345	20.9	24	3.1	53.5	20.3	5.8	15.1	2.2
Michigan	W TWIN R	11016	444	43,641	11	25,464	17,144	74,656	98.4	1	4.0	2.8	4.4	13.4	65.9	9.5
Michigan	PORTAGE R	11247	904.4	41,916	12	15,311	27,041	63,954	46.3	14	7.6	24.7	38.1	24.3	3.9	1.4
Michigan	MANISTIQUE R	11543	3,778	41,298	13	11	41,289	41,308	10.9	39	62.0	19.9	16.3	0.5	0.6	0.7
Michigan	BLACK CR	11467	387	37,257	14	23,048	15,435	75,989	96.2	2	1.9	81.9	9.7	1.9	4.3	0.3
Michigan	PERE MARQUETTE R	11477	2,002	35,337	15	4,639	30,159	40,711	17.7	27	39.6	12.1	27.3	9.1	9.4	2.5
Michigan	ESCANABA R	11141	2,389	32,045	16	18,596	13,008	59,152	13.4	33	58.5	18.0	17.3	1.6	3.1	1.5
Michigan	OCONTO R	11051	2,558	30,662	17	329	30,316	31,213	12.0	36	21.6	5.7	11.6	13.9	39.7	7.3
Michigan	ROOT R	11258	506	23,526	18	4,770	18,707	30,119	46.5	12	4.9	32.6	23.6	20.8	15.4	2.7
Michigan	WHITE R	90615	1,373	21,542	19	12,543	7,689	42,198	15.7	29	24.4	21.2	25.0	12.5	14.5	2.5
Michigan	BLACK R	11332	666	21,263	20	12,453	8,028	41,652	31.9	19	9.7	10.4	15.4	18.4	43.2	2.9
Michigan	PESHTIGO R	11061	2,937	20,322	21	1,001	19,229	21,964	6.9	49	40.4	8.9	17.0	7.7	21.2	5.0
Michigan	KEWAUNEE R	11040	357	20,196	22	2,385	17,803	23,689	56.6	6	3.0	2.6	3.3	15.4	66.9	8.8
Michigan	DUCK CR	11050	391	18,822	23	2,740	15,665	23,085	48.1	10	4.9	1.1	14.8	16.4	54.5	8.3
Michigan	FORD R	11131	1,232	18,821	24	11,001	7,473	35,591	15.3	30	76.1	2.8	11.6	2.3	4.8	2.4
Michigan	AHNAPEE R	11037	302	18,676	25	10,894	7,627	31,354	61.8	5	6.7	3.7	5.0	17.5	59.5	7.6
Michigan	PENSAUKEE R	11045	379	17,662	26	10,337	6,777	33,830	46.6	11	9.0	0.8	7.1	16.3	57.8	8.9
Michigan	CEDAR R	11137	973	17,494	27	10,132	7,006	29,929	18.0	26	55.2	7.6	14.0	4.5	14.6	4.1
Michigan	MACATAWA R	11337	218	16,755	28	10,017	6,061	33,302	76.9	3	1.0	3.7	17.3	13.8	61.9	2.4
Michigan	E TWIN R	11015	345	16,717	29	9,824	7,241	32,244	48.5	8	4.5	23.1	7.5	10.9	47.8	6.2
Michigan	GALIEN R	11253	464	16,006	30	9,309	6,505	31,438	34.5	17	13.1	26.4	11.6	30.4	15.4	3.1
Michigan	PIGEON R	11026	216	15,462	31	9,055	6,055	27,545	71.6	4	3.9	1.9	7.1	16.3	62.3	8.6
Michigan	BOARDMAN R	11533	730	13,464	32	7,954	5,086	29,296	18.4	25	21.8	36.9	34.4	3.0	2.3	1.6
Michigan	PENTWATER R	11472	414	11,788	33	6,854	4,566	21,621	28.5	22	11.2	42.6	15.8	18.5	9.7	2.1
Michigan	PLATTE R	12403	494	11,049	34	6,795	4,741	24,734	22.4	23	13.0	70.9	12.4	1.6	1.3	0.8
Michigan	JORDAN/BOYNE R	11538	904.8	10,722	35	6,403	3,766	18,532	11.9	37	27.7	33.9	25.0	4.5	6.1	2.8
Michigan	SAUMICO R	11049	211	10,358	36	6,113	3,200	20,082	49.1	7	5.1	15.1	8.4	11.8	51.6	8.1
Michigan	WHITEFISH R	11152	818	8,447	37	4,970	3,357	14,837	10.3	40	81.7	0.0	13.0	1.2	2.6	1.5
Michigan	ELK R	12398	1,297.4	7,302	38	4,461	2,749	15,594	5.6	51	34.5	6.6	40.0	10.3	6.1	2.5
Michigan	BETSIE R	11526	610	6,030	39	3,765	2,211	12,062	9.9	41	42.3	0.2	48.5	3.1	3.4	2.5
Michigan	LITTLE SUAMICO R	11048	186	5,576	40	3,299	2,106	9,286	30.0	21	8.5	0.5	12.8	15.9	53.3	9.1
Michigan	STURGEON R	11164	572	4,576	41	2,684	1,696	8,869	8.0	47	80.4	0.0	18.6	0.2	0.3	0.6
Michigan	RAPID R	11159	347	4,499	42	2,636	2,084	8,364	13.0	34	80.5	0.0	13.3	1.6	2.9	1.7
Michigan	LINCOLN R	11484	278	4,220	43	2,481	1,360	7,866	15.2	31	27.5	0.0	29.3	19.4	19.2	4.6
Michigan	BEAR R	11541	295	3,589	44	2,122	1,442	6,838	12.2	35	49.8	0.0	34.2	3.6	7.8	4.5
Michigan	CEDAR R	12396	331.3	3,252	45	1,945	1,374	6,152	9.8	42	29.2	0.0	33.7	23.4	10.7	3.0
Michigan	BIG SABLE R	11487	466	3,099	46	1,857	1,093	5,124	6.7	50	50.1	0.0	32.1	7.7	7.7	2.3
Michigan	MILLECOQUINS R	11564	273	2,663	47	1,550	1,057	4,883	9.7	43	61.6	0.0	24.7	0.9	9.6	3.1
Michigan	FISHDAM R	11163	256	1,977	48	1,157	745	3,773	7.7	48	64.7	0.0	25.4	3.5	4.4	2.0

Michigan	CARP LAKE R	11542	155	1,732	49	1,013	548	3,714	11.2	38	54.9	0.0	29.8	3.0	7.8	4.4
Michigan	DAYS R	11161	158	1,484	50	874	560	2,803	9.4	44	75.5	0.0	19.2	1.4	2.4	1.5
Michigan	MILAKOKIA R	11565	174.0	1,472	51	868	577	2,710	8.5	45	83.4	0.0	15.8	0.0	0.2	0.6
Michigan	BREVOORT R	11563	234	809	52	491	254	1,464	3.5	52	75.1	0.0	23.5	0.1	0.5	0.8
Ontario	OSWEGO R	12147	13,182	543,010	1	318,080	204,198	1,053,202	41.2	6	13.3	29.1	8.9	16.6	27.2	4.9
Ontario	GENESEE R	12065	6,431	302,570	2	156,012	124,836	490,838	47.0	4	16.3	16.1	7.7	18.9	33.8	7.2
Ontario	BLACK R	12152	4,888	145,727	3	5,337	139,764	154,991	29.8	11	32.1	32.1	3.3	5.6	23.2	3.7
Ontario	TONAWANDA CR	11978	1,735	142,713	4	82,365	57,179	306,560	82.3	2	9.4	34.1	11.4	15.3	25.3	4.6
Ontario	OAK ORCHARD CR	11999	574	47,536	5	27,768	21,746	105,272	82.8	1	7.6	38.9	4.0	25.6	19.7	4.3
Ontario	SALMON R	12099	690	22,802	6	13,832	9,406	42,552	33.0	8	40.8	54.5	1.7	0.9	1.5	0.6
Ontario	EIGHTEENMILE CR	11994	238	15,889	7	9,515	5,955	32,811	66.8	3	6.8	56.6	8.2	14.2	11.1	3.1
Ontario	SANDY CR	12111	395	14,999	8	8,700	5,279	29,840	38.0	7	26.9	4.6	5.2	9.9	45.0	8.4
Ontario	IRONDEQUOIT CR	12546	440	12,147	9	1,477	10,805	14,114	27.6	12	10.1	0.0	67.6	13.4	7.0	1.8
Ontario	PERCH R	12564	252	11,620	10	6,781	4,767	19,641	46.1	5	19.2	0.0	4.9	11.7	53.9	10.3
Ontario	JOHNSON CR	11996	245	7,461	11	4,315	2,936	14,520	30.5	10	15.0	9.8	9.3	41.1	18.9	5.9
Ontario	S SANDY CR	12108	270	6,787	12	3,955	2,152	13,468	25.2	14	56.6	0.0	4.5	6.0	27.6	5.3
Ontario	STERLING CR	12095	209	6,526	13	3,810	2,749	11,791	31.3	9	22.2	0.0	6.6	22.7	41.2	7.3
Ontario	LITTLE SALMON R	12098	242	5,994	14	3,475	2,164	11,902	24.8	15	58.9	10.8	9.0	7.0	10.8	3.5
Ontario	WEST CR	12006	208	5,709	15	3,311	1,964	10,389	27.5	13	25.0	0.0	20.4	37.9	12.7	4.0
Superior	ST LOUIS R	12311	9,409	126,934	1	5,870	120,583	135,108	13.5	20	69.0	13.1	11.8	1.0	2.5	2.7
Superior	ONTONAGON R	10928	3,620	83,919	2	1,248	82,570	85,905	23.2	6	84.7	1.4	9.4	0.4	2.3	1.8
Superior	WHITE R	10897	2,488	74,127	3	73	74,048	74,262	29.8	4	74.7	2.2	7.6	1.8	9.6	4.1
Superior	NEMADJI R	10877	1,199	68,426	4	1,134	67,176	70,225	57.1	1	70.9	0.5	10.3	1.9	9.6	6.8
Superior	PORTAGE R	12321	2,349	26,677	5	15,886	10,130	51,903	11.4	26	74.2	7.6	13.1	0.9	2.8	1.3
Superior	TAHQUAMENON R	10994	2,138	19,148	6	11,241	7,772	34,174	9.0	35	77.6	5.8	15.3	0.3	0.4	0.6
Superior	PRESQUE ISLE R	10918	962	16,250	7	9,586	5,917	27,237	16.9	16	83.7	7.0	9.2	0.0	0.0	0.0
Superior	BLACK R	10915	669	14,777	8	8,654	4,946	29,127	22.1	8	76.2	13.7	9.6	0.0	0.2	0.3
Superior	CHOCOLAY R	10978	367	14,125	9	8,494	5,413	27,806	38.5	2	19.1	72.0	7.7	0.3	0.5	0.4
Superior	MONTREAL R	10912	696	11,092	10	9	11,082	11,106	15.9	18	64.6	20.2	12.2	0.6	1.5	0.9
Superior	BOIS BRULE R	10885	522	9,134	11	797	8,254	10,403	17.5	15	70.9	4.6	16.6	0.7	3.9	3.3
Superior	FISH CR	10893	390	8,214	12	4,736	3,802	14,931	21.1	10	47.9	0.0	10.2	3.6	29.7	8.5
Superior	AMNICON R	10882	343	8,111	13	4,753	3,464	14,387	23.6	5	82.0	0.1	7.0	1.1	5.9	3.9
Superior	BRULE R	10811	691	7,486	14	4,581	2,911	15,523	10.8	29	98.4	0.0	1.5	0.0	0.0	0.0
Superior	KNIFE R	10837	219	7,415	15	12	7,402	7,440	33.9	3	89.3	0.0	6.4	0.8	1.6	1.9
Superior	IRON R	10888	388	6,795	16	3,924	1,992	13,056	17.5	14	49.2	20.5	15.6	1.2	10.2	3.3
Superior	BEAVER R	10834	317	5,162	17	3,097	1,982	10,320	16.3	17	76.4	19.2	4.1	0.0	0.0	0.2
Superior	TEMPERANCE R	10822	471	4,911	18	3,020	1,746	8,838	10.4	30	98.6	0.0	1.4	0.0	0.0	0.0
Superior	WAISKA R	11009	368	4,802	19	2,787	1,727	8,976	13.0	21	56.4	0.0	24.1	1.5	10.6	7.5
Superior	IRON R	10923	264	4,692	20	2,806	1,709	11,794	17.7	13	92.5	2.2	5.2	0.0	0.0	0.2
Superior	PIGEON R	10809	1,483	4,676	21	2,889	2,215	8,098	3.2	44	99.0	0.0	1.0	0.0	0.0	0.0
Superior	BAPTISM R	10830	345	4,430	22	2,678	1,809	9,741	12.8	22	95.6	0.2	4.1	0.0	0.0	0.1
Superior	PORTAGE L SHIP CANAL	12320	182	4,203	23	2,449	1,391	7,912	23.1	7	31.4	36.7	28.7	0.7	1.7	0.8
Superior	CARP R	10974	184	3,978	24	2,328	1,506	7,658	21.6	9	34.8	41.5	23.2	0.0	0.1	0.4
Superior	POPLAR R	10819	287	3,537	25	2,152	1,457	6,317	12.3	24	97.8	0.0	2.1	0.0	0.0	0.0
Superior	CASCADE R	10818	309	3,536	26	2,175	1,343	6,201	11.5	25	99.3	0.0	0.7	0.0	0.0	0.0
Superior	TWO HEARTED R	10985	539	3,469	27	2,051	1,377	6,894	6.4	42	82.8	0.0	16.7	0.0	0.0	0.5
Superior	FLINTSTEEL R	10947	160	3,127	28	1,834	1,219	5,970	19.5	11	85.3	0.0	9.6	0.4	2.5	2.2
Superior	DEAD R	10973	424	3,017	29	1,773	1,206	5,996	7.1	38	71.2	0.0	27.9	0.0	0.0	0.8
Superior	TOBACCO R	10953	154	2,794	30	1,657	897	5,280	18.2	12	92.7	0.0	7.3	0.0	0.0	0.0
Superior	MANITOU R	10829	253	2,787	31	1,713	1,223	6,233	11.0	28	98.4	0.0	1.4	0.0	0.0	0.2
Superior	GOOSEBERRY R	10835	198	2,730	32	1,650	1,004	5,563	13.8	19	96.2	0.0	3.5	0.0	0.0	0.2
Superior	FIRESTEEL R	10948	233	2,426	33	1,431	909	4,387	10.4	31	85.3	0.0	13.0	0.1	0.6	0.9
Superior	AU TRAIN R	12273	314	2,324	34	1,375	775	4,460	7.4	37	76.6	0.0	18.0	0.9	2.2	2.3
Superior	SILVER R	10966	208	2,311	35	1,369	964	4,219	11.1	27	85.5	0.0	13.2	0.0	0.0	1.3
Superior	HURON R	10968	217	2,211	36	1,301	824	3,750	10.2	32	84.4	0.0	15.0	0.0	0.0	0.6
Superior	FLUTE REED CR	10810	172	2,179	37	1,332	909	4,187	12.7	23	98.5	0.0	1.5	0.0	0.0	0.0

Superior	CROSS R	10825	196	1,966	38	1,205	629	3,464	10.0	33	97.9	0.0	2.0	0.0	0.0	0.1
Superior	SIOUX R	10892	190	1,735	39	1,026	775	3,474	9.1	34	79.4	0.0	13.5	0.6	4.8	1.8
Superior	BETSY R	10993	230	1,613	40	947	677	3,219	7.0	39	78.7	0.0	18.0	0.0	0.0	3.4
Superior	DEVIL TRACK R	12302	193	1,489	41	900	600	3,085	7.7	36	94.7	0.0	5.2	0.0	0.0	0.1
Superior	SUCKER R	10984	200	1,372	42	805	478	2,820	6.9	40	79.3	0.0	19.7	0.0	0.0	1.1
Superior	YELLOW DOG R	12270	235	1,349	43	801	579	2,622	5.7	43	79.8	0.0	19.0	0.0	0.0	1.2
Superior	LITTLE TWO HEARTED R	10992	161	1,090	44	641	390	1,699	6.8	41	75.0	0.0	24.3	0.0	0.0	0.7
Ohio River	OHIO R	90796	373,067	27,713,307	1	0	27,713,307	27,713,307	74.3	1	18.1	21.1	8.9	23.7	17.4	10.8
Red River	RED R	38654	84,508	1,938,663	1	199,964	1,707,617	2,222,186	22.9	1	5.5	6.5	7.2	66.9	8.6	5.4
Upper Mississippi River	MISSISSIPPI R	65695	446,475	31,359,903	1	0	31,359,903	31,359,903	70.2	1	4.5	21.0	6.3	30.0	30.6	7.6

Table S6_TN. Total annual phosphorus and nitrogen loads and yields, with confidence intervals, for all tributaries to each lake with a drainage area greater than 150 square kilometers.
Tributaries to each Great Lake are ranked based on their relative loads and yields. A value of 1 indicates it has the largest load or relatively largest yield.
All loads and yields are adjusted to remove known spatial biases by only predicting areas that are not monitored. This results in very small standard errors and confidence intervals for basins for which a large percentage is monitored.

Supplemental Material to Robertson and Saad, 2011, Journal of the American Water Resources Association, Nutrient Inputs to the Laurentian Great Lakes By Source and River Basin Estimated Using SPARROW Watershed Models.

Yield is load per unit area of the HUC8 watershed.
[%, percent; CI, Confidence interval; kg, kilogram; km^2, square kilometer]

											Percent Contribution by Source					
Great Lake/River Basin	River Name	MRB Identification Number	Area (km^2)	Total Nitrogen Load (kg)	Total Nitrogen Load Rank	Standard Error in Nitrogen Load Estimate (kg)	Lower 95% CI Total Nitrogen Load (kg)	Upper 95% CI Total Nitrogen Load (kg)	Total Nitrogen Yield (kg/km^2)	Total Nitrogen Yield Rank	Atmosphere	Point sources	Additional agricultural sources	Farm fertilizers	Manure (confined)
Erie	MAUMEE R	11873	16,948	55,288,998	1	2,486,356	53,879,475	57,532,096	3,262	8	18.2	6.4	23.9	41.7	9.8
Erie	SANDUSKY R	11898	3,462	14,421,976	2	278,555	14,253,040	14,745,849	4,166	5	19.3	1.1	25.3	46.6	7.7
Erie	RIVER ROUGE	11782	1,208	11,558,685	3	9,617,448	5,552,744	24,286,857	9,570	1	2.6	96.8	0.2	0.3	0.1
Erie	PORTAGE R	11892	1,477	6,549,270	4	5,410,275	3,127,428	11,151,006	4,434	4	20.2	1.7	28.3	46.8	2.9
Erie	CUYAHOGA R	11920	2,091	5,410,101	5	16,693	5,400,015	5,420,583	2,588	9	13.5	79.5	1.7	3.8	1.5
Erie	HURON R	11910	1,078	2,546,312	6	252,339	2,385,240	2,827,723	2,362	10	18.9	6.0	25.0	43.3	6.8
Erie	TOUSSAINT CR	11891	594	2,452,507	7	2,027,385	1,305,663	4,582,105	4,131	6	20.1	0.8	27.8	48.9	2.4
Erie	RIVER RAISIN	11795	2,843	2,388,661	8	821	2,388,044	2,389,217	840	24	23.4	8.5	22.4	35.9	9.8
Erie	VERMILLION R	11913	678	2,334,837	9	21,713	2,320,604	2,358,055	3,445	7	21.4	3.8	22.3	41.8	10.7
Erie	BLACK R	11753	1,853	2,215,948	10	1,405	2,214,940	2,216,606	1,196	17	25.8	1.2	24.1	35.2	13.8
Erie	BLACK R	11914	1,230	1,621,687	11	234,031	1,455,248	1,799,470	1,318	15	25.0	13.3	16.8	33.7	11.3
Erie	MUDDY CR	11897	300	1,479,006	12	1,225,428	782,303	2,927,418	4,932	2	18.5	0.1	25.6	52.1	3.7
Erie	CLINTON R	12280	2,570	1,471,085	13	338,301	1,238,600	1,717,731	572	29	29.8	53.3	6.3	8.6	2.0
Erie	HURON R	12520	2,390	1,466,475	14	522,754	1,137,867	2,004,475	614	28	25.2	50.8	6.4	13.8	3.8
Erie	BUFFALO CR	11961	1,167	1,443,633	15	910,097	936,157	2,292,508	1,237	16	30.7	28.1	6.9	9.6	24.7
Erie	CATTARAUGUS CR	11946	1,449	1,410,215	16	245,044	1,251,671	1,613,673	973	20	42.5	3.7	14.3	10.6	28.9
Erie	GREEN CR	11909	284	1,332,869	17	1,114,096	645,737	2,296,066	4,693	3	18.7	0.0	26.8	49.9	4.6
Erie	GRAND R	11931	1,839	1,255,984	18	30,342	1,234,848	1,287,959	683	27	40.3	1.8	17.6	27.4	13.0
Erie	ROCKY R	11917	756	1,099,078	19	126,717	1,021,248	1,244,196	1,454	14	22.2	57.3	5.3	10.3	4.8
Erie	OTTAWA CR	11794	440	899,434	20	742,944	437,157	1,624,233	2,045	11	25.4	8.2	20.8	38.1	7.5
Erie	BELLE R	11763	577	555,752	21	460,785	269,716	1,065,162	962	23	31.8	13.5	22.2	25.4	7.1
Erie	STONY CR	11793	359	541,525	22	450,836	256,751	843,161	1,509	13	28.2	3.0	22.3	40.0	6.5
Erie	CONNEAUT CR	11936	487	528,923	23	437,582	258,593	834,424	1,086	19	42.6	11.8	17.4	12.5	15.8
Erie	CHAGRIN R	11927	673	495,785	24	105,752	435,750	576,583	737	26	41.4	38.9	3.9	12.4	3.4
Erie	SWAN CR	11790	281	429,351	25	353,905	169,810	691,572	1,529	12	27.4	1.4	21.2	46.2	3.7
Erie	ASHTABULA R	11930	361	409,059	26	337,827	197,122	626,101	1,133	18	41.0	6.5	18.3	19.4	14.7
Erie	PINE R	11762	470	348,270	27	288,742	158,406	613,861	742	25	42.2	2.9	25.1	24.3	5.5
Erie	EIGHTEENMILE CR	11959	305	295,696	28	243,614	125,864	542,942	970	21	48.0	0.0	13.4	13.2	25.4
Erie	ELK CR	11937	251	241,531	29	199,465	110,985	407,502	962	22	50.2	0.0	14.5	19.5	15.8
Huron	SAGINAW R	11747	16,258	11,852,946	1	519,023	11,516,730	12,315,865	729	7	25.8	21.8	16.9	27.6	7.8
Huron	PINNEBOG R	11661	462	1,097,872	2	917,128	560,845	2,048,769	2,374	3	16.0	1.7	20.3	39.0	22.9
Huron	PIGEON R	11660	399	1,033,268	3	863,953	471,564	1,765,658	2,589	2	15.7	0.9	21.2	39.6	22.7
Huron	KAWKAWLIN R	11654	576	693,088	4	553,891	326,947	1,162,843	1,203	5	29.1	0.2	23.7	43.4	3.6
Huron	THUNDER BAY R	11606	3,306	673,503	5	552,981	368,302	1,145,223	204	15	65.6	6.9	7.9	9.3	10.3
Huron	WILLOW R	11664	263	497,173	6	398,380	220,987	910,536	1,888	4	17.1	0.0	19.7	39.8	23.4

Huron	AU GRES R	11647	745	465,769	7	381,542	261,628	897,531	625	8	42.1	3.3	13.5	23.1	17.9
Huron	WISCOGGIN DRAIN	11659	167	440,564	8	357,430	211,204	703,350	2,640	1	18.5	0.0	28.2	44.6	8.8
Huron	AU SABLE R	11619	5,265	390,518	9	105,325	320,354	485,465	74	19	88.2	2.3	3.2	2.5	3.8
Huron	RIFLE R	11652	972	376,283	10	312,708	204,926	688,232	387	10	54.5	6.3	7.6	15.3	16.3
Huron	CHEBOYGAN R	11593	3,900	281,271	11	234,307	127,668	470,752	72	20	78.9	0.6	10.1	4.7	5.6
Huron	MUNUSCONG R	12285	633	241,153	12	200,320	126,473	521,250	381	11	62.0	7.0	20.9	1.7	8.4
Huron	PINE R, N BR	11658	255	239,598	13	200,840	108,540	365,578	940	6	34.6	1.0	17.7	37.7	9.0
Huron	PINE R	11578	739	196,334	14	161,358	110,842	349,353	266	13	77.2	5.8	12.4	0.8	3.8
Huron	WHITNEY DRAIN	11643	374	134,930	15	113,186	67,138	194,500	360	12	59.7	2.7	8.2	15.0	14.4
Huron	OCQUEOC R	11584	361	82,770	16	69,902	39,252	146,832	229	14	75.8	0.0	8.1	11.0	5.1
Huron	CHARLOTTE R	12284	152	80,826	17	67,830	36,012	161,474	533	9	51.3	0.0	35.8	2.4	10.5
Huron	SILVER CR	11640	385	77,701	18	62,770	37,589	133,586	202	16	59.4	18.9	4.7	7.3	9.8
Huron	CARP R	11583	396	73,017	19	61,591	33,150	137,117	185	18	96.5	0.0	2.1	0.2	1.2
Huron	LONG L CR	11588	176	34,923	20	28,800	18,788	90,211	198	17	68.3	0.0	6.2	16.8	8.8
Michigan	GRAND R	11412	14,205	12,709,791	1	920,113	12,165,239	13,509,185	895	14	25.2	15.3	15.8	25.9	17.8
Michigan	ST JOSEPH R	11271	12,196	10,613,665	2	22	10,613,645	10,613,680	870	16	24.5	10.1	18.9	30.7	15.8
Michigan	FOX R	11232	16,459	6,283,337	3	5	6,283,333	6,283,341	382	26	21.4	20.6	15.1	17.3	25.6
Michigan	KALAMAZOO R	11343	5,249	4,847,824	4	2,930,430	2,971,536	7,085,705	924	13	24.4	17.3	13.0	22.2	23.1
Michigan	MUSKEGON R	11488	7,047	2,714,116	5	2,233,385	1,261,471	5,203,536	385	25	51.5	2.9	12.3	14.7	18.6
Michigan	MENOMINEE R	11104	10,525	1,631,808	6	174,885	1,517,113	1,780,762	155	50	71.3	10.0	7.3	4.3	7.1
Michigan	MANISTEE R	11515	5,046	1,263,383	7	429,133	989,226	1,729,879	250	33	66.8	16.2	8.6	4.8	3.5
Michigan	PORTAGE R	11247	904.4	1,135,200	8	606,211	797,228	1,561,714	1,255	7	32.1	25.6	14.8	25.2	2.3
Michigan	SHEBOYGAN R	11027	1,112	1,045,669	9	86,671	986,327	1,131,680	941	12	19.3	11.8	17.0	17.3	34.6
Michigan	MANITOWOC R	11019	1,395	1,018,487	10	138,432	927,122	1,138,686	730	20	16.2	15.3	12.8	14.7	41.0
Michigan	MANISTIQUE R	11543	3,778	946,169	11	75	946,109	946,223	250	32	91.6	5.3	1.5	0.9	0.7
Michigan	MILWAUKEE R	11260	2,260	921,389	12	413	921,079	921,722	408	24	26.8	22.9	13.3	13.7	23.3
Michigan	BLACK R	11332	666	691,519	13	564,988	278,768	1,064,923	1,039	9	29.8	2.8	18.6	25.5	23.2
Michigan	ROOT R	11258	506	684,502	14	65,346	642,240	719,580	1,352	4	28.4	14.6	23.4	23.4	10.2
Michigan	PESHTIGO R	11061	2,937	649,626	15	35,666	627,827	673,464	221	40	51.6	5.6	13.6	10.5	18.8
Michigan	WHITE R	90615	1,373	617,152	16	509,406	290,971	1,231,040	449	23	48.1	8.4	11.8	18.6	13.1
Michigan	W TWIN R	11016	444	612,743	17	509,237	257,651	933,182	1,382	3	18.8	2.3	8.0	17.2	53.7
Michigan	OCONTO R	11051	2,558	585,350	18	15,804	575,538	600,014	229	36	38.3	5.5	14.8	14.8	26.5
Michigan	BLACK CR	11467	387	569,619	19	465,555	284,045	1,046,787	1,470	2	15.6	61.6	7.0	7.1	8.7
Michigan	PERE MARQUETTE R	11477	2,002	527,281	20	138,304	444,050	701,342	263	31	57.6	11.0	11.6	12.0	7.8
Michigan	ESCANABA R	11141	2,389	463,985	21	385,918	258,981	789,513	194	43	83.1	5.4	4.7	3.0	3.8
Michigan	E TWIN R	11015	345	452,662	22	370,844	229,671	787,884	1,313	6	19.4	7.0	7.8	17.2	48.5
Michigan	KEWAUNEE R	11040	357	431,358	23	42,761	406,982	456,384	1,209	8	16.5	2.8	7.6	19.4	53.7
Michigan	PIGEON R	11026	216	399,134	24	321,634	211,594	618,111	1,848	1	19.2	1.6	18.5	17.8	42.9
Michigan	GALIEN R	11253	464	378,069	25	294,428	229,036	637,288	816	17	32.5	9.8	20.6	30.5	6.6
Michigan	PENSAUKEE R	11045	379	332,728	26	276,598	152,196	596,837	877	15	23.2	0.3	17.6	18.6	40.2
Michigan	PENTWATER R	11472	414	304,648	27	253,264	170,864	517,910	736	19	35.2	2.9	19.5	32.4	10.0
Michigan	AHNAPEE R	11037	302	302,465	28	248,363	147,713	522,767	1,001	11	22.2	3.8	8.8	20.2	44.9
Michigan	MACATAWA R	11337	218	290,171	29	235,336	126,648	534,506	1,333	5	18.4	1.0	15.4	24.9	40.3
Michigan	FORD R	11131	1,232	277,728	30	233,011	135,071	475,876	225	38	83.6	0.8	6.6	3.9	5.1
Michigan	CEDAR R	11137	973	276,491	31	230,103	150,954	504,772	284	28	63.5	4.4	9.4	7.3	15.4
Michigan	DUCK CR	11050	391	272,986	32	46,503	240,555	309,688	698	21	19.9	1.0	18.3	19.8	41.0
Michigan	BOARDMAN R	11533	730	260,328	33	217,224	115,628	409,681	357	27	58.6	23.9	8.8	6.2	2.6
Michigan	SAUMICO R	11049	211	213,928	34	173,192	117,204	368,173	1,013	10	17.5	4.1	17.0	16.4	45.0
Michigan	JORDAN/BOYNE R	11538	904.8	169,390	35	138,221	85,824	240,745	187	46	57.3	10.4	17.5	8.3	6.5
Michigan	BETSIE R	11526	610	165,632	36	139,477	71,897	290,045	272	30	82.7	0.2	9.8	4.8	2.5
Michigan	WHITEFISH R	11152	818	160,533	37	133,229	82,969	298,775	196	42	91.8	0.0	3.3	2.0	3.0
Michigan	ELK R	12398	1,297.4	159,056	38	126,898	72,295	295,109	123	51	63.1	4.0	15.1	13.3	4.4
Michigan	LITTLE SUAMICO R	11048	186	147,179	39	119,051	78,506	287,379	793	18	21.9	0.0	19.4	18.9	39.7

Michigan	LINCOLN R	11484	278	144,301	40	117,500	72,360	238,195	518	22	50.2	0.0	18.0	19.6	12.2
Michigan	STURGEON R	11164	572	100,869	41	85,505	40,166	178,020	176	48	99.0	0.0	0.5	0.3	0.3
Michigan	CEDAR R	12396	331.3	93,890	42	76,486	43,170	194,768	283	29	52.2	0.0	18.7	23.2	5.9
Michigan	PLATTE R	12403	494	93,406	43	76,095	48,607	144,840	189	45	72.9	6.6	10.3	7.3	2.9
Michigan	BIG SABLE R	11487	466	89,642	44	73,109	43,457	181,630	192	44	74.4	0.0	9.6	9.8	6.2
Michigan	RAPID R	11159	347	79,258	45	66,671	35,027	123,268	229	37	89.0	0.0	5.2	2.8	3.0
Michigan	BEAR R	11541	295	68,921	46	57,943	34,772	124,167	234	34	71.0	0.0	16.1	5.4	7.5
Michigan	MILLECOQUINS R	11564	273	63,407	47	52,725	34,740	121,033	232	35	79.5	0.0	8.8	1.6	10.1
Michigan	FISHDAM R	11163	256	50,614	48	41,433	27,969	97,370	197	41	78.3	0.0	11.6	5.6	4.6
Michigan	CARP LAKE R	11542	155	34,200	49	28,831	17,649	66,031	221	39	75.3	0.0	12.4	4.7	7.5
Michigan	DAYS R	11161	158	29,467	50	24,042	14,372	52,415	187	47	90.2	0.0	4.6	2.5	2.7
Michigan	MILAKOKIA R	11565	174.0	29,234	51	24,195	13,431	43,426	168	49	99.5	0.0	0.3	0.0	0.2
Michigan	BREVOORT R	11563	234	16,311	52	13,035	7,483	26,392	70	52	98.3	0.0	0.9	0.1	0.7
Ontario	OSWEGO R	12147	13,182	9,400,412	1	7,528,976	5,253,829	14,776,564	713	12	34.7	23.7	9.8	11.8	20.0
Ontario	GENESEE R	12065	6,431	4,824,705	2	2,973,254	2,831,217	7,962,136	750	8	31.2	7.3	13.4	15.5	32.6
Ontario	BLACK R	12152	4,888	3,560,858	3	142,272	3,462,800	3,663,894	729	9	63.2	4.6	4.0	5.0	23.1
Ontario	TONAWANDA CR	11978	1,735	2,275,496	4	1,876,724	1,150,017	4,396,226	1,312	2	27.0	26.0	9.1	12.8	25.2
Ontario	OAK ORCHARD CR	11999	574	586,130	5	481,166	286,878	1,056,950	1,021	6	29.9	6.3	20.8	24.0	19.0
Ontario	SALMON R	12099	690	561,979	6	461,137	283,971	974,429	814	7	91.1	3.7	1.2	1.4	2.5
Ontario	SANDY CR	12111	395	530,630	7	438,714	283,759	999,470	1,345	1	51.6	1.7	10.7	6.0	29.9
Ontario	PERCH R	12564	252	304,169	8	248,973	132,949	555,998	1,208	4	50.2	0.0	5.0	7.4	37.4
Ontario	EIGHTEENMILE CR	11994	238	294,805	9	234,340	150,889	580,105	1,239	3	22.1	46.0	12.0	11.0	8.9
Ontario	S SANDY CR	12108	270	294,386	10	242,374	110,749	496,483	1,091	5	74.4	0.0	5.3	3.4	16.9
Ontario	LITTLE SALMON R	12098	242	175,682	11	146,657	82,035	309,837	727	10	81.9	3.9	2.9	4.2	7.1
Ontario	JOHNSON CR	11996	245	173,995	12	143,034	78,931	286,056	711	13	36.2	3.4	22.8	25.8	11.8
Ontario	STERLING CR	12095	209	151,498	13	122,557	73,750	247,421	727	11	42.7	0.0	11.8	15.7	29.8
Ontario	IRONDEQUOIT CR	12546	440	137,746	14	20,076	124,526	150,350	313	15	62.9	0.0	9.4	17.5	10.3
Ontario	WEST CR	12006	208	98,062	15	79,054	44,191	166,370	472	14	50.2	0.0	20.0	21.6	8.2
Superior	ST LOUIS R	12311	9,409	2,616,997	1	65,233	2,576,939	2,663,917	278	8	84.2	10.3	1.1	1.7	2.7
Superior	ONTONAGON R	10928	3,620	741,966	2	25,339	723,725	757,960	205	17	86.6	0.9	9.0	0.7	2.8
Superior	WHITE R	10897	2,488	601,522	3	2,174	600,150	603,430	242	11	84.8	1.1	3.3	2.5	8.2
Superior	TAHQUAMENON R	10994	2,138	404,031	4	337,901	226,119	747,649	189	21	94.9	3.7	0.5	0.6	0.3
Superior	NEMADJI R	10877	1,199	399,221	5	18,802	386,504	411,051	333	5	87.5	0.4	1.8	2.5	7.9
Superior	PORTAGE R	12321	2,349	353,884	6	289,213	148,571	641,620	151	28	80.8	7.4	7.2	1.6	2.9
Superior	PRESQUE ISLE R	10918	962	169,111	7	140,033	88,661	243,636	176	23	97.0	2.7	0.2	0.1	0.0
Superior	BLACK R	10915	669	167,186	8	139,548	99,095	311,411	250	9	78.0	17.2	4.3	0.1	0.4
Superior	MONTREAL R	10912	696	166,633	9	136,548	78,059	302,560	240	12	66.0	22.5	8.8	1.0	1.6
Superior	PIGEON R	10809	1,483	141,307	10	115,967	76,177	279,051	95	44	100.0	0.0	0.0	0.0	0.0
Superior	FISH CR	10893	390	138,533	11	114,163	56,168	264,367	355	1	69.4	0.0	4.3	4.3	21.9
Superior	IRON R	10888	388	129,182	12	105,852	57,559	225,175	333	4	80.4	3.6	1.5	2.4	12.2
Superior	WAISKA R	11009	368	129,071	13	108,087	65,990	210,732	350	2	66.0	0.0	25.3	1.6	7.1
Superior	AMNICON R	10882	343	116,866	14	97,575	56,958	205,772	340	3	92.4	0.0	1.4	1.6	4.6
Superior	BOIS BRULE R	10885	522	101,175	15	15,437	90,765	116,762	194	20	91.9	2.3	1.0	1.2	3.6
Superior	TWO HEARTED R	10985	539	81,994	16	67,041	39,299	121,745	152	27	100.0	0.0	0.0	0.0	0.0
Superior	BRULE R	10811	691	79,142	17	65,411	33,638	124,592	114	40	100.0	0.0	0.0	0.0	0.0
Superior	CHOCOLAY R	10978	367	75,558	18	63,304	34,099	113,618	206	16	80.6	13.3	2.0	1.6	2.4
Superior	KNIFE R	10837	219	69,187	19	274	68,946	69,444	316	6	96.6	0.0	0.3	1.3	1.8
Superior	TEMPERANCE R	10822	471	60,989	20	51,191	28,440	86,181	130	36	100.0	0.0	0.0	0.0	0.0
Superior	IRON R	10923	264	60,120	21	49,277	31,889	107,786	227	14	98.9	1.1	0.0	0.0	0.0
Superior	PORTAGE L SHIP CANAL	12320	182	53,960	22	44,720	29,086	87,676	297	7	47.4	43.4	5.1	1.7	2.4
Superior	DEAD R	10973	424	50,922	23	42,087	25,385	89,800	120	39	99.8	0.0	0.1	0.0	0.1
Superior	BETSY R	10993	230	46,471	24	39,290	20,379	74,530	202	18	100.0	0.0	0.0	0.0	0.0
Superior	FIRESTEEL R	10948	233	46,039	25	38,727	17,595	73,201	198	19	96.3	0.0	2.7	0.2	0.8

Superior	CARP R	10974	184	45,684	26	37,141	20,457	86,879	248	10	56.0	43.5	0.2	0.1	0.2
Superior	BAPTISM R	10830	345	44,855	27	37,049	20,460	88,723	130	35	99.4	0.3	0.2	0.1	0.0
Superior	SIOUX R	10892	190	42,495	28	35,530	22,499	65,485	223	15	93.2	0.0	0.6	1.0	5.2
Superior	BEAVER R	10834	317	42,446	29	34,751	22,571	85,750	134	32	99.1	0.6	0.2	0.1	0.0
Superior	CASCADE R	10818	309	42,130	30	35,266	17,468	68,472	137	31	99.9	0.0	0.0	0.0	0.0
Superior	AU TRAIN R	12273	314	41,376	31	33,533	19,681	67,462	132	33	94.8	0.0	2.0	1.2	2.0
Superior	SILVER R	10966	208	39,007	32	32,824	18,109	69,587	187	22	99.8	0.0	0.2	0.0	0.0
Superior	POPLAR R	10819	287	37,420	33	30,654	16,799	66,779	131	34	100.0	0.0	0.0	0.0	0.0
Superior	FLINTSTEEL R	10947	160	36,484	34	30,555	18,327	63,138	228	13	87.8	0.0	8.5	0.8	3.0
Superior	SUCKER R	10984	200	33,866	35	28,924	16,215	48,638	169	25	100.0	0.0	0.0	0.0	0.0
Superior	HURON R	10968	217	32,369	36	26,596	16,736	61,387	149	29	99.9	0.0	0.1	0.0	0.0
Superior	MANITOU R	10829	253	32,073	37	26,926	17,022	50,366	127	37	99.2	0.0	0.7	0.1	0.0
Superior	GOOSEBERRY R	10835	198	28,427	38	23,204	10,495	47,139	144	30	99.8	0.0	0.1	0.1	0.0
Superior	LITTLE TWO HEARTED R	10992	161	27,470	39	22,415	13,700	49,158	171	24	100.0	0.0	0.0	0.0	0.0
Superior	YELLOW DOG R	12270	235	25,534	40	21,119	13,072	43,257	108	41	99.8	0.0	0.1	0.0	0.1
Superior	CROSS R	10825	196	24,196	41	20,102	11,344	36,909	123	38	100.0	0.0	0.0	0.0	0.0
Superior	TOBACCO R	10953	154	23,921	42	20,216	11,012	34,747	155	26	100.0	0.0	0.0	0.0	0.0
Superior	FLUTE REED CR	10810	172	18,517	43	15,181	8,319	28,618	108	42	100.0	0.0	0.0	0.0	0.0
Superior	DEVIL TRACK R	12302	193	18,379	44	14,614	8,818	39,194	95	43	99.3	0.0	0.5	0.1	0.0
Ohio River	OHIO R	90796	373,067	357,231,370	1	0	357,231,370	357,231,370	958	1	35.2	12.8	14.3	27.3	10.4
Red River	RED R	38654	84,508	14,197,391	1	4,556,073	11,154,530	18,749,404	168	1	16.0	3.3	35.6	42.9	2.2
Upper Mississippi River	MISSISSIPPI R	65695	446,475	479,811,492	1	0	479,811,492	479,811,492	1,075	1	19.4	7.9	20.5	35.1	17.2
